# Supplementary material for: Identifying and prioritising future interventions with stakeholders to improve paediatric urgent care pathways in Scotland, UK: a mixed-methods study
Source: BMJ Open. 2023 Oct 12;13(10):e074141. doi: 10.1136/bmjopen-2023-074141 (PMC10582902; doi:10.1136/bmjopen-2023-074141)
Supplement: Supplementary data [file bmjopen-2023-074141supp001.pdf]

Supplementary file 1. FLAMINGO study detailed methods for data linkage and qualitative interviews

Study design and setting

The mixed-methods FLAMINGO study was undertaken in Scotland, where the NHS is organised into 14 geographically distinct terrestrial Health Boards, each responsible for healthcare provision to their region's population. The intervention development used a mixed methods phased approach and was target population centred, incorporating front-line health professional and patient perspectives collected through Public and Patient Involvement (PPI), stakeholders, and qualitative interviews. Patient and public involvement occurred throughout the project from inception to prioritisation of findings. Prioritising outcomes that matter to patient and staff participants (the target population) is important when designing an intervention with a congruent theory of change and will inform future decisions about the Population Intervention Comparator Outcome (PICO) for future evaluation design.[1] A programme theory of change was developed early in Phase 1 informed by the DAGIT process/model. [2] [Supplement 5] The DAGIT was referred to iteratively throughout the FLAMINGO study and incorporated into a final diagrammatic theory of change once the data analysis was complete [Supplement 5a]. The whole Flamingo team met monthly, the quantitative and qualitative sub teams met separately in between. An underpinning Systematic Review of interventions to reduce acute paediatric hospital admissions was undertaken by a FLAMINGO sub-team.[3] It was unclear at the start whether the systematic review would identify an intervention with sufficient promise to be adapted or whether developing a new intervention would be indicated. The findings were shared with the FLAMINGO team once qualitative data collection was complete and were incorporated into the intervention prioritisation process.

The study was approved by the NHS North of Scotland Research Ethics Service (REC reference: 19/NS/0134).

Data linkage methods

Study design

The data linkage study is reported in detail elsewhere.[4] Data from the following five clinical services were linked: Hospital admission details (Scottish Morbidity Record 01, SMR01); Out-of-Hours, Emergency Department and NHS 24 (all within the Unscheduled Care Datamart, UCD); general

practice data (provided by Albasoft, an NHS trusted third party). The period of was 2015-2017 and was determined by the availability of data from UCD (beginning April 2014) and the onset of the study (2019). A referral source was defined as the location (ED, GP, OOH or combinations thereof) where the child had a clinical record for the day of admission. When there was contact with more than one referral source on the day of admission, sources were placed in chronological order where possible (time of GP contact was not provided in the majority of records). When no referral source was identified we took two additional approaches (i) contact with a referral source the day before admission was sought (ii) we determined whether the child had been discharged from hospital on the same day as admission or the day before ("open access admissions").

Setting

Two NHS Scotland terrestrial health boards (Lothian, and Greater Glasgow and Clyde) have ED facilities staffed by paediatricians but in all other health boards, staff trained in emergency medicine see children in an ED. Three health boards have very limited in-patient paediatric facilities (Orkney, Shetland, and Western Isles) and no data from these boards were included in this analysis. Four health boards have children's hospitals (Greater Glasgow and Clyde, Grampian, Lothian, and Tayside).

Participants

Individuals <16 years with ≥ one urgent admission to hospital under the care of a medical paediatric team in Scotland in the calendar years 2015-2017. Children seen in and discharged from the ED or admitted under paediatric surgical, orthopaedic or dental teams were ineligible.

Ethical approval

The study was approved by the Public Benefit and Privacy Panel for Health and Social Care (reference 1718-0183). Data were analysed in the National Data Safe Haven of Scotland,[5] a secure repository accessed through a virtual private network by trained researchers.

Data sources

Scottish Morbidity Record 01, SMR01.[6] The SMR01 data are assessed for quality and completeness and are estimated to be 99% complete as of March 2018.[7] Data provided were: sex; ethnic group; decimal age; date of admission; date of discharge; admission type (emergency or elective); Scottish Index of Multiple Deprivations (SIMD) quintile; up to six ICD-10 diagnoses; Health Board of admission; specialty (paediatric medicine, paediatric surgery or paediatric dentistry). Time of admission was not available.

Unscheduled Care Datamart (ED, OOH and NHS24 data). This resource provides data from ED, OOH and NHS 24 (the latter including the Scottish Ambulance Service). All records have a valid CHI number.[8]

GP Data (Albasoft). GP data were made available through NHS Trusted Third Party (Albasoft) using a previously described method.[9] Briefly all Scottish general practices have software potentially accessible by Albasoft and practices can opt in and consent to share data with researchers via Albasoft. Typically, 15-20% of practices agree to provide data.[9] Where records were available, Read codes were used to distinguish clinical contact leading to a referral from administrative entries arising from clinical contact (e.g. a letter from ED after presenting there).

Bias

Completeness of data for SMR01 and the UCD ensures no bias. GP data were only available after practices opted in and this may introduce bias.

Study size

The study size was all individuals aged <16 years with an urgent admission to hospitals in Scotland in the calendar years 2015-2017.

Statistics

Descriptive statistics are provided for comparisons between groups. Multivariate logistic models were used to compare odds ratio (OR) for the ten most common composite diagnoses stratified by referral source. Composite diagnosis was defined as a group of very similar conditions, e.g. "asthma" included International Classification of Disease (ICD)-10 code J45.0 (predominantly allergic asthma), J45.9 (other and unspecified asthma) and J46X (Status Asthmaticus).[10] The benefit of using composite diagnoses is that they minimise the potential for variations in coding between units.[11] The following accounted for 46.4% of all urgent admissions in Scotland 2000-2013: asthma, bronchiolitis, convulsion (including febrile and afebrile convulsions), croup, gastroenteritis, upper respiratory tract infection (URTI), viral infection, tonsillitis, lower respiratory tract infections (LRTI) and admissions with a diagnosis of cough or wheeze or shortness of breath.[10] A stringent p value of <0.001 was used to indicate a meaningful association since the sample size was so large. Standard statistical software was used for the analysis (IBM® SPSS® version 24).

FLAMINGO qualitative interview methods

A qualitative exploratory approach was used to gain a better understanding of the experiences of parents and health professionals with regards to the circumstances around unscheduled SSAs of children (up to the age of 16) to hospital.

Qualitative setting

The FLAMINGO project team purposively identified five Health Boards to act as cases that would allow for maximal variation in characteristics such as deprivation, urban-rural, and hospital structure (with or without a dedicated children's hospital or short stay facility). Case selection was also informed by the quantitative data emerging from the data linkage exercise to allow for variation in numbers of unscheduled hospital admissions.

Participants and sampling

Health professionals were recruited by the team from a paediatric medical conference in Edinburgh in 2019, using their professional networks, and snowball sampling. Purposive sampling was used to invite health professionals working in primary care, GP out of hours services (OOH), and hospital emergency departments (ED)/ paediatric ED (PEDs) workplaces, NHS24, and the Scottish Ambulance service in the five case Health Boards, with responsibility for referring or receiving children for acute paediatric medical admission. Recruitment was unsuccessful from NHS24 and the Scottish Ambulance service.

Initial Patient and Public Involvement discussions revealed that many parents were unsure at what point in a hospital visit their child was officially categorised being admitted. The quantitative analysis of the data linkage analysed SSA where admission and discharge occur on the same calendar date.[4] To improve clarity when recruiting parents for interview, the definition of a SSA was therefore adapted to include stays in hospital lasting less than 24 hours. Parents were eligible to participate if they had experience of a SSA for their child with an acute medical illness and taken place within the past five years. SSAs for surgical or dental conditions were excluded.

The COVID-19 pandemic coincided with our qualitative recruitment phase and associated restrictions such as 'lockdowns' meant that previously planned in person recruitment methods at hospitals and places frequented by parents could not be undertaken. Alternative convenience sampling methods were adopted including online platforms and social media, press releases and advertisements in local newspapers. Parents responding to the invitation were sent a participant information sheet, study details and guidance on how to contact the main researcher (EK) to arrange a telephone interview.

Due to the challenges of recruiting to health research during the pandemic,[12] and the project's objective of ensuring maximal variation of participants, the recruitment press release in January 2021 invited eligible participants from outside the five Health Board case areas.

In addition, Phase 1 quantitative analysis and early interviews with families raised seizures as a particularly interesting reason for a SSA. Further purposive sampling and recruitment of health professionals and families with experience of seizure and findings will be reported separately.

Data collection

Interviews were conducted by two experienced qualitative researchers (EK, CM) with different backgrounds (health services research and paediatric nursing) and reflective field notes were kept. Semi-structured interviews were chosen for consistency between the two interviewers (EK, CM) whilst allowing interviewees to spontaneously raise issues. Separate semi-structured interview topic guides were developed for parents and health professional interviews by our multidisciplinary research team, with patient and public involvement (PPI) and drawing on existing literature on urgent short stay hospital admissions [the interview topic guides are in Supplementary files 2 and 3]. The topic guides included a specific question about recommendations for change regarding urgent SSAs. Health professionals were asked to propose potential solutions to improve urgent care pathways for children between home and hospital admission. Parents were asked questions relating to how their family's experiences could be improved in future.

The interviews took place between December 2019 and March 2021, pausing due to the COVID-19 lockdown between March and May 2020. Telephone interviews replaced face-to-face interviews. The COVID-19 pandemic was a context disruptor and data collected during this period might not be fully representative of typical SSA practices; therefore, interviewees were asked to speak about experiences pre-COVID. Interviewees were then asked specifically if and how they had observed differences during, or as a consequence of, the pandemic.

The duration of the interviews ranged from 18 to 62 minutes (median 27) and each interview was audio recorded, transcribed verbatim and anonymised.

Data analysis

Anonymised transcripts were uploaded to QSR NVivo 12 data management software (QSR International Pty Ltd., Version 12, 2019, Victoria, Australia) and were analysed using the Framework Method, used widely across health services research, which offers a systematic and flexible model for supporting qualitative thematic analysis.[13] It is particularly useful in situations where there is a desire to identify themes through making comparisons both within and between cases.[13] This was the aim of the current research which set out to explore the experiences of SSAs from the perspective of both parents and health professionals. The framework approach involves a five-stage iterative process of 1) data familiarisation; 2) identification of a thematic coding framework; 3) indexing and further refinement of the coding framework; 4) charting; and 5) mapping and interpretation to search for patterns and explanations in the data. During the initial data familiarisation stage, members of the FLAMINGO qualitative team (EK, CM, EF, PH) independently read a sample of four transcripts and field notes, two from each participant group (parents and health professionals), in depth and a high-level coding framework was devised by each member. The group met on several occasions to discuss overlaps and differences between the four coding indexes which were merged to form the first version of the coding framework. This framework, and the main thematic areas to focus on in the analysis, was then shared with the wider FLAMINGO team for additional comments and input. A subset of six transcripts were independently coded by EK and CM and the results compared to reveal considerable reliability in coding. Subsequently, minor changes to the codes were made, in order to further improve consistency.

The third indexing stage involved systematically applying the analytical coding framework to the remaining transcripts in NVivo. Line by line coding was carried out by three researchers (EK, CM, EF), with ongoing discussion and debate at weekly meetings. A framework matrix was developed to allow for organisation of the data samples (parents and health professionals) by themes to facilitate cross-case and within-sample comparisons. The FLAMINGO qualitative team reviewed the final themes to reach consensus in the mapping and interpretation of the data, thus enhancing rigour and trustworthiness.

Analysis of interview data relating to the outcomes and values that matter to parents and professionals when designing an intervention to improve care for children with SSAs is reported in Malcolm et al.[14]

References

1. Tovey D, Blaine C. How to clarify a clinical question 2022 [Available from: https://bestpractice.bmj.com/info/toolkit/learn-ebm/how-to-clarify-a-clinical-question.
2. Williams TC, Bach CC, Matthiesen NB, Henriksen TB, Gagliardi L. Directed acyclic graphs: a tool for causal studies in paediatrics. Pediatr Res. 2018;84(4):487-93.
3. Dick S, MacRae C, McFaul C, Rasul U, Wilson P, Turner SW. Interventions to reduce acute paediatric hospital admissions: a systematic review. Arch Dis Child. 2022;107(3):234-43.
4. Dick S, Kyle R, Wilson P, Aucott L, France E, King E, et al. Insights from and limitations of data linkage studies: analysis of short-stay urgent admission referral source from routinely collected Scottish data. Archives of Disease in Childhood. 2022:archdischild-2022-324171.
5. ISD Scotland. National Safe Haven [Available from: https://www.isdscotland.org/Products-and-Services/eDRIS/Use-of-the-National-Safe-Haven/.
6. ISD Scotland. General Acute Inpatient and Day Case - Scottish Morbidity Record (SMR01) [Available from: https://www.ndc.scot.nhs.uk/National-Datasets/data.asp?SubID=5.
7. ISD SG. Data Quality and Completeness for the Annual Acute Hospital Activity and NHS Beds Activity Publication 2018 [Available from: https://www.isdscotland.org/Health-Topics/Hospital-Care/Publications/2018-09-25/Data-Issues-and-Completeness-Sep18.pdf.
8. ISD Scotland. Unscheduled Care Datamart (UCD) [Available from: https://www.ndc.scot.nhs.uk/National-Datasets/data.asp?SubID=111.
9. Ellis DA, McQueenie R, McConnachie A, Wilson P, Williamson AE. Demographic and practice factors predicting repeated non-attendance in primary care: a national retrospective cohort analysis. Lancet Public Health. 2017;2(12):e551-e9.
10. Al-Mahtot M, Barwise-Munro R, Wilson P, Turner S. Changing characteristics of hospital admissions but not the children admitted-a whole population study between 2000 and 2013. European Journal Of Pediatrics. 2018;177(3):381-8.
11. Burns EM, Rigby E, Mamidanna R, Bottle A, Aylin P, Ziprin P, et al. Systematic review of discharge coding accuracy. J Public Health (Oxf). 2012;34(1):138-48.
12. Singh JA, Bandewar SV, Bukusi EA. The impact of the COVID-19 pandemic response on other health research. Bull World Health Organ. 2020;98(9):625-31.
13. Gale NK, Heath G, Cameron E, Rashid S, Redwood S. Using the framework method for the analysis of qualitative data in multi-disciplinary health research. BMC Medical Research Methodology. 2013;13(1):117.
14. Malcolm C, King E, France E, Kyle RG, Kumar S, Dick S, et al. Short stay hospital admissions for an acutely unwell child: A qualitative study of outcomes that matter to parents and professionals. PLoS One. 2022;17(12):e0278777.
