# Supplementary material for: Identifying and prioritising future interventions with stakeholders to improve paediatric urgent care pathways in Scotland, UK: a mixed-methods study
Source: BMJ Open. 2023 Oct 12;13(10):e074141. doi: 10.1136/bmjopen-2023-074141 (PMC10582902; doi:10.1136/bmjopen-2023-074141)
Supplement: Supplementary data [file bmjopen-2023-074141supp002.pdf]

The FLAMINGO project (**FL**ow of **A**d**M**issions in ch**I**ldren and you**NG** pe**O**ple)

Health Professional Interview Topic Guide

Can you start by confirming what your role is and your employer?
Probes – confirm role, employer, how long they have been in this role.

Experience of short stay admissions (SSAs)
We are interested in unplanned or emergency hospital admissions in children, particularly short stay admissions (SSA) where a child is admitted and then discharged from hospital on the same day (less than 24 hours).

Can you tell me about your experiences of SSAs of children?
Probes: frequency; typical reasons for them; referral sources – self, GP, NHS 111 etc; conditions, different decision makers (e.g. junior or senior paediatrician, A+E doctor, nurse practitioner) any other care pathways that we haven't talked about.

HOSPITAL STAFF ONLY. Please tell me about your experiences of receiving referrals for/admitting a child where you suspected they did not need to be admitted to hospital. (Probe – can you give me a specific example…)

COMMUNITY STAFF ONLY Can you think of any examples of where a child was admitted to hospital where you thought it was inappropriate/unnecessary?

Do you think that any hospital admissions of less than 24 hours could be safely avoided? (Probe – how?)

What involvement does the child and family have in the referral and admission decision-making process?

What role do the child and family's circumstances have in the referral and admission decision-making process, if any?

What role do other staff in the hospital (probe - nurses, managers) or in the community (e.g. information from the GP about the family) play. Can you think of any examples?

Some people we have talked to refer to appropriate and inappropriate admissions. What are your thoughts about these terms….? What do you consider to be an appropriate referral of an unwell child to a hospital for a short stay? Tell me about some of your experiences.

Readmissions
Can you think of occasions where a child was admitted to hospital for less than 24 hours and then required re-admission within a week or so for the same episode?

Tell me about your experiences of readmission (Probes: something that happens frequently/infrequently; when these types of readmissions do occur, what do you think are the reasons; factors which contribute to this; typical presentation – child/family)

Communication processes between the professional referring and the professional admitting child to hospital

Can you think of an example where communication, between the professional referring and the professional admitting child to hospital, went really well?

- What helped?

Can you think of an example where communication, between the professional referring and the professional admitting child to hospital, did not run so smoothly?

- What were the issues, what were the consequences, how could this be improved?

Question for Referrers only

Please tell me about your experiences of referring a child where you were uncertain if they needed to be admitted, to hospital.

- How do families that you know feel about referral to hospital and short admissions of < 24 hours
- What factors trigger a decision for you to refer a child [when you suspect a SSA might be the outcome]? Probe: for example, the family/parental circumstances

Thinking about the range of different children and families who access our emergency care service:

- Do family social issues have any impact on decisions (give examples)
- Do language issues (such as when English is not the first language) have any impact on decisions to admit children?
- Does the distance to hospital/transport issues/rurality have any impact on decisions to admit children?
- What are your reflections on health inequalities or deprivation and the potential impact on children's admissions, particularly SSAs?

Current situation – COVID-19 pandemic

- With the current situation with COVID-19 are you seeing a change in children attending hospital for unplanned visits? [Probe – which conditions etc]
- How do you think care of sick children at home is changing as a result of Covid-19? (prompts… are there any particular examples you have come across? Tell me more…. Any different examples…?)
- Are there any changes that you think will or should remain after the pandemic?

Final questions- looking forward

Thinking about moving forward and SSAs in children….

- What would be your recommendations for change regarding admissions of less than 24 hours?

- Are there any particular issues or health conditions you would highlight for the next stages of our research?
- What do you think will be happening with children's SSAs in five years' time?
- Is there someone you work with/a clinician you know of who has views very different to yours with respect to admitting children to hospital. Who do suggest we should be speaking to gain different perspectives on SSAs?
