# Supplementary material for: Identifying and prioritising future interventions with stakeholders to improve paediatric urgent care pathways in Scotland, UK: a mixed-methods study
Source: BMJ Open. 2023 Oct 12;13(10):e074141. doi: 10.1136/bmjopen-2023-074141 (PMC10582902; doi:10.1136/bmjopen-2023-074141)
Supplement: Supplementary data [file bmjopen-2023-074141supp003.pdf]

The FLAMINGO project (**FL**ow of **A**d**M**issions in ch**I**ldren and you**NG** pe**O**ple)

Family Interview Topic Guide

We are particularly interested in children who are unwell who spend a short time, less than 24 hours, in hospital. We are interested in infections and children who are unwell, so this research is not about accidents and injuries or operations.

Past medical history

- Brief history of child's health and past hospital admissions (purpose to determine the frequency of short stay admissions experienced and identify the admission that the family choose to talk about)

Explore the events and experiences leading up to the child's most recent or most memorable short stay hospital admission, asking families to tell their story from the start…

- What was worrying you about your child (ie. same as symptoms experienced in past? different? worrying?)
- What reasons do you recall for seeking advice?
- Who did you seek advice from? (ie. services and professionals such GP, pharmacist, NHS 24 111, internet, minor injuries unit, A&E; family; friends; other)
- Can you tell me what you understand by the service provided by: Emergency Departments [probe A&E vs minor injuries], NHS 111 / NHS 24?
- What happened next, what were the outcome/s of these initial consultations?
- What other challenges do you face as a family when your child is sick? [Probe: No money in phone, no access to technology, transport, other childcare.]

Explore the family's experience of a zero-day hospital admission,

- Can you tell me what happened from when you walked through the door of the hospital?
- Who did you see first, what happened next, can you recall any feelings experienced?
- Who made the decision that your child would be admitted to hospital? What involvement did you and your family have in any decision making related to the admission?
- What thoughts and feelings did the family have about the decision to admit your child to hospital?
- How often did you move to a different area in the hospital?
- How confident were you in the care team who looked after your child? How consistent was the information that you received from different people?
- Would anything have made a difference to your experience?

Explore the family's experiences of going home after the admission…

- Can you tell me about how the decision was made to discharge your child from hospital and send them home? What was your involvement in that decision making?

- What happened once you were back at home? Did any questions or concerns arise the first few days after discharge from hospital? Was there anything you felt unsure about or wanted further advice on?
- Was your child readmitted for the same illness within two weeks of being sent home? If so how did you feel about this?
- How did your experience change what you would do if a similar situation occurred again?

Explore how the family's experiences could be improved in future…

- How satisfied were you with the process of accessing services for your child?
- Tell me about communication with professionals involved in your child's care?
- What (if anything) would they have liked to be different about your story?
- What other stories have you heard from family and friends about short-stay admissions?
- Some people we have spoken to think some short-stay admissions are unnecessary… what are your thoughts about this?
- What are your views about telephone and face to face consultations for sick children?
- Could technology make a difference – e.g. video call to a children's specialist doctor?

Reflect on current situation – COVID-19 pandemic

- How has the current situation with COVID-19 changed how you would access healthcare for your child?
- How do you feel about monitoring and caring for your child at home?
- How do you think this will impact on how you access healthcare in the future?

Closing the interview…

Thank the parents for sharing their experiences.

Explain how they can get in touch with the research team if they have any thoughts or concerns after the interview.

Explain how they will find out about the results of the research.
