# Supplementary material for: Identifying and prioritising future interventions with stakeholders to improve paediatric urgent care pathways in Scotland, UK: a mixed-methods study
Source: BMJ Open. 2023 Oct 12;13(10):e074141. doi: 10.1136/bmjopen-2023-074141 (PMC10582902; doi:10.1136/bmjopen-2023-074141)
Supplement: Supplementary data [file bmjopen-2023-074141supp004.pdf]

Parent and carer information sheet version 6 26-11-19

FLAMINGO

FLow of AdMissions to hospital in chIldren and younG peOple

Experience of unwell children needing a short stay in hospital (less than 24hrs)? We would like to talk with you.

We would like to invite you to take part in a research project. The project is led by Professor Steve Turner of the University of Aberdeen in partnership with the University of Stirling and Edinburgh Napier University. This part of the research is being carried out by the Nursing Midwifery and Allied Health professions (NMAHP) Research Unit at the University of Stirling.

Before you decide, it is important for you to understand why the research is being done and what it will involve. Please take time to read the following information carefully and to discuss it with others if you wish. Ask us if there is anything that is not clear or if you would like more information. Take time to decide whether you wish to take part. Thank you for reading this.

What is the purpose of the research?

The aim of this project is to understand why children are admitted to hospital for stays of less than 24 hours. To answer this question we are collecting lots of data about hospital admissions and speaking to doctors and nurses. We are also talking to parents about their experience of having a child being admitted to hospital.

Who can take part?

Parents or carers whose child has ever been admitted to hospital for a stay of less than 24 hours for an illness such as a chest infection or fever and not for a broken bone.

Do I have to take part?

No. It is up to you to decide whether you want to take part or not. If you do decide to take part, you will be asked to sign a consent form. You will still be free to stop taking part at any time and without giving a reason. If you stop taking part, or decide not to take part, this will not affect the standard of health care you and your child receive.

What happens if I take part?

You will take part in a short (about 20-45 minutes) interview where a researcher will ask questions about your experiences of your child being admitted to hospital. We can do the interview in a place of your choice, for example, in your home or in a meeting room at the

King E, *et al. BMJ Open* 2023; 13:e074141. doi: 10.1136/bmjopen-2023-074141

university. If you wish, your partner or someone close to you to be present. If your child is 6 years of age or older and they want to take part, then they can take part in the interview with you at the same time. We will give your child age appropriate information on the study and ask them to complete their own consent form.

We might ask the person who referred your child to hospital to also take part in this study. We will audio-record the interview so that we can transcribe what you said and then analyse it. The person who is transcribing (i.e. putting into written text what you have said) will be a third party (i.e. not one of the research team) who will not know who you are. You will have up to 7 days after your interview to let us know if you no longer want us to use your comments. Some parents may find the interview upsetting. If you get upset our experienced interviewers will encourage you to take breaks, you can stop at any time and not take part any more.

What are the possible benefits of taking part?
There will be no direct personal benefit in taking part in this study. However, we hope that your views will be useful in improving the care of children in the future.

What are the possible risks of taking part?
There is a possibility that some parents may become upset. Our staff are trained and very experienced in providing support during interviews

Will my participation in the study be kept confidential?
Yes. We will keep your personal information, such as your name, address and telephone number, confidential in records held in Stirling University. We will record the interview so that your comments can be transcribed and analysed afterwards. We might quote something you said in project publications but we will make sure you cannot be identified. Your comments will not be linked to your name or other personal information and will be stored securely. All data will be managed in line with current data protection guidelines.

If you tell us something during the study which gives us cause for concern about your safety or that of your child, then we have a duty of care to take appropriate action. This could mean that we cannot keep what you told us confidential.

How will we use information about you?
We will need to use information from you for this research project.
This information will include your name and contact details. People will use this information to do the research or to check your records to make sure that the research is being done properly.

People who do not need to know who you are will not be able to see your name or contact details. Your data will have a code number instead. We will keep all information about you safe and secure.

King E, *et al. BMJ Open* 2023; 13:e074141. doi: 10.1136/bmjopen-2023-074141

Once we have finished the study, we will keep some of the data so we can check the results. We will write our reports in a way that no-one can work out that you took part in the study.

What are your choices about how your information is used?
You can stop being part of the study at any time, without giving a reason, but we will keep information about you that we already have.
We need to manage your records in specific ways for the research to be reliable. This means that we won't be able to let you see or change the data we hold about you.

Where can you find out more about how your information is used?
You can find out more about how we use your information

- at www.hra.nhs.uk/information-about-patients/
- our leaflet available from the research team
- by sending an email to dpo@abdn.ac.uk, or by ringing us on 01224-272596.

Who has reviewed this project?
The North of Scotland Research Ethics Committee and specialists in research and health care have reviewed this project.

Who is funding this study?
The Chief Scientist's Office of the Scottish Government.

What if I want to complain?
If you have a concern about any aspect of this project, you should ask to speak to the researchers first 01786-466277. If you are still unhappy and wish to complain formally, you can do this by contacting the University of Aberdeen Research Governance Team via researgovernance@abdn.ac.uk or by phoning 01224-554362.

What do I do if I want to take part?
If you are interested in taking part please call the researcher Emma King at the University of Stirling on 01786-466277, or you can email us on flamingo@stir.ac.uk

Contact details for queries: Dr Emma France. Telephone 01786-466421. Email: emma.france@stir.ac.uk

King E, *et al. BMJ Open* 2023; 13:e074141. doi: 10.1136/bmjopen-2023-074141

Healthcare professional information sheet version 5 12-11-19

FLAMINGO

FLow of AdMissions to hospital in chIldren and younG peOple

Experience of unwell children needing a short stay in hospital (less than 24hrs)? We would like to talk with you.
We would like to invite you to participate in a research project. Before you decide, it is important for you to understand why the research is being done and what it will involve. Please take time to read this following information carefully and to discuss it with others if you wish. Ask us if there is anything that is not clear or if you would like more information. Take time to decide whether you wish to take part. Thank you for reading this.

What is the purpose of the research?
The aim of this study is to understand why children are admitted to hospital. To achieve this aim we are using both quantitative and qualitative methods. We are keen to talk to clinicians or practitioners who refer and receive acute paediatric medical cases, so that we can gain a clinician's perspective on why children are admitted to hospital.

Why have I been chosen?
We have approached you about this study because you are a clinician or practitioner who is involved in acute paediatric admissions to hospital.

Do I have to take part?
No, it is up to you to decide whether or not to take part.

What happens if I take part?
You will take part in a short (about 10 minute) telephone interview where a researcher will ask questions about the decision making which leads to a child being admitted to hospital or discharged home.

What are the possible benefits of taking part?
There will be no direct personal benefit in taking part in this study. However, we hope that your views will be useful in improving the care of children in future.

What are the possible risks of taking part?
There are no risks.

Will participation in the study be kept confidential?
Yes. We will keep your personal information, such as your name and telephone number, confidential. We will record the interview so that we can then transcribe and analyse it. We might quote something you said in project publications but we will make sure you cannot be identified. Your comments will not be linked to your name, workplace name or other personal information and will be stored securely. All data will be managed in line with current data protection guidelines.

King E, *et al. BMJ Open* 2023; 13:e074141. doi: 10.1136/bmjopen-2023-074141

The University of Aberdeen is the sponsor for this study based in the United Kingdom. We will be using information from you in order to undertake this study and will act as the data controller for this study. This means that we are responsible for looking after your information and using it properly. The University of Aberdeen will keep identifiable information about you for 10 years after the study has finished.

Your rights to access, change or move your information are limited, as we need to manage your information in specific ways in order for the research to be reliable and accurate. If you withdraw from the study, we will keep the information about you that we have already obtained. To safeguard your rights, we will use the minimum personally-identifiable information possible.

Your local NHS board will keep your name, and contact details confidential and will not pass this information to University for Aberdeen. Your NHS board will use this information as needed, to contact you about the research study, and make sure that relevant information about the study is recorded for your care, and to oversee the quality of the study. Certain individuals from University of Aberdeen and regulatory organisations may look at your research records to check the accuracy of the research study. The University of Aberdeen will only receive information without any identifying information. The people who analyse the information will not be able to identify you and will not be able to find out your name, or contact details. Your local NHS board will not keep identifiable information about you from this after the study has finished.

You can find out more about how we use your information at http://www.abdn.ac.uk/privacy

Who has reviewed this study?
The study has been reviewed by the North of Scotland Research Ethics Committee and also specialists in the field.

What if I want to complain?
If you have a concern about any aspect of this project, you should ask to speak to the researchers first on 01786-466277. If you are still unhappy and wish to complain formally, you can do this by contacting Professor Jayne Donaldson, the Dean of the Faculty of Health Sciences and Sport, University of Stirling on 01786-466340 or jayne.donaldson@stir.ac.uk.

You have the right to lodge a complaint against the University of Stirling regarding data protection issues with the Information Commissioner's Office (https://ico.org.uk/concerns/).
The University's Data Protection Officer is Joanna Morrow, Deputy Secretary. If you have any questions relating to data protection these can be sent to data.protection@stir.ac.uk.

What do I do now?
If you are interested in taking part, please contact the researcher Dr Emma King at the University of Stirling on 01786-466277. Email flamingo@stir.ac.uk

If you require any further information, Dr Emma France. Telephone 01786-466421. Email: emma.france@stir.ac.uk

King E, *et al. BMJ Open* 2023; 13:e074141. doi: 10.1136/bmjopen-2023-074141
