# Supplementary material for: Identifying and prioritising future interventions with stakeholders to improve paediatric urgent care pathways in Scotland, UK: a mixed-methods study
Source: BMJ Open. 2023 Oct 12;13(10):e074141. doi: 10.1136/bmjopen-2023-074141 (PMC10582902; doi:10.1136/bmjopen-2023-074141)
Supplement: Supplementary data [file bmjopen-2023-074141supp005.pdf]

DAG model Nov 2021

Purpose and population

Intervention development in the pathway for unscheduled acute illness in children, access to care and admissions to hospital

Whole Systems and Multi-Stakeholder Approach to Intervention Development

Systematic Review Evidence

Programme Theory

Context - macro
- Rising admissions
- Covid pandemic
- NHS staff shortages and workload
- Health Inequalities

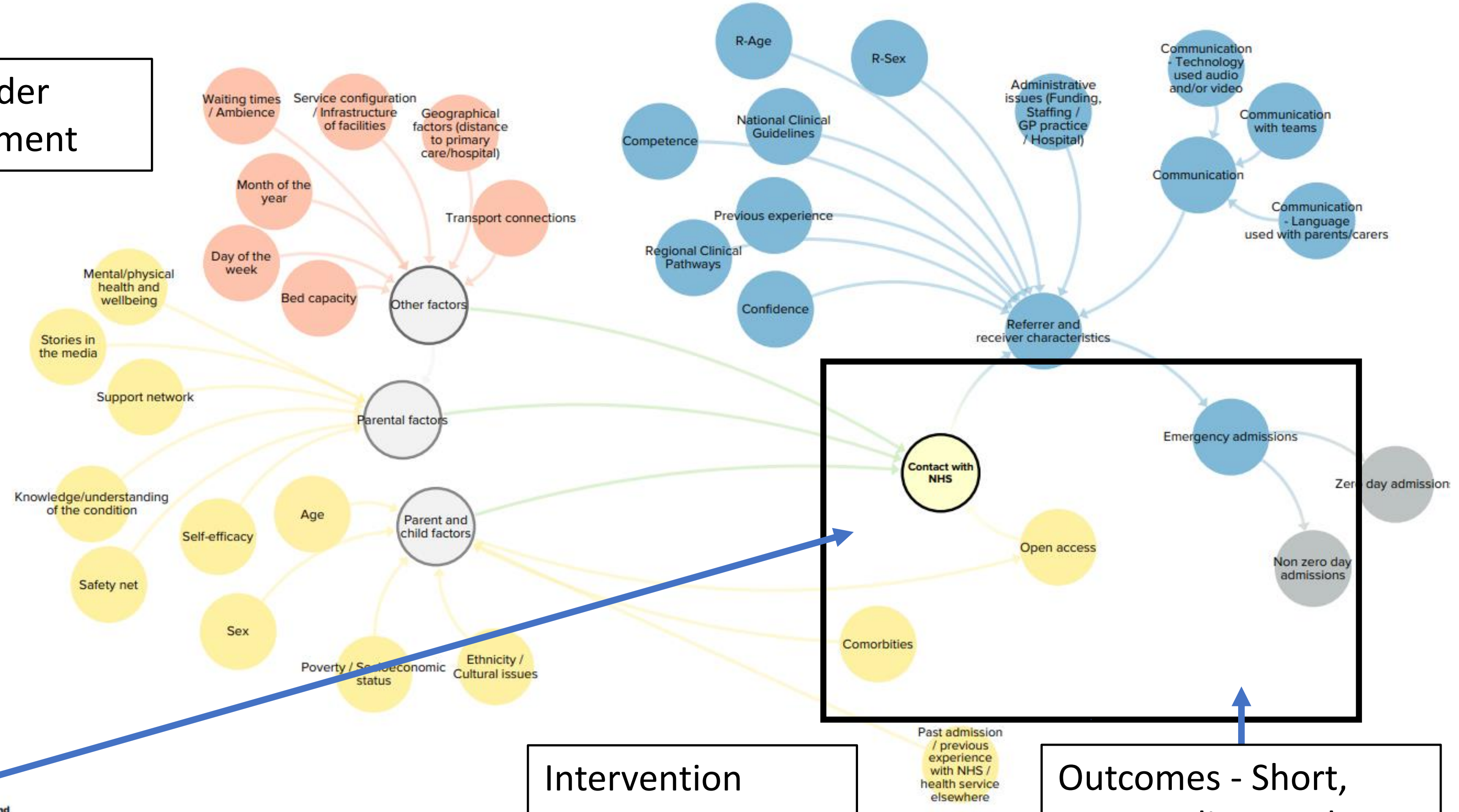

Right method, time, place, person, process,

Family, Staff and Society Values

Intervention Components, Processes and Mechanisms: TIDieR

Outcomes - Short, Intermediate and Long Term that matter to children, families, staff, society
