# Supplementary figures and images for: Identifying and prioritising future interventions with stakeholders to improve paediatric urgent care pathways in Scotland, UK: a mixed-methods study
Source: BMJ Open. 2023 Oct 12;13(10):e074141. doi: 10.1136/bmjopen-2023-074141 (PMC10582902; doi:10.1136/bmjopen-2023-074141)

FLAMINGO Revised Logic Model post data analysis

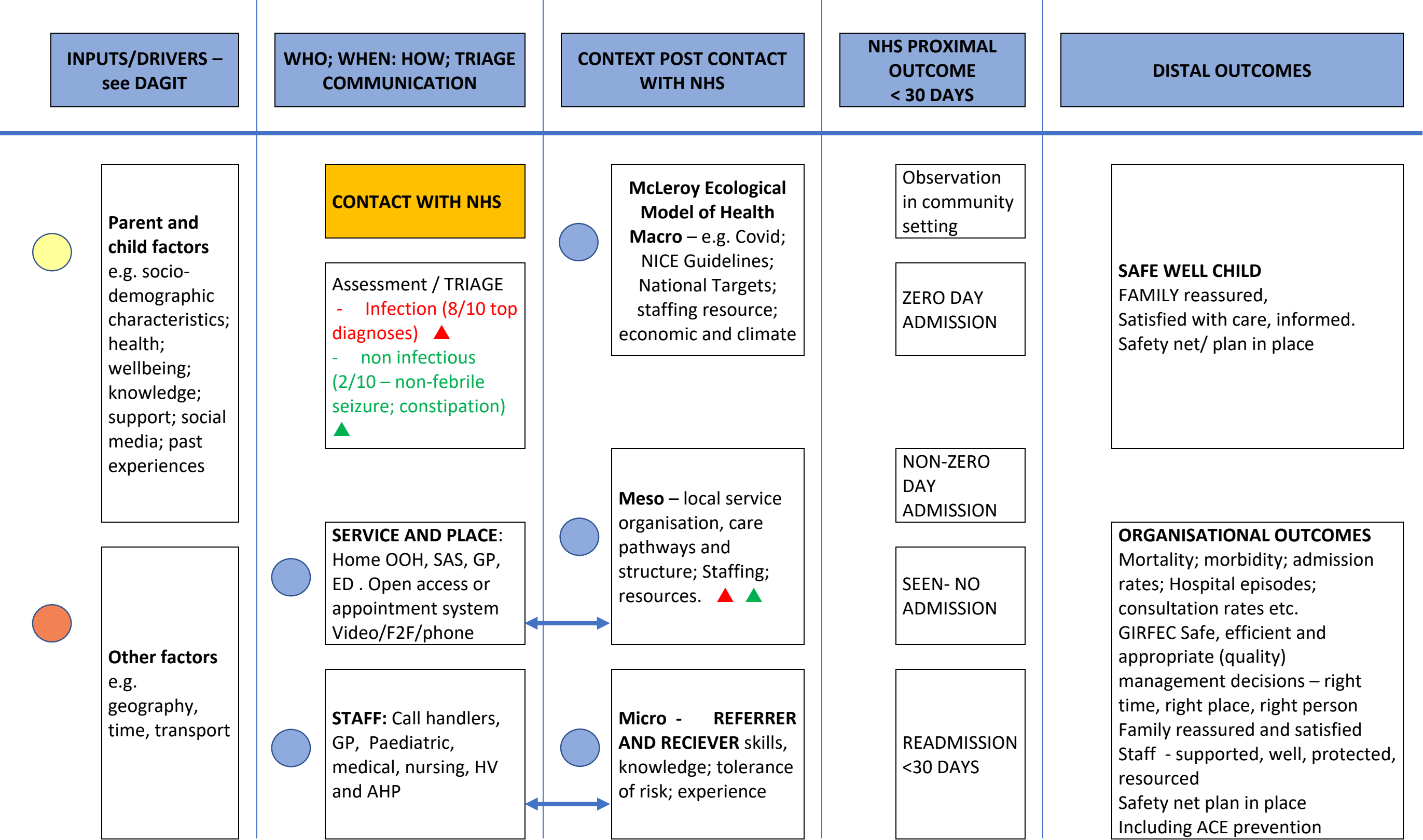

Supplement: Supplementary data [file bmjopen-2023-074141supp006.pdf]
