# Supplementary material for: Identifying and prioritising future interventions with stakeholders to improve paediatric urgent care pathways in Scotland, UK: a mixed-methods study
Source: BMJ Open. 2023 Oct 12;13(10):e074141. doi: 10.1136/bmjopen-2023-074141 (PMC10582902; doi:10.1136/bmjopen-2023-074141)
Supplement: Supplementary data [file bmjopen-2023-074141supp007.pdf]

Supplementary file 6. Flamingo stakeholder event methods and materials

Purpose/objective

To present project findings and to discuss prioritisation of potential interventions.

Setting

The five-hour event was held on 3 September 2021 at Edinburgh Zoo, a location accessible for most stakeholders. Travel expenses were offered to families and health professionals who had taken part in interviews.

Participants

The face-to-face and online attendees included parent and health professional interviewees, health professionals, Principle Investigators for the case study sites and a purposive sample of professional contacts of the team with relevant experience, a representative from the study funder the Chief Scientist Office, and representatives from stakeholder organisations e.g. charities and parent groups.

Agenda

10-10.15 Introduction

10.15-11.00* What we found

11-12ish Group discussions followed by time to explore the zoo

12.30-1.15 Lunch

1.15-2.15* Opportunities for change

2.15-2.45 Group discussions

2.45-3.00 Wrap up

*tea and coffee available

Procedures & data collection

The Stakeholder Event discussed FLAMINGO project findings in the morning and prioritisation of potential new interventions in the afternoon. Attendees were split into six groups, five in-person and one online. In-person attendees were seated at pre-allocated tables of five attendees with a mix of families, health professionals and other stakeholders (e.g. third sector or government representatives). One member from the FLAMINGO team (SD, EF, EK, ST, or PW) facilitated each

table's discussion. An online group took place via Microsoft Teams© facilitated by two remote members of the FLAMINGO team (CM, RK). Facilitators took written notes during or after the discussion and audio-recorded all discussions to assist accurate note-taking. To ensure all contributions were captured, attendees were also asked to write any ideas they had on post-it notes which were collated and recorded. After the morning presentations, facilitators asked each group three questions to stimulate discussion about the study findings:

1. What is the most important thing you have heard this morning?
2. What surprised you the most?
3. What did not surprise you?

In the afternoon the project team presented the seven potential future health systems interventions Subsequently, the groups were asked the following two questions to spark discussion about prioritisation of interventions:

1. If you were in charge, what would you change first and why?
2. Imagine you are in charge five years after your first choice has been in action. What problems would people come to you with about your "new" service?

The second question drew on Klein and Kahneman & Klein's [1, 2] 'pre-mortem' method to improve decisions, which asks groups to prospectively imagine the future and predict what has gone wrong; the pre-mortem can overcome the suppression of dissenting views and increases identification of risks from a project's outset.

Finally, attendees were asked to individually rank the priority of seven interventions during the stakeholder event on a pro-forma, where 1 was the highest priority and 7 was the lowest. Online participants accessed an online pro-forma.

Analysis

Notes taken during discussions were checked for accuracy and completeness against the audio-recordings where possible, however, due to high background noise in two recordings facilitators produced more detailed notes of the discussions from memory and post-it notes were checked and incorporated into the discussion notes. Notes were uploaded into NVivo and were coded (by EF and EK) under broad codes for each of the seven interventions with separate codes for confirming or disconfirming perspectives, plus a further code for other interventions attendees suggested. Codes

were read repeatedly and themes identified. For the intervention priority ranking exercise, scores were summed and the average rank calculated for each of the seven interventions.

1. Klein G. Performing a Project PreMortem. Harvard Business Review. 2007.
2. Kahneman D, Klein G. Conditions for intuitive expertise: A failure to disagree. American Psychologist. 2009;64(4):515-26.
