# Supplementary material for: Identifying and prioritising future interventions with stakeholders to improve paediatric urgent care pathways in Scotland, UK: a mixed-methods study
Source: BMJ Open. 2023 Oct 12;13(10):e074141. doi: 10.1136/bmjopen-2023-074141 (PMC10582902; doi:10.1136/bmjopen-2023-074141)
Supplement: Supplementary data [file bmjopen-2023-074141supp008.pdf]

BMJ Publishing Group Limited (BMJ) disclaims all liability and responsibility arising from any reliance placed on this supplemental material which has been supplied by the author(s)

FLAMINGO
Flow of hospital admissions in children and young people - Introduction

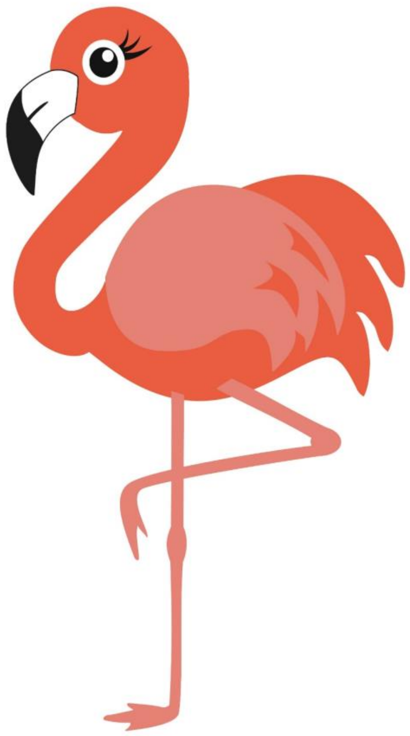

CHIEF
SCIENTIST
OFFICE

BMJ Publishing Group Limited (BMJ) disclaims all liability and responsibility arising from any reliance placed on this supplemental material which has been supplied by the author(s)

Good morning. Welcome

- Housekeeping
- Thanks
- Why are we meeting?
- Plan for the day
- Introductions

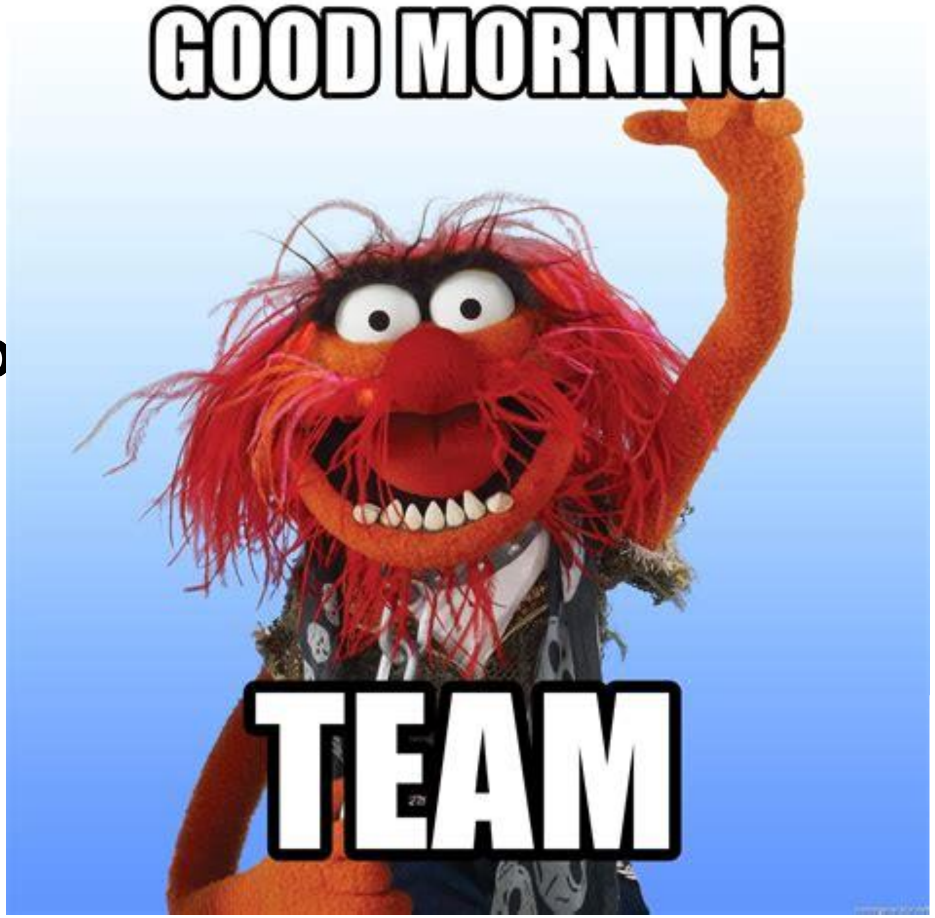

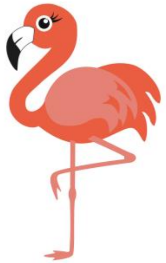

CHIEF SCIENTIST OFFICE

Housekeeping

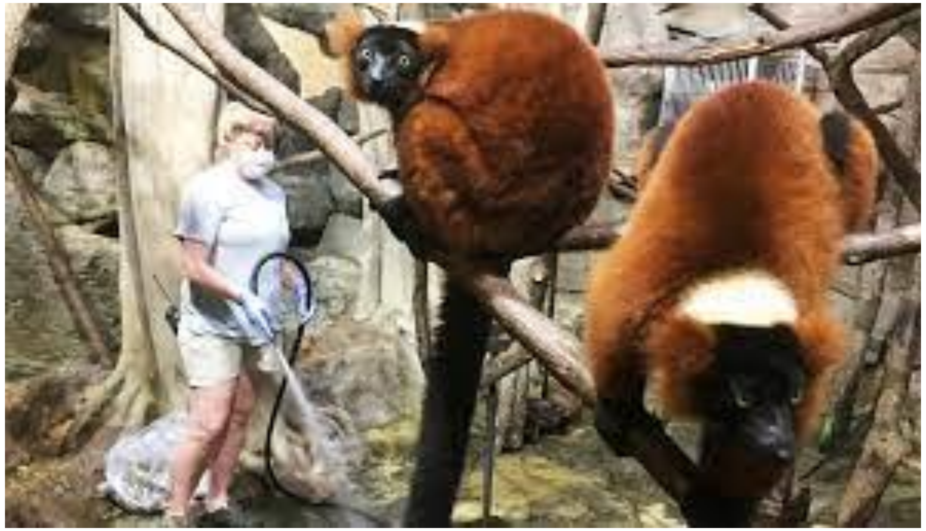

- Fire exits, toilets, mobile phones
- Please don’t share/tweet
- Permission to record (note taking)
- COVID considerations
- Please speak into microphone
- No jargon. Please don’t be afraid to ask for clarification
- Teams. Camera on, microphone on mute please. Raise hand.

CHIEF SCIENTIST OFFICE

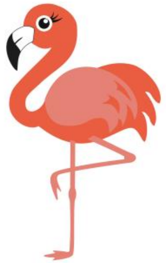

Why are we meeting?

- Zero day admissions rising
- Is there a……..
- Win (children)
- Win (parents/carers)
- Win (primary care)
- Win (secondary care)
- Win (NHS Scotland/Government)

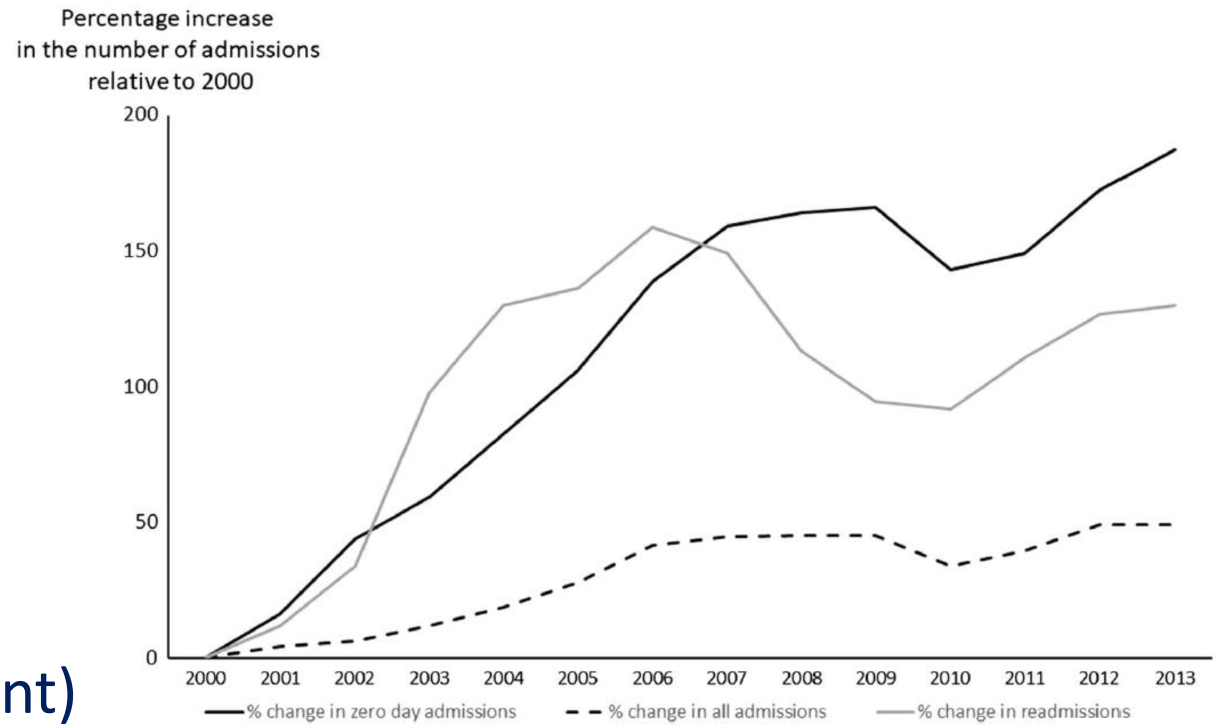

Eur J Pediatr 2018 ;177:381-388

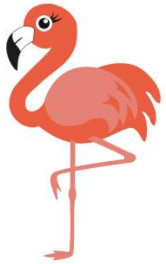

CHIEF
SCIENTIST
OFFICE

Why are we meeting?

This is stakeholder engagement

Two main aims

1. Share results with you
   - What surprises you
2. What are the priorities?
   - What would you do first?

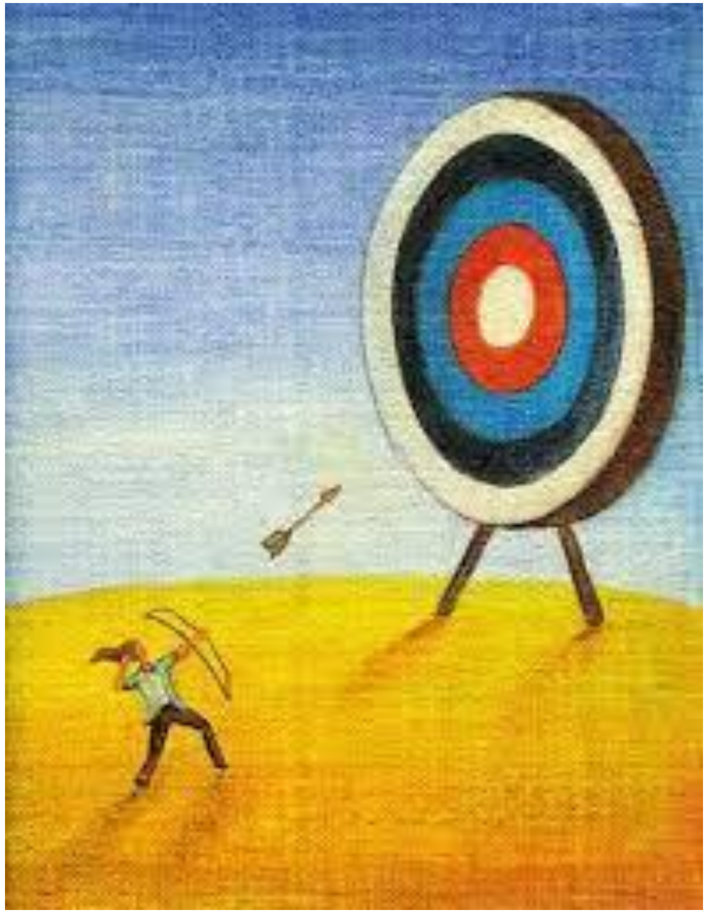

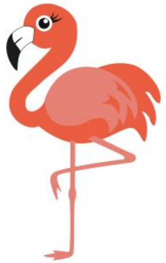

CHIEF SCIENTIST OFFICE

What is the plan for today?

10-10.15 introduction

10.15-11.00* What we found

11-12ish Group discussions, zoo

12.30-1.15 Lunch

1.15-2.15* Opportunities for change

2.15-2.45 Group discussions

2.45-3.00 Wrap up

*

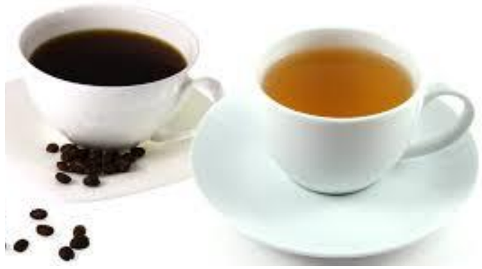

CHIEF SCIENTIST OFFICE

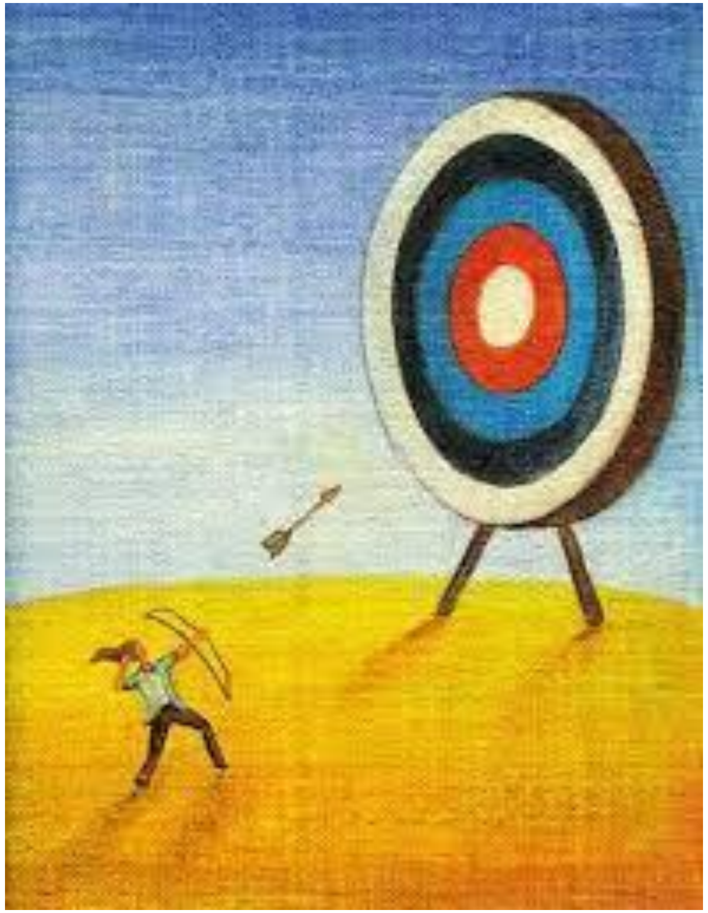

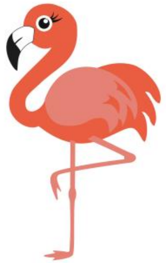

Introductions

- Who is here today?

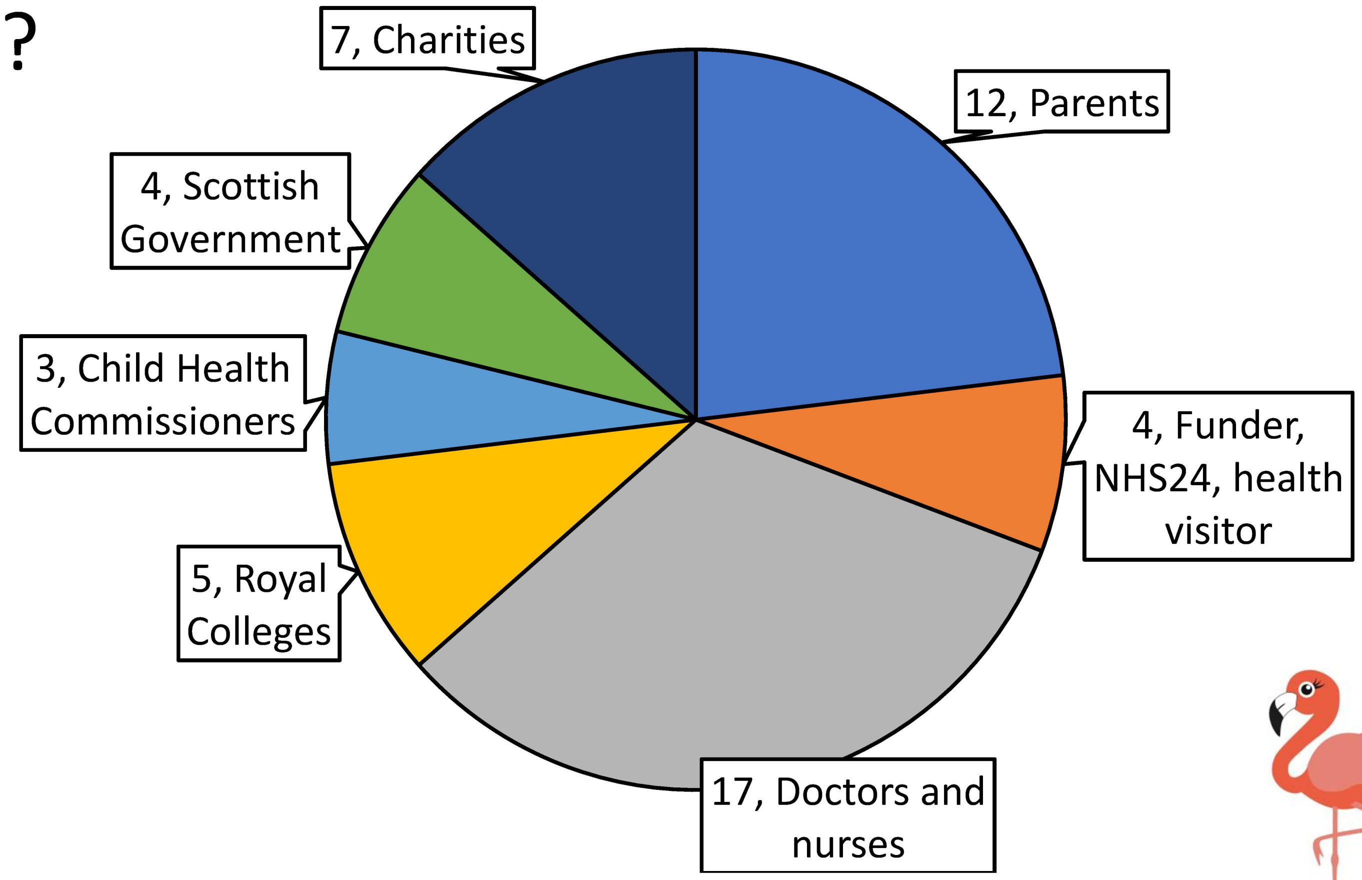

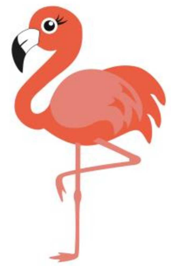

CHIEF SCIENTIST OFFICE

Introductions

- ~35+5 in person
- ~19+2 on Teams
- Some just for am/pm
- ?no reps from
  - Orkney
  - Western Isles
  - Tayside
  - D&G
  - Forth Valley
  - Lanarkshire

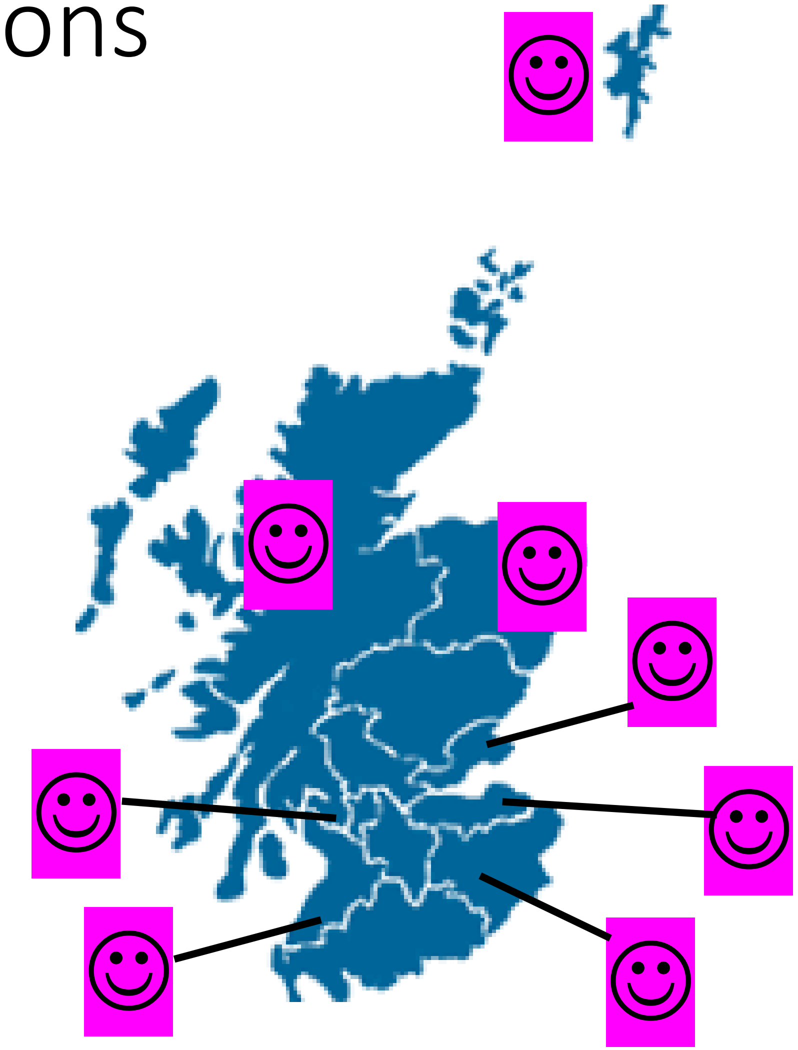

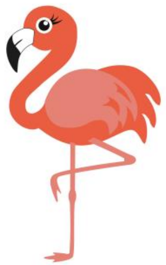

CHIEF SCIENTIST OFFICE

Introductions

Please spend a few minutes

At the Zoo:

- Name
- Which group you are representing
- Why are you here?

Teams:

Please put this in the chat

Note to self:

What do you want to achieve by 3pm?

CHIEF SCIENTIST OFFICE

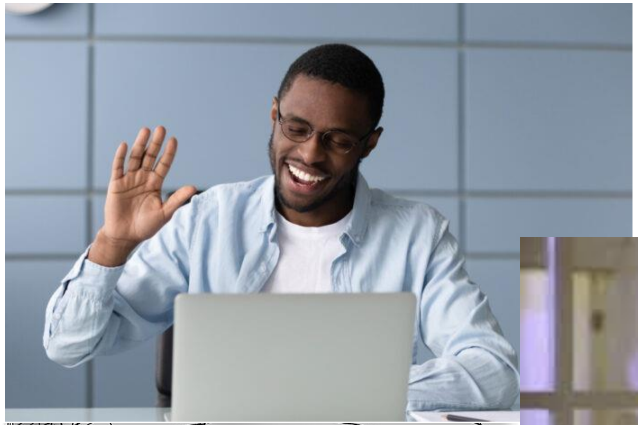

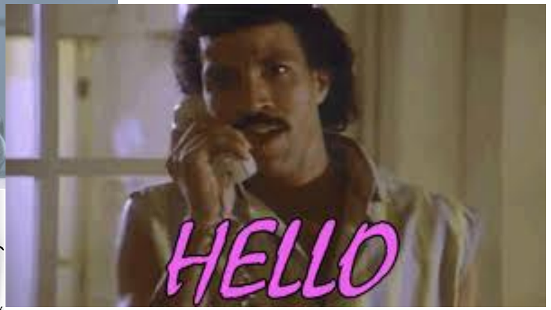

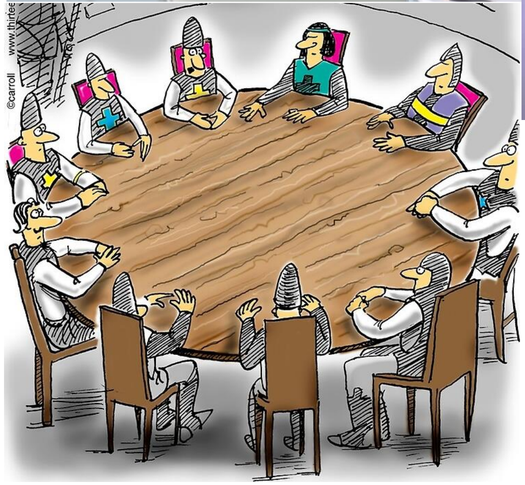

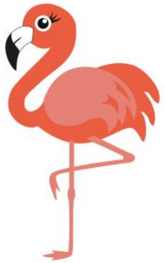

FLAMINGO

Flow of hospital admissions in children and young people – Methods and Results

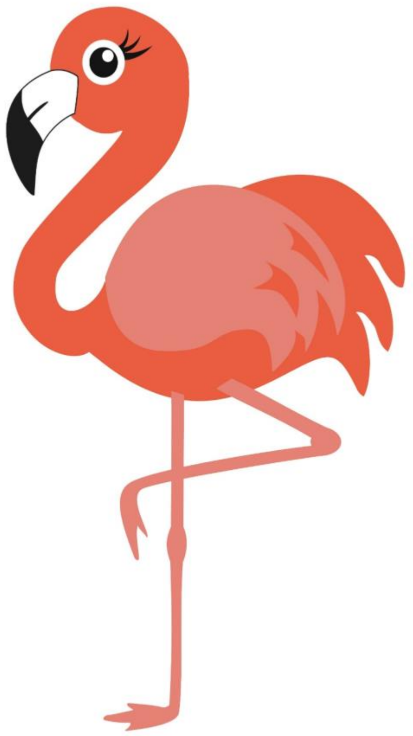

CHIEF SCIENTIST OFFICE

Overview

- Definitions
- Why we did this
- How we did this
- What we found

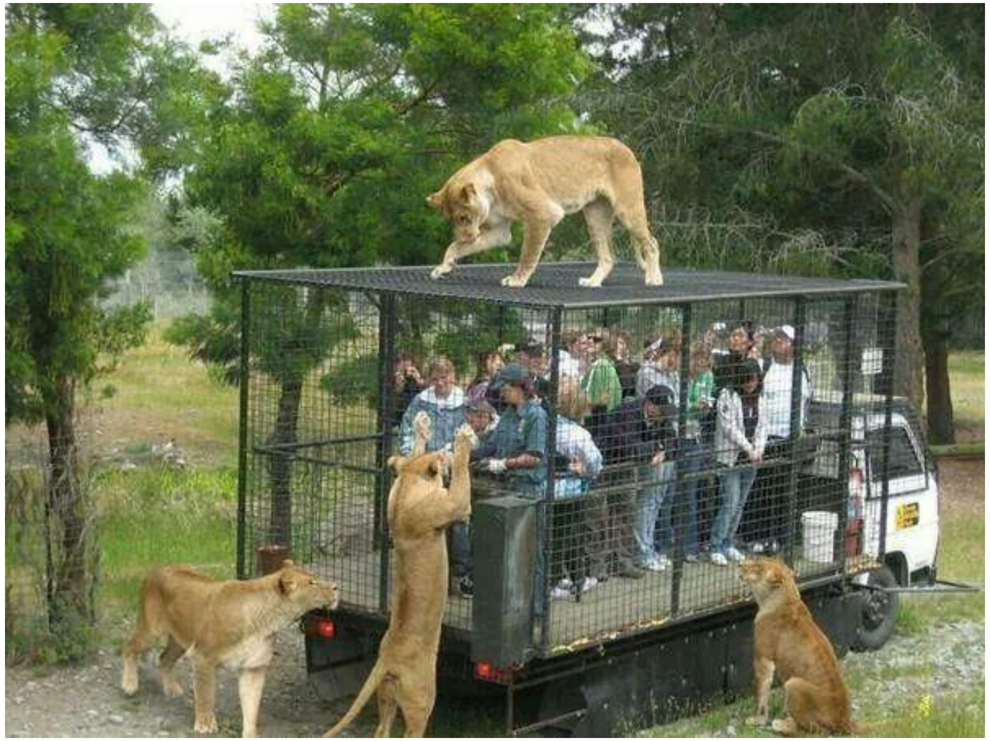

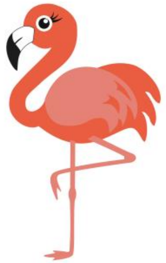

Definitions

- Emergency department
- General practice
- NHS 24
- Out of Hours
- Health board
- Admission
- Zero day admission

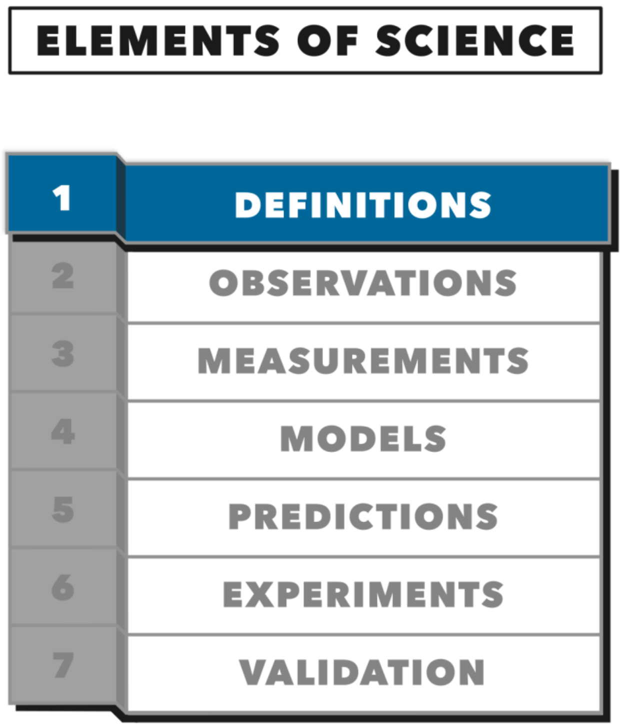

CHIEF SCIENTIST OFFICE

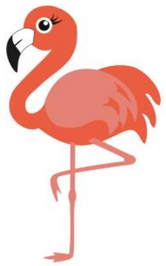

Why

Risers and fallers?

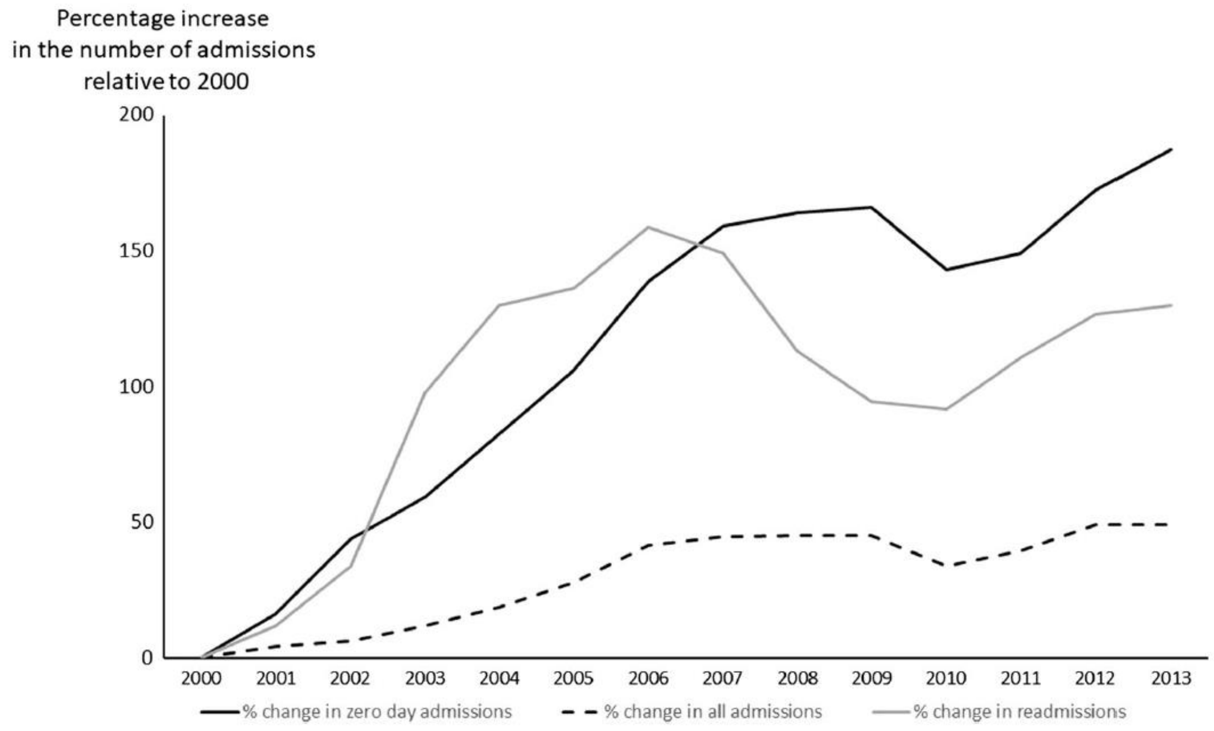

Eur J Pediatr 2018 ;177:381-388

CHIEF SCIENTIST OFFICE

Why

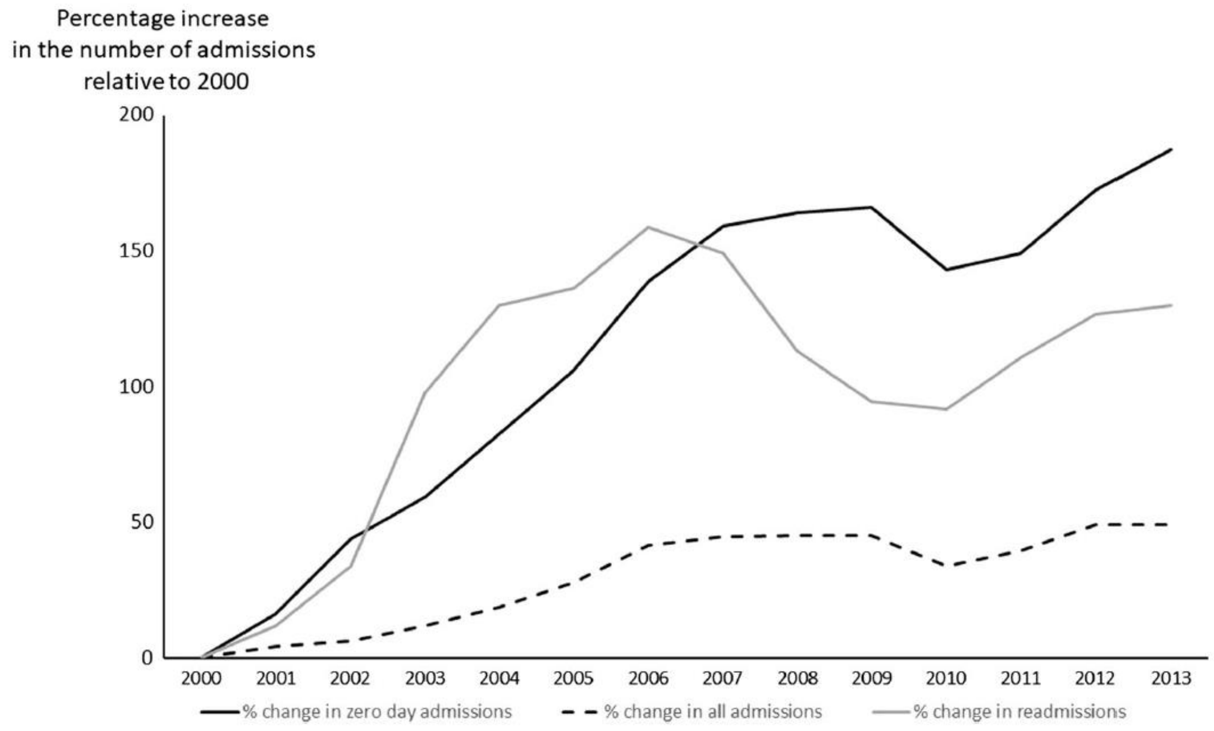

Eur J Pediatr 2018 ;177:381-388

Risers and fallers

↑ 100% "viral infection"

↑45% tonsillitis

↑25% bronchiolitis

↑10% chest infection

↓15% croup

↓15% gastroenteritis

↓25% convulsions

↓25% asthma

CHIEF SCIENTIST OFFICE

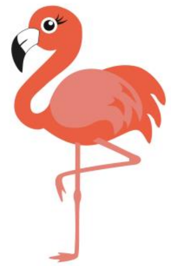

Why

? True change

√ Asthma, convulsions

? Change in parent help-seeking

√ Poverty

√ Expectations

? Change in primary care

? Change in hospital

? All of the above!

Risers and fallers:

↑ 100% “viral infection”

↑45% tonsillitis

↑25% bro[illegible]chiol[illegible]tis

↑[illegible]0% c[illegible]est in[illegible]ection

↓[illegible]5% c[illegible]oup

↓15% gastroenteritis

↓25% convulsions

↓25% asthma

CHIEF SCIENTIST OFFICE

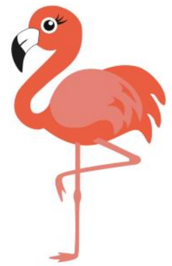

Why

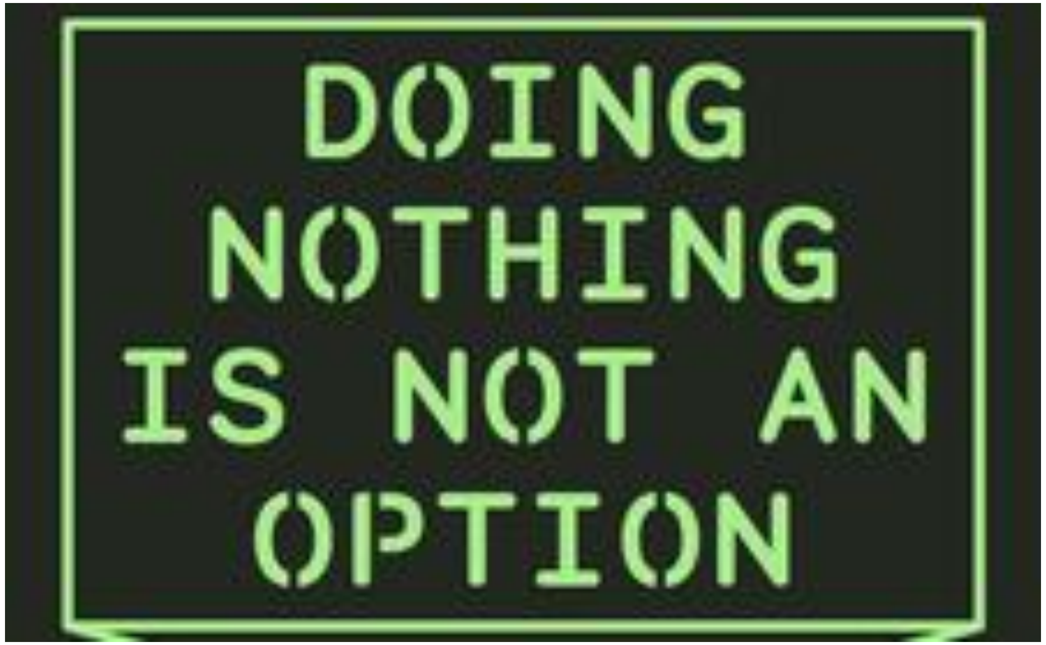

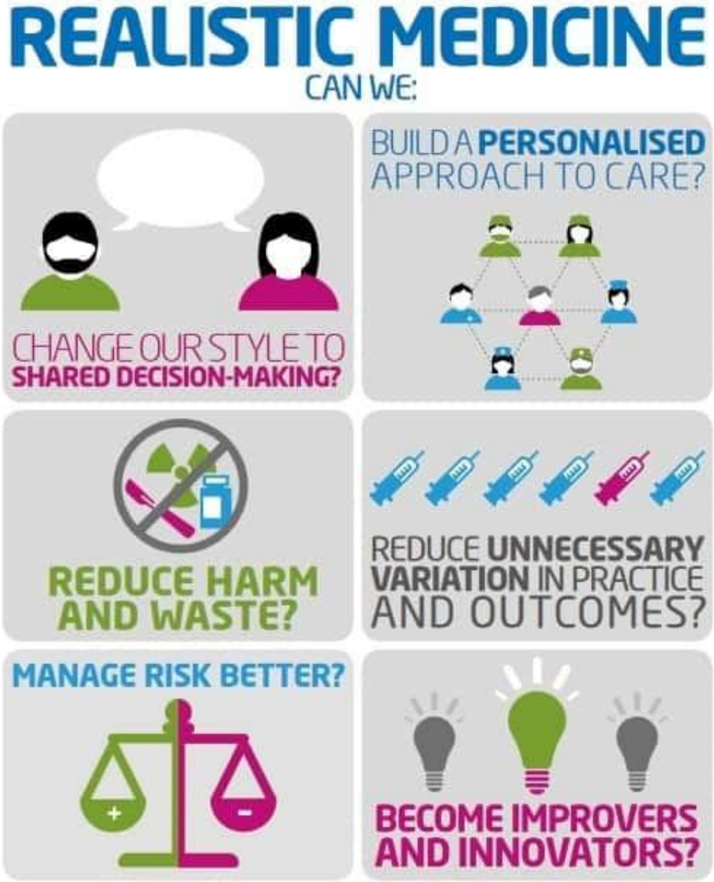

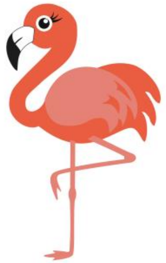

CHIEF SCIENTIST OFFICE

Question

1. How can we best involve parents/carers and children in research to improve emergency paediatric admission pathways?

CHIEF SCIENTIST OFFICE

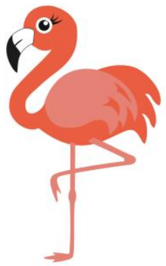

BMJ Publishing Group Limited (BMJ) disclaims all liability and responsibility arising from any reliance placed on this supplemental material which has been supplied by the author(s)

Question and “answer”

1. How can we best involve parents/carers and children in research to improve emergency paediatric admission pathways?

1. Work with parents (mother-baby clubs, hospitals). Pre-COVID.

CHIEF SCIENTIST OFFICE

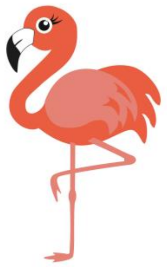

BMJ Publishing Group Limited (BMJ) disclaims all liability and responsibility arising from any reliance placed on this supplemental material which has been supplied by the author(s)

Question and "answer"

1. How can we best involve parents/carers and children in research to improve emergency paediatric admission pathways?
2. Within the 'flow' of zero day admissions, are there identifiable 'streams' of zero day admissions which could be slowed?

1. Work with parents (mother-baby clubs, hospitals). Pre-COVID.

CHIEF SCIENTIST OFFICE

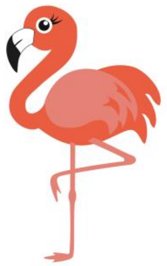

Question and "answer"

1. How can we best involve parents/carers and children in research to improve emergency paediatric admission pathways?
2. Within the 'flow' of zero day admissions, are there identifiable 'streams' of zero day admissions which could be slowed?

1. Work with parents (mother-baby clubs, hospitals). Pre-COVID.
2. Collect data (numbers and words).

CHIEF
SCIENTIST
OFFICE

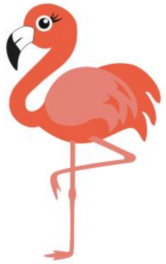

Question and "answer"

1. How can we best involve parents/carers and children in research to improve emergency paediatric admission pathways?
2. Within the 'flow' of zero day admissions, are there identifiable 'streams' of zero day admissions which could be slowed?
3. Which of these 'streams' is the most important to slow down first?

1. Work with parents (mother-baby clubs, hospitals). Pre-COVID.
2. Collect data (numbers and words).

CHIEF SCIENTIST OFFICE

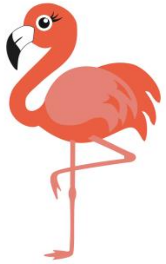

Question and “answer”

1. How can we best involve parents/carers and children in research to improve emergency paediatric admission pathways?
2. Within the ‘flow’ of zero day admissions, are there identifiable ‘streams’ of zero day admissions which could be slowed?
3. Which of these ‘streams’ is the most important to slow down first?

1. Work with parents (mother-baby clubs, hospitals). Pre-COVID.
2. Collect data (numbers and words).
3. Meeting at the zoo.

CHIEF SCIENTIST OFFICE

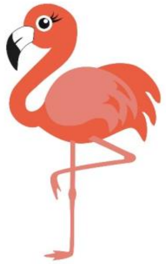

BMJ Publishing Group Limited (BMJ) disclaims all liability and responsibility arising from any reliance placed on this supplemental material which has been supplied by the author(s)

Numbers and words

Numbers = what is happening (quant)

Words = why it is happening (qual)

Mixed methods, sum>parts

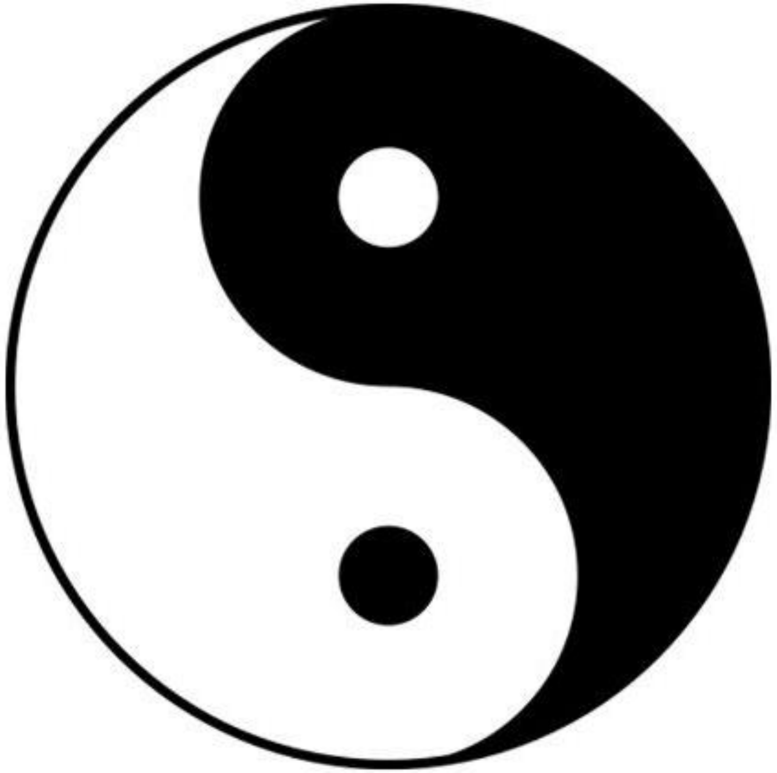

CHIEF SCIENTIST OFFICE

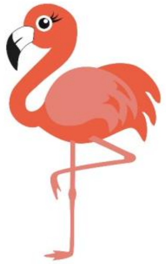

BMJ Publishing Group Limited (BMJ) disclaims all liability and responsibility arising from any reliance placed on this supplemental material which has been supplied by the author(s)

Qualitative Methods

Development of research questions

Visiting a number of parent and toddler groups

Health boards case studies

Five health boards chosen for diversity of geography and population size

Recruitment

Social media, posters, sharing by stakeholder groups, emails to contacts

Telephone interviews

Coding and analysis

Transcription. Coded and analysed by the qualitative team

CHIEF SCIENTIST OFFICE

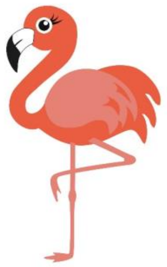

BMJ Publishing Group Limited (BMJ) disclaims all liability and responsibility arising from any reliance placed on this supplemental material which has been supplied by the author(s)

Quantitative Methods

- NHS Scotland collects data
- Collect data from 3 routes into hospital
- 2015-2017
- Link them to map the journey
- Identify "streams" within the "flow"
- Permissions

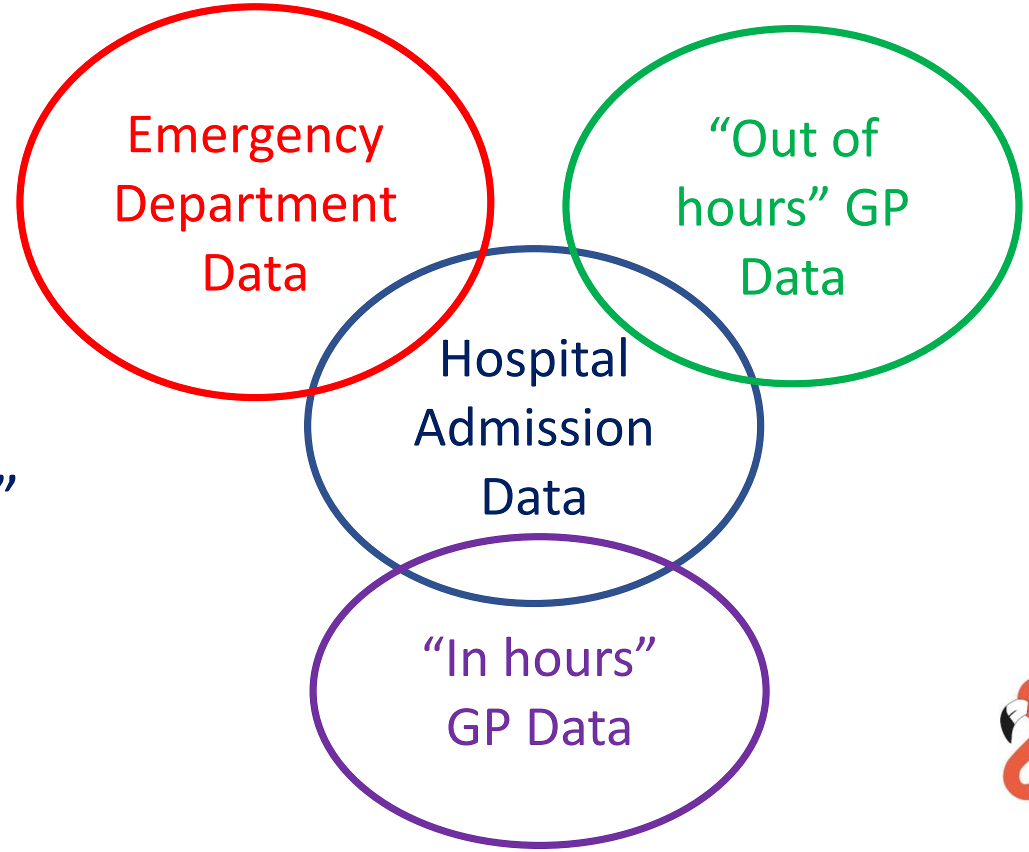

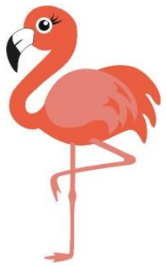

CHIEF SCIENTIST OFFICE

Quantitative Results

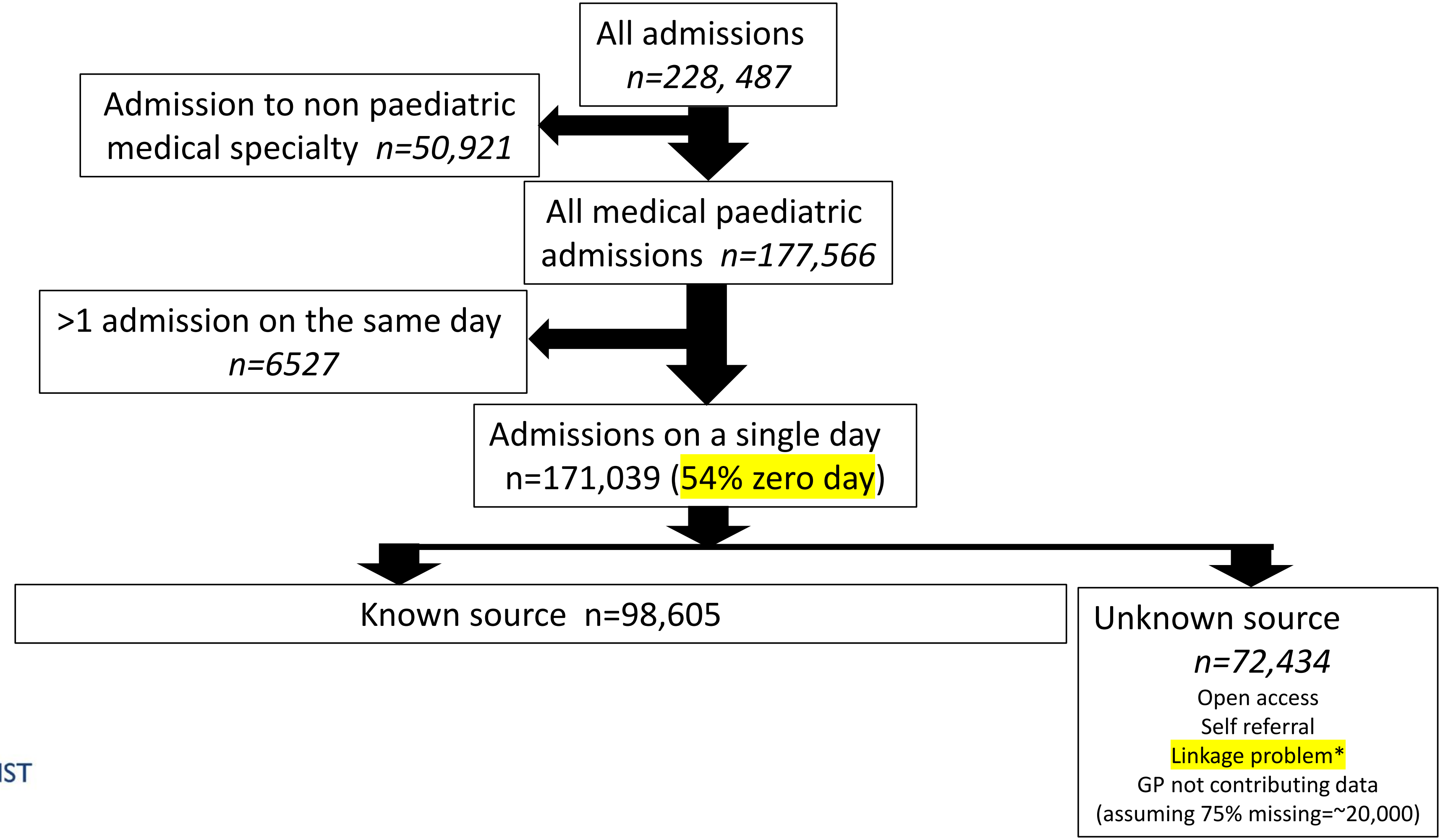

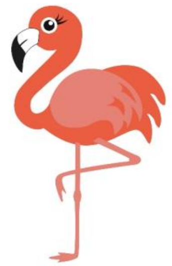

BMJ Publishing Group Limited (BMJ) disclaims all liability and responsibility arising from any reliance placed on this supplemental material which has been supplied by the author(s)

Quantitative Results

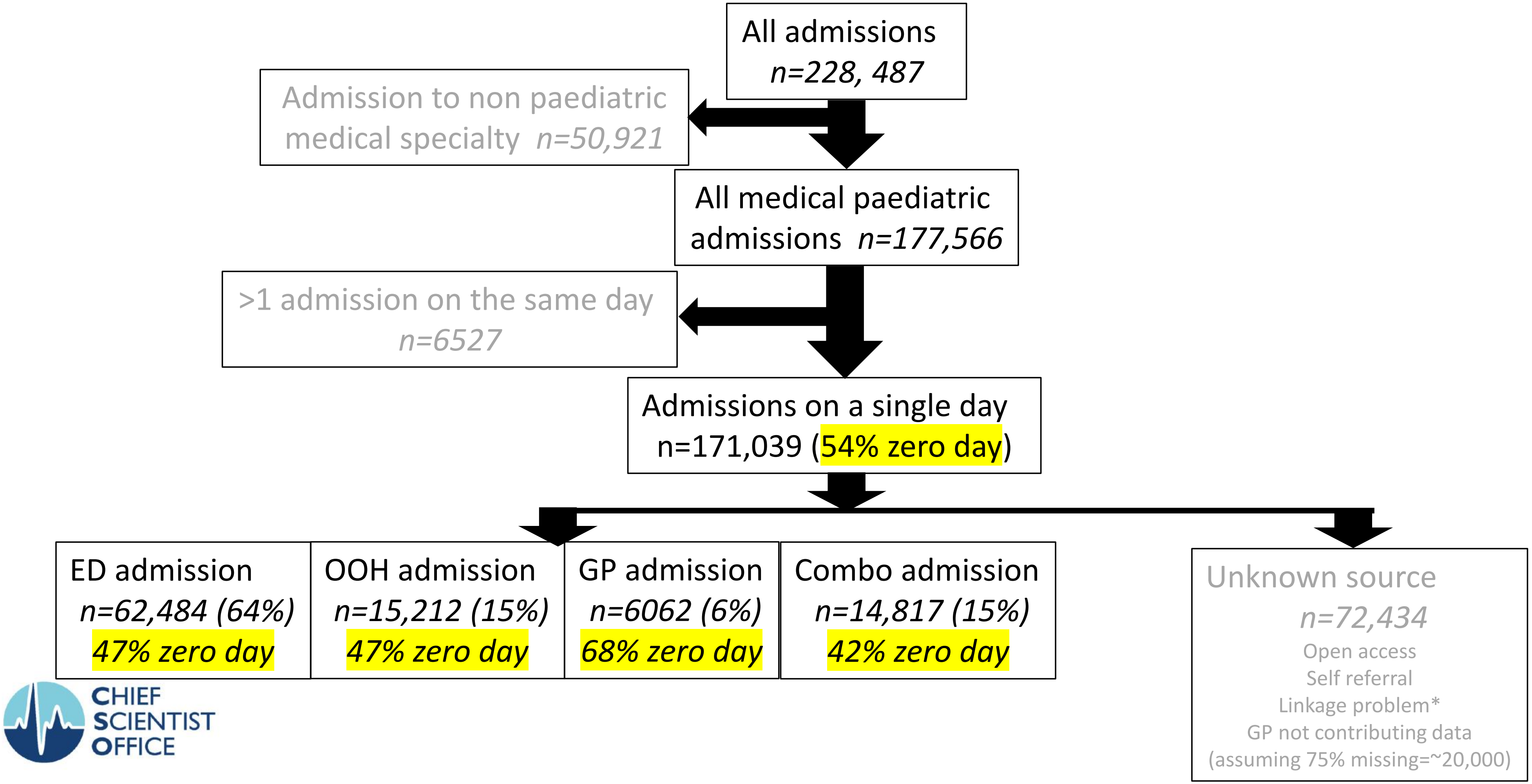

CHIEF SCIENTIST OFFICE

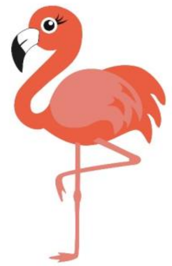

BMJ Publishing Group Limited (BMJ) disclaims all liability and responsibility arising from any reliance placed on this supplemental material which has been supplied by the author(s)

Quantitative Results

- What about regional variation?
- Conditions don’t recognise boundaries
- Can this reflect differences in journeys through NHS?

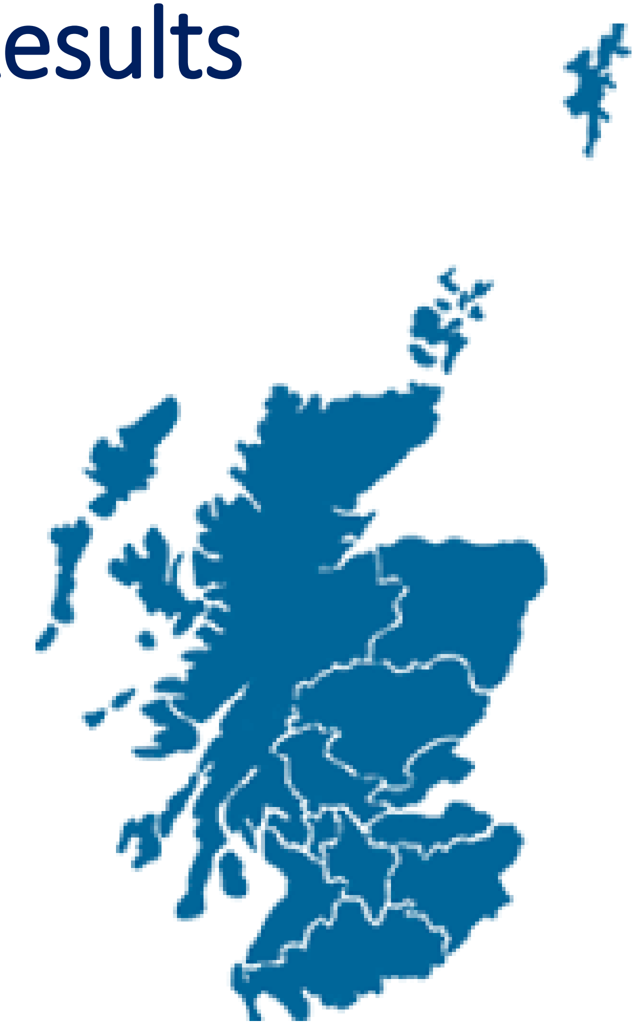

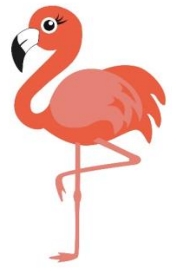

CHIEF SCIENTIST OFFICE

Quantitative Results

- What about regional variation?
- Conditions don't recognise boundaries
- Can this reflect differences in journeys through NHS?

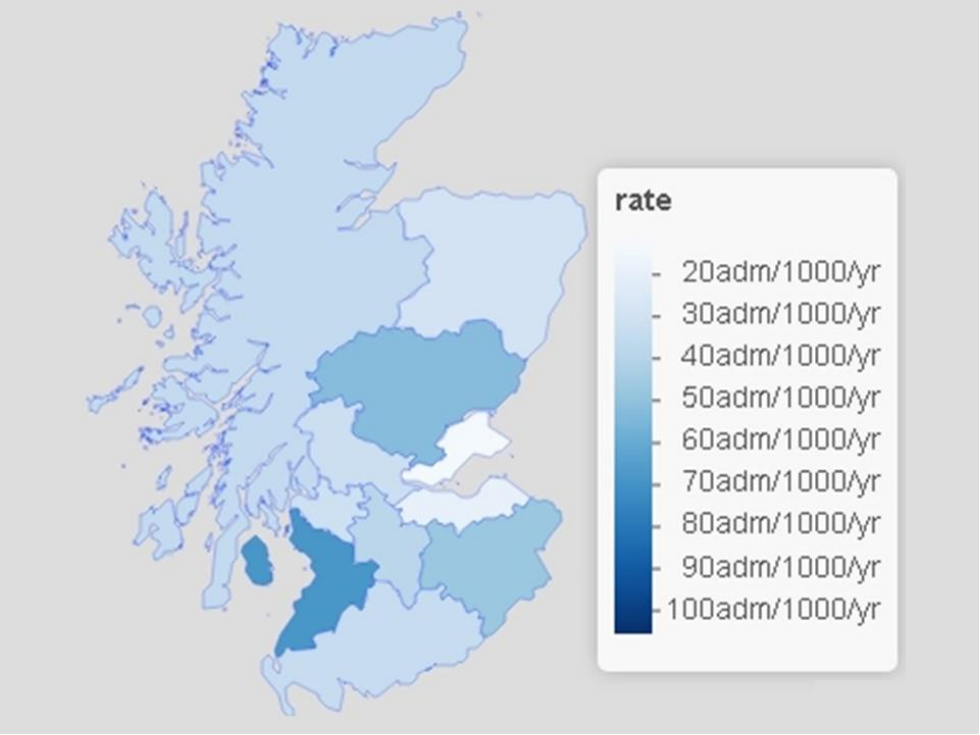

CHIEF SCIENTIST OFFICE

Quantitative Results

Lower than average form this source. Higher than average from this source

Zero day admissions	GP 8.9%	OOH 15.2%	ED 62.5%	Combo 13.4%
Asthma (n=2148)	5.7%	14.3%	68.7%	11.3%
Bronchiolitis (n=6295)	10.1%	18.1%	56.3%	15.4%
Croup (n=2799)	5.2%	14.1%	64.3%	16.4%
Afebrile convulsion (n=1344)	2.3%	2.1%	87.3%	8.3%
Febrile convulsion (n=976)	1.6%	2.2%	84.3%	11.9%
Chest infection (n=2552)	10.4%	20.3%	56.5%	12.8%
Tonsillitis (n=3706)	9.2%	21.0%	54.5%	15.4%
URTI (n=8165)	7.2%	18.5%	58.3%	16.0%
Viral infection (n=10,236)	7.8%	18.1%	58.7%	15.4%

CHIEF SCIENTIST OFFICE

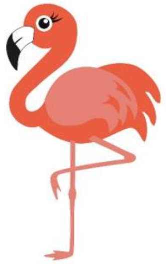

Quantitative Results

Lower than average form this source. Higher than average from this source

Zero day admissions	GP [illegible]	[illegible]	ED [illegible]	Combo 13.4%
Asthma (n=2148)	[illegible]	[illegible]	[illegible]	11.3%
Bronchiolitis (n=6295)	[illegible]	[illegible]	[illegible]	15.4%
Croup (n=2799)	[illegible]	[illegible]	[illegible]	16.4%
Afebrile convulsion (n=1344)	2.3%	2.1%	[illegible]	8.3%
Febrile convulsion (n=976)	1.6%	2.2%	84.[illegible]	11.9%
Chest infection (n=2552)	10.4%	20.3%	56.5%	[illegible]
Tonsillitis (n=3706)	9.2%	21.0%	54.5%	15.4%
URTI (n=8165)	7.2%	18.5%	58.3%	16.0%
Viral infection (n=10,236)	7.8%	18.1%	58.7%	15.4%

All very interesting but what is the story behind the numbers?

CHIEF SCIENTIST OFFICE

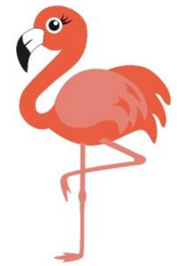

BMJ Publishing Group Limited (BMJ) disclaims all liability and responsibility arising from any reliance placed on this supplemental material which has been supplied by the author(s)

Quantitative Results

- Match cases and controls
- What “discretionary factors” differ
- Time of presentation (0-6am highest)
- Causation not proven!

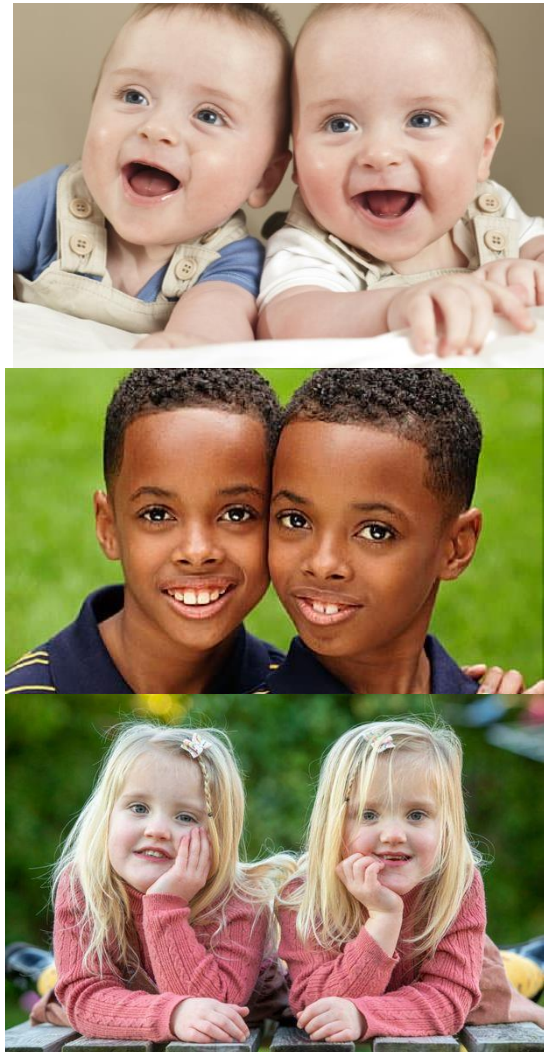

CHIEF SCIENTIST OFFICE

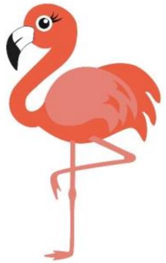

Quantitative Results

Summary

- There are streams within the flow
- Zero day admissions vary by
  - Referral source
  - Geography
  - Time of presentation
- Deprivation gradient as for all admissions
  - 30% vs 13%
- Time for some interviews!

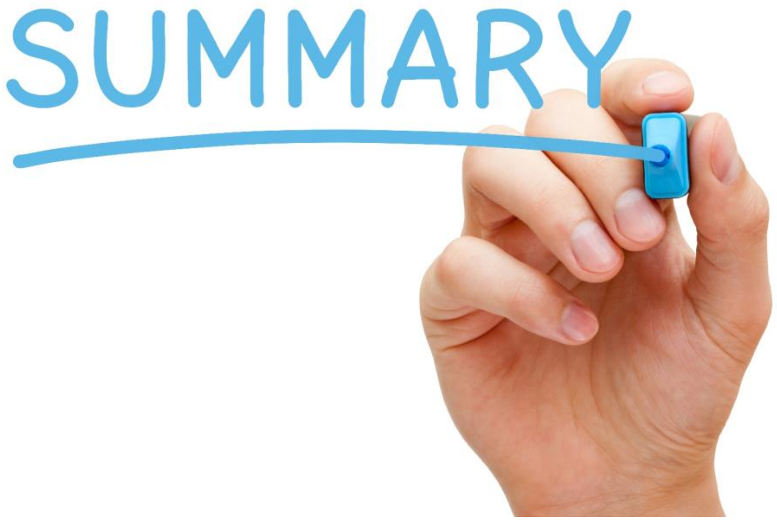

CHIEF SCIENTIST OFFICE

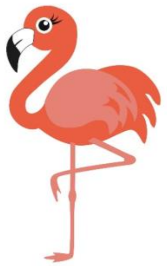

Research interview participants

Type of participant	GP	Nurse (community)	Doctor (hospital)	Consultant (hospital)	Nurse (hospital)	Families	Total
**No.**	10	5	7	10	16	21	**69**

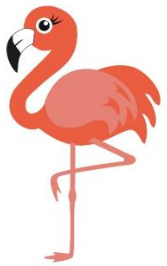

BMJ Publishing Group Limited (BMJ) disclaims all liability and responsibility arising from any reliance placed on this supplemental material which has been supplied by the author(s)

Recording admissions

Consultant

It gets terribly confusing - how do you actually count referrals, admissions, turnarounds whatever? The way our unit is set up, the assessment unit has got X beds in it and it is physically, geographically next door to the ward. And I think if, for whatever reason, the child is transferred through to the ward, then that becomes an admission. Otherwise it is not counted as an admission. The child will be sent through to the ward because there's no space in the assessment unit to let them sit for three or four hours but then they're sent home from the ward after another couple of hours. That still falls into your criteria of a one day admission or less than one day admission, but it wouldn't be counted as an admission on our numbers.

How are admissions recorded in different hospitals and how does this then impact on the quantitative data for zero day admissions?

CHIEF SCIENTIST OFFICE

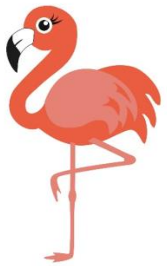

BMJ Publishing Group Limited (BMJ) disclaims all liability and responsibility arising from any reliance placed on this supplemental material which has been supplied by the author(s)

Children going to different areas/ health boards

Doctor

So even the ones that present at hospitals A and B - which is about a two, two-and-a-half hour drive away, ‘cause they're an adult hospital and got no paediatric services, so they see and treat children under the advice from us - … we can VC into them and help them, but if that child needs to be admitted then there's no services elsewhere for them, they have to come down here.

Parent

The medical team in hospital C debated sending Ella (fictitious name) to hospital D and I was surprised that the decision to send her to hospital D wasn’t made sooner. When the Senior Nurse came on shift, things started to move quicker and thankfully the decision was made to send us to hospital D. My husband and daughter left the hospital at 6pm to be taken to the air ambulance to fly to hospital D.

Potential impact on zero day numbers from children having to travel to specialist hospitals outside their own health board?

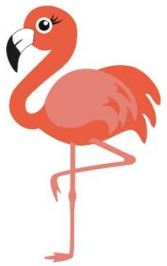

CHIEF SCIENTIST OFFICE

Resources: people and physical

Doctor

Overnight when a child is admitted we don't have nursing staff for our observations unit so therefore the child goes straight to inpatients and is admitted as an inpatient overnight, 'cause generally the majority of them that come in overnight stay for the morning anyway.

Doctor

If I wasn't busy on an evening, a nightshift, I would wander down to the out-of-hours because it was on the same site and just said, 'Well look, I'll just come down and see the child there.' And all they wanted was a bit of reassurance that what they were doing was correct and the child was okay.

How do resources impact on whether a child can be managed in that area, or on where a child is sent and therefore how an admission is recorded?

CHIEF SCIENTIST OFFICE

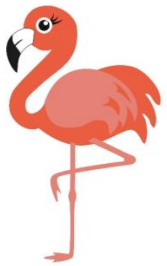

BMJ Publishing Group Limited (BMJ) disclaims all liability and responsibility arising from any reliance placed on this supplemental material which has been supplied by the author(s)

Policies and targets

Consultant

So all of the patients that come through our department are subject to the Scottish Government's unscheduled care four-hour target. So obviously that target does drive our practice and behaviour to a certain extent. We had set up an adjacent area and badged it as a clinical decision unit to essentially allow our department to continue to function with the volume of patients that we expected to allow patients to flow in and flow out of the back, while still obviously being able to meet that target.

GP

They have a rule in the ward in NHS X that they won't keep children longer than 24 hours, that’s their rule. If the child needs to stay in hospital beyond 24 hours the child needs to go to Hospital E.

How do NHS, health board, or hospital targets impact on decisions made about a child’s care?

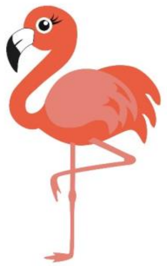

CHIEF SCIENTIST OFFICE

Physical access – primary care

Parent

Where we stay we're kind of out the way a wee bit, and they were saying it's taking ages to get an ambulance … I would've probably taken him in the car and just drove … but yeah public transport would be a no go.

GP

We are quite rural but I think it has an effect of families in two ways; one way the patient is reluctant to do that travel, they'd prefer to stay at home but also it actually means that sometimes we're taking on more risk because if things do go badly it takes longer to get someone into hospital as well.

How does physical access impact on decisions in primary care about whether to refer a child to hospital or treat them in the community?

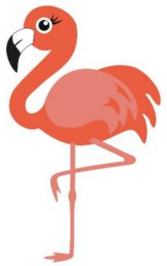

CHIEF SCIENTIST OFFICE

BMJ Publishing Group Limited (BMJ) disclaims all liability and responsibility arising from any reliance placed on this supplemental material which has been supplied by the author(s)

Physical access – secondary care

Consultant

I guess one thing with our geography, so the juniors, whenever they come and ask me advice about kids I'm always, like, 'where do they stay?' and they're, like, 'why does that matter?' but once you get to know our geography - so, for example, it can be over an hour to get back to the hospital, so I would be more likely to bring those kids in, particularly out-of-hours, if they’ve got croup or bronchiolitis or something, sometimes they'd get watched overnight, whereas if they were very close to the hospital you would send them home.

How does physical access impact on decisions made by hospital staff about whether a child is kept in or discharged home?

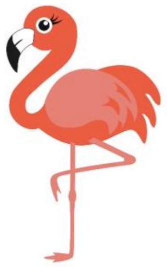

CHIEF SCIENTIST OFFICE

BMJ Publishing Group Limited (BMJ) disclaims all liability and responsibility arising from any reliance placed on this supplemental material which has been supplied by the author(s)

Social issues, including deprivation, and language issues

Consultant

Actually I will definitely be influenced by if they are going home to a dirty home. If they don't have a car then I'm not going to expect them to somehow come in on three buses to come and get an IV antibiotic 'cause it saves me a bed. It does influence your decision making.

Consultant

So there is a lot of deprivation but then you have pockets of really well off communities. So on the one hand you'd have the stereotype of the more deprived family maybe presenting more often, but then you've also got the more entitled wealthier people maybe feeling more entitled and therefore would they present more often.

How do social issues and deprivation/language issues impact on whether or not a child is admitted or discharged home?

CHIEF SCIENTIST OFFICE

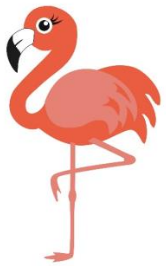

BMJ Publishing Group Limited (BMJ) disclaims all liability and responsibility arising from any reliance placed on this supplemental material which has been supplied by the author(s)

Expectations and experiences of services

Nurse

So I personally believe we're the victim of our own success and with what's happening out in the GP community and things like that, I think we can't turn back time. People do not feel safe and secure being managed out in the community and I think they feel it's safer that they're being assessed in the hospital and that’s a feeling they get from the press and from GPs and things like that. I think we need to accept that this is our workload and we should get better at managing it.

Parent

If he was to get that breathless again I would probably phone an ambulance ‘cause I probably didn’t realise at the time how serious it was…. But we are only along the road so it wasn’t as if we’re miles away but I probably wouldn’t phone 111 the next time, I would just take him in or phone an ambulance.

How and where do families seek healthcare advice and what do they expect from that?

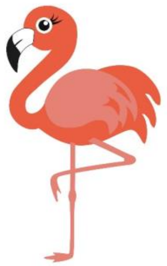

CHIEF SCIENTIST OFFICE

Funded by:

BMJ Publishing Group Limited (BMJ) disclaims all liability and responsibility arising from any reliance placed on this supplemental material which has been supplied by the author(s)

A few questions to consider….

1. **What is the most important thing you have heard this morning?**

2. **What surprised you most?**

3. **What did not surprise you?**

See you back here at 1.15 ☺

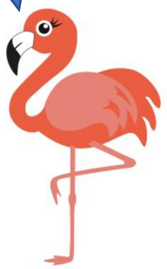

CHIEF SCIENTIST OFFICE
