# Supplementary material for: Identifying and prioritising future interventions with stakeholders to improve paediatric urgent care pathways in Scotland, UK: a mixed-methods study
Source: BMJ Open. 2023 Oct 12;13(10):e074141. doi: 10.1136/bmjopen-2023-074141 (PMC10582902; doi:10.1136/bmjopen-2023-074141)
Supplement: Supplementary data [file bmjopen-2023-074141supp009.pdf]

BMJ Publishing Group Limited (BMJ) disclaims all liability and responsibility arising from any reliance placed on this supplemental material which has been supplied by the author(s)


# Welcome back

Time for a recap
and to welcome
new colleagues

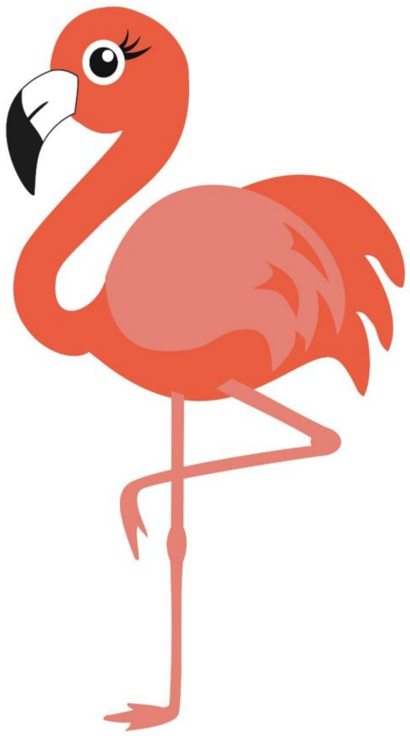

CHIEF
SCIENTIST
OFFICE

# Why are we meeting?

This is stakeholder engagement

Two main aims

1. Share results with you
   - What surprises you
2. What are the priorities?
   - What would <u>you</u> do first?

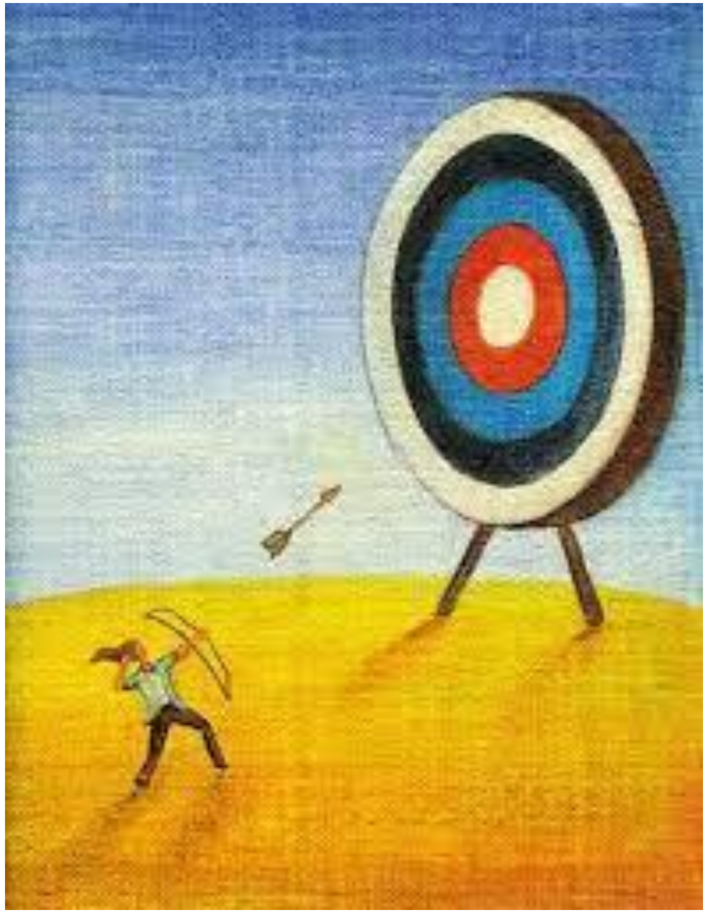

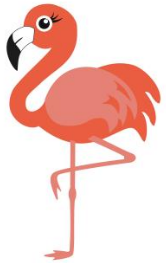

CHIEF SCIENTIST OFFICE

# Introductions

- Who is here today?

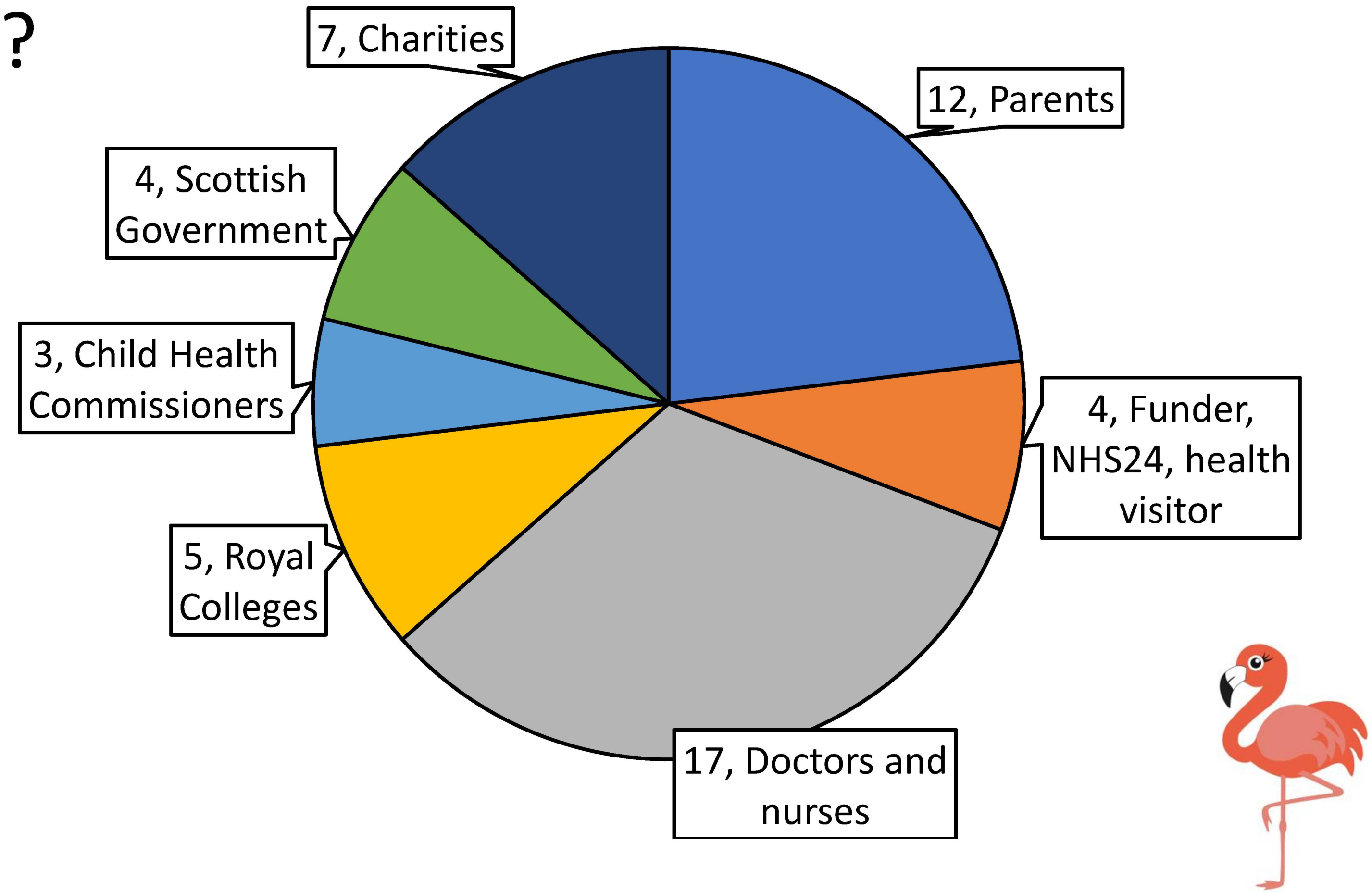

# FLAMINGO
# Flow of hospital admissions in children and young people

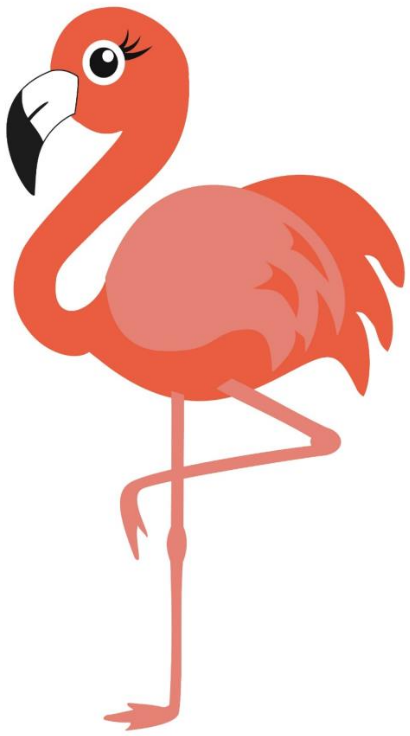

## Potential for Innovation and Change

CHIEF SCIENTIST OFFICE

# Practices & approaches tried & deemed successful by interviewees

- Access to specialist paediatric staff to inform decision-making, assessment & management of care for staff with less paediatric experience
- Change layout of Emergency Departments (EDs) to separate infectious & non-infectious patients
- Areas in ED where children can be observed for short periods
- 24-hour open access directly back to paediatric ward for patients following hospital discharge
- Dedicated specialist nursing teams for management of long-term conditions

CHIEF SCIENTIST OFFICE

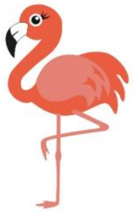

BMJ Publishing Group Limited (BMJ) disclaims all liability and responsibility arising from any reliance placed on this supplemental material which has been supplied by the author(s)


# Two levels of change to consider

**1. Quality Improvement initiatives within existing systems**

- Quicker to implement
- Existing evidence for effectiveness
- 'Easy wins' around improving education & communication

**2. System or Pathway changes**

- Uncertain evidence for its effectiveness
- Required further research & development
- Take longer to implement
- Require significant resources

CHIEF SCIENTIST OFFICE

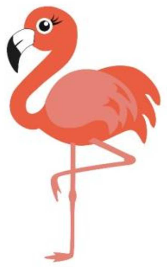

BMJ Publishing Group Limited (BMJ) disclaims all liability and responsibility arising from any reliance placed on this supplemental material which has been supplied by the author(s)


# 1. Suggestions for Quality Improvement

- More innovative educational resources – pictures, videos, practical education for common scenarios, websites/social media, including for non-English speakers
- Information sharing activities & social events alongside services where all attend e.g. immunisations
- Improve NHS secondary/primary care/education/social team communication to prevent children falling through the gap
- Access to clinical support for GPs from expert colleagues
- Rotation of GP and hospital trainees/junior staff
- Separate places for infectious/non-infectious admissions
- Fast-track appointment within one week as an alternative to admission
- Evaluate use of video conferencing for patient assessment and triage

CHIEF SCIENTIST OFFICE

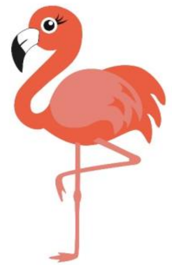

BMJ Publishing Group Limited (BMJ) disclaims all liability and responsibility arising from any reliance placed on this supplemental material which has been supplied by the author(s)


# 2. System or Pathway Changes

- Gap in <u>acute</u> paediatric nursing and medical skills in the community

- Assessment and observation in community settings
- Holistic children's hubs
- Hospital at Home model

- Care pathways for specific conditions

CHIEF SCIENTIST OFFICE

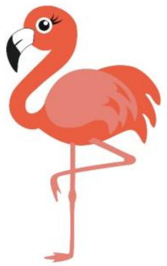

BMJ Publishing Group Limited (BMJ) disclaims all liability and responsibility arising from any reliance placed on this supplemental material which has been supplied by the author(s)


# Gap in acute paediatric skills in the community

## Hospital Nurse

It might be worth thinking about putting advanced paediatric nurse practitioners in GP surgeries. Again you've got well-experienced paediatric nurses that could go out into the community, see these patients, maybe be able to keep them at home by reassessing during the day, knowing what they're reassessing and also be able to do some teaching with the GPs. I think the way forward is maybe to try and put more paediatric experienced staff out in the community that can see acute unwell children.

**What are the opportunities and challenges?**

**Would this be fair and sustainable for all patients?**

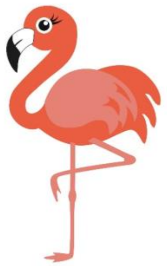

CHIEF SCIENTIST OFFICE

# Assessment and observation in community settings

## Hospital doctor

It'd be lovely to get more paediatric experience in the peripheries, in which case we might be able to reduce the number of patients that come down to us. And that's for the parents' benefit as well and the family's benefit as well.

**How do we get the right skills at the right time in the right place?**

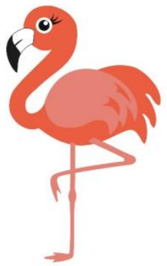

CHIEF SCIENTIST OFFICE

# Holistic children's hubs linked to Children's Hospital

Hospital Doctor

What I find is that children are born and there's nothing else for children and families apart from a health visitor and, actually, it's almost like if we could have hubs and community hubs.

Hospital Doctor

Having a facility where you can sit and watch them, whether or not you actually do a specific intervention, even if you can just watch them and repeat their observations over an hour or two, we have found that that can help. We had a period of time where one of our consultants went out and spent time in the out-of-hours service and persuaded them to set aside an area in the out-of-hours building where children could wait and be observed. […]. You need space and you need the appropriate staff to look after them, you can't just sit them down and abandon them, so you need staff that are focused on acute assessment and acute treatment, you need a geographical location that allows you to do that.

**Infection hubs? Assessment and observation hubs? Integrated holistic Physical, Mental and Social hubs? Range of staff and skills?**

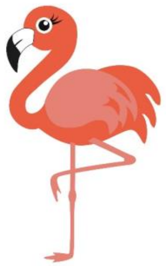

CHIEF SCIENTIST OFFICE

# Hospital at Home model

Hospital doctor

So what we've found increasingly is that if there's no disease reason for the child being admitted it's actually do to with social support. And I think because we don’t have the community infrastructures in place to be able to say, ‘You were utterly distraught at two in the morning, we calmed you down, you went home and this person is going to come out to your house to see how you're doing in the day,' because we don’t have that level of sophistication, then often we use the hospital as a safety net.

**Logistics of home visits - e.g. in rural areas / winter months / pandemics?**

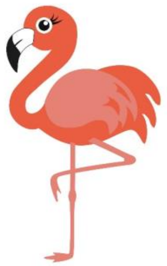

CHIEF SCIENTIST OFFICE

# Specialist care pathways?

Parent

999 was the first call, just purely because it was a Sunday evening. Normally his diabetic nurse is absolutely amazing, so if that happened during the week we would phone her and get advice from her.

Parent

I really, really wasn't happy with the way that NHS111 call was handled. I immediately recognised she was having a seizure. The call handler basically made me feel like I was an overreacting mother. When the nurse phoned back was a bit more reassuring, but again, had gone with it being a viral response and I kept on saying 'I don't think it's a viral response.' I didn't even bother phoning 111 [the second time], I just took her straight up to A&E, but because everything was normal by the time we got there, again I was just sent home and kind of made to feel like I was a bit overreacting. So when she finally was seen and diagnosed by the clinician up at the hospital, who's a consultant, he was brilliant, he turned round and gave the diagnosis of it being epilepsy.

**Suggested special pathways for admission**
**Early postnatal pathway for babies under 2 years? Wheezers? Bronchiolitis? Seizure?**

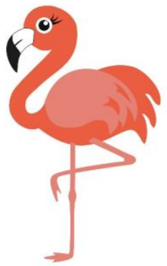

CHIEF SCIENTIST OFFICE

# Is there an opportunity for a seizure pre-hospital care pathway?

## A Case Study

CHIEF SCIENTIST OFFICE

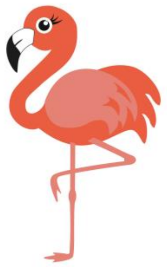

# Characteristics of unscheduled paediatric hospital admissions for febrile and afebrile convulsions

| Characteristic | Febrile Convulsion n (%) | Afebrile Convulsion n (%) |
|---|---|---|
| Unscheduled Admission | 1916 (1.1)* | 2661 (1.6)* |
| Zero Day Admission | 976 (51.0) | 1344 (51.0) |
| Sex: | | |
| Female | 424 (43.4) | 633 (47.1) |
| Male | 552 (56.6) | 711 (52.9) |
| Age at Admission (years): | | |
| < 1 | 96 (9.8) | 131 (9.8) |
| 1-4 | 818 (83.8) | 499 (37.1) |
| 5-9 | 54 (5.5) | 387 (28.8) |
| 10-15 | 5 (0.5) | 323 (24.0) |

* % of total emergency medical paediatric admissions to Scottish hospitals between 2015 and 2017 which was 171 039.

CHIEF SCIENTIST OFFICE

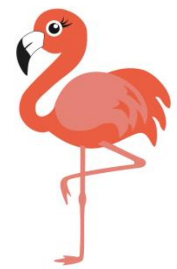

# Interviews with health professionals and parents…

**Health Care Professionals (HCPs)**

- To explore their experiences of and decisions around pre-hospital referrals and subsequent ZDAs of children
- Range of HCPs including ED consultants, nurses, GPs (n=48)

**Parents**

- To explore their experiences of accessing hospital care resulting in a ZDA
- Range of experiences/conditions but only those focused on convulsions analysed for this presentation (n=4)

**Epilepsy Specialist Nurses (ESNs)**

- Specialist nursing service involved with and observers of a wide range of child and family experiences and scenarios – well placed to consider ZDAs for convulsions and suggest opportunities for change moving forward
- ESNs representing five Scottish HB areas (n=7)

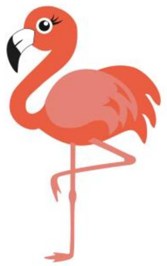

CHIEF SCIENTIST OFFICE

# Findings from the interviews...

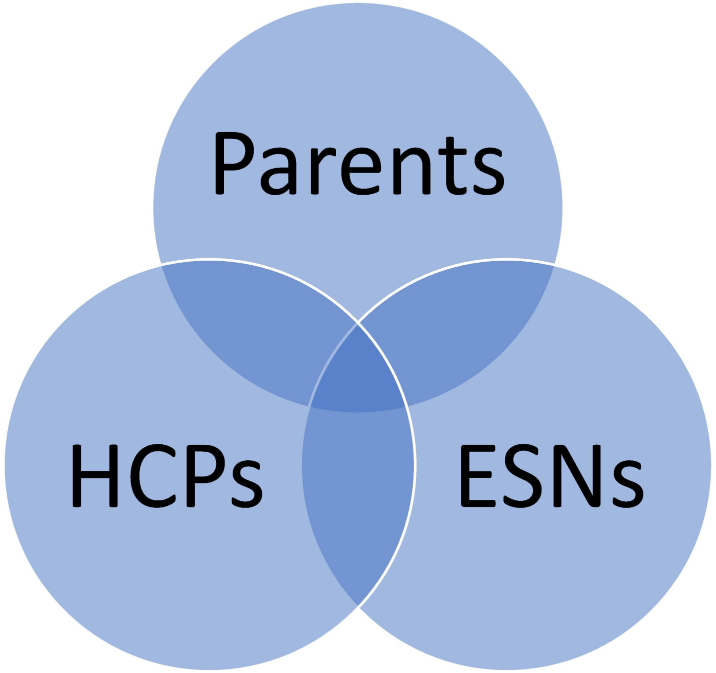


There was consistency and similarity in the themes emerging from interviews with these three different groups.

Analysis and synthesis of the interview data revealed three overarching themes related to experiences of unscheduled hospital attendance or admission of children with seizures

CHIEF SCIENTIST OFFICE

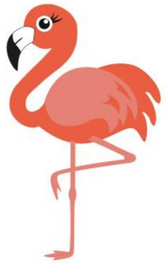

# Overarching themes…

| | |
|---|---|
| **Theme 1:**<br>Anxiety and panic | • The overwhelming feelings of anxiety and panic experienced by parents when their child has a seizure and often contributes to hospital attendance or admission. |
| **Theme 2:**<br>Reassurance, observation and forward planning | • Reassurance and ongoing support for parents through observation of their child following a seizure (by relevant HCP) and forward planning to diagnose new or manage existing seizures. |
| **Theme 3:**<br>Care from the right person, in the right place, at the right time | • Care pathways for acute seizure care that ensures care is provided by a professional with knowledge and expertise of seizures in children, in the most appropriate setting and with timely access. |

CHIEF SCIENTIST OFFICE

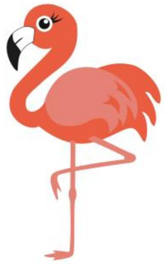

BMJ Publishing Group Limited (BMJ) disclaims all liability and responsibility arising from any reliance placed on this supplemental material which has been supplied by the author(s)


- Formally evaluate best practice around the use of video technology that was accelerated during the COVID-19 pandemic

- Explore the development of more child-oriented algorithms for NHS24 and ambulance service with additional education and training for these professionals.

- Raise awareness of febrile convulsions amongst parents, nurseries and schools, and the wider public.

- Expand the ESN service and nursing staff with expertise around acute seizure assessment and management.

CHIEF SCIENTIST OFFICE

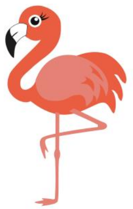

# Prioritising potential system innovations…

CHIEF SCIENTIST OFFICE

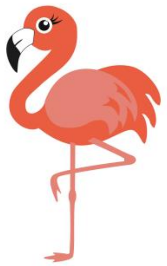

BMJ Publishing Group Limited (BMJ) disclaims all liability and responsibility arising from any reliance placed on this supplemental material which has been supplied by the author(s)


# Prioritising potential system innovations…

- Address gap in acute paediatric skills of healthcare professionals in the community.
- Assessment and observation in community settings
- Holistic children’s hubs linked to Children’s Hospital
- Hospital at Home model
- Specific care pathways for under twos.
- Specific care pathways for wheezy children/bronchiolitis.
- Specific care pathway for seizure.

CHIEF SCIENTIST OFFICE

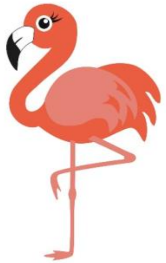

Funded by:

# Questions to consider....

## If you were in charge, what would you change first and why?

- Address gap in acute paediatric skills of healthcare professionals in the community
- Assessment and observation of patients in community settings
- Holistic children's hubs linked to Children's Hospital
- Hospital at Home model
- Specific Care Pathway for under 2 year olds
- Specific Care Pathway for wheezy children/bronchiolitis
- Specific Care Pathway for seizure
- Other ….. please state

## Imagine you are in charge five year after your first choice has been in action. What problems would people come to you with about your "new" service?

See you back here at 2.45 ☺

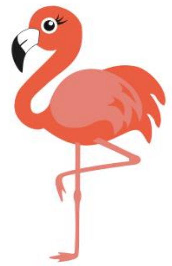

CHIEF SCIENTIST OFFICE

# FLAMINGO
# Flow of hospital admissions in children and young people – Wrap up

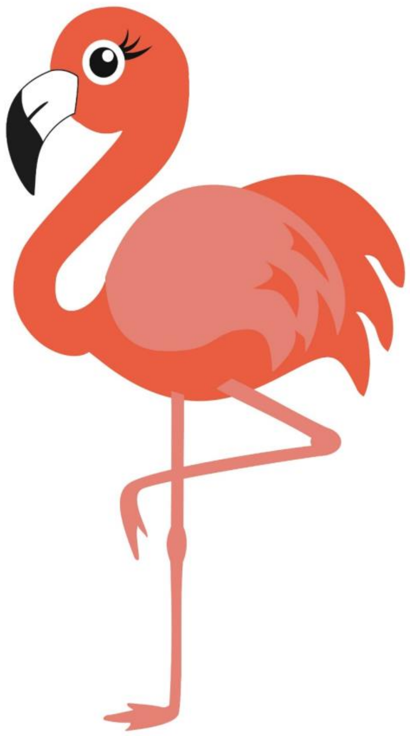

CHIEF SCIENTIST OFFICE

BMJ Publishing Group Limited (BMJ) disclaims all liability and responsibility arising from any reliance placed on this supplemental material which has been supplied by the author(s)


# Why are we meeting?

This is stakeholder engagement

Two main aims

1. Share results with you
   - What surprises you
2. What are the priorities?
   - What would <u>you</u> do first?

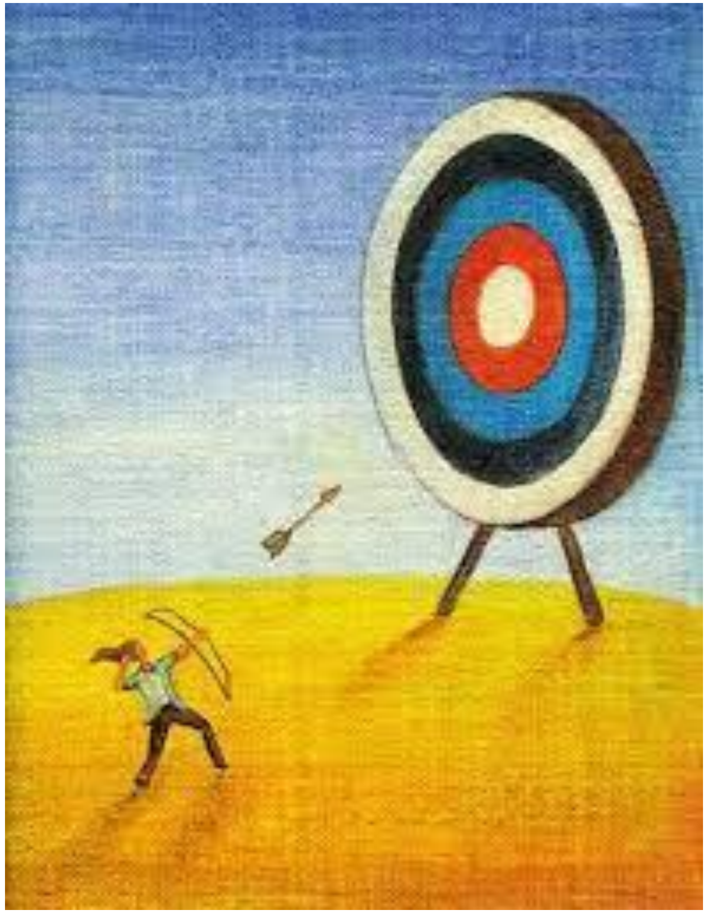

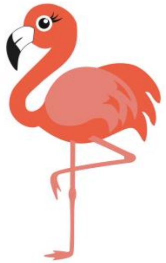

CHIEF SCIENTIST OFFICE

# Why are we meeting?

Please can you find your piece of paper from this morning?

Did you meet that aim?

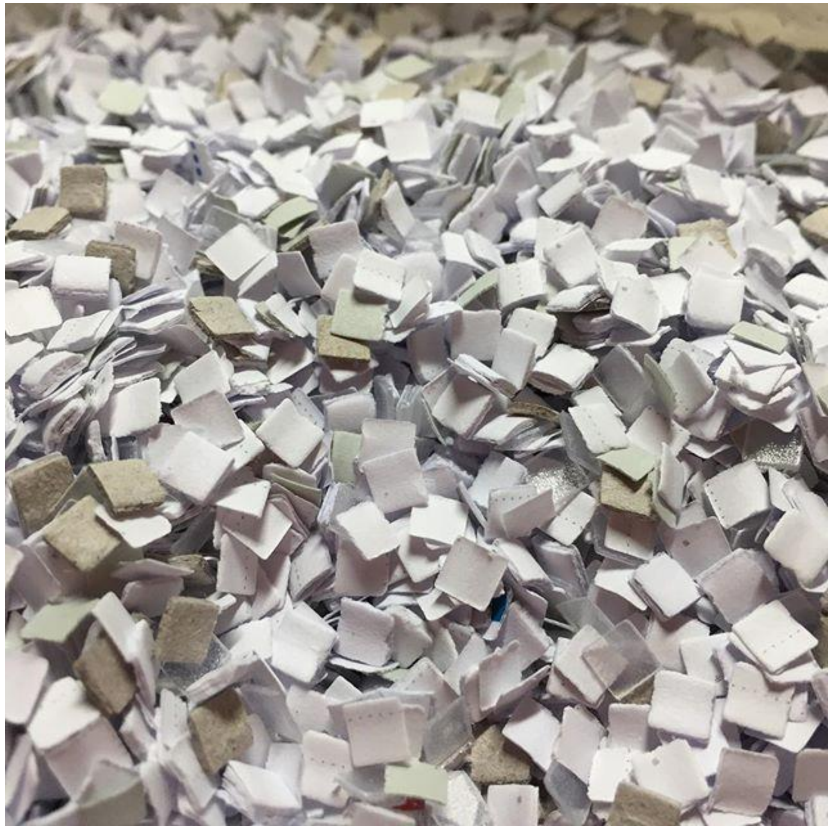

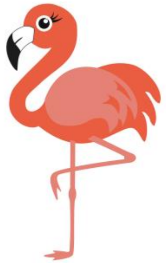

CHIEF SCIENTIST OFFICE

# So where next?

- Finalise analysis
  - Quant
  - Qual
- Write papers
  - Overall findings and methodology
  - Regional variation
  - Seizures
  - Post discharge journey
  - Matched case-control

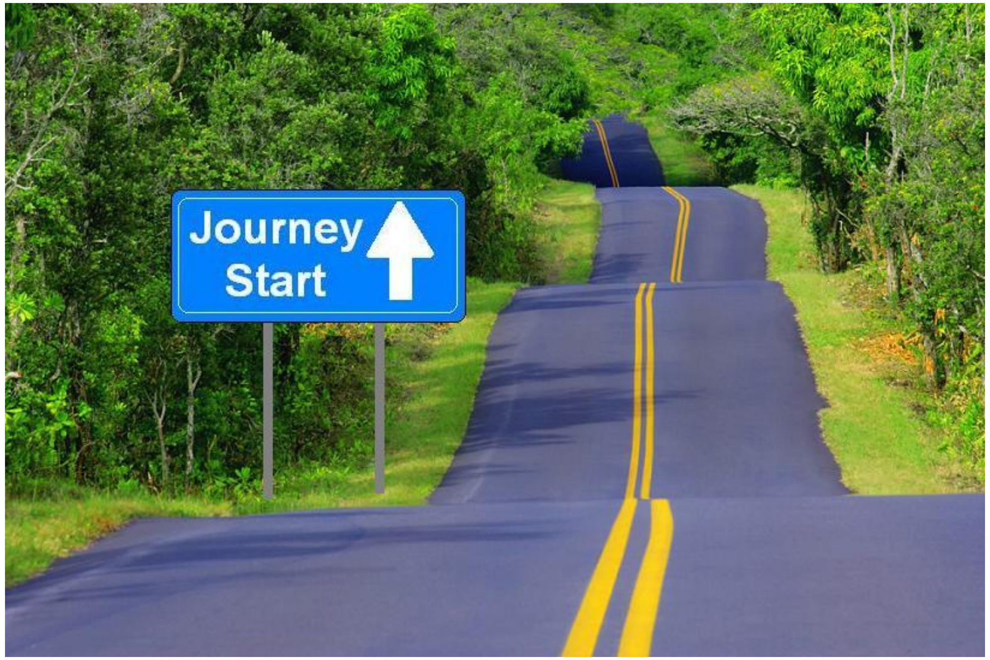


CHIEF SCIENTIST OFFICE

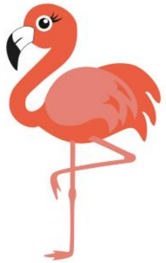

# So where next?

Many potential ways to start

- Agreed referral pathways
- Rapid review clinics
- Communication
- More staff
- New buildings
- Etc

CHIEF SCIENTIST OFFICE

Original research

## Interventions to reduce acute paediatric hospital admissions: a systematic review

Smita Dick ,[1] Clare MacRae,[2] Claire McFaul,[1] Usman Rasul,[1] Philip Wilson ,[3] Stephen W Turner [1]

**What this study adds**

- Since a review of this literature published in 2012, there has been a considerable increase in the number of publications describing interventions aimed at reducing unscheduled admissions.
- Despite these welcome additions to the literature, the evidence available lack robust studies (eg, randomised controlled trials) and is mostly based on single-centre experience.
- There is a need for multifaceted and multicentred interventions using resources from health (primary and secondary care) and social care to tackle the increasing admissions.

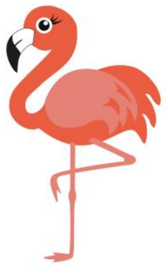

BMJ Publishing Group Limited (BMJ) disclaims all liability and responsibility arising from any reliance placed on this supplemental material which has been supplied by the author(s)


# Thank you all very much

- Could not have done this without you
- Post meeting questionnaire on its way
- Have a fun weekend

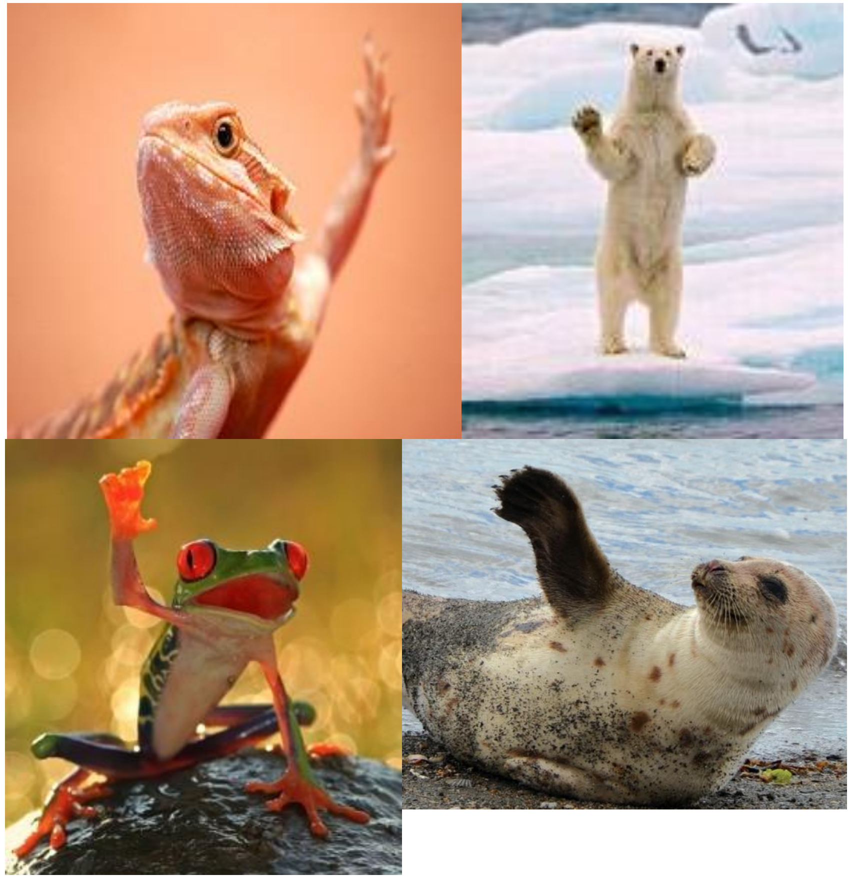

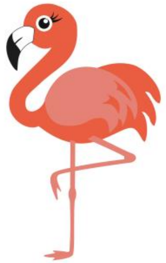

CHIEF SCIENTIST OFFICE
