# Supplementary material for: Identifying and prioritising future interventions with stakeholders to improve paediatric urgent care pathways in Scotland, UK: a mixed-methods study
Source: BMJ Open. 2023 Oct 12;13(10):e074141. doi: 10.1136/bmjopen-2023-074141 (PMC10582902; doi:10.1136/bmjopen-2023-074141)
Supplement: Supplementary data [file bmjopen-2023-074141supp010.pdf]

Supplementary file 7. Characteristics of qualitative interview participants

Full data available in: Malcolm C, King E, France E, et al. Short stay hospital admissions for an acutely unwell child: A qualitative study of outcomes that matter to parents and professionals. *PLoS One.* 2022 Dec 16;17(12):e0278777. doi: 10.1371/journal.pone.0278777. PMID: 36525432; PMCID: PMC9757586.

**Parents and children (n=21)**	
**Characteristic**	**Number (n)**
Parent's relationship to child:	
Mother	20
Father	1
Gender of child:	
Female	5
Male	16
Age of child at time of admission:	
0-5 years	16
6-10 years	4
11-16 years	1
Average SIMD 2020 Decile (range)	5.1 (1-10)
Reason for admission (infection vs non-infection):	
Infection	15
Non-infection	6
Time elapsed since child's admission and the date of interview (months):	
0-24	17
>24	2
Missing data	2
Admission was pre-COVID (pre-March 2020):	
Yes	7
No	14
**Health Professionals (n=48)**	
**Role and Setting**	
**Primary Care/General Practice/OOH Service**	16
**Paediatric Hospital Emergency Department**	18
**District General Hospital Emergency Department**	14
Length of experience (years)	
<5	3
5-30	34
>30	4
Missing data	7
