# Supplementary material for: Identifying and prioritising future interventions with stakeholders to improve paediatric urgent care pathways in Scotland, UK: a mixed-methods study
Source: BMJ Open. 2023 Oct 12;13(10):e074141. doi: 10.1136/bmjopen-2023-074141 (PMC10582902; doi:10.1136/bmjopen-2023-074141)
Supplement: Supplementary data [file bmjopen-2023-074141supp011.pdf]

Full supporting interview quotations for each intervention

**Addressing gaps in acute paediatric skills of health professionals working in community settings**
**Increase specialist acute paediatric nursing roles**
It might be worth thinking about putting ANPPs in GP surgeries. Again, you've got well-experienced paediatric nurses that could go out into the community, see these patients, maybe be able to keep them at home by reassessing during the day, knowing what they're reassessing and also be able to do some teaching with the GPs. I think the way forward is maybe to try and put more paediatric experienced staff out in the community that can see acute unwell children. (C003_Nurse)
I think the emergency department was very busy, we were lucky to get some attention and we were moved fairly promptly down to the children's ward where we had…, it was clearly, [sic]. We were getting more specialist advice and you got much more attention down there, and they were able to fit the nebuliser properly onto my son's face which they hadn't really done in the ED. (Parent P002)
**Provide additional paediatric urgent care training for GPs**
Yeah, it's also unfortunate but true that paediatrics is not a requirement for training in general practice. So, you will unfortunately get, it's sad to say but the quality of referrals from primary care can be very poor. So, you have practitioners who are not prepared to take any chances themselves because of a lack of experience and this is a big problem for us in the winter months. (C014_Consultant)
I think training the GPs and the OOHs staff to have more experience and more confidence in paediatric problems should theoretically help but actually practically doing that is very, very difficult to get a significant amount of teaching and learning across to the body of individuals that work in primary care, so it's challenging, it's very difficult. (C005_Consultant)
I did quite a lot of paediatric training here, I didn't do any psychiatry […] but I managed to muddle through with psychiatry over the years but the paediatric training I felt was pretty essential […]. They [GP trainees] can breeze through without anything formally and paediatrics, you know, there's an enormous amount of pitfalls there with paediatrics. (C038_GP)

Then she [GP] went to do his SATS, so she had one of those little probes, sort of like a completely mobile standalone thing and he wriggled and fussed […] she did take it several times although my concern was it was an adult probe, so I was thinking it's probably not particularly accurate, but anyway she did try several times, she was only getting 86 and she did seem to have a good trace getting 86. […] So [GP] phoned [hospital] from the GP surgery and said 'I have this boy, he's known to you, I can only get SATS of 86, are you happy for him to come in?' So they said 'yes but he needs to come in an ambulance if his SATS are 86, he needs oxygen'. […] They [hospital] put a SATS monitor on him but for a small child and his SATS were like 99/100, so I think we were all like 'oh god, this seems such a waste of everyone's time and resources!' (Parent P010)
**Create rotational posts between primary and secondary care settings**
Part of my role when they took me on in post […] was going to be facilitation of learning and development of other people's skills. So, because COVID came in we like every other service had to set up a COVID assessment centre […]. So again, these were adult trained clinicians that came in and didn't have any paediatric experience, so they were heavily relying on GP support within the in-hours period if children were referred […]. So for a wee while as the schools and nurseries all started to go back we had a bit of a boom in paediatric presentations and I actually came out of my out of hours post for a period of four weeks to support in-hours so that there was somebody there that could see the children, so that there was somebody there that could if the nurse practitioners wanted to come in and shadow. I did a little bit of just ad hoc teaching there. […] So it is something that I have tried to look into and I am intermittently just trying to push on and do little bits of ad hoc education as and when I can but, like I say, unfortunately COVID impacted all of our plans for pushing forward with that. […]. I suppose I'm quite lucky that I have got established relationships with the paediatric ward because that's where I came from. So I do have an established support network if you like, and established relationships. (C027_Nurse)
Yeah I'm a GP […]I also am piloting at the moment a joint clinic with a couple of the consultant paediatricians […]. We have a community child health consultant once a month and a general medical consultant once a month that we co-consult with on some more kind of challenging cases to try and prevent access issues and things that can potentially be dealt with in primary care. […]. I also do out of hours, so occasionally work in Hospital6-1 as a GP, but seeing the stuff that gets triaged from Hospital6-1 as being more GP stuff, so you know, it's often viral things and things like that, so I do that as well. (C031_GP)
**Assessment and observation over time in community settings**
Sometimes very obviously the correct thing to do is not to discharge the child but to give a slightly longer period of observation. (C018_Consultant)

I think the main thing with a lot of these kids is actually just time and giving them a chance to let their anti-pyretic settle and giving them a bit of fluid and just a period of observation, which I think is the limiting factor in GP practices and in GP out of hours, you know, they just don't have the facilities to be able to watch these children for a period of time. (C015_Doctor)
They [children] can't tell you what's wrong, you know, so you might just have a baby that's a bit fractious or maybe not feeding as well, really soft signs, and I don't think we have the facilities even really to get all the accurate measurements that we would want, we don't have a paediatric SATS monitor, we can't do blood pressure in children either, so we're basing things more on their heart rate and their temperature and it's maybe kind of soft but just the general way they are. (C013_GP)
**Creation of holistic children's 'hubs'**
What I find is that then these children are born and there's nothing else for children and families apart from a health visitor and actually, you know, it's almost like if we could have hubs and community hubs where if you come in to get our health visiting weighed and things like that, you get taught about childhood diseases and when to worry, you know, and so almost like a mass public education programme that you get taught about when to worry, about when your child is sick. (C002_Consultant)
Interviewer: And how did you feel about taking him home [from hospital] at that point, did you feel fairly confident that you could look after, you know, if it was just bronchiolitis that you could look after that?<br><br>*Respondent: No, not at all [laugh] no I wasn't, no, I wasn't confident. I mean, he's a baby with the cold and I'll have to manage it but at that point he still wasn't feeding well, I probably wasn't very well at that point, yeah I was mentally not very well at that point and did not feel equipped to look after him at home, but then I guess I didn't know what else they could realistically do for him in hospital. So I was accepting of it.* [Parent P018]
Having a facility where you can sit and watch them, whether or not you actually do a specific intervention, even if you can just watch them and repeat their observations over an hour or two, we have found that that can help. We had a period of time where one of our consultants went out and spent time in the out-of-hours service and persuaded them to set aside an area in the out-of-hours building where children could wait and be observed. […]. You need space and

you need the appropriate staff to look after them, you can't just sit them down and abandon them, so you need staff that are focused on acute assessment and acute treatment, you need a geographical location that allows you to do that. (C005_Consultant)
**Hospital at Home model**
It's ebbs and flows and there are periods of times when you seem to get a number of referrals which you think, 'Surely that could be handled in the community, or can be managed in a different way rather than coming into hospital,' yeah. As to whether they could've handed in their urine sample of something, went away and then you can advise on what they're doing at home and representing, you know, safety-netting and so on. Or there are certain things where you think actually the best way to handle the particular scenario would've been to speak to someone who actually… rather than bring them in the night - and that happens fairly often, I would think, where a referral is received at five o'clock in the evening or something along those lines, and you know that they need some investigation or imaging that isn't going to happen that night or they're going to need to see, for instance, a child and adult mental health person, and that's not going to happen in the middle of the night - and you think, actually, that child could potentially be risk assessed and managed at home and then referred to the appropriate services the next day. (C020_Consultant)
The 111 nurse [NHS24]*, she was just [laugh], I think because I was obviously slightly panicked because he was bringing up foam and he was unable to breathe properly, so I was obviously like 'oh my gosh he's not breathing properly' and she was like 'calm down' and I was like 'aargh!' [laugh]. So I think, yeah, the 111 nurse [laugh] probably thought I was a completely over-anxious mum, but this was the worst that he's had the croup so I'd never seen him that poorly apart from when he was on neonatal, so I think it probably brought back some memories and I probably did panic. But the clinicians at the hospital were great, I mean, I explained to the doctor that I didn't want him catching anything and she was lovely and very understanding and said 'we'll try and send you home as soon as we can'.* (Parent P007)
**Extend specialised care pathways for sub-groups of children**
There are specialist nurses for most specialties, so there's specialist nurses for diabetes, specialist nurses for oncology, specialist nurse for cystic fibrosis, asthma, so […] different specialist nurses who are usually the first contact for such parents. […] So, yeah, these long-term conditions, we have changed completely the way we do it. (C010_Consultant)

Yeah, well again if they have a diagnosis of epilepsy and they've got a paediatric epilepsy nurse specialist in their area I think it's pretty straightforward for them, they have a clear plan of what to do and who to contact. As I say, my job is to keep them out of hospital so in between clinic appointments they would be phoning myself if they have a seizure, they're advised to phone myself, I'm a nurse prescriber so I change medications over the phone. A medication is never just prescribed without a clear pathway of how to increase it, when to increase it, what the maximum dose is […] so they [parents] always know that they can do something because it's mainly the helplessness that causes anxiety with epilepsy and the parents not being able to have control of the situation, whereas if you're giving them a wee bit of control of what to do and you give them permission to increase the medicine with a clear plan. I definitely find that cuts down the amount of phone calls you get, it cuts down the anxiety. (C041_Epilepsy Specialist Nurse)
Fevers in young children, vomiting in young children definitely are huge areas where we get lots and lots of referrals. Children under three months with a temperature, absolutely right to refer because it could be a septic baby but, like a two- or three-year-old who has had a temperature for 24 hours it's giving them the right advice by the GP that they can have Paracetamol and Ibuprofen […]. So I think fever in young children, vomiting in young children and respiratory illnesses. (C003_Nurse)
