# Supplementary material for: Identifying and prioritising future interventions with stakeholders to improve paediatric urgent care pathways in Scotland, UK: a mixed-methods study
Source: BMJ Open. 2023 Oct 12;13(10):e074141. doi: 10.1136/bmjopen-2023-074141 (PMC10582902; doi:10.1136/bmjopen-2023-074141)
Supplement: Author's manuscript [file bmjopen-2023-074141.draft_revisions.pdf]

BMJ Open is committed to open peer review. As part of this commitment we make the peer review history of every article we publish publicly available.

When an article is published we post the peer reviewers' comments and the authors' responses online. We also post the versions of the paper that were used during peer review. These are the versions that the peer review comments apply to.

The versions of the paper that follow are the versions that were submitted during the peer review process. They are not the versions of record or the final published versions. They should not be cited or distributed as the published version of this manuscript.

BMJ Open is an open access journal and the full, final, typeset and author-corrected version of record of the manuscript is available on our site with no access controls, subscription charges or pay-per-view fees (http://bmjopen.bmj.com).

If you have any questions on BMJ Open's open peer review process please email info.bmjopen@bmj.com

BMJ Open

Identifying and prioritising future interventions with stakeholders to improve paediatric urgent care pathways in Scotland, UK: a mixed-methods study

Journal:	*BMJ Open*
Manuscript ID	bmjopen-2023-074141
Article Type:	Original research
Date Submitted by the Author:	29-Mar-2023
Complete List of Authors:	King, Emma; University of Stirling Department of Nursing and Midwifery,<br>France, Emma; University of Stirling, NMAHP Research Unit, School of Health Sciences<br>Malcolm, Cari; Edinburgh Napier University, School of Health and Social Care<br>Kumar, Simita; Public Health Scotland; Children's Health Scotland<br>Dick, Smita; University of Aberdeen, Child Health<br>Kyle, Richard G; University of Exeter<br>Wilson, Philip; University of Aberdeen, Institute of Health and Wellbeing<br>Aucott, Lorna; University of Aberdeen , Medical Statistics Team<br>Turner, Stephen; University of Aberdeen, Department of Child Health<br>Hoddinott, Pat; University of Stirling, Nursing, Midwifery and Allied Health Professional Research Unit
Keywords:	Patient Participation, Paediatric A&E and ambulatory care < PAEDIATRICS, QUALITATIVE RESEARCH, ACCIDENT & EMERGENCY MEDICINE

SCHOLARONE™
Manuscripts

I, the Submitting Author has the right to grant and does grant on behalf of all authors of the Work (as defined in the below author licence), an exclusive licence and/or a non-exclusive licence for contributions from authors who are: i) UK Crown employees; ii) where BMJ has agreed a CC-BY licence shall apply, and/or iii) in accordance with the terms applicable for US Federal Government officers or employees acting as part of their official duties; on a worldwide, perpetual, irrevocable, royalty-free basis to BMJ Publishing Group Ltd ("BMJ") its licensees and where the relevant Journal is co-owned by BMJ to the co-owners of the Journal, to publish the Work in this journal and any other BMJ products and to exploit all rights, as set out in our licence.

The Submitting Author accepts and understands that any supply made under these terms is made by BMJ to the Submitting Author unless you are acting as an employee on behalf of your employer or a postgraduate student of an affiliated institution which is paying any applicable article publishing charge ("APC") for Open Access articles. Where the Submitting Author wishes to make the Work available on an Open Access basis (and intends to pay the relevant APC), the terms of reuse of such Open Access shall be governed by a Creative Commons licence – details of these licences and which Creative Commons licence will apply to this Work are set out in our licence referred to above.

Other than as permitted in any relevant BMJ Author's Self Archiving Policies, I confirm this Work has not been accepted for publication elsewhere, is not being considered for publication elsewhere and does not duplicate material already published. I confirm all authors consent to publication of this Work and authorise the granting of this licence.

For peer review only

TITLE

IDENTIFYING AND PRIORITISING FUTURE INTERVENTIONS WITH STAKEHOLDERS TO IMPROVE PAEDIATRIC URGENT CARE PATHWAYS IN SCOTLAND, UK: A MIXED-METHODS STUDY

Authors:

E King (corresponding author)[1], E France[1], C Malcolm[2], S Kumar[3], S Dick[4], R G Kyle[5], P Wilson[4], L Aucott[4,7] S Turner[4,8], P Hoddinott[1]

[1]Nursing, Midwifery and Allied Health Professions Research Unit, University of Stirling

[2] School of Health Sciences, University of Dundee

[3]Screening and Immunisation, Public Health Scotland

[4] Child Health, University of Aberdeen

[5] Academy of Nursing, Department of Health and Care Professions, Faculty of Health and Life Sciences, University of Exeter

[6] Centre for Rural Health, University of Aberdeen

[7] Centre for Randomised Healthcare Trials, University of Aberdeen

[8] NHS Grampian, Aberdeen

Corresponding author: emma.king@stir.ac.uk

KEY WORDS

Urgent care, paediatrics, Patient and Public Involvement, short stay admission, qualitative, mixed methods

For peer review only - http://bmjopen.bmj.com/site/about/guidelines.xhtml

ABSTRACT

Objectives

To identify, and prioritise interventions, from the perspectives of parents and health professionals, which may be alternatives to current unscheduled paediatric urgent care pathways.

Design

FLAMINGO is a mixed-methods study with Public and Patient Involvement (PPI) throughout. Quantitative data from urgent admissions and three referral sources: emergency department (ED), out of hours service (OOH), and general practice (GP), and qualitative data from interviews were presented and discussed at a stakeholder intervention prioritisation event.

Setting

National Health Service (NHS) in Scotland, UK.

Participants

Quantitative data: children with urgent medical admission to hospital from 2015-2017. Qualitative interviews**:** parents and health professionals with experiences of urgent short stay hospital admissions of children. PPI engagement was conducted with nine parent-toddler groups and a university-based PPI advisory group. Stakeholder event: parents, health professionals, and representatives from Scottish Government, academia, charities and PPI attended.

Results

Data for 171,039 admissions which included 92,229 short stay admissions (SSAs) were analysed and 48 health professionals and 21 parents were interviewed. The stakeholder event included 7 parents, 12 health professionals and 28 other stakeholders. Analysis and synthesis of all data identified seven interventions which were prioritised at the stakeholder event: 1) addressing gaps in acute paediatric skills of health professionals working in community settings; 2) assessment and observation of acutely unwell children in community settings; 3) creation of holistic children's 'hubs;' 4) adoption of 'hospital at home' models; and three specialised care pathways for sub-groups of children 5)convulsions, 6) being aged <2 years old, and 7) wheeze/bronchiolitis. 'Stakeholders prioritised interventions 1, 2 and 3; these could

For peer review only - http://bmjopen.bmj.com/site/about/guidelines.xhtml

be combined into a whole population intervention. Barriers to progressing these include resources, staffing and rurality.

Conclusions

Health professionals and families want future interventions that are patient-centred, community-based, and aligned to outcomes that matter.

STRENGTHS AND LIMITATIONS OF THIS STUDY

- Analysis of multiple sources of data from a robust mixed-methods study allowed us to identify interventions that stakeholders prioritised.
- Health professional contributions represented most professional groups providing care to acutely unwell children.
- Parents did not suggest specific interventions as solutions but shared their experiences and their values.
- Children's and paramedics' views are not represented.
- Solutions might differ for remote and rural communities and in a post-pandemic context.

For peer review only - http://bmjopen.bmj.com/site/about/guidelines.xhtml

INTRODUCTION

Unscheduled hospital admissions of children in the United Kingdom (UK) have increased steadily over past decades, largely due to a rise in urgent short stay admissions (SSAs) [1, 2]. The rate of hospital medical admissions for children with acute illness in Scotland rose by 49% between 2000 and 2013, with SSAs rising by 186% from 8.6 to 24.6/1000 children per annum [2]. Children under two years of age account for the largest proportion of urgent SSAs [1], with upper and lower respiratory tract infections being a major reason for parents seeking an assessment [2]. Families can face difficulty obtaining urgent medical attention for their acutely unwell children due to pressures on the National Health Service (NHS), particularly during seasonal peaks of viral infections, so changes to the present pathways of care are needed [3]. Urgent admissions initially fell rapidly during the COVID-19 pandemic [4], however in December 2021 they were 38% higher than pre-Covid [5].

A 2012 systematic review found no evidence of effective interventions for reducing admission rates [6] and a recent update [7] found a lack of high-quality evidence to support interventions aimed at preventing paediatric admissions. There is therefore a need for effective interventions to improve paediatric urgent care pathways [7]. It is not known which parts of the pathway interventions should target, e.g. particular clinical settings or clinical presentations, or which interventions to develop [6, 7]. The identification and development of complex interventions needs to take account of, and build upon, existing evidence, and be conducted in collaboration with patient and public involvement (PPI) and other stakeholders [8, 9]. Our aim is to use all data with PPI from the mixed-methods FLAMINGO (**FL**ow of **A**d**M**issions in ch**I**ldren and you**NG** pe**O**ple) study to identify,and prioritise from the perspectives of parents and health professionals, interventions which may improve the efficacy of current unscheduled urgent care pathways for children for future research and development.

METHODS

Study design

FLAMINGO is a mixed-methods study and this paper describes the identification of potential interventions, informed by the INDEX Approach [9] which has not been followed previously to address this problem. It draws together findings from across our team's work:

For peer review only - http://bmjopen.bmj.com/site/about/guidelines.xhtml

- linked national datasets examining pre-referral pathways and characteristics of SSAs of children to Scottish hospitals. Methods are reported in supplementary file 1 and in Dick *et al.* [10]

- qualitative interviews with parents and health professionals providing insights into their values (including an important shared outcome of preserving the child's safety) [11] and experiences of unscheduled urgent care for children with SSAs and suggestions for change. Methods are reported in supplementary file 1 and in Malcolm *et al*. [11]

- PPI as a core component of the FLAMINGO project [12, 13].

- a systematic review of hospital-based interventions to reduce acute paediatric admissions [7]. This was used to help identify interventions, along with data from the quantitative and qualitative research and PPI input.

- a stakeholder event to discuss, debate and prioritise identified interventions for further research and development.

FLAMINGO was undertaken in Scotland, where the NHS is organised into 14 geographically distinct Health Boards, each responsible for healthcare provision to their region's population. The project ran from January 2019 to December 2021 (Figure 1). The FLAMINGO team met monthly, the quantitative and qualitative sub-teams met separately in between.

The study was approved by the NHS North of Scotland Research Ethics Service (REC reference: 19/NS/0134).

Figure 1. Project timeline

Patient and Public Involvement

PPI was established at the outset to ensure the views of families were considered throughout all project stages. Attendance at parent-toddler groups, including those accommodating people from lower socio-economic groups and minority ethnic backgrounds, and a university-based PPI advisory group, enabled parents to share their experiences of accessing healthcare and attending hospital for urgent healthcare. Their experiences informed the qualitative interview topic guide; ensured the materials used for recruitment were appropriate; and their experiences of urgent care supplemented the qualitative data to

For peer review only - http://bmjopen.bmj.com/site/about/guidelines.xhtml

inform ideas for potential interventions. An independent PPI advisor critically reviewed and commented on manuscript drafts.

Intervention identification and development

Data Collection

The intervention development approach was target population-centred, incorporating front-line health professional and parent perspectives collected through PPI and qualitative interviews [9]. Potential future interventions were developed by the qualitative team [CM, EK, EF, PH] combining and interpreting FLAMINGO data, a systematic review [7], and the quantitative data showing the large contribution that wheeze/bronchitis and under 2's have to the number of paediatric SSAs.

Semi-structured interviews questions to health professionals asked about potential improvements to pathways for children between home and urgent SSAs; parents were asked about how their family's experiences could be improved in future. Interview topic guides are in Supplementary files 2 and 3 and Malcolm *et al.* [11]. Participant information sheets are included in Supplementary file 4. For identification of interventions to take forward for prioritisation, an intervention was defined as a change/innovation where there is equipoise, i.e. no evidence to support effectiveness, so it would need further research on acceptability, feasibility, effectiveness and cost-effectiveness before implementation.

A logic model was developed early in the study (Figure 2) informed by a Directed Acyclic Graph (DAG) model (a causal diagram) [14, 15]. The DAG was referred to iteratively throughout the FLAMINGO project and incorporated into a revised/refined logic model once the data analysis was complete (Supplementary file 5).

Figure 2: Original system/process logic model – the acutely sick child. May 2020

Data analysis

The shared outcomes of care that are important to both health professionals and parents to inform the design of a new care pathway for acutely unwell children are: prioritising child safety; resolving uncertainty and anxiety about the illness trajectory; parents greatly value timely access to care from

For peer review only

experienced paediatric staff; and health professionals acknowledge the need to improve system pathways for pre-hospital care and support for families within the community [11].

Interventions were identified from the qualitative interview data through the following steps, guided by framework analysis for applied policy research [16] applied in QSR International NVivo 12 software: familiarisation with transcripts; developing and agreeing a coding frame; indexing and further refinement of the coding framework; charting; and mapping and interpretation to search for patterns and explanations in the data. In-depth analysis was undertaken by EK and PH of potential solutions suggested by health professionals to improve urgent care pathways for children, any experiences of initiatives undertaken in their clinical settings, what had worked well and less well, and any consequences together with drawing on the collective experiences of their professions. Similarly, in-depth analysis was undertaken of parents' accounts through both PPI consultation and interviews about how their family's experiences could be improved in future. Families mainly voiced their problems and experiences, therefore possible solutions were implicit, whereas HCPs often suggested solutions explicitly. The team discussed the potential interventions generated through this process and decided whether each one met our intervention definition.

Reference was made to an underpinning systematic review of interventions to reduce acute paediatric hospital admissions, conducted by a FLAMINGO sub-team, to generate a final list of interventions for stakeholders to prioritise [7]. The review findings were shared with the FLAMINGO qualitative team (EK, CM, EF, PH) once qualitative data collection was complete (Table 1). The review of mostly hospital-based/secondary care interventions in 28 studies [7] identified four groups of interventions: condition specific care pathways; staff reconfiguration; new building; and telemedicine. Telemedicine interventions were ruled out of our list of interventions to prioritise, based on the qualitative data from professionals and parents; there had been a huge shift to using video/telephone consultation during the COVID19 pandemic, therefore there was no longer equipoise. The review did not identify any additional interventions addressing the entire care pathway from home to hospital which showed sufficient promise to pursue in our prioritisation.

For peer review only - http://bmjopen.bmj.com/site/about/guidelines.xhtml

Intervention prioritisation

Data Collection

We held an interactive stakeholder event to discuss and prioritise potential interventions on 3 September 2021. Attendees could attend in person or by video conference and included parents, health professionals, and representatives from stakeholder organisations, e.g., charities and parent groups, government. Six discussion groups with attendees were facilitated and audio-recorded by a team member (SD, EF, EK, ST, CM, RGK, or PW) who also took written notes. Attendees individually ranked the priority of the interventions after the group discussions. Detailed methods are in Supplement 6.

Data analysis

Notes and transcripts from stakeholder group discussions were analysed in NVivo (by EF and EK) by coding data under 15 broad codes corresponding to confirming versus disconfirming perspectives for each of the interventions and a further code for other interventions suggested by attendees. Coded data were read repeatedly and themes identified. For the intervention priority ranking exercise, scores were summed and the average rank calculated for each of the identified interventions.

RESULTS

Intervention identification and development

The results report how different evidence sources informed a short-list of interventions for prioritisation at the stakeholder workshop. Data were available from 92,229 SSA admissions, which were linked to other databases [10], and interviews with 48 health professionals - including GPs, hospital doctors and consultants, community nurses, and hospital nurses - and 21 parents - 20 mothers and one father of a child who had had a SSA for acute illness. Characteristics of participants are provided in supplementary file 7 and elsewhere [11]. We involved nine parent-toddler groups and one university-based PPI advisory group attended by 112 adults and 107 children.

For peer review only

Identification of interventions

Analysis of all project data and a systematic review [7] identified seven potential interventions meeting our definition, four whole-system: 1) addressing gaps in acute paediatric skills of health professionals working in community settings, 2) assessment and observation of acutley unwell children in community settings, 3) creation of holistic children's 'hubs,' 4) adoption of 'hospital at home' models, and three sub-group interventions extending specialised care pathways for: 5)convulsions, 6) children age <2 years old, and 7) wheeze, cough, shortness of breath/bronchiolitis. Table 1 summarises FLAMINGO data linkage results and systematic review data that support each intervention.

For peer review only

For peer review only - http://bmjopen.bmj.com/site/about/guidelines.xhtml

Table 1. Summarised quantitative and review data that informed intervention selection, triangulated with the qualitative data, for the stakeholder event

Intervention	FLAMINGO Data linkage results [10, 17]	Systematic review evidence [7] and other evidence sources.
Addressing gaps in acute paediatric skills of health professionals working in community settings	Admissions of children with respiratory infections dominated SSAs from all referral sources indicating the need to improve skills and confidence in management of respiratory infections. Asthma, bronchiolitis, croup, lower respiratory tract infection and cough/wheeze/shortness of breath accounted for a total of 17,764 SSA (19% all SSA)<br><br>Hypothesis: increasing paediatric skills of community staff in management of respiratory infections may benefit child outcomes and/or reduce SSA's	Not addressed by the review<br><br>Supported by other studies showing increasing proportion of SSA 2000-2013 [2]; a recognition that conditions may have been previously managed in the community [2, 3]; knowledge that ~70% of GPs have no post graduate paediatric training.
Assessment and observation of acutely unwell children in community settings	N/A	The review focused on interventions in secondary care only
Creation of holistic children's 'hubs'	There were twice as many SSAs for children from the most deprived compared to the least deprived communities (27% (n=25022) vs 13% (n=12032). Those referred by ED (n=29461) were over represented by children from white ethnic groups 72.5 % (n=21360) v non-white 27.5 % (n=8101); .<br>Hypothesis: interventions in deprived communities may improve engagement with primary care; they may improve parent and child outcomes and/or reduce	The review focused on interventions in secondary care only<br><br>Other evidence supports targeting more disadvantaged communities. [18, 19]

For peer review only - http://bmjopen.bmj.com/site/about/guidelines.xhtml

	SSAs.	
Adoption of 'hospital at home' models	Of all SSA's (n=92229) n=12378 readmissions within 30 days<br>Hypothesis: Hospital at home may improve parental and child outcomes; this may reduce the number of readmissions	The review focused on interventions delivered in secondary care only. There is some evidence to support this. [20]
Extending specialised care pathways for convulsions	Ten composite diagnoses accounted for 52% (n=47959) of SSA: asthma, bronchiolitis, convulsion (including febrile and afebrile convulsions), croup, gastroenteritis, upper respiratory tract infection (URTI), viral infection, tonsillitis, lower respiratory tract infections (LRTI) and admissions with a diagnosis of cough or wheeze or shortness of breath.<br>Convulsions are most commonly admitted directly from ED, rather than through OOH or GP referral.<br>Hypothesis: targeted interventions to the urgent care pathway that focus on specific diagnoses may improve parental and child outcomes and/or may reduce SSAs	Evidence from the review was that care pathways for specific conditions are instrumental in reducing admissions especially when they are standardized.<br>There was considerable heterogeneity between the studies and no randomised controlled trials were included.
Extending specialised care pathways for children age <2 years old	Most SSAs were for children under two years- of the 92,229 children with an SSA, 44,063 (48%) were <2 years, 28,306 (31%) were <1. The median age for SSA was 2.2 years (interquartile values 0.74-5.8).	Not included in the review<br>Supported by other evidence.[21]
Extending specialised care pathways for wheeze/bronchiolitis	Prevalence of respiratory symptoms (19% of all SSA) described above.	As described above

For peer review only - http://bmjopen.bmj.com/site/about/guidelines.xhtml

The interventions align with reports published by Scottish Government since the early 1970s which advocate care in the community [22-24]. The perspectives of professionals, parents and PPI supporting the interventions are described with selected illustrative quotations in Table 2 and further quotations in Supplementary File 8.

**Table 2 Selected supporting interview quotations for each intervention**
**Addressing gaps in acute paediatric skills of health professionals working in community settings**
**Increase specialist acute paediatric nursing roles**
It might be worth thinking about putting ANPPs in GP surgeries. Again, you've got well-experienced paediatric nurses that could go out into the community, see these patients, maybe be able to keep them at home by reassessing during the day, knowing what they're reassessing and also be able to do some teaching with the GPs. I think the way forward is maybe to try and put more paediatric experienced staff out in the community that can see acute unwell children. (C003_Nurse)
**Provide additional paediatric urgent care training for GPs**
Yeah, it's also unfortunate but true that paediatrics is not a requirement for training in general practice. So, you will unfortunately get, it's sad to say but the quality of referrals from primary care can be very poor. So, you have practitioners who are not prepared to take any chances themselves because of a lack of experience and this is a big problem for us in the winter months. (C014_Consultant)
Then she [GP] went to do his SATS, so she had one of those little probes, sort of like a completely mobile standalone thing and he wriggled and fussed […] she did take it several times although my concern was it was an adult probe, so I was thinking it's probably not particularly accurate, but anyway she did try several times, she was only getting 86. They [hospital] put a SATS monitor on him but for a small child and his SATS were like 99/100, so I think we were all like 'oh god, this seems such a waste of everyone's time and resources!' (Parent P010)
**Create rotational posts between primary and secondary care settings**
Part of my role when they took me on in post […] was going to be facilitation of learning and development of other people's skills. So, because COVID came in we like every other service had to set up a COVID assessment centre […] So for a wee while as the schools and nurseries all started to go back we had a bit of a boom in paediatric presentations and I actually came out of my out of hours post for a period of four weeks to support in-hours so that there was somebody there that could see the children, so that there was somebody there that could if the nurse practitioners wanted to come in and shadow. [..] I suppose I'm quite lucky that I have got established relationships with the paediatric ward because that's where I came from. (C027_Nurse)

For peer review only - http://bmjopen.bmj.com/site/about/guidelines.xhtml

Yeah I'm a GP […]I also am piloting at the moment a joint clinic with a couple of the consultant paediatricians […]. We have a community child health consultant once a month and a general medical consultant once a month that we co-consult with on some more kind of challenging cases to try and prevent access issues and things that can potentially be dealt with in primary care. […]. (C031_GP)
**Assessment and observation over time in community settings**
I think the main thing with a lot of these kids is actually just time and giving them a chance to let their anti-pyretic settle and giving them a bit of fluid and just a period of observation, which I think is the limiting factor in GP practices and in GP out of hours, you know, they just don't have the facilities to be able to watch these children for a period of time. (C015_Doctor)
**Creation of holistic children's 'hubs'**
What I find is that then these children are born and there's nothing else for children and families apart from a health visitor and actually, you know, it's almost like if we could have hubs and community hubs where if you come in to get our health visiting weighed and things like that, you get taught about childhood diseases and when to worry, you know, and so almost like a mass public education programme that you get taught about when to worry, about when your child is sick. (C002_Consultant)
**Hospital at Home model**
It's ebbs and flows and there are periods of times when you seem to get a number of referrals which you think, 'Surely that could be handled in the community, or can be managed in a different way rather than coming into hospital,' yeah. As to whether they could've handed in their urine sample of something, went away and then you can advise on what they're doing at home and representing, you know, safety-netting and so on. Or there are certain things where you think actually the best way to handle the particular scenario would've been to speak to someone who actually..[…] where a referral is received at five o'clock in the evening or something along those lines, and you know that they need some investigation or imaging that isn't going to happen that night […], actually, that child could potentially be risk assessed and managed at home and then referred to the appropriate services the next day. (C020_Consultant)
**Extend specialised care pathways for sub-groups of children**
If they have a diagnosis of epilepsy and they've got a paediatric epilepsy nurse specialist in their area I think it's pretty straightforward for them, they have a clear plan of what to do and who to contact. As I say, my job is to keep them out of hospital so in between clinic appointments they would be phoning myself if they have a seizure, they're advised to phone myself […] (C041_Epilepsy Specialist Nurse)

For peer review only - http://bmjopen.bmj.com/site/about/guidelines.xhtml

For peer review only

Addressing gaps in acute paediatric skills of health professionals working in community settings

Health professional interviewees described how staff working in the community often lack the necessary training and experience to manage acute paediatric conditions which could contribute to the increasing referrals of children to hospital. Three potential changes to the provision of care were identified: i) increase specialist acute paediatric nursing roles, ii) provide additional paediatric training for GPs, and iii) create rotational posts between primary and secondary care settings.

Increase specialist acute paediatric nursing roles

Professionals and parents perceive an assessment by a health professional with acute paediatric skills, at an early stage in the acutely unwell child's pathway, as beneficial. Parents see specialist children's hospitals with a dedicated paediatric ED as a key advantage, with some criticising the lack of paediatric expertise in a general hospital ED. Health professionals recognise the potential benefits of increasing Advanced Nurse Practitioners with specialist paediatric clinical assessment skills (ANPPs), within community settings and EDs not located in dedicated children's hospitals. ANPPs require the autonomy to act as senior decision makers working with general practices may facilitate the management of acutely unwell children at home safely and with appropriate referral to other services.

Provide additional paediatric urgent care training for GPs

Paediatric training is not compulsory for GPs and expertise can vary across GPs and primary care staff A knock-on effect is that newly-qualified GPs may be trained by GPs who have limited or no formal paediatric qualifications. Health professionals based in hospitals perceived that some children were referred to ED because GPs lacked the experience or confidence to provide care in the community. GPs who had undertaken additional paediatric training acknowledged they had foregone training in other areas, such as care of the elderly or mental health.

Parental perspectives focused on comparing the reassurance and expertise they felt when encountering staff in dedicated paediatric settings and on the barriers that some encountered in gaining this in the community.

GPs with experience and skills in acute paediatrics may be able to improve access, triage and appropriate referral of acutely unwell children in the community. Additional training for GPs may be feasible within

For peer review only - http://bmjopen.bmj.com/site/about/guidelines.xhtml

the current UK three-year GP training prior to certification to practice. In the longer term this may improve urgent care pathways and reduce attendances at ED and/or SSAs.

Create rotational posts between primary and secondary care settings

Health professionals indicate that staff rotating between primary and secondary care can build capacity, capability, knowledge, experience of illness trajectories and relationships. Hospital staff gain greater awareness of issues such as the time pressures of 10-minute appointment schedules in general practice, and the concerns when a hospital paediatric service is hours away. Skills development is important, for example, ANPPs from hospital working in primary care can support development of acute paediatric assessment skills amongst primary care staff.

Some health professionals identified gaps in the primary care workforce as a key issue for timely access to appropriate care, for example where unfilled GP shifts in OOH has led to increased ED workload. Whilst rotating staff between primary and secondary care may improve urgent care pathway outcomes, in the current staff resource context the feasibility of this is uncertain.

Assessment and observation over time in community settings

A key outcome desired by parents and health professionals is to reduce uncertainty about the illness trajectory; this involves having time, space, and staff to effectively assess and observe an unwell child [11]. However, hospital health professionals noted the lack of appropriate spaces for observation and availability of appropriately skilled staff to perform it in the community.

Health professionals reported a large proportion of SSAs to hospital were the result of children requiring an extended period of assessment and observation to inform clinical decision making and safe discharge of the child home: e.g. responses to a fluid challenge in the vomiting child, or to antipyretics in the febrile child.

Parents prioritised timely assessment of their acutely unwell child by a health professional, therefore, few deemed hospital attendances to be inconvenient. PPI views were largely consistent; however, parents did consider general ED departments, where both children and adults are waiting to be seen, as an inappropriate setting for children. The challenges, as reiterated by health professionals for providing child-friendly community settings timely assessment and extended observation are: community paediatric skills gapsand the current lack of infrastructure, equipment, and suitable places, and the current NHS staff shortages.

For peer review only - http://bmjopen.bmj.com/site/about/guidelines.xhtml

The key overarching outcome of importance to both health professionals and families is safety of the child; parents value easy, direct access to urgent care for expert assessment and treatment [11]. Therefore, if community services were able to provide easy-access to staff with paediatric expertise and resources for timely assessment and observation, parents may be more likely to use community services than ED, with the potential to reduce SSAs to hospital.

Creation of holistic children's 'hubs'

PPI indicated that parents have difficulties taking their child to the GP due to busy services and parental work. Parents in low paid or unstable work lose money taking time off to attend appointments and instead attend pharmacies and ED.

Health professionals see holistic children's 'hubs' as an alternative to increasing paediatric services within existing primary care systems, although significantly more resources and personnel would be required. They described potential hubs as providing a 'one stop shop' for children's health and psychosocial needs, such as health education, routine appointments, community paediatric appointments, social work advice, and mental health services. Parents called for more tailored holistic care for both children and parents. Hubs could help by shifting more resources to community healthcare and integrating health visiting.

Some described hubs as combining the interventions discussed above: i.e. staffed by professionals skilled in paediatrics who could observe and assess children. Children's hubs could be an accessible location, target disadvantaged communities and may improve outcomes and/or impact on GP and hospital workload, although in remote and rural areas there may still be considerable travel distance.

Hospital at Home model

Parent and toddler group PPI strongly indicated that families face multiple financial and logistical barriers to attending healthcare services including: no car, the high cost of public transport and taxis; no 24-hour public transport; parking challenges; time availability; and lack of childcare for other children.

Health professionals suggested an intervention which would provide more support and medical care for families in their own home, through a 'hospital at home' model. They cited hospital admissions for non-medical reasons, for example parental concern, or lack of capacity in the current system to assess children or follow-up at home.

The experiences of some parents support the model, e.g. a child with croup and parents' desire for easy and/or direct access to urgent care from paediatric specialists (depending on the intervention design and

For peer review only - http://bmjopen.bmj.com/site/about/guidelines.xhtml

delivery). There is some complementarity and potential to combine interventions :i increasing primary-care staff paediatric skills, assessment and observation in the community, community hubs, and hospital at home to address the outcomes of importance to parents and professionals.[10}

Extend specialised care pathways for sub-groups of children

Some parents described specialist care pathways for their children with acute or chronic relapsing conditions. Consultants, nurses and GPs from spoke of the benefit of existing specialist care pathways for a minority of conditions, e.g. epilepsy and diabetes. A primary aim of these pathways is to equip and support parents to manage children at home, and thus avoid unnecessary trips to hospital. Interviewees proposed extending specialist care pathways to other acute conditions and/or age groups as a way of triaging to appropriate expertise.

Many health professionals viewed babies with fever, and young children with respiratory illnesses as primary targets for future interventions which may improve their outcomes and impact on attendances in primary and secondary care. Convulsions were frequently raised and are a common reason for SSA explored in depth elsewhere (Malcolm et al, 2023).

Phase 2 Results of Stakeholder Intervention Prioritisation

The stakeholder event had 47 attendees (22 attending online): 7 parents, 12 health professionals (primary and secondary care nurses, consultants, nurse consultants and GPs), and 28 representatives from stakeholder organisations (charities and parent groups' co-ordinators, academics, and government).

Increase community specialist acute paediatric nursing roles for care of the acutely unwell child

Stakeholders affirmed the importance of ANPPs with expertise in care of the acutely unwell child, especially in areas where patients live far from a hospital. A skilled ANPP in the GP practice could potentially send parents home with a care plan and prevent SSAs and/or reduce pressure on OOH services.

For peer review only - http://bmjopen.bmj.com/site/about/guidelines.xhtml

ANPPs are seen as appropriate professionals to undertake neonatal assessments and manage chronic paediatric conditions for which children would otherwise attend hospital out-patient clinics. A caveat expressed by stakeholders (but not interviewees), was the importance of ANPPs being rooted in secondary care, whether through rotation or regular shifts, to keep up to date and avoid losing their skills in dealing with acutely unwell children. More integrated and fluid professional roles across primary-secondary care services, rather than the current dichotomy of community versus hospital were envisioned.

Stakeholders identified several challenges for increasing community ANPP roles: to fully train an ANPP can take around five years, so increasing numbers needs advance planning; some hospital ANPPs do not see children under the ages of 5 or 12 years, and thus require more training to become confident in managing younger children; many community ANP roles are advertised for adult care only. Some hospital health professionals lamented the potential loss of their skilled ANPPs to primary care. Stakeholders with paediatric nursing experience suggested extending roles in primary care could de-skill other GP and nursing staff through reducing contact with unwell children.

Improve paediatric skills by creating rotational posts between primary and secondary care settings

Stakeholders recognised the value of observing acutely unwell children in community settings but acknowledged that it would require confidence-building in both staff and parents. Increasing paediatric skills in the community for GPs and nurses was acknowledged to be dependent on population geography, existing service structure and staff resource, with only one health professional specifically mentioning rotational posts for nurses. Others mentioned the benefits of less formal cross-overs between care settings, e.g. skills gained from previous roles, GPs training in secondary care, or hospital clinicians holding occasional clinics in primary care. A more integrated/fluid rotation of staff across primary and secondary care would provide a ‘safety net’ when creating a service that relies on a few highly specialised staff.

Creation of holistic children’s ‘hubs’

Similar to interviewees, stakeholders suggested potential hubs as providing holistic paediatric services include a focus on preventing and managing childhood illnesses; Rs and situated in the community with good transport links and parking. Hubs could provide care for extended hours, bridging the current gap

For peer review only - http://bmjopen.bmj.com/site/about/guidelines.xhtml

between general practice opening hours (8am-6pm) and the busiest time in urgent care (6pm -2am). A few health professionals commented that observation and assessment in hubs may particularly suit children where National Institute of Health and Care Excellence (NICE) pathways apply, e.g., bronchiolitis.

Unlike interviewees, stakeholders raised several concerns about potential hubs: they may become too busy; parents may become over-reliant rather than learning how to manage childhood illnesses at home; information and advice currently provided by the 24 hour NHS telephone advice and triage service (NHS24) could be replaced by the hubs, which may result in unforeseen issues such as a lack of access to the large bank of language interpreters. A substantial cultural shift in parental confidence with primary care and community services will be required, otherwise parents may simply bypass the hubs and go straight to ED.

Stakeholders perceived the massive investment and political change required for hubs as unrealistic, given previous promises of more investment in community health services have not translated into practice. Furthermore, they highlighted that interventions such as hubs would only be viable with adequate staffing which could be a barrier with the current workforce shortage.

Hospital at Home model

Stakeholders discussed the hospital at home model only briefly. Some stakeholders raised concerns about who would staff a hospital at home model because this might deplete staff in existing services, putting even more strain on them. Others felt that hospital at home might be a way of retaining staff, e.g., nurses, who no longer want to work on acute wards. In support of the model, some parents stated that, with hindsight, the help and reassurance they had received from SSAs could have been provided in the community and the model could potentially provide the continuity of care and familiar staff that parents want.

Extend specialised care pathways

Stakeholders spent less time discussing specialist care pathways for bronchiolitis, convulsions, and infants aged under 2 years. Some parents had experience of specialist diabetic and epilepsy nurses and praised the care their children had received. Health professionals preferred condition-based, rather than age-based, pathways. They felt that specialist nursing was appropriate for relatively uncommon conditions

For peer review only - http://bmjopen.bmj.com/site/about/guidelines.xhtml

but was not suitable for frequently occurring conditions and those with existing standardised NICE pathways, such as bronchiolitis. Even where NICE care pathways do exist, health professionals lamented that adherence is variable and education is needed. They recognised that if a child arrives with multiple issues, it often leads to health professionals' confusion over which pathway to follow.

Other specialised pathways were discussed, example e.g. a paediatric-specific NHS24 phoneline, but it was concluded this might be confusing for parents to have yet another telephone number, and that staffing it would be challenging. Stakeholders wanted greater recognition of direct referral to pre-hospital specialists, such as opticians (NHS24 already refer children straight to opticians when appropriate) and pharmacists, who could advise parents around common childhood illnesses.

Results of ranking the priority of interventions

Twenty attendees at the interactive stakeholder event (16 in person and four online) submitted their anonymised priority rankings for the interventions they considered most important with a score of 1 indicating the most popular option and 7 the least popular option. Table 3 shows a cluster of three more popular options (ranks 1-3) and options that were not so popular (ranks 4-7). Stakeholders considered creating specialist care pathways for bronchiolitis, convulsions, and infants aged under 2 years as lower priority interventions, with the lowest priority pathway being one for infants.

**Potential intervention**	**Mean score**	**Rank**
Addressing gaps in acute paediatric skills of health professionals working in community settings	2.53	1
Creation of holistic children's 'hubs'	2.73	2
Assessment and observation of acutely unwell children in community settings	3.00	3

For peer review only - http://bmjopen.bmj.com/site/about/guidelines.xhtml

Hospital at home model.	4.47	4
Specialised care pathways - for wheeze /bronchiolitis.	4.76	5
Specialised care pathways - for convulsions.	4.94	6
Specialised care pathways - for under twos.	5.33	7

Table 3. Results of stakeholder intervention prioritisation ranking

For peer review only

DISCUSSION

In a robust mixed-methods study combining analysis of quantitative and qualitative data, PPI engagement and systematic review evidence [7], we were able to identify and prioritise interventions with potential to improve urgent care pathways for children. Of seven potential interventions, stakeholders including parents and health professionals identified three higher priority and four lower priority future interventions. Higher priority interventions were to address gaps in the acute paediatric skills of health professionals working in community settings, to create holistic children's hubs, and to facilitate assessment and observation of acutely ill children in community settings. Lower priority interventions were "hospital at home" and specialised care pathways for convulsions, infants under two years old, and wheeze/bronchiolitis. The higher priority interventions could be combined into a whole population intervention which provides community-based assessment and observation by appropriately skilled health professionals in an accessible location/hub.

Strengths include using the INDEX guidance [9] for intervention development which has not been followed previously to address the gap in the systematic evidence [7] about how to improve pre-hospital acute paediatric care and combining qualitative and quantitative methods with stakeholder engagement and PPI. Different data collection, settings and framing of approach generate different perspectives and help in the search for disconfirming data. The health professional contributions are representative of different professional groups (apart from paramedics who we tried unsuccessfully to recruit) and represented a range of urban and rural /remote areas.

For peer review only - http://bmjopen.bmj.com/site/about/guidelines.xhtml

Limitations include that parents talked about their care experiences and preferences, but did not offer specific solutions. Due to COVID19, we could not recruit parents and children face to face at hospitals as intended which limited sample diversity. Children's views are not represented, as many were infants and no-one took up the invitation to a video- conference or telephone interview with their child. Solutions for cities might look different to those for remote and rural communities which may have been under-represented at the stakeholder event. Rural and remote areas have distance, travel and different workload/ resource considerations. Generalisability of findings beyond Scotland and to the post-COVID years is uncertain, however many staff have cross-UK and international experience. The priority ranking exercise was exploratory and only 20 out of 47 attendees completed it.

Our study is unique in its inclusion of health professional perspectives and triangulation of different stakeholder perspectives to inform paediatric care intervention development. Previous published qualitative research has focused on parents' perspectives of admissions [25, 26]. Prioritising what matters to patients and health professional (the target population) is important when designing an intervention with a congruent theory of change and will inform future decisions about the focus for future evaluation design [27]. Our findings complement a recent rapid literature review of (not paediatric-specific) patient urgent and emergency care experiences [28]. Another recent review of the literature was published after our study was completed [29]. Although this did not inform our findings it also calls for prompt introduction and evaluation of improved pre-hospital pathways for the care of acutely unwell children.

The importance of rotation of staff and the role of ANPPs in integrated services to facilitate early referral to the Community Children's Nursing Teams as an alternative to hospital care; strong personal relationships between consultants and community children's nurses are considered important [30]. In England, what reassures professionals, especially GPs, when referring to Community Children's Nurses are clear clinical governance protocols [31]. There is some evidence to indicate that an experienced GP with paediatric training may be the best person to decide whether a child is ill or not [32]. Research in Scotland revealed that the quality of relationships, communication, and expectations between GPs and hospital specialists at the interface between primary and secondary care were important influences on patient care [33].

Evidence of the best short- and long-term outcomes for children and families is required before novel pathways are implemented into routine acute care pathways. Further intervention acceptability,

For peer review only - http://bmjopen.bmj.com/site/about/guidelines.xhtml

feasibility/pilot testing research is required using randomised controlled trial methodology to establish effectiveness, cost-effectiveness and any unintended consequences over short to longer term horizons.

The key message for policy-makers is that to improve paediatric urgent care pathways stakeholders, including health professionals and families, want future interventions that are safe, patient-centred and community-based [11]. Close collaboration between academics and policy makers, senior decision makers and potential funders will be required to ensure that future interventions value contributions from parents and front-line health professionals and are rigorously evaluated to advance evidence based policy and care that improves child health outcomes.

FUNDING STATEMENT

This work was supported by the Chief Scientist Office of the Scottish Government, grant number HIPS/18/09.

COMPETING INTERESTS STATEMENT

No competing interests to declare.

AUTHOR CONTRIBUTIONS

ST, RK, EF, CM, LA, PW and PH conceived the idea. SD, LA and ST undertook the quantitative research. CM, EK, EF and PH undertook the qualitative research. SK undertook patient and public involvement engagement and co-ordination. EK wrote the first draft of the manuscript. All authors made important contributions to revisions of the paper and have seen the final submitted version. ST is the guarantor of the work. Amy Woodhouse of Children in Scotland registered charity critically reviewed and commented on drafts of this manuscript. We are grateful for Dave Kelly at Albasoft for providing primary care data. We are grateful to Rebecca Fairnie at Electronic Data Research and Innovation Service for managing our access to all data.

The authors would like to extend our thanks to the parents and health professionals who participated in this study, and to the stakeholders who participated in both PPI activities and the engagement and prioritisation event.

For peer review only - http://bmjopen.bmj.com/site/about/guidelines.xhtml

REFERENCES

1. Ruzangi J, Blair M, Cecil E, Greenfield G, Bottle A, Hargreaves DS, et al. Trends in healthcare use in children aged less than 15 years: a population-based cohort study in England from 2007 to 2017. BMJ Open. 2020;10(5):e033761.
2. Al-Mahtot M, Barwise-Munro R, Wilson P, Turner S. Changing characteristics of hospital admissions but not the children admitted-a whole population study between 2000 and 2013. European Journal Of Pediatrics. 2018;177(3):381-8.
3. Barwise-Munro R, Morgan H, Turner S. Physician and Parental Decision-Making Prior to Acute Medical Paediatric Admission. Healthcare (Basel, Switzerland). 2018;6(3).
4. Williams TC, MacRae C, Swann OV, Haseeb H, Cunningham S, Davies P, et al. Indirect effects of the COVID-19 pandemic on paediatric healthcare use and severe disease: a retrospective national cohort study. Archives of Disease in Childhood. 2021;106(9):911-7.
5. Morris J, Fisher E. Growing problems, in depth: The impact of Covid-19 on health care for children and young people in England. The Nuffield Trust, ; 2022 18/02/2022.
6. Coon JT, Martin A, Abdul-Rahman AK, Boddy K, Whear R, Collinson A, et al. Interventions to reduce acute paediatric hospital admissions: a systematic review. Arch Dis Child. 2012;97(4):304-11.
7. Dick S, MacRae C, McFaul C, Rasul U, Wilson P, Turner SW. Interventions to reduce acute paediatric hospital admissions: a systematic review. Arch Dis Child. 2022;107(3):234-43.
8. Skivington K, Matthews L, Simpson SA, Craig P, Baird J, Blazeby JM, et al. Framework for the development and evaluation of complex interventions: gap analysis, workshop and consultation-informed update. 2021;25:57.
9. O'Cathain A, Croot L, Duncan E, Rousseau N, Sworn K, Turner KM, et al. Guidance on how to develop complex interventions to improve health and healthcare. BMJ Open. 2019;9(8):e029954.
10. Dick S, Kyle R, Wilson P, Aucott L, France E, King E, et al. Insights from and limitations of data linkage studies: analysis of short-stay urgent admission referral source from routinely collected Scottish data. Archives of Disease in Childhood. 2022:archdischild-2022-324171.
11. Malcolm C, King E, France E, Kyle RG, Kumar S, Dick S, et al. Short stay hospital admissions for an acutely unwell child: A qualitative study of outcomes that matter to parents and professionals. PLoS One. 2022;17(12):e0278777.
12. National Institute for Health Research. Briefing notes for researchers - public involvement in NHS, health and social care research 2021 [Available from: https://www.nihr.ac.uk/documents/briefing-notes-for-researchers-public-involvement-in-nhs-health-and-social-care-research/27371.
13. UK Standards for Public Involvement. UK Standards for Public Involvement 2016 [Available from: https://sites.google.com/nihr.ac.uk/pi-standards/home.
14. Williams TC, Bach CC, Matthiesen NB, Henriksen TB, Gagliardi L. Directed acyclic graphs: a tool for causal studies in paediatrics. Pediatr Res. 2018;84(4):487-93.
15. Textor J. DAGitty - draw and analyze causal diagrams [Available from: http://www.dagitty.net/.
16. Ritchie J, Spencer L. Qualitative data analysis for applied policy research. Analyzing qualitative data: Routledge; 2002. p. 187-208.
17. King E, Dick S, Hoddinott P, Malcolm C, France E, Kyle RG, et al. Regional variations in short stay urgent paediatric hospital admissions - a sequential mixed methods approach exploring differences through data linkage and qualitative interviews. BMJ OPEN. Under review.

For peer review only - http://bmjopen.bmj.com/site/about/guidelines.xhtml

18. Coughlan CH, Ruzangi J, Neale FK, Nezafat Maldonado B, Blair M, Bottle A, et al. Social and ethnic group differences in healthcare use by children aged 0–14 years: a population-based cohort study in England from 2007 to 2017. Archives of Disease in Childhood. 2022;107(1):32-9.
19. Rose TC, Adams NL, Whitehead M, Wickham S, O'Brien SJ, Hawker J, et al. Neighbourhood unemployment and other socio-demographic predictors of emergency hospitalisation for infectious intestinal disease in England: A longitudinal ecological study. J Infect. 2020;81(5):736-42.
20. Sartain SA, Maxwell MJ, Todd PJ, Jones KH, Bagust A, Haycox A, et al. Randomised controlled trial comparing an acute paediatric hospital at home scheme with conventional hospital care. Arch Dis Child. 2002;87(5):371-5.
21. Jones E, Taylor B, Rudge G, MacArthur C, Jyothish D, Simkiss D, et al. Hospitalisation after birth of infants: cross sectional analysis of potentially avoidable admissions across England using hospital episode statistics. BMC Pediatrics. 2018;18(1):390.
22. Scottish Home and Health Department. Towards an integrated child health service: H.M. Stationery Office. Edinburgh.; 1973.
23. Dunhill Z. Developing a Community Child Health Service for the 21st Century. The Scottish Government, ; 2013.
24. Kerr A. Building a Health Service fit for the future. NHS Scotland; 2005.
25. Jones CH, Neill S, Lakhanpaul M, Roland D, Singlehurst-Mooney H, Thompson M. Information needs of parents for acute childhood illness: determining 'what, how, where and when'of safety netting using a qualitative exploration with parents and clinicians. BMJ open. 2014;4(1):e003874.
26. Neill SJ, Jones CHD, Lakhanpaul M, Roland DT, Thompson MJ. Parents' help-seeking behaviours during acute childhood illness at home: A contribution to explanatory theory. Journal Of Child Health Care: For Professionals Working With Children In The Hospital And Community. 2016;20(1):77-86.
27. Tovey D, Blaine C. How to clarify a clinical question 2022 [Available from: https://bestpractice.bmj.com/info/toolkit/learn-ebm/how-to-clarify-a-clinical-question.
28. The Patient Experience Library. Patient experience in urgent and emergency care. 2022.
29. Dick S, MacRae C, McFaul C, Wilson P, Turner SW. Interventions in primary and community care to reduce urgent paediatric hospital admissions: systematic review. Archives of Disease in Childhood. 2023:archdischild-2022-324986.
30. Kyle RG, Banks M, Kirk S, Powell P, Callery P. Integrating community children's nursing in urgent and emergency care: a qualitative comparison of two teams in North West England. BMC pediatrics. 2012;12(1):101.
31. Kyle RG, Banks M, Kirk S, Powell P, Callery P. Avoiding inappropriate paediatric admission: facilitating General Practitioner referral to Community Children's Nursing Teams. BMC family practice. 2013;14(1):4.
32. Stephenson T. Paediatric primary care in Europe: variation between countries. Arch Dis Child. 2010;95(10):767-8.
33. Sampson R, Barbour R, Wilson P. The relationship between GPs and hospital consultants and the implications for patient care: a qualitative study. BMC Family Practice. 2016;17(1):45.

SUPPLEMENTARY FILES

Supplementary file 1. Detailed methods

Supplementary file 2. Semi-structured interview guide (health professionals)

For peer review only - http://bmjopen.bmj.com/site/about/guidelines.xhtml

Supplementary file 3. Semi-structured interview guide (parents)

Supplementary file 4. Participant information sheet- qualitative study

Supplementary file 5. Dag model

Supplementary file 5a. Revised logic model

Supplementary file 6. Materials & methods for stakeholder event

Supplementary file 6a. Stakeholder event morning slides

Supplementary file 6b. Stakeholder event afternoon slides

Supplementary file 7. Characteristics of qualitative interview participants

Supplementary file 8. Supporting interview quotations

Research Checklists:

GUIDED reporting checklist

For peer review only - http://bmjopen.bmj.com/site/about/guidelines.xhtml

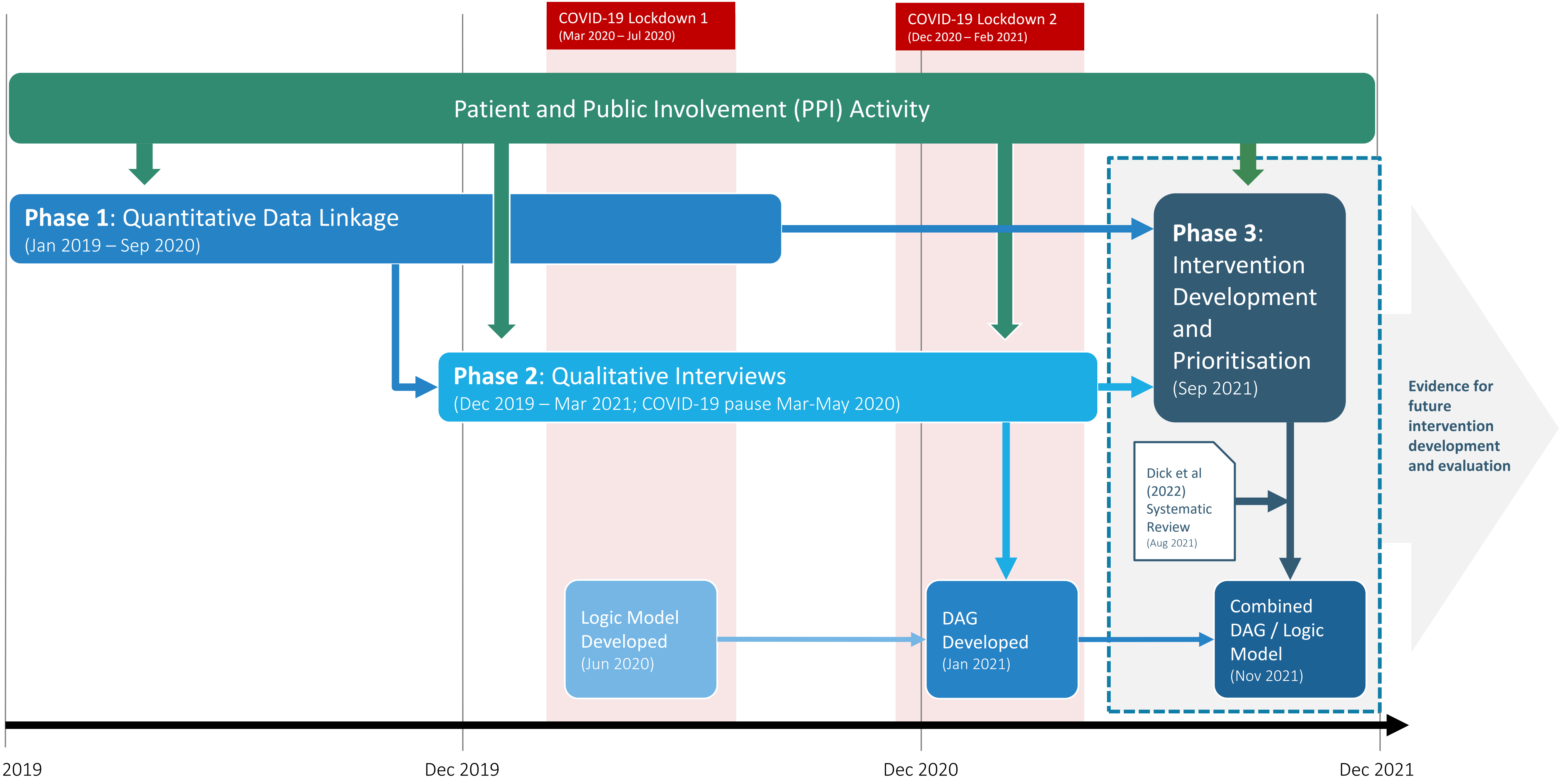
COVID-19 Lockdown 1
(Mar 2020 – Jul 2020)
COVID-19 Lockdown 2
(Dec 2020 – Feb 2021)
Patient and Public Involvement (PPI) Activity
Phase 1: Quantitative Data Linkage
(Jan 2019 – Sep 2020)
Phase 2: Qualitative Interviews
(Dec 2019 – Mar 2021; COVID-19 pause Mar-May 2020)
Phase 3: Intervention Development and Prioritisation
(Sep 2021)
Evidence for future intervention development and evaluation
Dick et al (2022) Systematic Review
(Aug 2021)
Logic Model Developed
(Jun 2020)
DAG Developed
(Jan 2021)
Combined DAG / Logic Model
(Nov 2021)
Jan 2019
Dec 2019
Dec 2020
Dec 2021

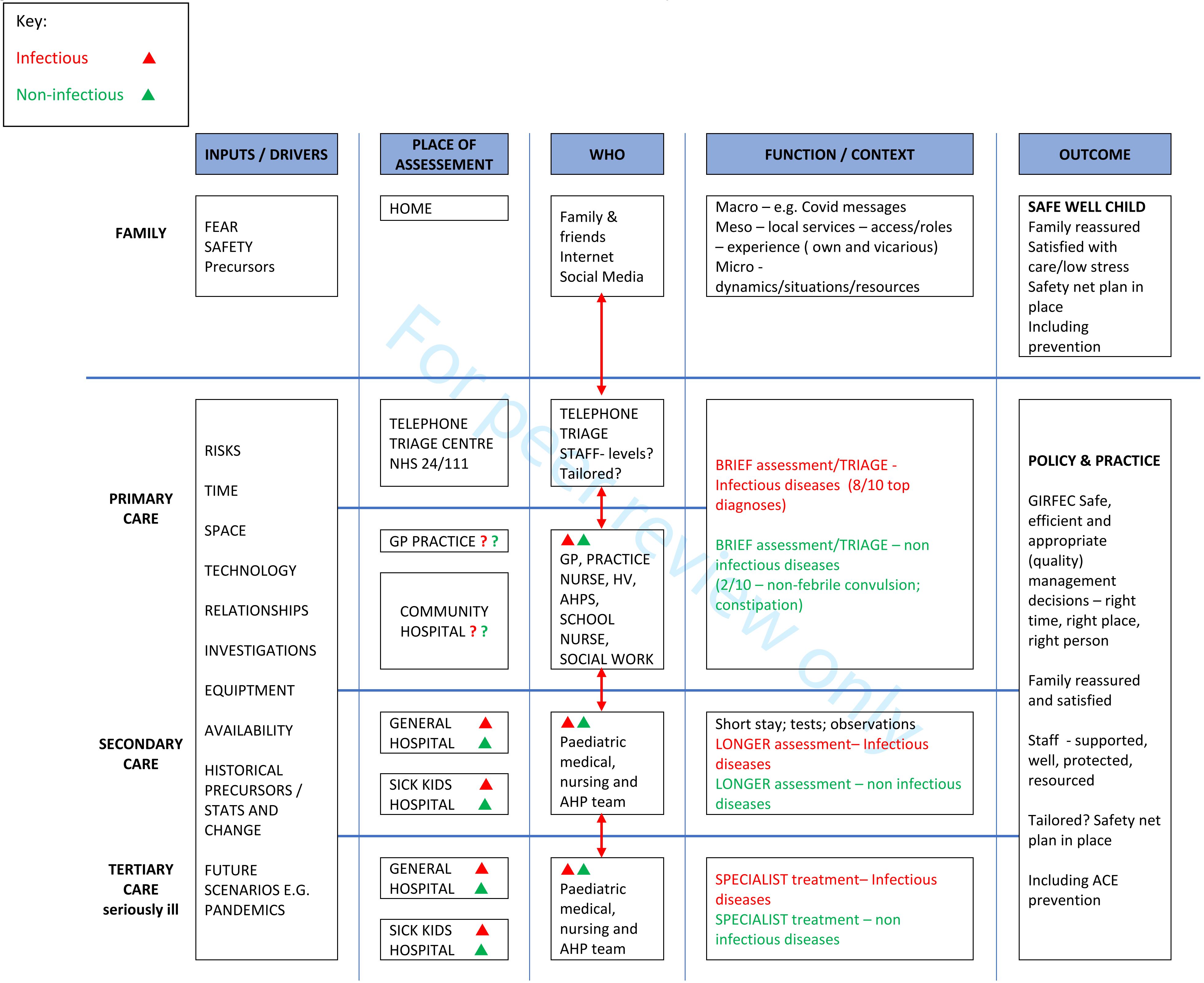

For peer review only

Supplementary file 1. FLAMINGO study detailed methods for data linkage and qualitative interviews

Study design and setting

The mixed-methods FLAMINGO study was undertaken in Scotland, where the NHS is organised into 14 geographically distinct terrestrial Health Boards, each responsible for healthcare provision to their region's population. The intervention development used a mixed methods phased approach and was target population centred, incorporating front-line health professional and patient perspectives collected through Public and Patient Involvement (PPI), stakeholders, and qualitative interviews. Patient and public involvement occurred throughout the project from inception to prioritisation of findings. Prioritising outcomes that matter to patient and staff participants (the target population) is important when designing an intervention with a congruent theory of change and will inform future decisions about the Population Intervention Comparator Outcome (PICO) for future evaluation design.[1] A programme theory of change was developed early in Phase 1 informed by the DAGIT process/model. [2] [Supplement 5] The DAGIT was referred to iteratively throughout the FLAMINGO study and incorporated into a final diagrammatic theory of change once the data analysis was complete [Supplement 5a]. The whole Flamingo team met monthly, the quantitative and qualitative sub teams met separately in between. An underpinning Systematic Review of interventions to reduce acute paediatric hospital admissions was undertaken by a FLAMINGO sub-team.[3] It was unclear at the start whether the systematic review would identify an intervention with sufficient promise to be adapted or whether developing a new intervention would be indicated. The findings were shared with the FLAMINGO team once qualitative data collection was complete and were incorporated into the intervention prioritisation process.

The study was approved by the NHS North of Scotland Research Ethics Service (REC reference: 19/NS/0134).

For peer review only

Data linkage methods

Study design

The data linkage study is reported in detail elsewhere.[4] Data from the following five clinical services were linked: Hospital admission details (Scottish Morbidity Record 01, SMR01); Out-of-Hours, Emergency Department and NHS 24 (all within the Unscheduled Care Datamart, UCD); general practice data (provided by Albasoft, an NHS trusted third party). The period of was 2015-2017 and was determined by the availability of data from UCD (beginning April 2014) and the onset of the study (2019). A referral source was defined as the location (ED, GP, OOH or combinations thereof) where the child had a clinical record for the day of admission. When there was contact with more than one referral source on the day of admission, sources were placed in chronological order where possible (time of GP contact was not provided in the majority of records). When no referral source was identified we took two additional approaches (i) contact with a referral source the day before admission was sought (ii) we determined whether the child had been discharged from hospital on the same day as admission or the day before ("open access admissions").

Setting

Two NHS Scotland terrestrial health boards (Lothian, and Greater Glasgow and Clyde) have ED facilities staffed by paediatricians but in all other health boards, staff trained in emergency medicine see children in an ED. Three health boards have very limited in-patient paediatric facilities (Orkney, Shetland, and Western Isles) and no data from these boards were included in this analysis. Four health boards have children's hospitals (Greater Glasgow and Clyde, Grampian, Lothian, and Tayside).

Participants

Individuals <16 years with ≥ one urgent admission to hospital under the care of a medical paediatric team in Scotland in the calendar years 2015-2017. Children seen in and discharged from the ED or admitted under paediatric surgical, orthopaedic or dental teams were ineligible.

Ethical approval

The study was approved by the Public Benefit and Privacy Panel for Health and Social Care (reference 1718-0183). Data were analysed in the National Data Safe Haven of Scotland,[5] a secure repository accessed through a virtual private network by trained researchers.

For peer review only

Data sources

Scottish Morbidity Record 01, SMR01.[6] The SMR01 data are assessed for quality and completeness and are estimated to be 99% complete as of March 2018.[7] Data provided were: sex; ethnic group; decimal age; date of admission; date of discharge; admission type (emergency or elective); Scottish Index of Multiple Deprivations (SIMD) quintile; up to six ICD-10 diagnoses; Health Board of admission; specialty (paediatric medicine, paediatric surgery or paediatric dentistry). Time of admission was not available.

Unscheduled Care Datamart (ED, OOH and NHS24 data). This resource provides data from ED, OOH and NHS 24 (the latter including the Scottish Ambulance Service). All records have a valid CHI number.[8]

GP Data (Albasoft). GP data were made available through NHS Trusted Third Party (Albasoft) using a previously described method.[9] Briefly all Scottish general practices have software potentially accessible by Albasoft and practices can opt in and consent to share data with researchers via Albasoft. Typically, 15-20% of practices agree to provide data.[9] Where records were available, Read codes were used to distinguish clinical contact leading to a referral from administrative entries arising from clinical contact (e.g. a letter from ED after presenting there).

Bias

Completeness of data for SMR01 and the UCD ensures no bias. GP data were only available after practices opted in and this may introduce bias.

Study size

The study size was all individuals aged <16 years with an urgent admission to hospitals in Scotland in the calendar years 2015-2017.

Statistics

Descriptive statistics are provided for comparisons between groups. Multivariate logistic models were used to compare odds ratio (OR) for the ten most common composite diagnoses stratified by referral source. Composite diagnosis was defined as a group of very similar conditions, e.g. “asthma” included International Classification of Disease (ICD)-10 code J45.0 (predominantly allergic asthma), J45.9 (other and unspecified asthma) and J46X (Status Asthmaticus).[10] The benefit of using composite diagnoses is that they minimise the potential for variations in coding between units.[11] The following accounted for 46.4% of all urgent admissions in Scotland 2000-2013: asthma, bronchiolitis, convulsion

For peer review only

(including febrile and afebrile convulsions), croup, gastroenteritis, upper respiratory tract infection (URTI), viral infection, tonsillitis, lower respiratory tract infections (LRTI) and admissions with a diagnosis of cough or wheeze or shortness of breath.[10] A stringent p value of <0.001 was used to indicate a meaningful association since the sample size was so large. Standard statistical software was used for the analysis (IBM® SPSS® version 24).

FLAMINGO qualitative interview methods

A qualitative exploratory approach was used to gain a better understanding of the experiences of parents and health professionals with regards to the circumstances around unscheduled SSAs of children (up to the age of 16) to hospital.

Qualitative setting

The FLAMINGO project team purposively identified five Health Boards to act as cases that would allow for maximal variation in characteristics such as deprivation, urban-rural, and hospital structure (with or without a dedicated children's hospital or short stay facility). Case selection was also informed by the quantitative data emerging from the data linkage exercise to allow for variation in numbers of unscheduled hospital admissions.

Participants and sampling

Health professionals were recruited by the team from a paediatric medical conference in Edinburgh in 2019, using their professional networks, and snowball sampling. Purposive sampling was used to invite health professionals working in primary care, GP out of hours services (OOH), and hospital emergency departments (ED)/ paediatric ED (PEDs) workplaces, NHS24, and the Scottish Ambulance service in the five case Health Boards, with responsibility for referring or receiving children for acute paediatric medical admission. Recruitment was unsuccessful from NHS24 and the Scottish Ambulance service.

Initial Patient and Public Involvement discussions revealed that many parents were unsure at what point in a hospital visit their child was officially categorised being admitted. The quantitative analysis of the data linkage analysed SSA where admission and discharge occur on the same calendar date.[4] To improve clarity when recruiting parents for interview, the definition of a SSA was therefore adapted to include stays in hospital lasting less than 24 hours. Parents were eligible to participate if they had

For peer review only

experience of a SSA for their child with an acute medical illness and taken place within the past five years. SSAs for surgical or dental conditions were excluded.

The COVID-19 pandemic coincided with our qualitative recruitment phase and associated restrictions such as 'lockdowns' meant that previously planned in person recruitment methods at hospitals and places frequented by parents could not be undertaken. Alternative convenience sampling methods were adopted including online platforms and social media, press releases and advertisements in local newspapers. Parents responding to the invitation were sent a participant information sheet, study details and guidance on how to contact the main researcher (EK) to arrange a telephone interview. Due to the challenges of recruiting to health research during the pandemic,[12] and the project's objective of ensuring maximal variation of participants, the recruitment press release in January 2021 invited eligible participants from outside the five Health Board case areas.

In addition, Phase 1 quantitative analysis and early interviews with families raised seizures as a particularly interesting reason for a SSA. Further purposive sampling and recruitment of health professionals and families with experience of seizure and findings will be reported separately.

Data collection

Interviews were conducted by two experienced qualitative researchers (EK, CM) with different backgrounds (health services research and paediatric nursing) and reflective field notes were kept. Semi-structured interviews were chosen for consistency between the two interviewers (EK, CM) whilst allowing interviewees to spontaneously raise issues. Separate semi-structured interview topic guides were developed for parents and health professional interviews by our multidisciplinary research team, with patient and public involvement (PPI) and drawing on existing literature on urgent short stay hospital admissions [the interview topic guides are in Supplementary files 2 and 3]. The topic guides included a specific question about recommendations for change regarding urgent SSAs. Health professionals were asked to propose potential solutions to improve urgent care pathways for children between home and hospital admission. Parents were asked questions relating to how their family's experiences could be improved in future.

The interviews took place between December 2019 and March 2021, pausing due to the COVID-19 lockdown between March and May 2020. Telephone interviews replaced face-to-face interviews. The COVID-19 pandemic was a context disruptor and data collected during this period might not be fully

For peer review only - http://bmjopen.bmj.com/site/about/guidelines.xhtml

representative of typical SSA practices; therefore, interviewees were asked to speak about experiences pre-COVID. Interviewees were then asked specifically if and how they had observed differences during, or as a consequence of, the pandemic.

The duration of the interviews ranged from 18 to 62 minutes (median 27) and each interview was audio recorded, transcribed verbatim and anonymised.

Data analysis

Anonymised transcripts were uploaded to QSR NVivo 12 data management software (QSR International Pty Ltd., Version 12, 2019, Victoria, Australia) and were analysed using the Framework Method, used widely across health services research, which offers a systematic and flexible model for supporting qualitative thematic analysis.[13] It is particularly useful in situations where there is a desire to identify themes through making comparisons both within and between cases.[13] This was the aim of the current research which set out to explore the experiences of SSAs from the perspective of both parents and health professionals. The framework approach involves a five-stage iterative process of 1) data familiarisation; 2) identification of a thematic coding framework; 3) indexing and further refinement of the coding framework; 4) charting; and 5) mapping and interpretation to search for patterns and explanations in the data. During the initial data familiarisation stage, members of the FLAMINGO qualitative team (EK, CM, EF, PH) independently read a sample of four transcripts and field notes, two from each participant group (parents and health professionals), in depth and a high-level coding framework was devised by each member. The group met on several occasions to discuss overlaps and differences between the four coding indexes which were merged to form the first version of the coding framework. This framework, and the main thematic areas to focus on in the analysis, was then shared with the wider FLAMINGO team for additional comments and input. A subset of six transcripts were independently coded by EK and CM and the results compared to reveal considerable reliability in coding. Subsequently, minor changes to the codes were made, in order to further improve consistency.

The third indexing stage involved systematically applying the analytical coding framework to the remaining transcripts in NVivo. Line by line coding was carried out by three researchers (EK, CM, EF), with ongoing discussion and debate at weekly meetings. A framework matrix was developed to allow for organisation of the data samples (parents and health professionals) by themes to facilitate cross-case and within-sample comparisons. The FLAMINGO qualitative team reviewed the final themes to

For peer review only

reach consensus in the mapping and interpretation of the data, thus enhancing rigour and trustworthiness.

Analysis of interview data relating to the outcomes and values that matter to parents and professionals when designing an intervention to improve care for children with SSAs is reported in Malcolm et al.[14]

References

1. Tovey D, Blaine C. How to clarify a clinical question 2022 [Available from: https://bestpractice.bmj.com/info/toolkit/learn-ebm/how-to-clarify-a-clinical-question.
2. Williams TC, Bach CC, Matthiesen NB, Henriksen TB, Gagliardi L. Directed acyclic graphs: a tool for causal studies in paediatrics. Pediatr Res. 2018;84(4):487-93.
3. Dick S, MacRae C, McFaul C, Rasul U, Wilson P, Turner SW. Interventions to reduce acute paediatric hospital admissions: a systematic review. Arch Dis Child. 2022;107(3):234-43.
4. Dick S, Kyle R, Wilson P, Aucott L, France E, King E, et al. Insights from and limitations of data linkage studies: analysis of short-stay urgent admission referral source from routinely collected Scottish data. Archives of Disease in Childhood. 2022:archdischild-2022-324171.
5. ISD Scotland. National Safe Haven [Available from: https://www.isdscotland.org/Products-and-Services/eDRIS/Use-of-the-National-Safe-Haven/.
6. ISD Scotland. General Acute Inpatient and Day Case - Scottish Morbidity Record (SMR01) [Available from: https://www.ndc.scot.nhs.uk/National-Datasets/data.asp?SubID=5.
7. ISD SG. Data Quality and Completeness for the Annual Acute Hospital Activity and NHS Beds Activity Publication 2018 [Available from: https://www.isdscotland.org/Health-Topics/Hospital-Care/Publications/2018-09-25/Data-Issues-and-Completeness-Sep18.pdf.
8. ISD Scotland. Unscheduled Care Datamart (UCD) [Available from: https://www.ndc.scot.nhs.uk/National-Datasets/data.asp?SubID=111.
9. Ellis DA, McQueenie R, McConnachie A, Wilson P, Williamson AE. Demographic and practice factors predicting repeated non-attendance in primary care: a national retrospective cohort analysis. Lancet Public Health. 2017;2(12):e551-e9.
10. Al-Mahtot M, Barwise-Munro R, Wilson P, Turner S. Changing characteristics of hospital admissions but not the children admitted-a whole population study between 2000 and 2013. European Journal Of Pediatrics. 2018;177(3):381-8.
11. Burns EM, Rigby E, Mamidanna R, Bottle A, Aylin P, Ziprin P, et al. Systematic review of discharge coding accuracy. J Public Health (Oxf). 2012;34(1):138-48.
12. Singh JA, Bandewar SV, Bukusi EA. The impact of the COVID-19 pandemic response on other health research. Bull World Health Organ. 2020;98(9):625-31.
13. Gale NK, Heath G, Cameron E, Rashid S, Redwood S. Using the framework method for the analysis of qualitative data in multi-disciplinary health research. BMC Medical Research Methodology. 2013;13(1):117.
14. Malcolm C, King E, France E, Kyle RG, Kumar S, Dick S, et al. Short stay hospital admissions for an acutely unwell child: A qualitative study of outcomes that matter to parents and professionals. PLoS One. 2022;17(12):e0278777.

For peer review only

The FLAMINGO project (**FL**ow of **A**d**M**issions in ch**I**ldren and you**NG** pe**O**ple)

Health Professional Interview Topic Guide

Can you start by confirming what your role is and your employer?
Probes – confirm role, employer, how long they have been in this role.

Experience of short stay admissions (SSAs)

We are interested in unplanned or emergency hospital admissions in children, particularly short stay admissions (SSA) where a child is admitted and then discharged from hospital on the same day (less than 24 hours).

Can you tell me about your experiences of SSAs of children?
Probes: frequency; typical reasons for them; referral sources – self, GP, NHS 111 etc; conditions, different decision makers (e.g. junior or senior paediatrician, A+E doctor, nurse practitioner) any other care pathways that we haven't talked about.

HOSPITAL STAFF ONLY. Please tell me about your experiences of receiving referrals for/admitting a child where you suspected they did not need to be admitted to hospital. (Probe – can you give me a specific example…)

COMMUNITY STAFF ONLY Can you think of any examples of where a child was admitted to hospital where you thought it was inappropriate/unnecessary?

Do you think that any hospital admissions of less than 24 hours could be safely avoided? (Probe – how?)

What involvement does the child and family have in the referral and admission decision-making process?

What role do the child and family's circumstances have in the referral and admission decision-making process, if any?

What role do other staff in the hospital (probe - nurses, managers) or in the community (e.g. information from the GP about the family) play. Can you think of any examples?

Some people we have talked to refer to appropriate and inappropriate admissions. What are your thoughts about these terms….? What do you consider to be an appropriate referral of an unwell child to a hospital for a short stay? Tell me about some of your experiences.

Readmissions

Can you think of occasions where a child was admitted to hospital for less than 24 hours and then required re-admission within a week or so for the same episode?

For peer review only

Tell me about your experiences of readmission (Probes: something that happens frequently/infrequently; when these types of readmissions do occur, what do you think are the reasons; factors which contribute to this; typical presentation – child/family)

Communication processes between the professional referring and the professional admitting child to hospital

Can you think of an example where communication, between the professional referring and the professional admitting child to hospital, went really well?

- What helped?

Can you think of an example where communication, between the professional referring and the professional admitting child to hospital, did not run so smoothly?

- What were the issues, what were the consequences, how could this be improved?

Question for Referrers only

Please tell me about your experiences of referring a child where you were uncertain if they needed to be admitted, to hospital.

- How do families that you know feel about referral to hospital and short admissions of < 24 hours
- What factors trigger a decision for you to refer a child [when you suspect a SSA might be the outcome]? Probe: for example, the family/parental circumstances

Thinking about the range of different children and families who access our emergency care service:

- Do family social issues have any impact on decisions (give examples)
- Do language issues (such as when English is not the first language) have any impact on decisions to admit children?
- Does the distance to hospital/transport issues/rurality have any impact on decisions to admit children?
- What are your reflections on health inequalities or deprivation and the potential impact on children's admissions, particularly SSAs?

Current situation – COVID-19 pandemic

- With the current situation with COVID-19 are you seeing a change in children attending hospital for unplanned visits? [Probe – which conditions etc]
- How do you think care of sick children at home is changing as a result of Covid-19? (prompts… are there any particular examples you have come across? Tell me more…. Any different examples…?)
- Are there any changes that you think will or should remain after the pandemic?

Final questions- looking forward

Thinking about moving forward and SSAs in children….

- What would be your recommendations for change regarding admissions of less than 24 hours?

For peer review only

- Are there any particular issues or health conditions you would highlight for the next stages of our research?
- What do you think will be happening with children's SSAs in five years' time?
- Is there someone you work with/a clinician you know of who has views very different to yours with respect to admitting children to hospital. Who do suggest we should be speaking to gain different perspectives on SSAs?

For peer review only

The FLAMINGO project (**FL**ow of **A**d**M**issions in ch**I**ldren and you**NG** pe**O**ple)

Family Interview Topic Guide

We are particularly interested in children who are unwell who spend a short time, less than 24 hours, in hospital. We are interested in infections and children who are unwell, so this research is not about accidents and injuries or operations.

Past medical history

- Brief history of child's health and past hospital admissions (purpose to determine the frequency of short stay admissions experienced and identify the admission that the family choose to talk about)

Explore the events and experiences leading up to the child's most recent or most memorable short stay hospital admission, asking families to tell their story from the start…

- What was worrying you about your child (ie. same as symptoms experienced in past? different? worrying?)
- What reasons do you recall for seeking advice?
- Who did you seek advice from? (ie. services and professionals such GP, pharmacist, NHS 24 111, internet, minor injuries unit, A&E; family; friends; other)
- Can you tell me what you understand by the service provided by: Emergency Departments [probe A&E vs minor injuries], NHS 111 / NHS 24?
- What happened next, what were the outcome/s of these initial consultations?
- What other challenges do you face as a family when your child is sick? [Probe: No money in phone, no access to technology, transport, other childcare.]

Explore the family's experience of a zero-day hospital admission,

- Can you tell me what happened from when you walked through the door of the hospital?
- Who did you see first, what happened next, can you recall any feelings experienced?
- Who made the decision that your child would be admitted to hospital? What involvement did you and your family have in any decision making related to the admission?
- What thoughts and feelings did the family have about the decision to admit your child to hospital?
- How often did you move to a different area in the hospital?
- How confident were you in the care team who looked after your child? How consistent was the information that you received from different people?
- Would anything have made a difference to your experience?

Explore the family's experiences of going home after the admission…

- Can you tell me about how the decision was made to discharge your child from hospital and send them home? What was your involvement in that decision making?

For peer review only

- What happened once you were back at home? Did any questions or concerns arise the first few days after discharge from hospital? Was there anything you felt unsure about or wanted further advice on?
- Was your child readmitted for the same illness within two weeks of being sent home? If so how did you feel about this?
- How did your experience change what you would do if a similar situation occurred again?

Explore how the family's experiences could be improved in future…

- How satisfied were you with the process of accessing services for your child?
- Tell me about communication with professionals involved in your child's care?
- What (if anything) would they have liked to be different about your story?
- What other stories have you heard from family and friends about short-stay admissions?
- Some people we have spoken to think some short-stay admissions are unnecessary… what are your thoughts about this?
- What are your views about telephone and face to face consultations for sick children?
- Could technology make a difference – e.g. video call to a children's specialist doctor?

Reflect on current situation – COVID-19 pandemic

- How has the current situation with COVID-19 changed how you would access healthcare for your child?
- How do you feel about monitoring and caring for your child at home?
- How do you think this will impact on how you access healthcare in the future?

Closing the interview…

Thank the parents for sharing their experiences.

Explain how they can get in touch with the research team if they have any thoughts or concerns after the interview.

Explain how they will find out about the results of the research.

For peer review only

FLAMINGO

FLow of AdMissions to hospital in chIldren and younG peOple

Experience of unwell children needing a short stay in hospital (less than 24hrs)? We would like to talk with you.

We would like to invite you to take part in a research project. The project is led by Professor Steve Turner of the University of Aberdeen in partnership with the University of Stirling and Edinburgh Napier University. This part of the research is being carried out by the Nursing Midwifery and Allied Health professions (NMAHP) Research Unit at the University of Stirling.

Before you decide, it is important for you to understand why the research is being done and what it will involve. Please take time to read the following information carefully and to discuss it with others if you wish. Ask us if there is anything that is not clear or if you would like more information. Take time to decide whether you wish to take part. Thank you for reading this.

What is the purpose of the research?

The aim of this project is to understand why children are admitted to hospital for stays of less than 24 hours. To answer this question we are collecting lots of data about hospital admissions and speaking to doctors and nurses. We are also talking to parents about their experience of having a child being admitted to hospital.

Who can take part?

Parents or carers whose child has ever been admitted to hospital for a stay of less than 24 hours for an illness such as a chest infection or fever and not for a broken bone.

Do I have to take part?

No. It is up to you to decide whether you want to take part or not. If you do decide to take part, you will be asked to sign a consent form. You will still be free to stop taking part at any time and without giving a reason. If you stop taking part, or decide not to take part, this will not affect the standard of health care you and your child receive.

What happens if I take part?

You will take part in a short (about 20-45 minutes) interview where a researcher will ask questions about your experiences of your child being admitted to hospital. We can do the interview in a place of your choice, for example, in your home or in a meeting room at the

For peer review only - http://bmjopen.bmj.com/site/about/guidelines.xhtml

university. If you wish, your partner or someone close to you to be present. If your child is 6 years of age or older and they want to take part, then they can take part in the interview with you at the same time. We will give your child age appropriate information on the study and ask them to complete their own consent form.

We might ask the person who referred your child to hospital to also take part in this study. We will audio-record the interview so that we can transcribe what you said and then analyse it. The person who is transcribing (i.e. putting into written text what you have said) will be a third party (i.e. not one of the research team) who will not know who you are. You will have up to 7 days after your interview to let us know if you no longer want us to use your comments. Some parents may find the interview upsetting. If you get upset our experienced interviewers will encourage you to take breaks, you can stop at any time and not take part any more.

What are the possible benefits of taking part?
There will be no direct personal benefit in taking part in this study. However, we hope that your views will be useful in improving the care of children in the future.

What are the possible risks of taking part?
There is a possibility that some parents may become upset. Our staff are trained and very experienced in providing support during interviews

Will my participation in the study be kept confidential?
Yes. We will keep your personal information, such as your name, address and telephone number, confidential in records held in Stirling University. We will record the interview so that your comments can be transcribed and analysed afterwards. We might quote something you said in project publications but we will make sure you cannot be identified. Your comments will not be linked to your name or other personal information and will be stored securely. All data will be managed in line with current data protection guidelines.

If you tell us something during the study which gives us cause for concern about your safety or that of your child, then we have a duty of care to take appropriate action. This could mean that we cannot keep what you told us confidential.

How will we use information about you?
We will need to use information from you for this research project.
This information will include your name and contact details. People will use this information to do the research or to check your records to make sure that the research is being done properly.

People who do not need to know who you are will not be able to see your name or contact details. Your data will have a code number instead. We will keep all information about you safe and secure.

For peer review only - http://bmjopen.bmj.com/site/about/guidelines.xhtml

Once we have finished the study, we will keep some of the data so we can check the results. We will write our reports in a way that no-one can work out that you took part in the study.

What are your choices about how your information is used?
You can stop being part of the study at any time, without giving a reason, but we will keep information about you that we already have.
We need to manage your records in specific ways for the research to be reliable. This means that we won't be able to let you see or change the data we hold about you.

Where can you find out more about how your information is used?
You can find out more about how we use your information

- at www.hra.nhs.uk/information-about-patients/
- our leaflet available from the research team
- by sending an email to dpo@abdn.ac.uk, or by ringing us on 01224-272596.

Who has reviewed this project?
The North of Scotland Research Ethics Committee and specialists in research and health care have reviewed this project.

Who is funding this study?
The Chief Scientist's Office of the Scottish Government.

What if I want to complain?
If you have a concern about any aspect of this project, you should ask to speak to the researchers first 01786-466277. If you are still unhappy and wish to complain formally, you can do this by contacting the University of Aberdeen Research Governance Team via researgovernance@abdn.ac.uk or by phoning 01224-554362.

What do I do if I want to take part?
If you are interested in taking part please call the researcher Emma King at the University of Stirling on 01786-466277, or you can email us on flamingo@stir.ac.uk

Contact details for queries: Dr Emma France. Telephone 01786-466421. Email: emma.france@stir.ac.uk

For peer review only - http://bmjopen.bmj.com/site/about/guidelines.xhtml

UNIVERSITY of STIRLING UNIVERSITY OF ABERDEEN Edinburgh Napier UNIVERSITY FLAMINGO Flow of hospital admissions in children and young people

FLAMINGO

FLow of AdMissions to hospital in chIldren and younG peOple

Experience of unwell children needing a short stay in hospital (less than 24hrs)? We would like to talk with you.

We would like to invite you to participate in a research project. Before you decide, it is important for you to understand why the research is being done and what it will involve. Please take time to read this following information carefully and to discuss it with others if you wish. Ask us if there is anything that is not clear or if you would like more information. Take time to decide whether you wish to take part. Thank you for reading this.

What is the purpose of the research?

The aim of this study is to understand why children are admitted to hospital. To achieve this aim we are using both quantitative and qualitative methods. We are keen to talk to clinicians or practitioners who refer and receive acute paediatric medical cases, so that we can gain a clinician's perspective on why children are admitted to hospital.

Why have I been chosen?

We have approached you about this study because you are a clinician or practitioner who is involved in acute paediatric admissions to hospital.

Do I have to take part?

No, it is up to you to decide whether or not to take part.

What happens if I take part?

You will take part in a short (about 10 minute) telephone interview where a researcher will ask questions about the decision making which leads to a child being admitted to hospital or discharged home.

What are the possible benefits of taking part?

There will be no direct personal benefit in taking part in this study. However, we hope that your views will be useful in improving the care of children in future.

What are the possible risks of taking part?

There are no risks.

Will participation in the study be kept confidential?

Yes. We will keep your personal information, such as your name and telephone number, confidential. We will record the interview so that we can then transcribe and analyse it. We might quote something you said in project publications but we will make sure you cannot be identified. Your comments will not be linked to your name, workplace name or other personal information and will be stored securely. All data will be managed in line with current data protection guidelines.

For peer review only - http://bmjopen.bmj.com/site/about/guidelines.xhtml

The University of Aberdeen is the sponsor for this study based in the United Kingdom. We will be using information from you in order to undertake this study and will act as the data controller for this study. This means that we are responsible for looking after your information and using it properly. The University of Aberdeen will keep identifiable information about you for 10 years after the study has finished.

Your rights to access, change or move your information are limited, as we need to manage your information in specific ways in order for the research to be reliable and accurate. If you withdraw from the study, we will keep the information about you that we have already obtained. To safeguard your rights, we will use the minimum personally-identifiable information possible.

Your local NHS board will keep your name, and contact details confidential and will not pass this information to University for Aberdeen. Your NHS board will use this information as needed, to contact you about the research study, and make sure that relevant information about the study is recorded for your care, and to oversee the quality of the study. Certain individuals from University of Aberdeen and regulatory organisations may look at your research records to check the accuracy of the research study. The University of Aberdeen will only receive information without any identifying information. The people who analyse the information will not be able to identify you and will not be able to find out your name, or contact details. Your local NHS board will not keep identifiable information about you from this after the study has finished.

You can find out more about how we use your information at http://www.abdn.ac.uk/privacy

Who has reviewed this study?
The study has been reviewed by the North of Scotland Research Ethics Committee and also specialists in the field.

What if I want to complain?
If you have a concern about any aspect of this project, you should ask to speak to the researchers first on 01786-466277. If you are still unhappy and wish to complain formally, you can do this by contacting Professor Jayne Donaldson, the Dean of the Faculty of Health Sciences and Sport, University of Stirling on 01786-466340 or jayne.donaldson@stir.ac.uk.

You have the right to lodge a complaint against the University of Stirling regarding data protection issues with the Information Commissioner's Office (https://ico.org.uk/concerns/).
The University's Data Protection Officer is Joanna Morrow, Deputy Secretary. If you have any questions relating to data protection these can be sent to data.protection@stir.ac.uk.

What do I do now?
If you are interested in taking part, please contact the researcher Dr Emma King at the University of Stirling on 01786-466277. Email flamingo@stir.ac.uk

If you require any further information, Dr Emma France. Telephone 01786-466421. Email: emma.france@stir.ac.uk

For peer review only - http://bmjopen.bmj.com/site/about/guidelines.xhtml

DAG model Nov 2021

Purpose and population

Intervention development in the pathway for unscheduled acute illness in children, access to care and admissions to hospital

Whole Systems and Multi-Stakeholder Approach to Intervention Development

Systematic Review Evidence

Programme Theory

Context - macro
- Rising admissions
- Covid pandemic
- NHS staff shortages and workload
- Health Inequalities

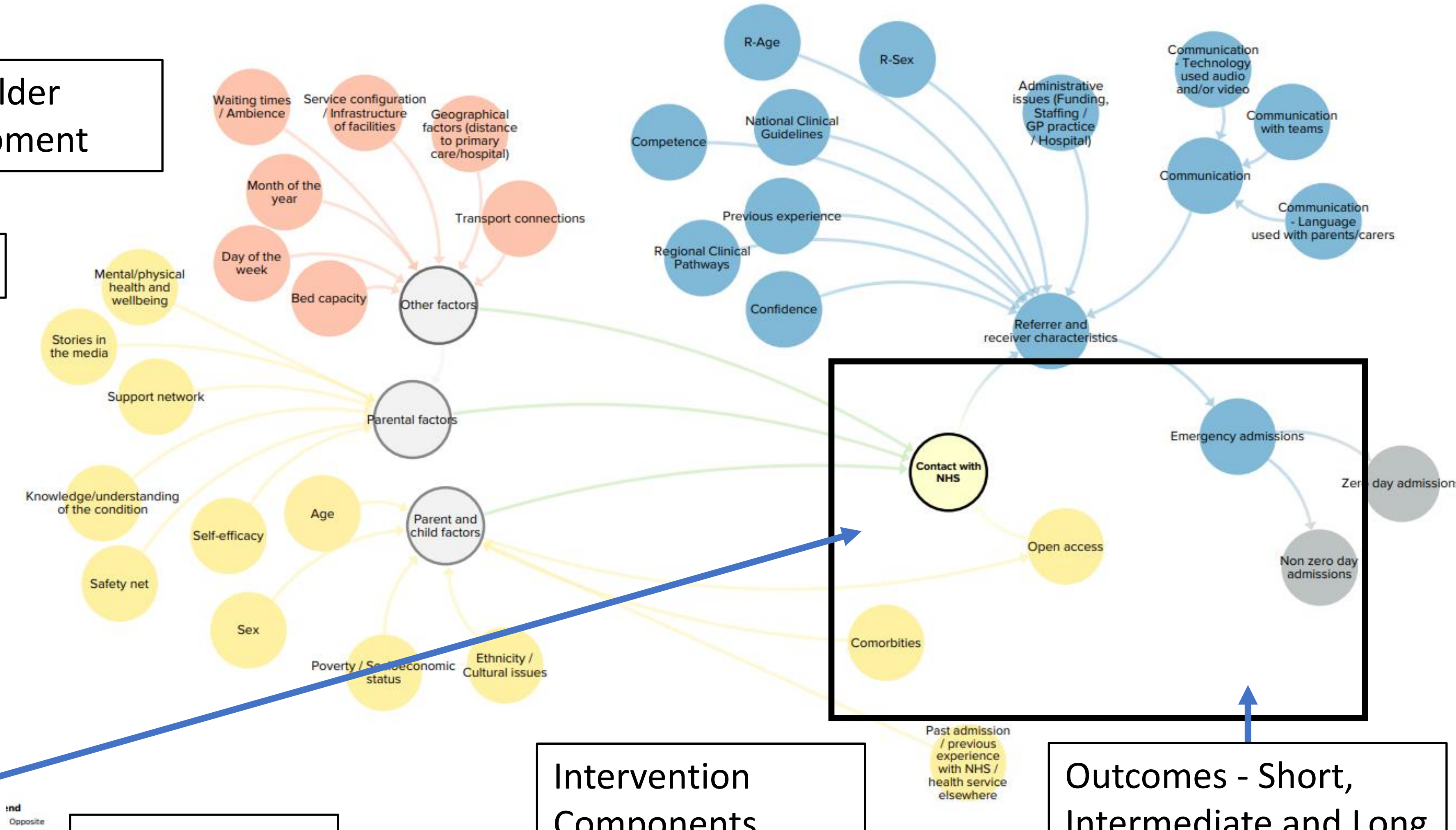

Right method, time, place, person, process,

Family, Staff and Society Values

Intervention Components, Processes and Mechanisms: TIDieR

Outcomes - Short, Intermediate and Long Term that matter to children, families, staff, society

FLAMINGO Revised Logic Model post data analysis

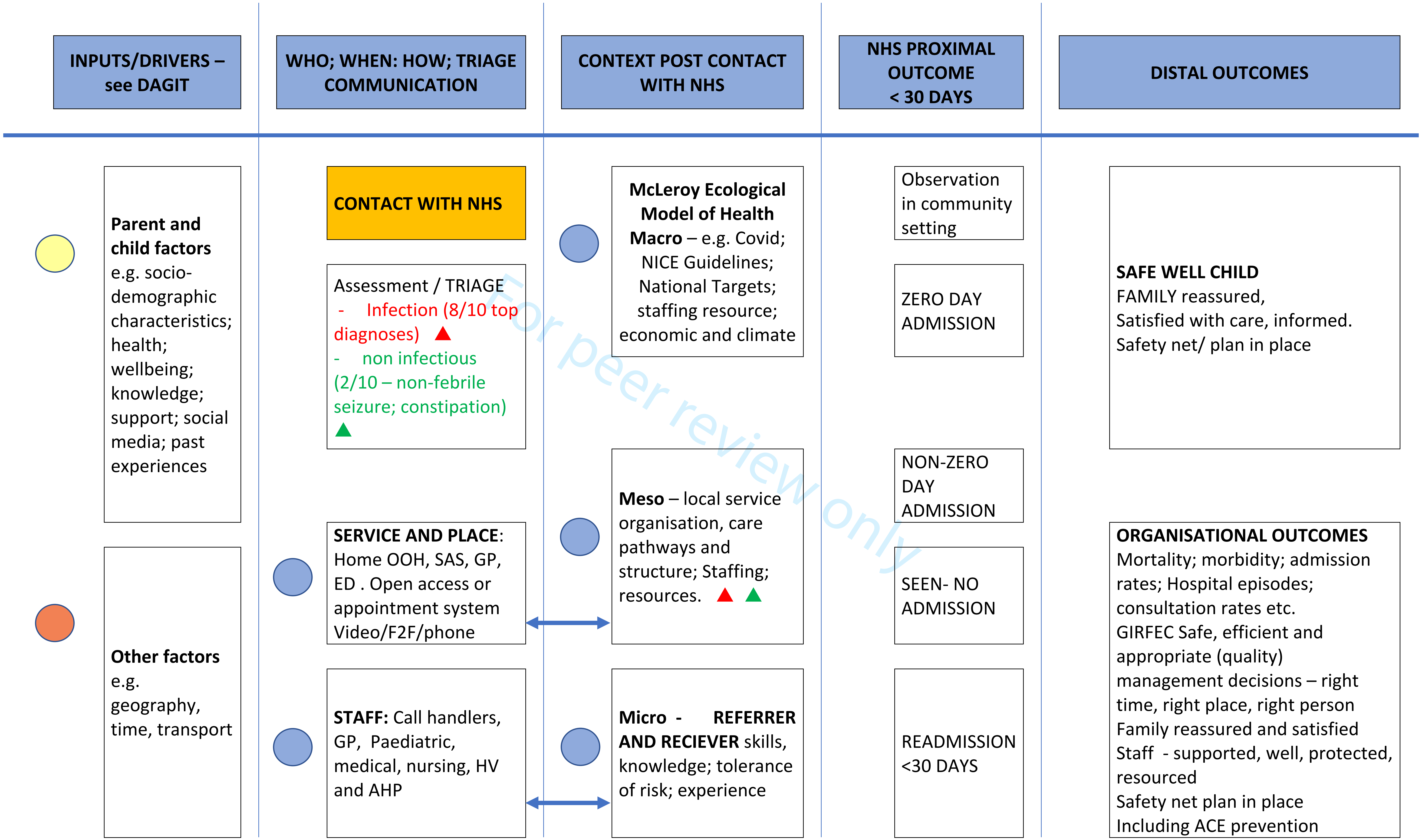

For peer review only

Supplementary file 6. Flamingo stakeholder event methods and materials

Purpose/objective

To present project findings and to discuss prioritisation of potential interventions.

Setting

The five-hour event was held on 3 September 2021 at Edinburgh Zoo, a location accessible for most stakeholders. Travel expenses were offered to families and health professionals who had taken part in interviews.

Participants

The face-to-face and online attendees included parent and health professional interviewees, health professionals, Principle Investigators for the case study sites and a purposive sample of professional contacts of the team with relevant experience, a representative from the study funder the Chief Scientist Office, and representatives from stakeholder organisations e.g. charities and parent groups.

Agenda

10-10.15 Introduction

10.15-11.00* What we found

11-12ish Group discussions followed by time to explore the zoo

12.30-1.15 Lunch

1.15-2.15* Opportunities for change

2.15-2.45 Group discussions

2.45-3.00 Wrap up

*tea and coffee available

Procedures & data collection

The Stakeholder Event discussed FLAMINGO project findings in the morning and prioritisation of potential new interventions in the afternoon. Attendees were split into six groups, five in-person and one online. In-person attendees were seated at pre-allocated tables of five attendees with a mix of families, health professionals and other stakeholders (e.g. third sector or government

For peer review only

representatives). One member from the FLAMINGO team (SD, EF, EK, ST, or PW) facilitated each table's discussion. An online group took place via Microsoft Teams© facilitated by two remote members of the FLAMINGO team (CM, RK). Facilitators took written notes during or after the discussion and audio-recorded all discussions to assist accurate note-taking. To ensure all contributions were captured, attendees were also asked to write any ideas they had on post-it notes which were collated and recorded. After the morning presentations, facilitators asked each group three questions to stimulate discussion about the study findings:

1. What is the most important thing you have heard this morning?
2. What surprised you the most?
3. What did not surprise you?

In the afternoon the project team presented the seven potential future health systems interventions Subsequently, the groups were asked the following two questions to spark discussion about prioritisation of interventions:

1. If you were in charge, what would you change first and why?
2. Imagine you are in charge five years after your first choice has been in action. What problems would people come to you with about your "new" service?

The second question drew on Klein and Kahneman & Klein's [1, 2] 'pre-mortem' method to improve decisions, which asks groups to prospectively imagine the future and predict what has gone wrong; the pre-mortem can overcome the suppression of dissenting views and increases identification of risks from a project's outset.

Finally, attendees were asked to individually rank the priority of seven interventions during the stakeholder event on a pro-forma, where 1 was the highest priority and 7 was the lowest. Online participants accessed an online pro-forma.

Analysis

Notes taken during discussions were checked for accuracy and completeness against the audio-recordings where possible, however, due to high background noise in two recordings facilitators produced more detailed notes of the discussions from memory and post-it notes were checked and incorporated into the discussion notes. Notes were uploaded into NVivo and were coded (by EF and EK) under broad codes for each of the seven interventions with separate codes for confirming or

disconfirming perspectives, plus a further code for other interventions attendees suggested. Codes were read repeatedly and themes identified. For the intervention priority ranking exercise, scores were summed and the average rank calculated for each of the seven interventions.

1. Klein G. Performing a Project PreMortem. Harvard Business Review. 2007.
2. Kahneman D, Klein G. Conditions for intuitive expertise: A failure to disagree. American Psychologist. 2009;64(4):515-26.

For peer review only

FLAMINGO
Flow of hospital admissions in children and young people - Introduction

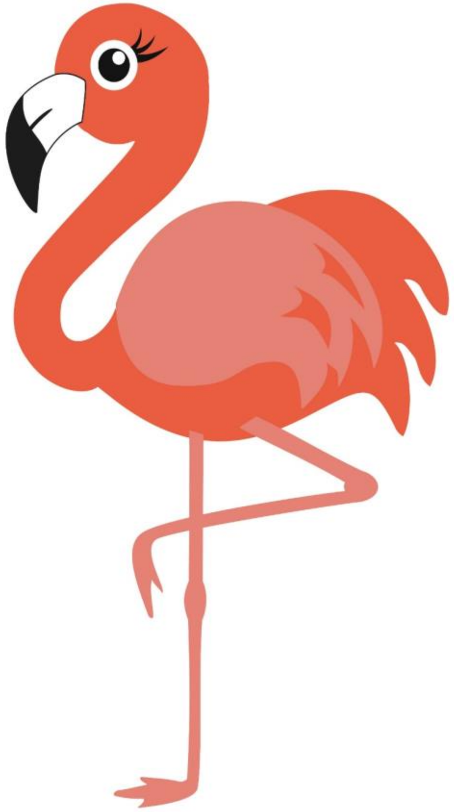

Good morning. Welcome

- Housekeeping
- Thanks
- Why are we meeting?
- Plan for the day
- Introductions

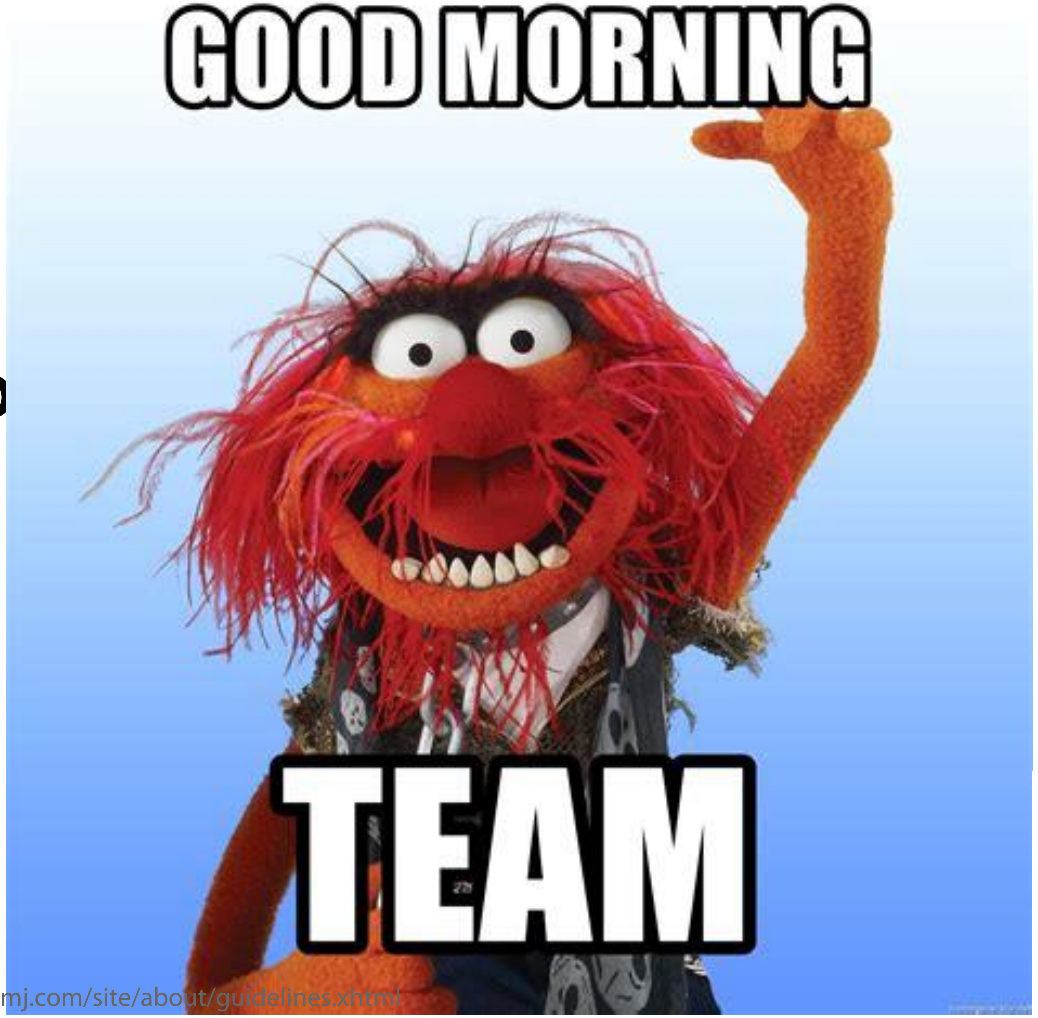

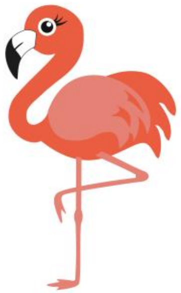

CHIEF SCIENTIST OFFICE

For peer review only - http://bmjopen.bmj.com/site/about/guidelines.xhtml

Housekeeping

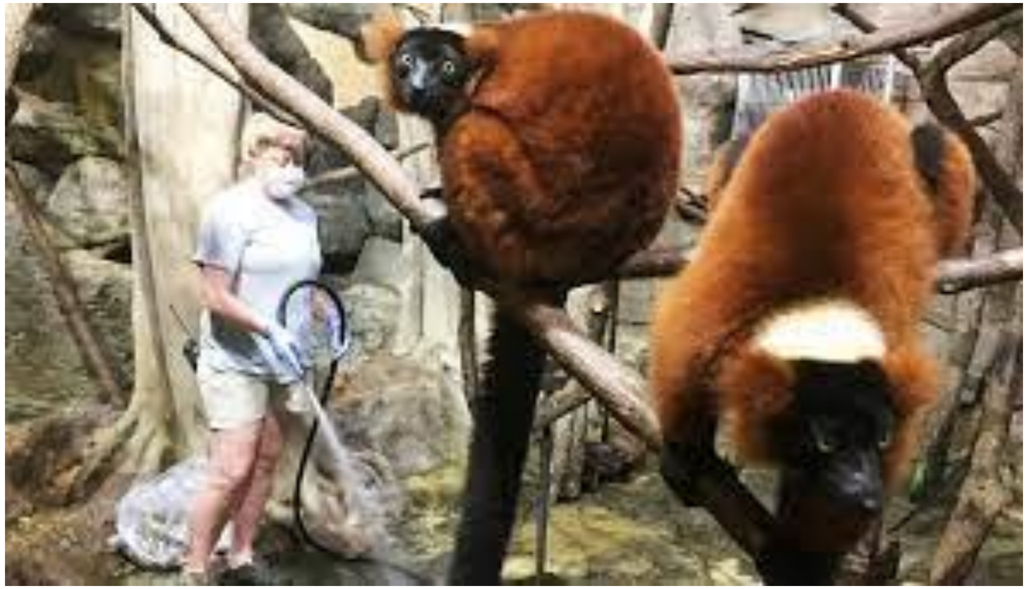

- Fire exits, toilets, mobile phones
- Please don’t share/tweet
- Permission to record (note taking)
- COVID considerations
- Please speak into microphone
- No jargon. Please don’t be afraid to ask for clarification
- Teams. Camera on, microphone on mute please. Raise hand.

CHIEF SCIENTIST OFFICE

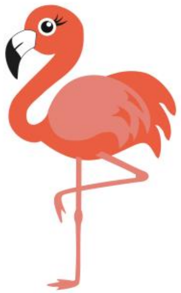

Why are we meeting?

- Zero day admissions rising
- Is there a……..
- Win (children)
- Win (parents/carers)
- Win (primary care)
- Win (secondary care)
- Win (NHS Scotland/Government)

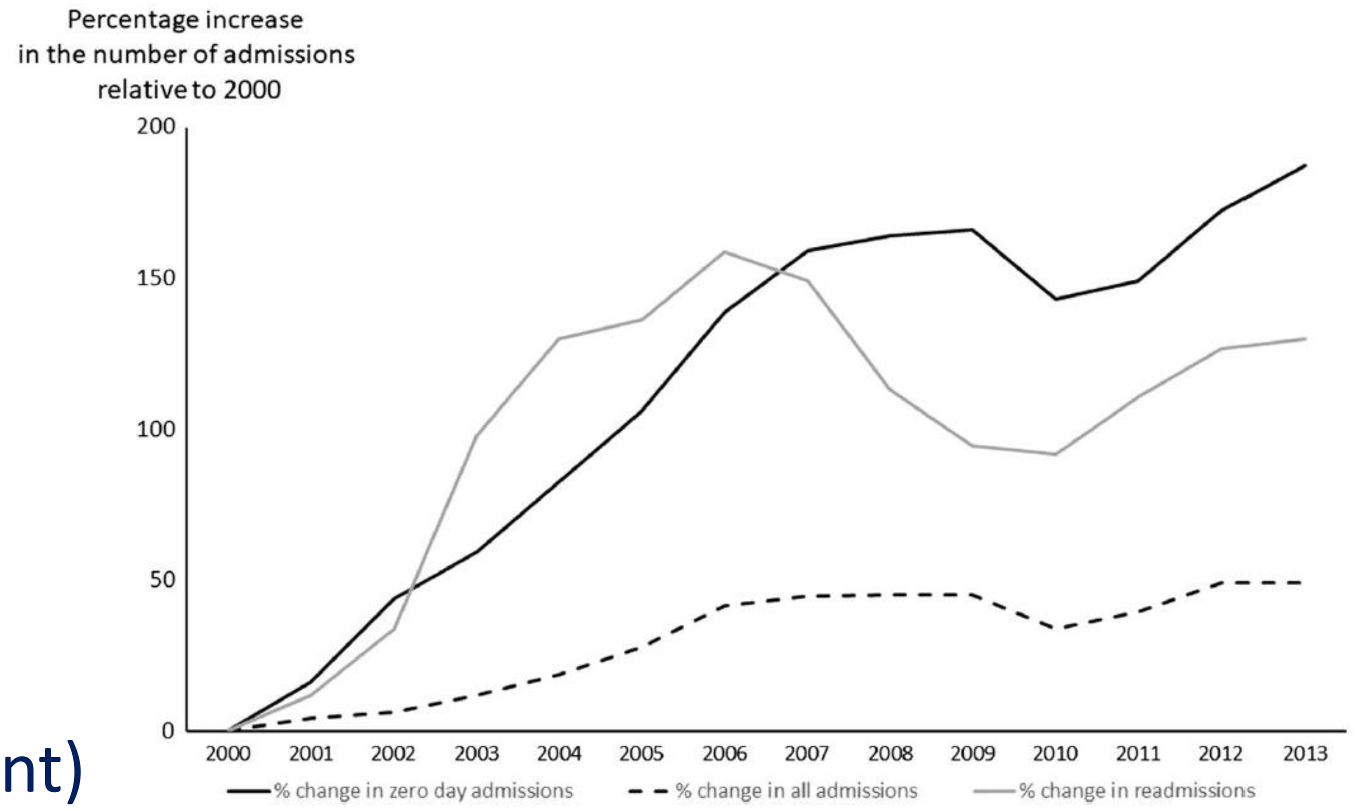

Eur J Pediatr 2018 ;177:381-388

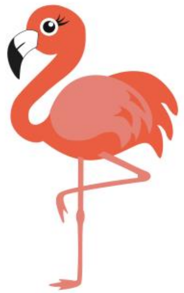

CHIEF SCIENTIST OFFICE

Why are we meeting?

This is stakeholder engagement

Two main aims

1. Share results with you
   - What surprises you
2. What are the priorities?
   - What would you do first?

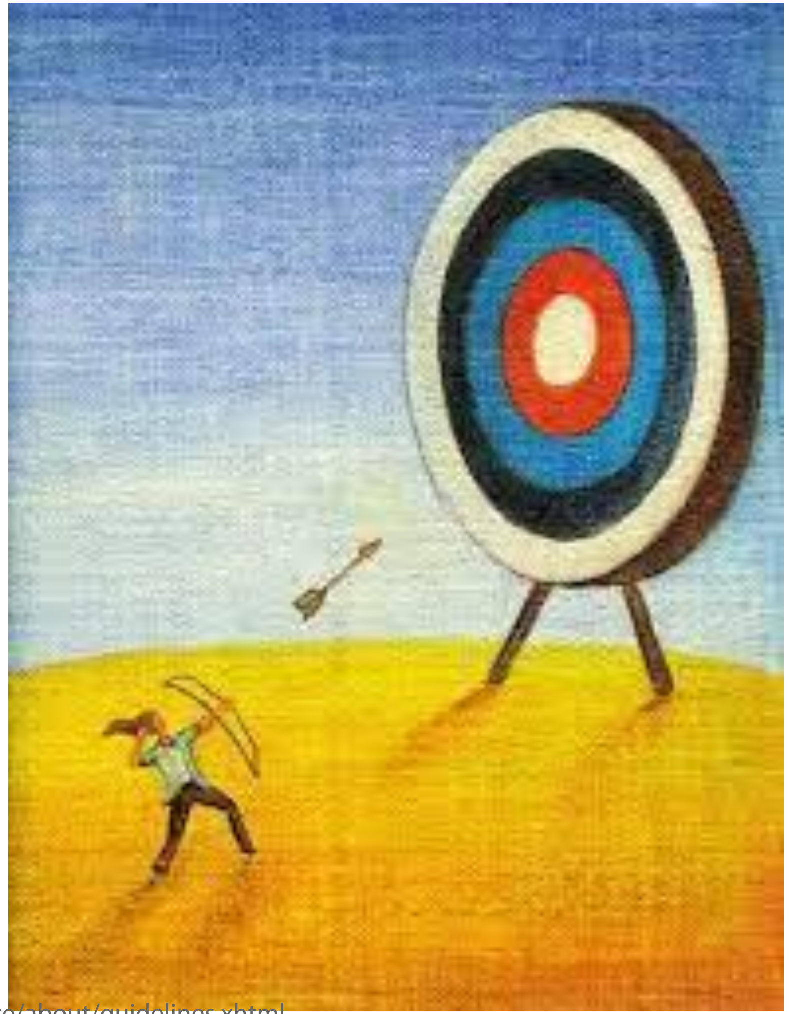

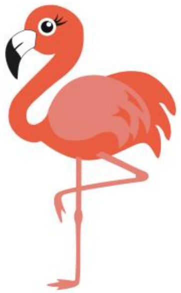

CHIEF SCIENTIST OFFICE

For peer review only - http://bmjopen.bmj.com/site/about/guidelines.xhtml

What is the plan for today?

10-10.15 introduction

10.15-11.00* What we found

11-12ish Group discussions, zoo

12.30-1.15 Lunch

1.15-2.15*   Opportunities for change

2.15-2.45   Group discussions

2.45-3.00   Wrap up

*

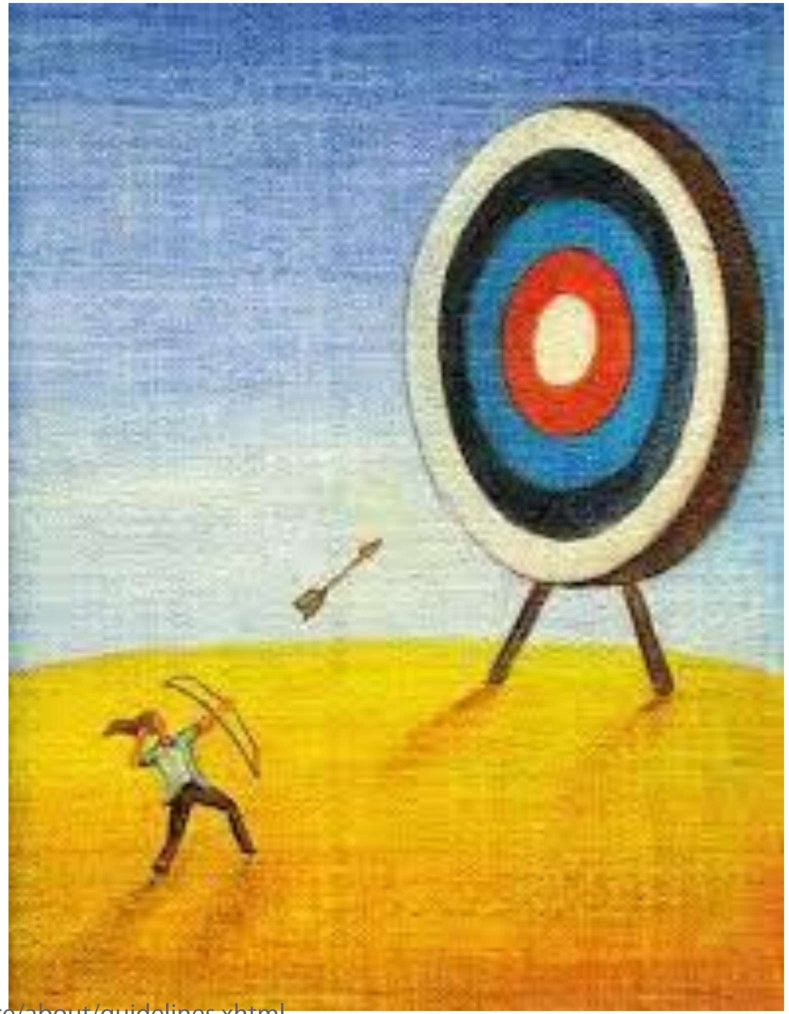

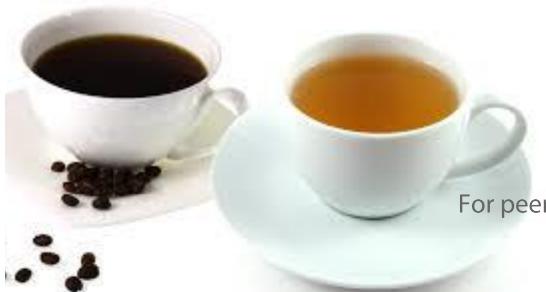

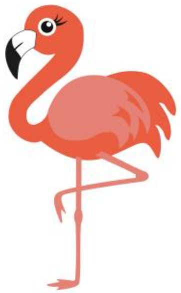

CHIEF SCIENTIST OFFICE

For peer review only - http://bmjopen.bmj.com/site/about/guidelines.xhtml

Introductions

- Who is here today?

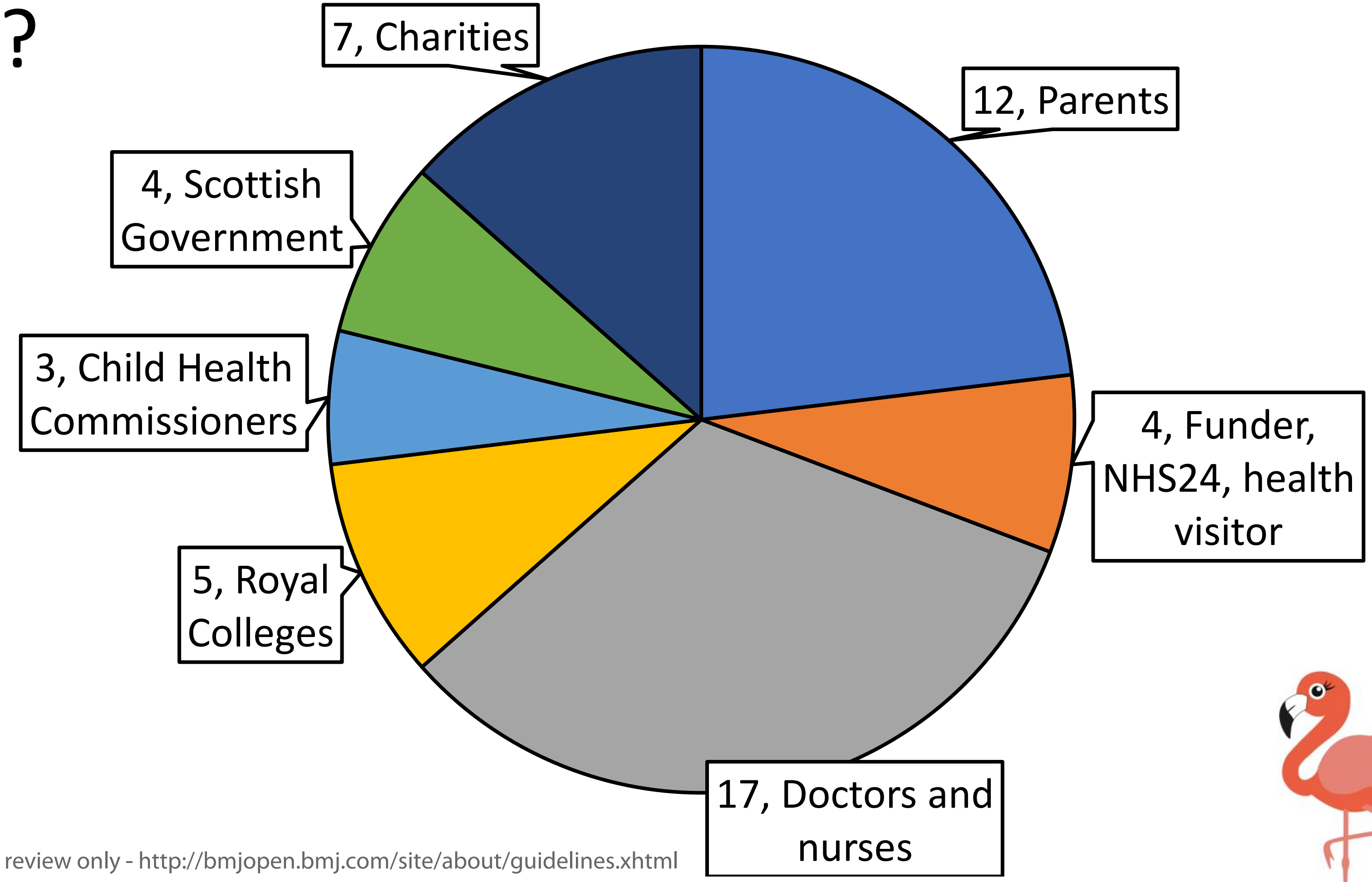

CHIEF SCIENTIST OFFICE

Introductions

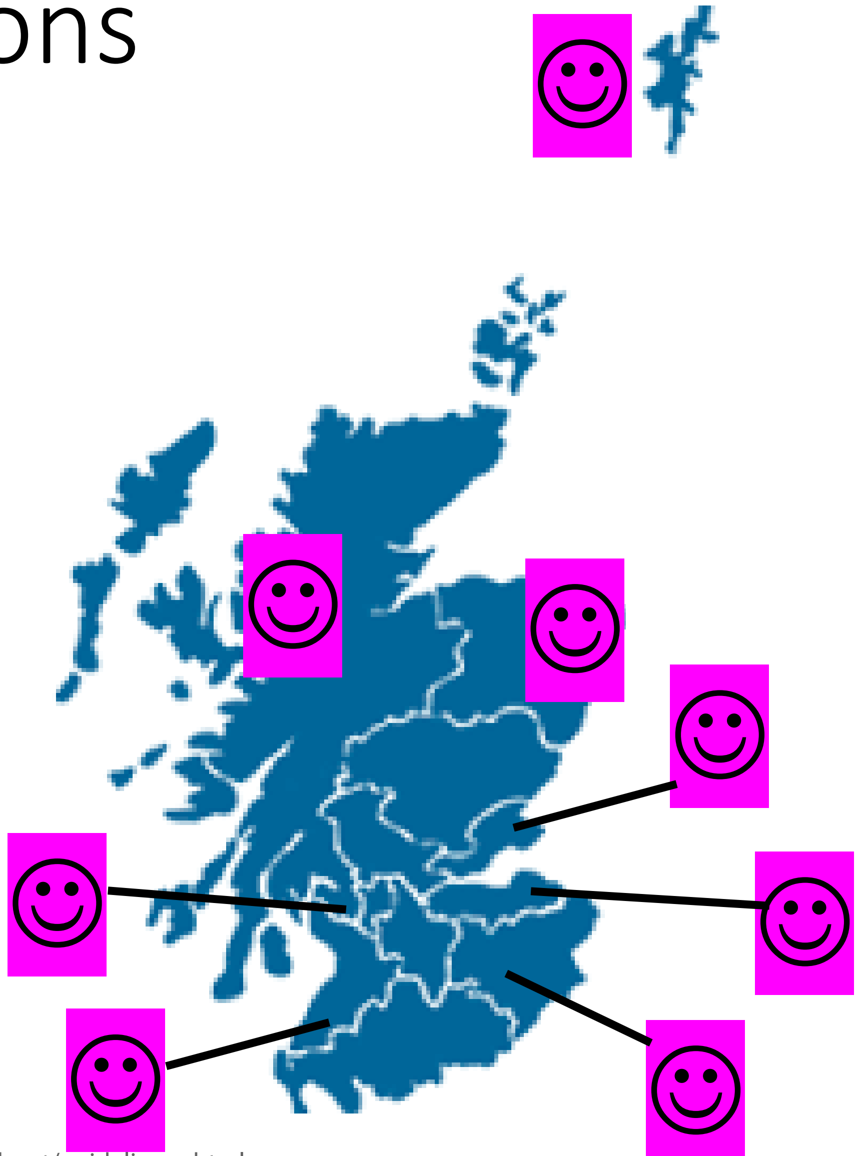

- ~35+5 in person
- ~19+2 on Teams
- Some just for am/pm
- ?no reps from
  - Orkney
  - Western Isles
  - Tayside
  - D&G
  - Forth Valley
  - Lanarkshire

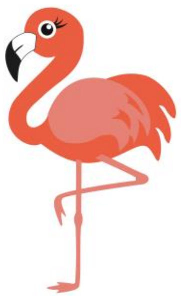

CHIEF SCIENTIST OFFICE

Introductions

Please spend a few minutes

At the Zoo:

- Name
- Which group you are representing
- Why are you here?

Teams:

Please put this in the chat

Note to self:

What do you want to achieve by 3pm?

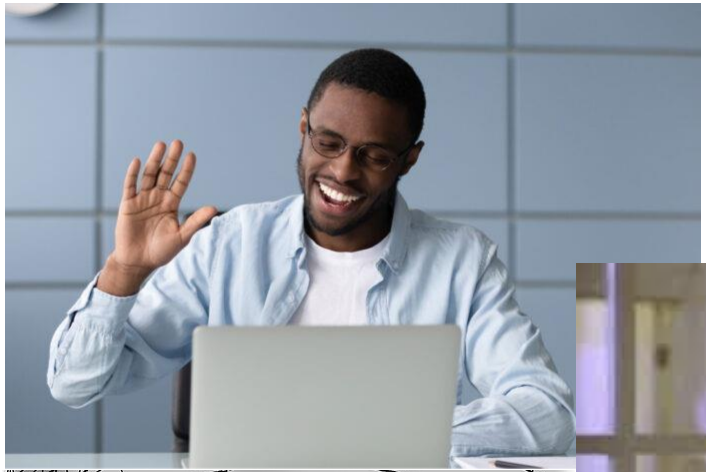

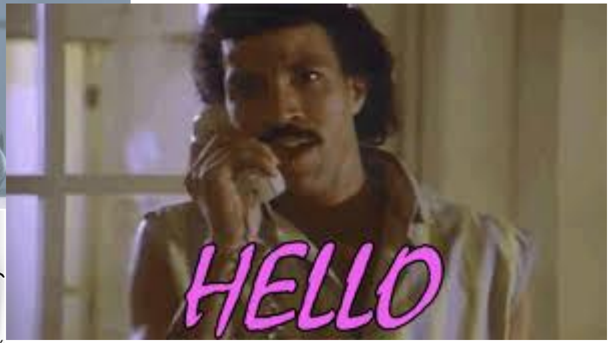

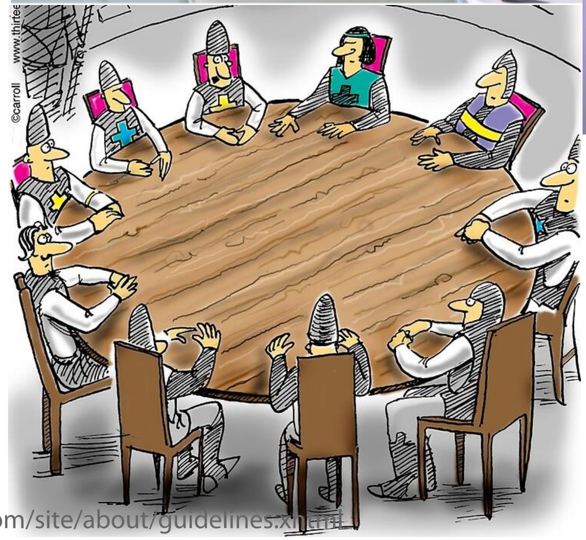

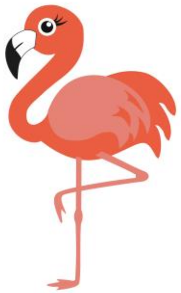

CHIEF SCIENTIST OFFICE

For peer review only - http://bmjopen.bmj.com/site/about/guidelines.xhtml

FLAMINGO

Flow of hospital admissions in children and young people – Methods and Results

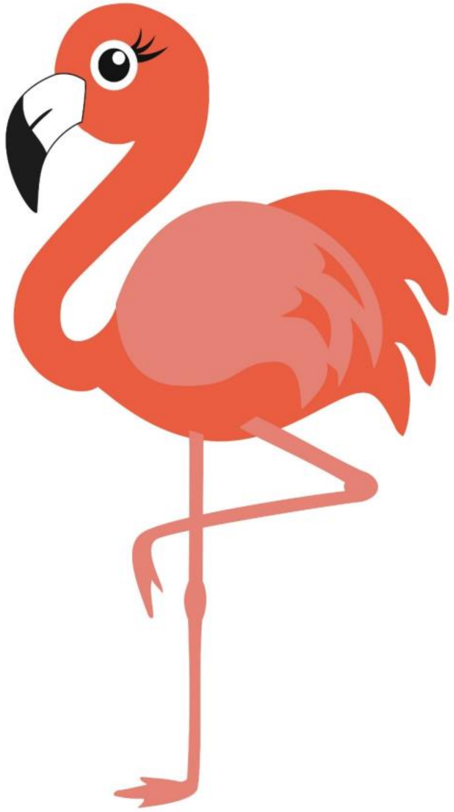

CHIEF SCIENTIST OFFICE

Overview

- Definitions
- Why we did this
- How we did this
- What we found

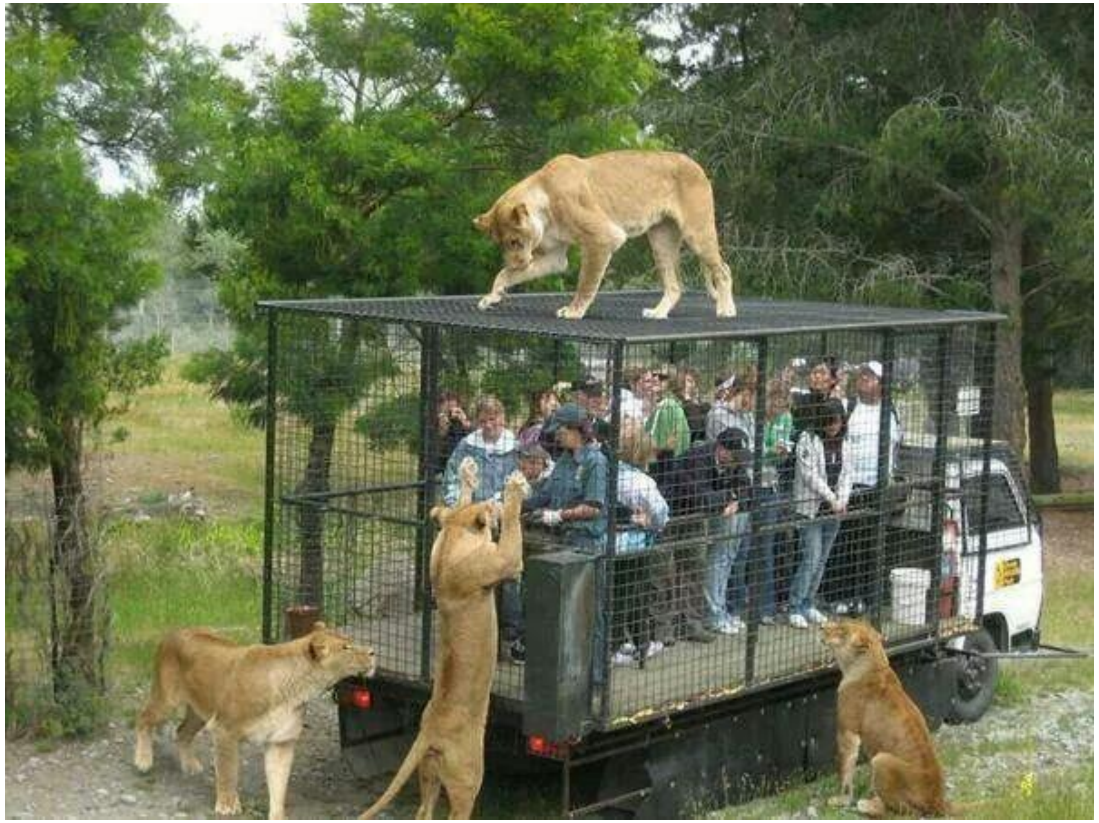

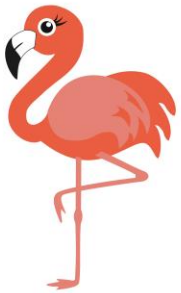

CHIEF SCIENTIST OFFICE

Definitions

- Emergency department
- General practice
- NHS 24
- Out of Hours
- Health board
- Admission
- Zero day admission

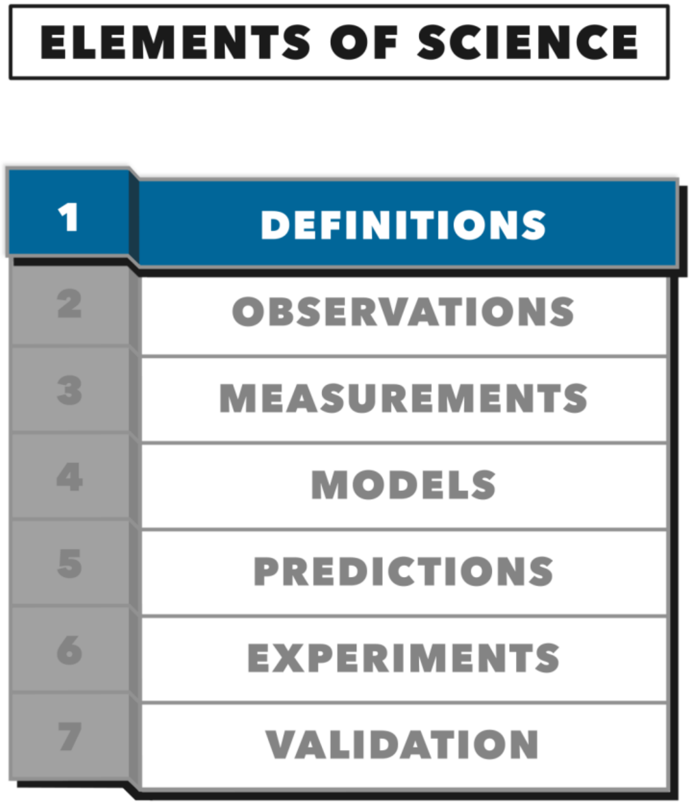

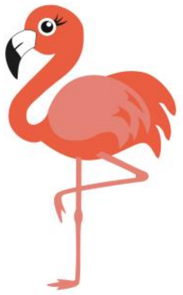

CHIEF SCIENTIST OFFICE

For peer review only - http://bmjopen.bmj.com/site/about/guidelines.xhtml

Why

Risers and fallers?

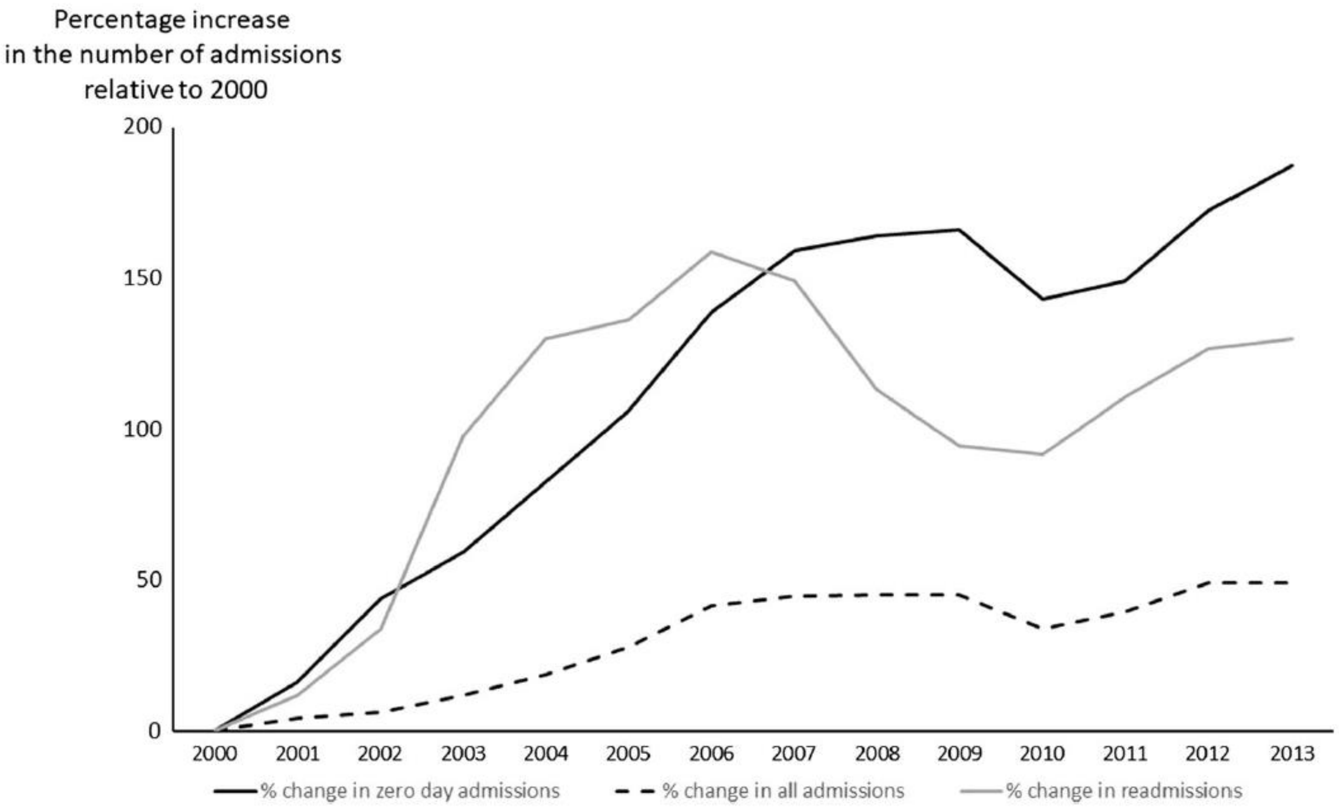

Eur J Pediatr 2018 ;177:381-388

CHIEF SCIENTIST OFFICE

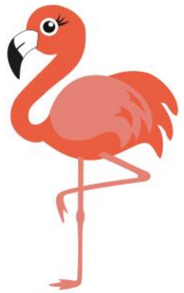

Why

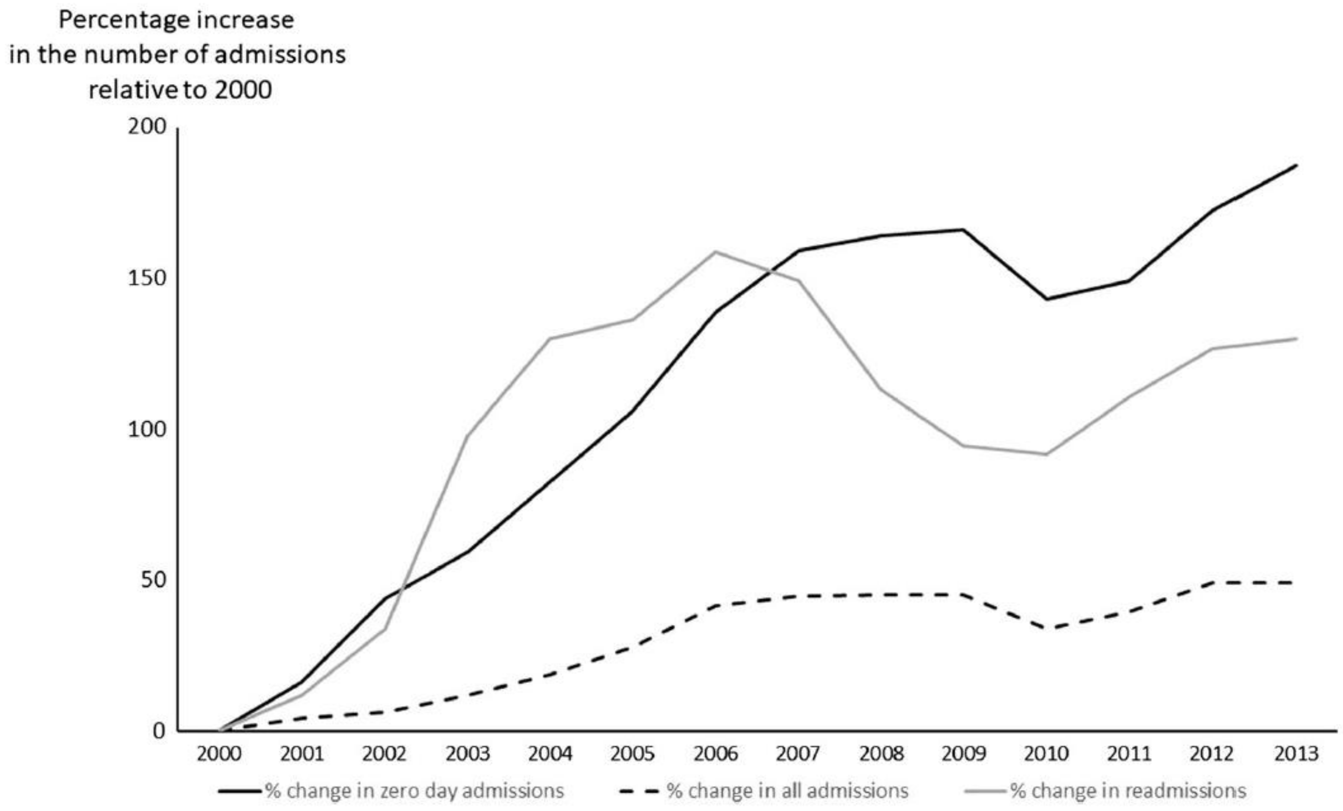

Risers and fallers

↑ 100% “viral infection”

↑45% tonsillitis

↑25% bronchiolitis

↑10% chest infection

↓15% croup

↓15% gastroenteritis

↓25% convulsions

↓25% asthma

Eur J Pediatr 2018 ;177:381-388

CHIEF SCIENTIST OFFICE

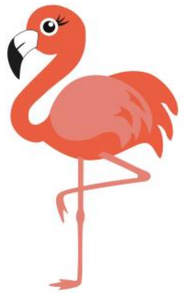

Why

? True change

√ Asthma, convulsions

? Change in parent help-seeking

√ Poverty

√ Expectations

? Change in primary care

? Change in hospital

? All of the above!

Risers and fallers:

↑ 100% "viral infection"

↑45% tonsillitis

↑25[illegible]% bro[illegible]chiol[illegible]tis

↑[illegible]0% c[illegible]est in[illegible]ection

↓15% c[illegible]oup

↓15% gastroenteritis

↓25% convulsions

↓25% asthma

? ? ?

CHIEF SCIENTIST OFFICE

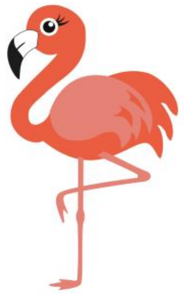

Why

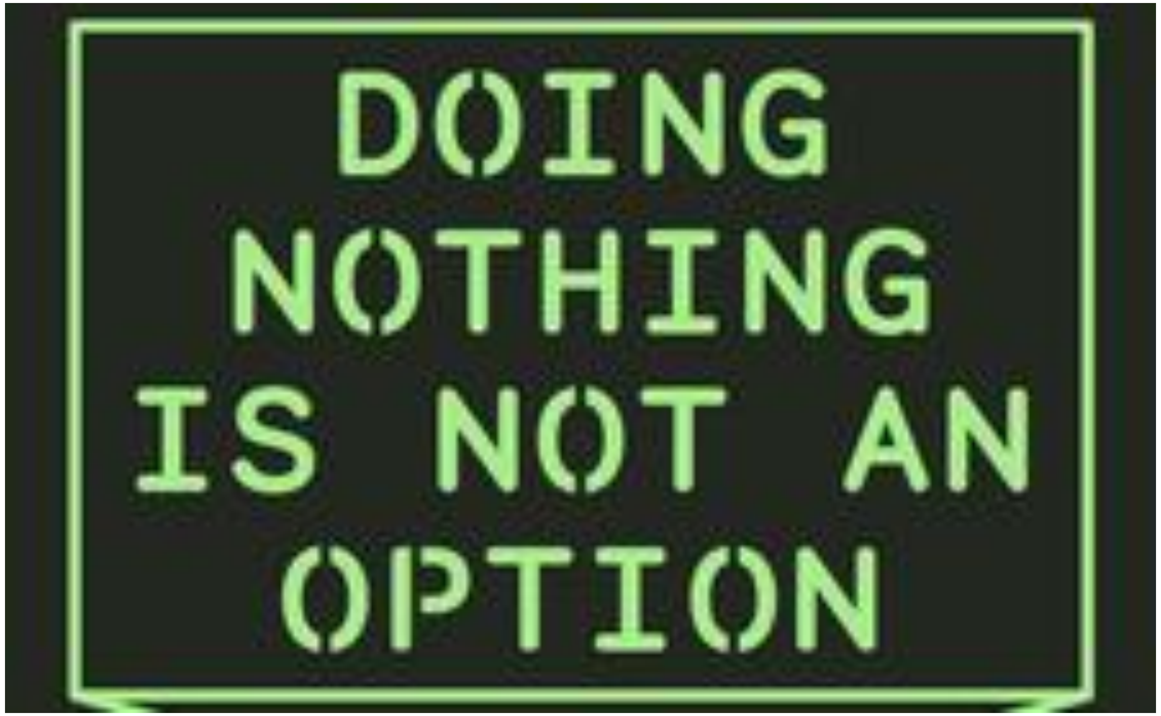

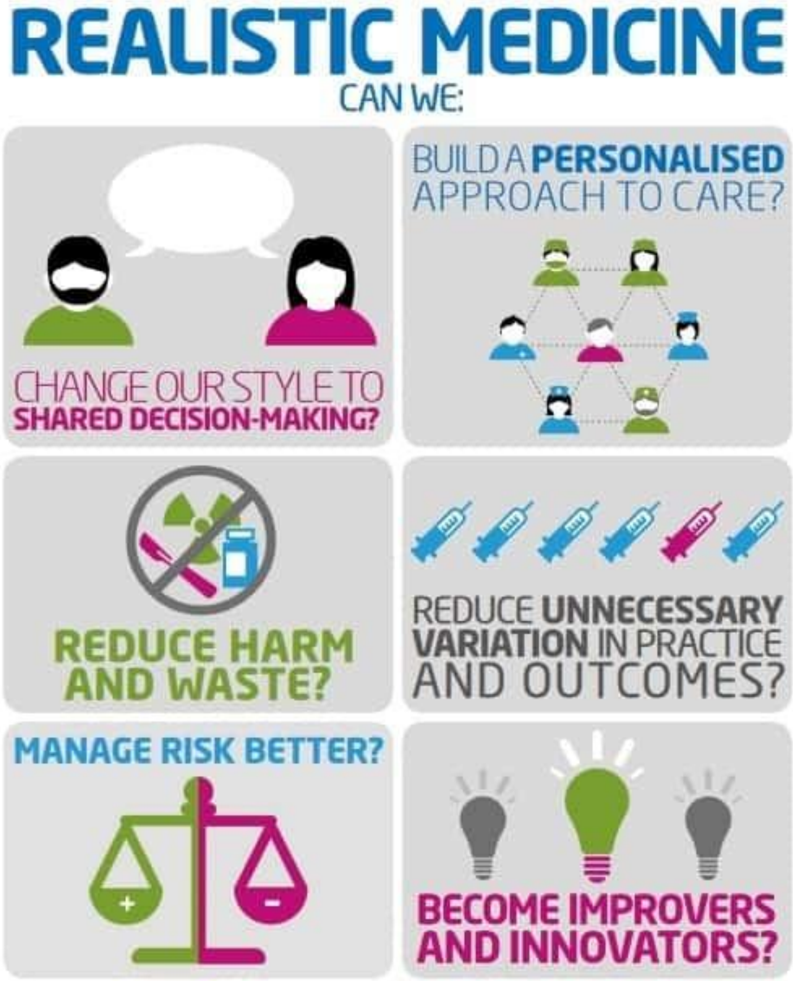

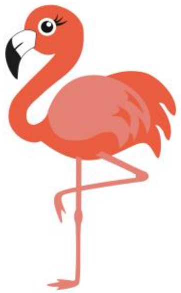

CHIEF
SCIENTIST
OFFICE

Question

1. How can we best involve parents/carers and children in research to improve emergency paediatric admission pathways?

CHIEF SCIENTIST OFFICE

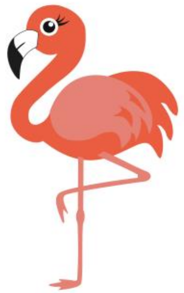

Question and “answer”

1. How can we best involve parents/carers and children in research to improve emergency paediatric admission pathways?

1. Work with parents (mother-baby clubs, hospitals). Pre-COVID.

CHIEF SCIENTIST OFFICE

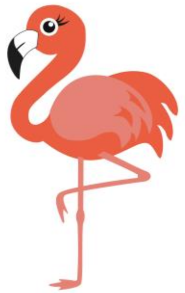

Question and "answer"

1. How can we best involve parents/carers and children in research to improve emergency paediatric admission pathways?
2. Within the 'flow' of zero day admissions, are there identifiable 'streams' of zero day admissions which could be slowed?

1. Work with parents (mother-baby clubs, hospitals). Pre-COVID.

CHIEF SCIENTIST OFFICE

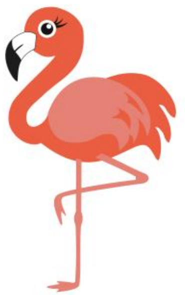

Question and "answer"

1. How can we best involve parents/carers and children in research to improve emergency paediatric admission pathways?
2. Within the 'flow' of zero day admissions, are there identifiable 'streams' of zero day admissions which could be slowed?

1. Work with parents (mother-baby clubs, hospitals). Pre-COVID.
2. Collect data (numbers and words).

CHIEF SCIENTIST OFFICE

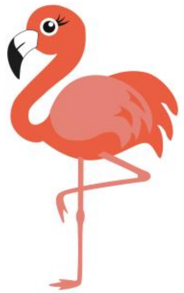

Question and "answer"

1. How can we best involve parents/carers and children in research to improve emergency paediatric admission pathways?
2. Within the 'flow' of zero day admissions, are there identifiable 'streams' of zero day admissions which could be slowed?
3. Which of these 'streams' is the most important to slow down first?

1. Work with parents (mother-baby clubs, hospitals). Pre-COVID.
2. Collect data (numbers and words).

CHIEF SCIENTIST OFFICE

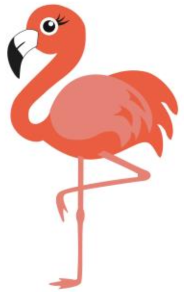

Question and "answer"

1. How can we best involve parents/carers and children in research to improve emergency paediatric admission pathways?
2. Within the 'flow' of zero day admissions, are there identifiable 'streams' of zero day admissions which could be slowed?
3. Which of these 'streams' is the most important to slow down first?

1. Work with parents (mother-baby clubs, hospitals). Pre-COVID.
2. Collect data (numbers and words).
3. Meeting at the zoo.

CHIEF SCIENTIST OFFICE

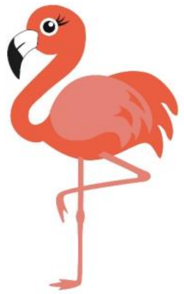

Numbers and words

Numbers = what is happening (quant)

Words = why it is happening (qual)

Mixed methods, sum>parts

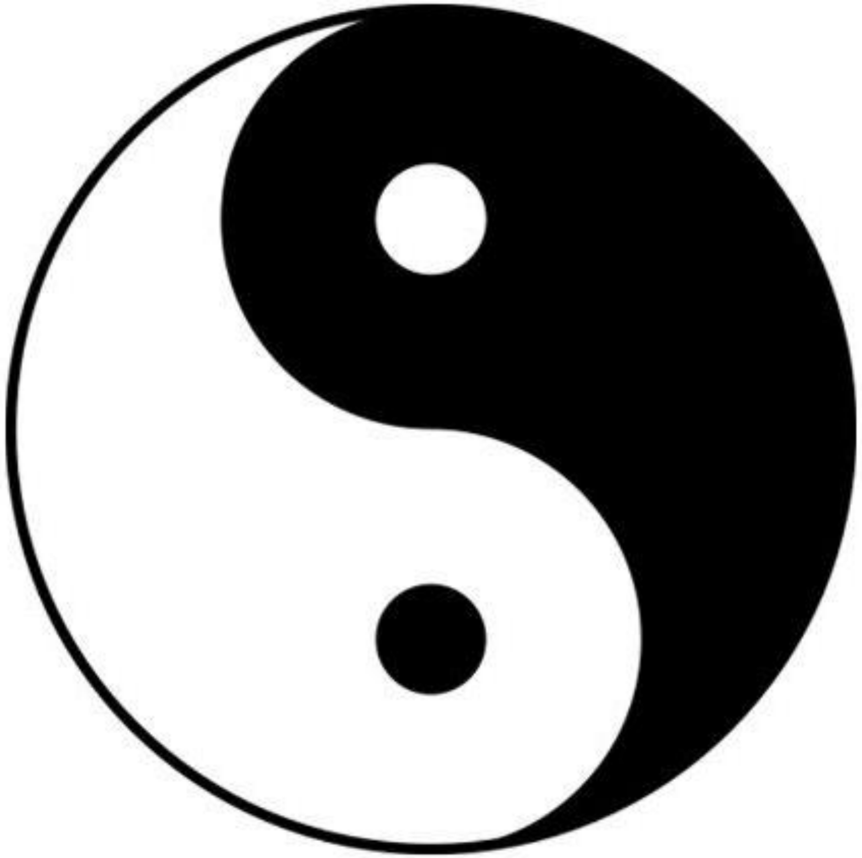

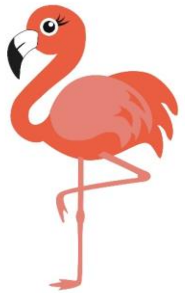

CHIEF SCIENTIST OFFICE

Qualitative Methods

Development of research questions

Visiting a number of parent and toddler groups

Health boards case studies

Five health boards chosen for diversity of geography and population size

Recruitment

Social media, posters, sharing by stakeholder groups, emails to contacts

Telephone interviews

Coding and analysis

Transcription. Coded and analysed by the qualitative team

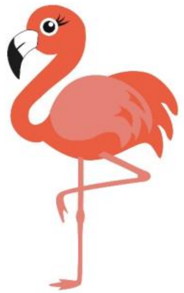

CHIEF SCIENTIST OFFICE

Quantitative Methods

- NHS Scotland collects data
- Collect data from 3 routes into hospital
- 2015-2017
- Link them to map the journey
- Identify “streams” within the “flow”
- Permissions

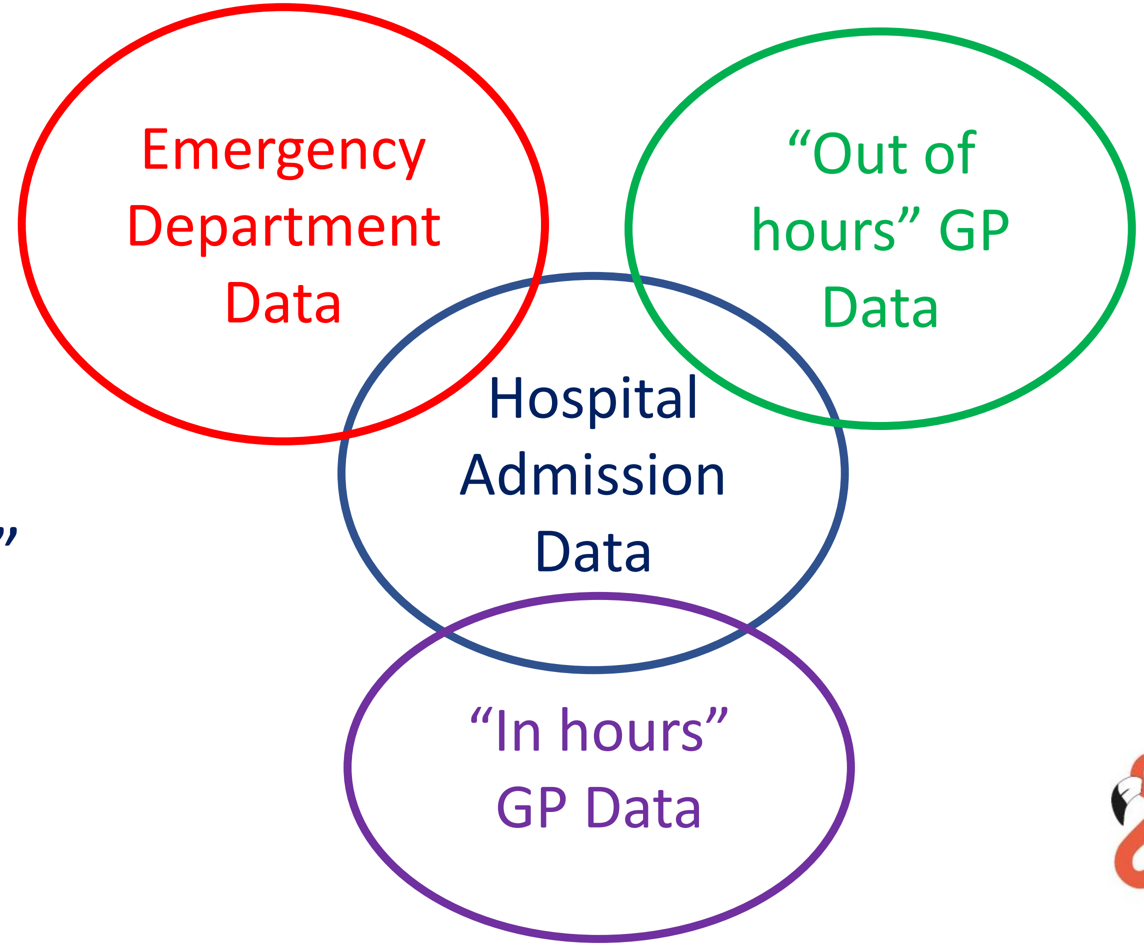

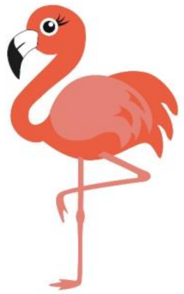

Quantitative Results

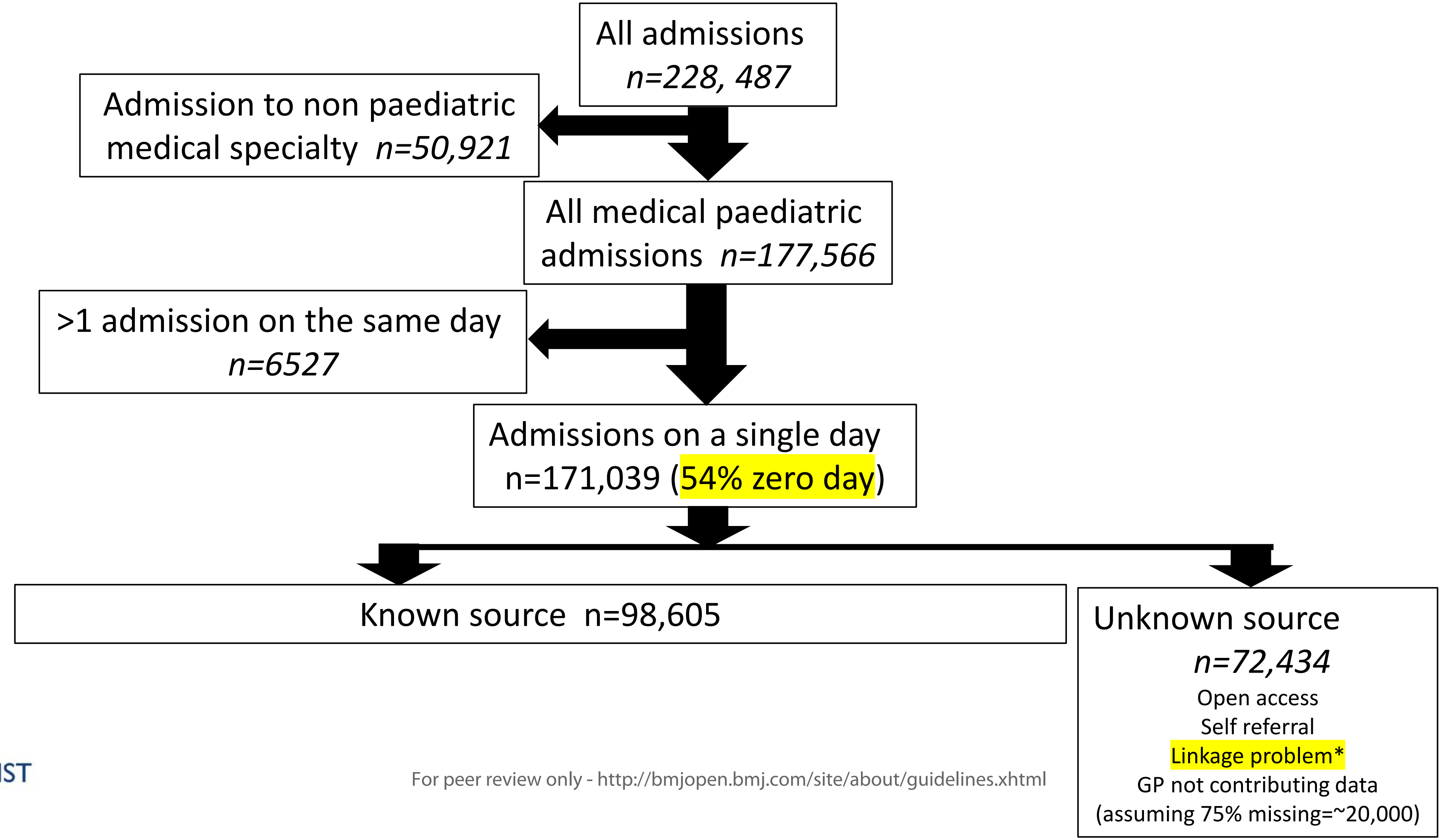

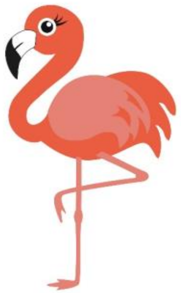

CHIEF SCIENTIST OFFICE

For peer review only - http://bmjopen.bmj.com/site/about/guidelines.xhtml

Quantitative Results

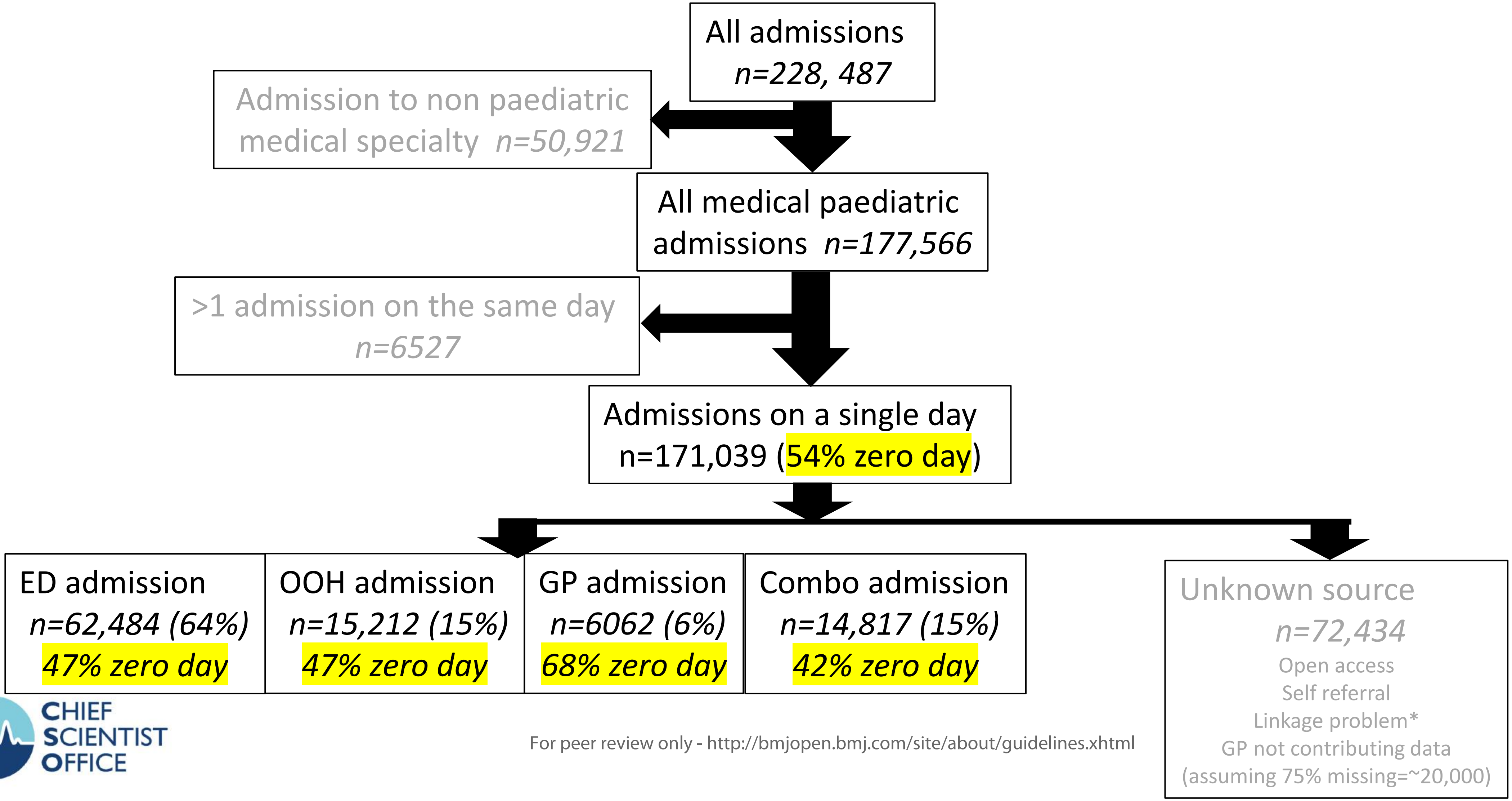

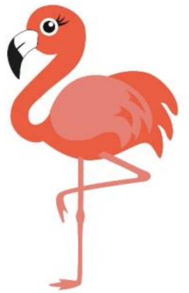

Quantitative Results

- What about regional variation?
- Conditions don't recognise boundaries
- Can this reflect differences in journeys through NHS?

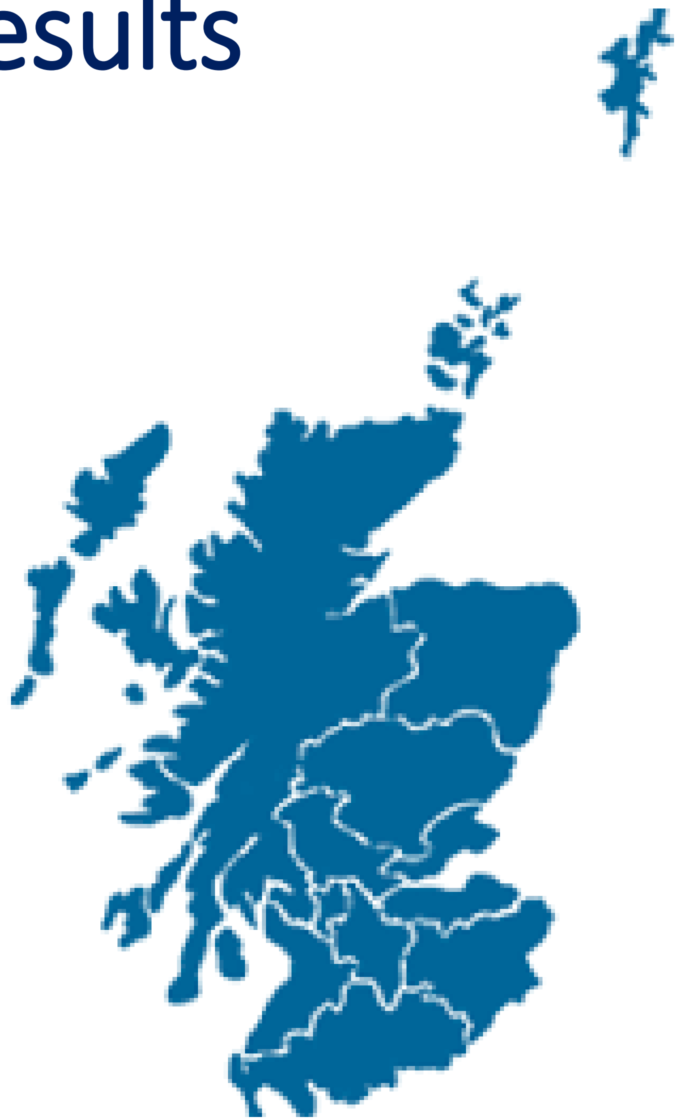

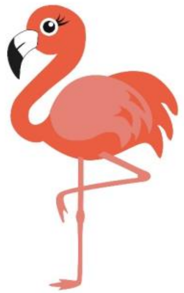

Quantitative Results

- What about regional variation?
- Conditions don't recognise boundaries
- Can this reflect differences in journeys through NHS?

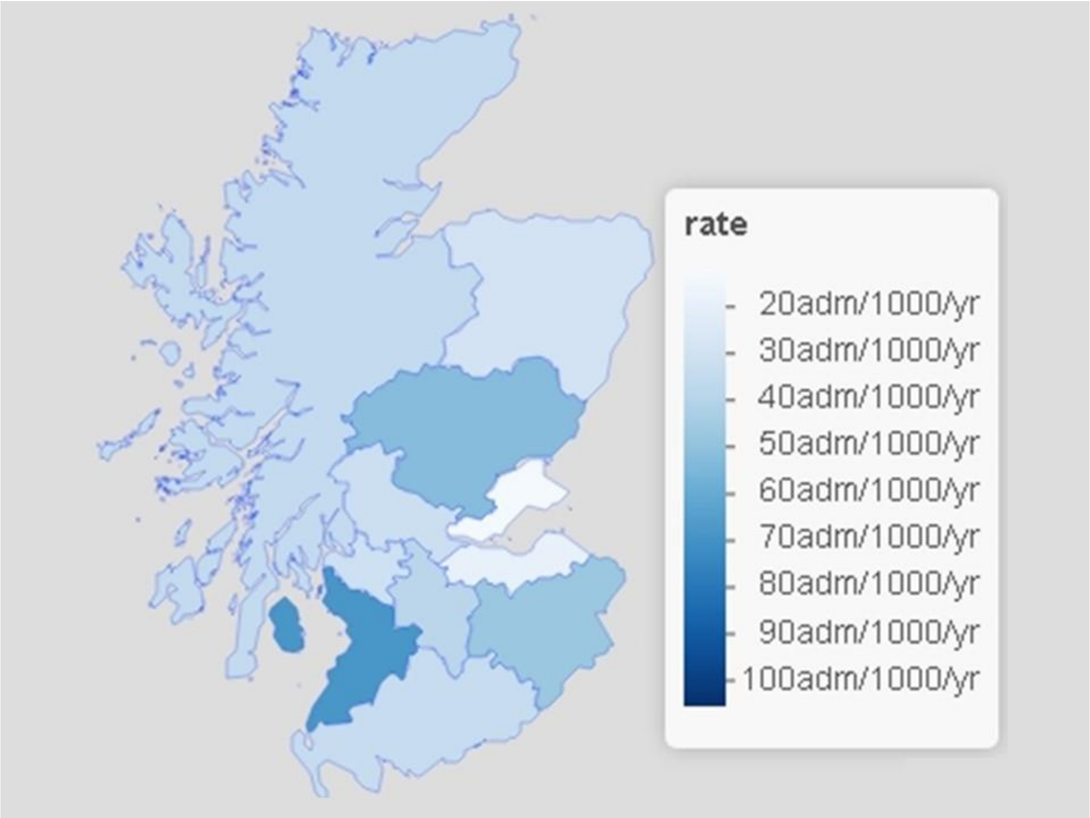

CHIEF SCIENTIST OFFICE

Quantitative Results

Lower than average form this source. Higher than average from this source

Zero day admissions	GP 8.9%	OOH 15.2%	ED 62.5%	Combo 13.4%
Asthma (n=2148)	5.7%	14.3%	68.7%	11.3%
Bronchiolitis (n=6295)	10.1%	18.1%	56.3%	15.4%
Croup (n=2799)	5.2%	14.1%	64.3%	16.4%
Afebrile convulsion (n=1344)	2.3%	2.1%	87.3%	8.3%
Febrile convulsion (n=976)	1.6%	2.2%	84.3%	11.9%
Chest infection (n=2552)	10.4%	20.3%	56.5%	12.8%
Tonsillitis (n=3706)	9.2%	21.0%	54.5%	15.4%
URTI (n=8165)	7.2%	18.5%	58.3%	16.0%
Viral infection (n=10,236)	7.8%	18.1%	58.7%	15.4%

CHIEF SCIENTIST OFFICE

Quantitative Results

Lower than average form this source. Higher than average from this source

Zero day admissions	GP	[illegible]	ED [illegible]	Combo 13.4%
Asthma (n=2148)	[illegible]	[illegible]	[illegible]	11.3%
Bronchiolitis (n=6295)	[illegible]	[illegible]	[illegible]	15.4%
Croup (n=2799)	[illegible]	[illegible]	[illegible]	16.4%
Afebrile convulsion (n=1344)	2.3%	[illegible]	[illegible]	8.3%
Febrile convulsion (n=976)	1.6%	2.2%	[illegible]	11.9%
Chest infection (n=2552)	10.4%	20.3%	56.5%	[illegible]
Tonsillitis (n=3706)	9.2%	21.0%	54.5%	15.4%
URTI (n=8165)	7.2%	18.5%	58.3%	16.0%
Viral infection (n=10,236)	7.8%	18.1%	58.7%	15.4%

All very interesting but what is the story behind the numbers?

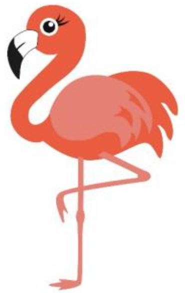

CHIEF SCIENTIST OFFICE

Quantitative Results

- Match cases and controls
- What “discretionary factors” differ
- Time of presentation (0-6am highest)
- Causation not proven!

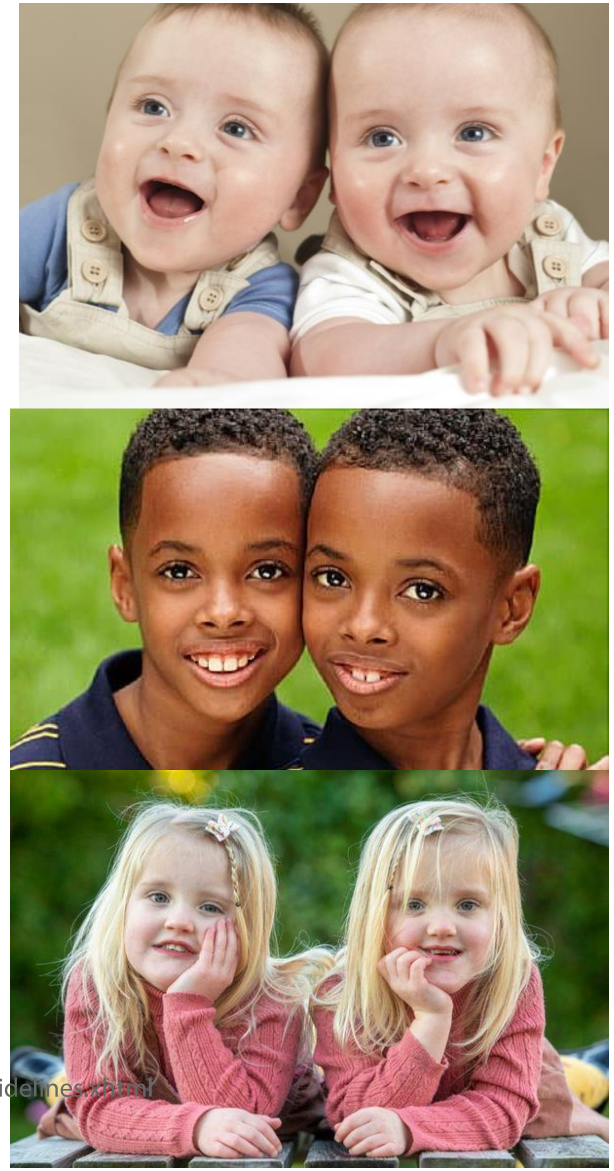

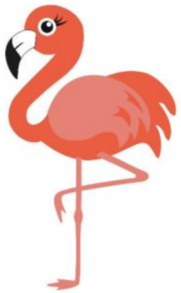

CHIEF SCIENTIST OFFICE

For peer review only - http://bmjopen.bmj.com/site/about/guidelines.xhtml

Quantitative Results

Summary

- There are streams within the flow
- Zero day admissions vary by
  - Referral source
  - Geography
  - Time of presentation
- Deprivation gradient as for all admissions
  - 30% vs 13%
- Time for some interviews!

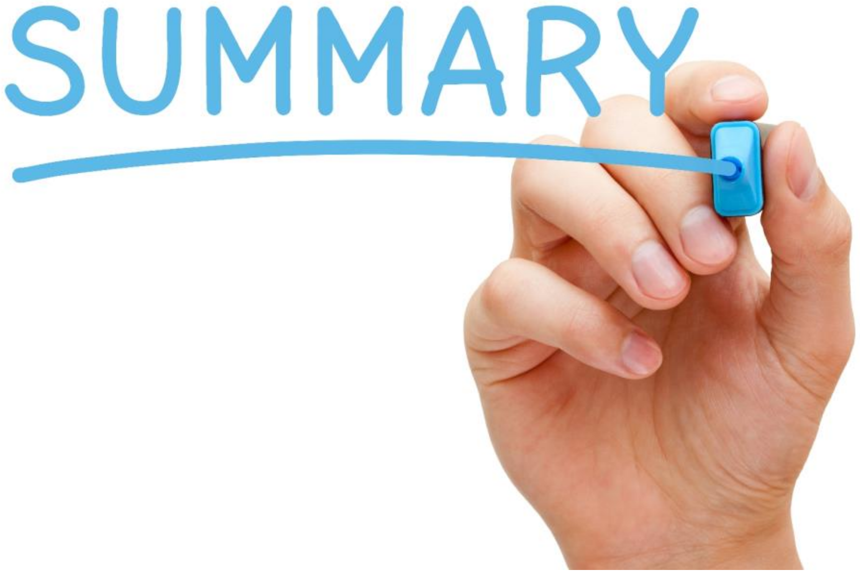

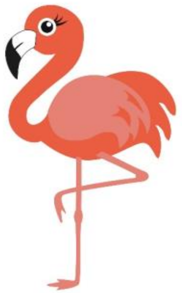

CHIEF SCIENTIST OFFICE

Research interview participants

Type of participant	GP	Nurse (community)	Doctor (hospital)	Consultant (hospital)	Nurse (hospital)	Families	Total
**No.**	10	5	7	10	16	21	**69**

CHIEF SCIENTIST OFFICE

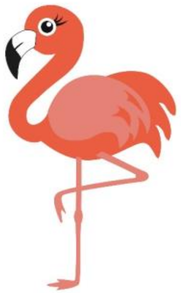

Recording admissions

Consultant

It gets terribly confusing - how do you actually count referrals, admissions, turnarounds whatever? The way our unit is set up, the assessment unit has got X beds in it and it is physically, geographically next door to the ward. And I think if, for whatever reason, the child is transferred through to the ward, then that becomes an admission. Otherwise it is not counted as an admission. The child will be sent through to the ward because there's no space in the assessment unit to let them sit for three or four hours but then they're sent home from the ward after another couple of hours. That still falls into your criteria of a one day admission or less than one day admission, but it wouldn't be counted as an admission on our numbers.

How are admissions recorded in different hospitals and how does this then impact on the quantitative data for zero day admissions?

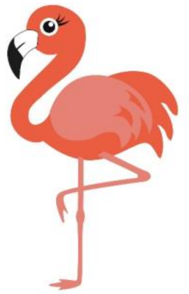

CHIEF SCIENTIST OFFICE

Children going to different areas/ health boards

Doctor

So even the ones that present at hospitals A and B - which is about a two, two-and-a-half hour drive away, ‘cause they're an adult hospital and got no paediatric services, so they see and treat children under the advice from us - … we can VC into them and help them, but if that child needs to be admitted then there's no services elsewhere for them, they have to come down here.

Parent

The medical team in hospital C debated sending Ella (fictitious name) to hospital D and I was surprised that the decision to send her to hospital D wasn’t made sooner. When the Senior Nurse came on shift, things started to move quicker and thankfully the decision was made to send us to hospital D. My husband and daughter left the hospital at 6pm to be taken to the air ambulance to fly to hospital D.

Potential impact on zero day numbers from children having to travel to specialist hospitals outside their own health board?

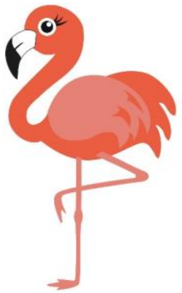

CHIEF SCIENTIST OFFICE

Resources: people and physical

Doctor

Overnight when a child is admitted we don’t have nursing staff for our observations unit so therefore the child goes straight to inpatients and is admitted as an inpatient overnight, ‘cause generally the majority of them that come in overnight stay for the morning anyway.

Doctor

If I wasn’t busy on an evening, a nightshift, I would wander down to the out-of-hours because it was on the same site and just said, ‘Well look, I'll just come down and see the child there.' And all they wanted was a bit of reassurance that what they were doing was correct and the child was okay.

How do resources impact on whether a child can be managed in that area, or on where a child is sent and therefore how an admission is recorded?

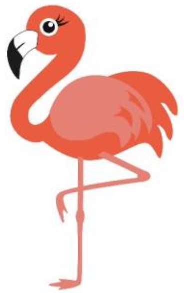

CHIEF SCIENTIST OFFICE

Policies and targets

Consultant

So all of the patients that come through our department are subject to the Scottish Government's unscheduled care four-hour target. So obviously that target does drive our practice and behaviour to a certain extent. We had set up an adjacent area and badged it as a clinical decision unit to essentially allow our department to continue to function with the volume of patients that we expected to allow patients to flow in and flow out of the back, while still obviously being able to meet that target.

GP

They have a rule in the ward in NHS X that they won't keep children longer than 24 hours, that’s their rule. If the child needs to stay in hospital beyond 24 hours the child needs to go to Hospital E.

How do NHS, health board, or hospital targets impact on decisions made about a child’s care?

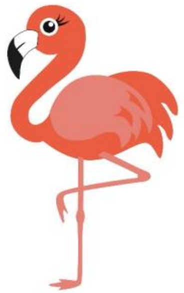

CHIEF SCIENTIST OFFICE

Physical access – primary care

Parent

Where we stay we're kind of out the way a wee bit, and they were saying it's taking ages to get an ambulance … I would've probably taken him in the car and just drove … but yeah public transport would be a no go.

GP

We are quite rural but I think it has an effect of families in two ways; one way the patient is reluctant to do that travel, they'd prefer to stay at home but also it actually means that sometimes we're taking on more risk because if things do go badly it takes longer to get someone into hospital as well.

How does physical access impact on decisions in primary care about whether to refer a child to hospital or treat them in the community?

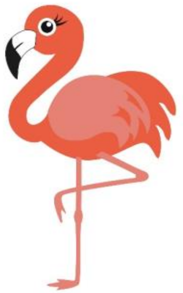

CHIEF SCIENTIST OFFICE

Physical access – secondary care

Consultant

I guess one thing with our geography, so the juniors, whenever they come and ask me advice about kids I'm always, like, 'where do they stay?' and they're, like, 'why does that matter?' but once you get to know our geography - so, for example, it can be over an hour to get back to the hospital, so I would be more likely to bring those kids in, particularly out-of-hours, if they’ve got croup or bronchiolitis or something, sometimes they'd get watched overnight, whereas if they were very close to the hospital you would send them home.

How does physical access impact on decisions made by hospital staff about whether a child is kept in or discharged home?

CHIEF SCIENTIST OFFICE

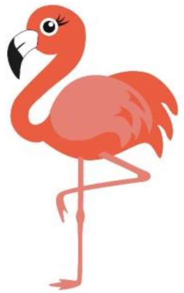

Social issues, including deprivation, and language issues

Consultant

Actually I will definitely be influenced by if they are going home to a dirty home. If they don't have a car then I'm not going to expect them to somehow come in on three buses to come and get an IV antibiotic 'cause it saves me a bed. It does influence your decision making.

Consultant

So there is a lot of deprivation but then you have pockets of really well off communities. So on the one hand you'd have the stereotype of the more deprived family maybe presenting more often, but then you've also got the more entitled wealthier people maybe feeling more entitled and therefore would they present more often.

How do social issues and deprivation/language issues impact on whether or not a child is admitted or discharged home?

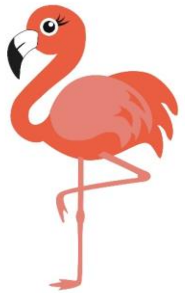

CHIEF SCIENTIST OFFICE

Expectations and experiences of services

Nurse

So I personally believe we're the victim of our own success and with what's happening out in the GP community and things like that, I think we can't turn back time. People do not feel safe and secure being managed out in the community and I think they feel it's safer that they're being assessed in the hospital and that's a feeling they get from the press and from GPs and things like that. I think we need to accept that this is our workload and we should get better at managing it.

Parent

If he was to get that breathless again I would probably phone an ambulance 'cause I probably didn't realise at the time how serious it was.... But we are only along the road so it wasn't as if we're miles away but I probably wouldn't phone 111 the next time, I would just take him in or phone an ambulance.

How and where do families seek healthcare advice and what do they expect from that?

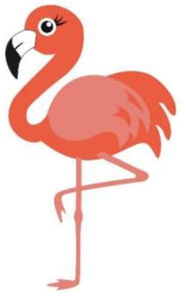

CHIEF SCIENTIST OFFICE

1495 UNIVERSITY OF ABERDEEN

nmahp-ru
Nursing, Midwifery and Allied Health Professions Research Unit
Improving health through research

NHS Grampian

UNIVERSITY of STIRLING

Edinburgh Napier UNIVERSITY

GIG CYMRU NHS WALES | Iechyd Cyhoeddus Cymru | Public Health Wales

Funded by: CHIEF SCIENTIST OFFICE

A few questions to consider….

1. **What is the most important thing you have heard this morning?**

2. **What surprised you most?**

3. **What did not surprise you?**

See you back here at 1.15 ☺

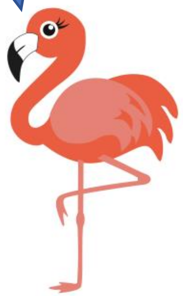

CHIEF SCIENTIST OFFICE

Welcome back

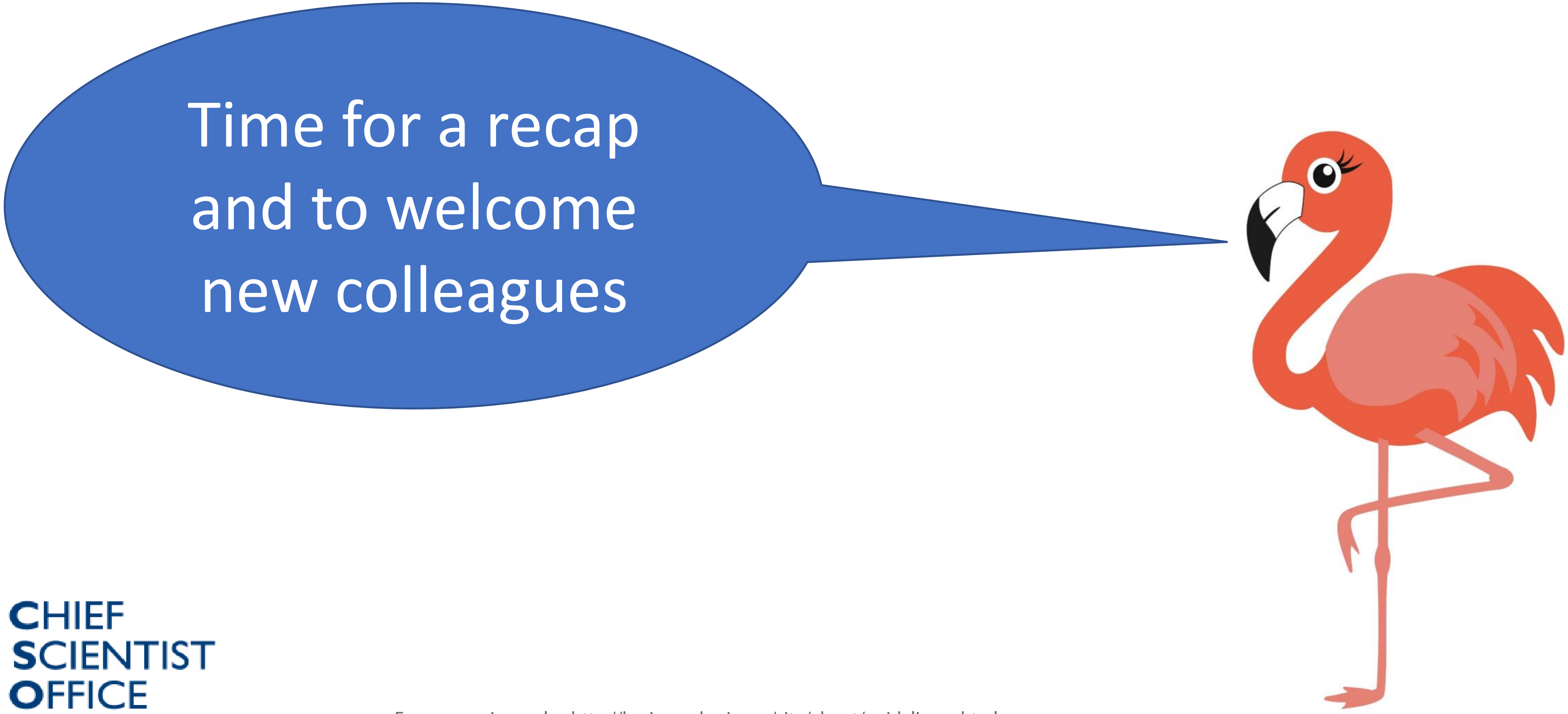

CHIEF SCIENTIST OFFICE

Why are we meeting?

This is stakeholder engagement

Two main aims

1. Share results with you
   - What surprises you
2. What are the priorities?
   - What would you do first?

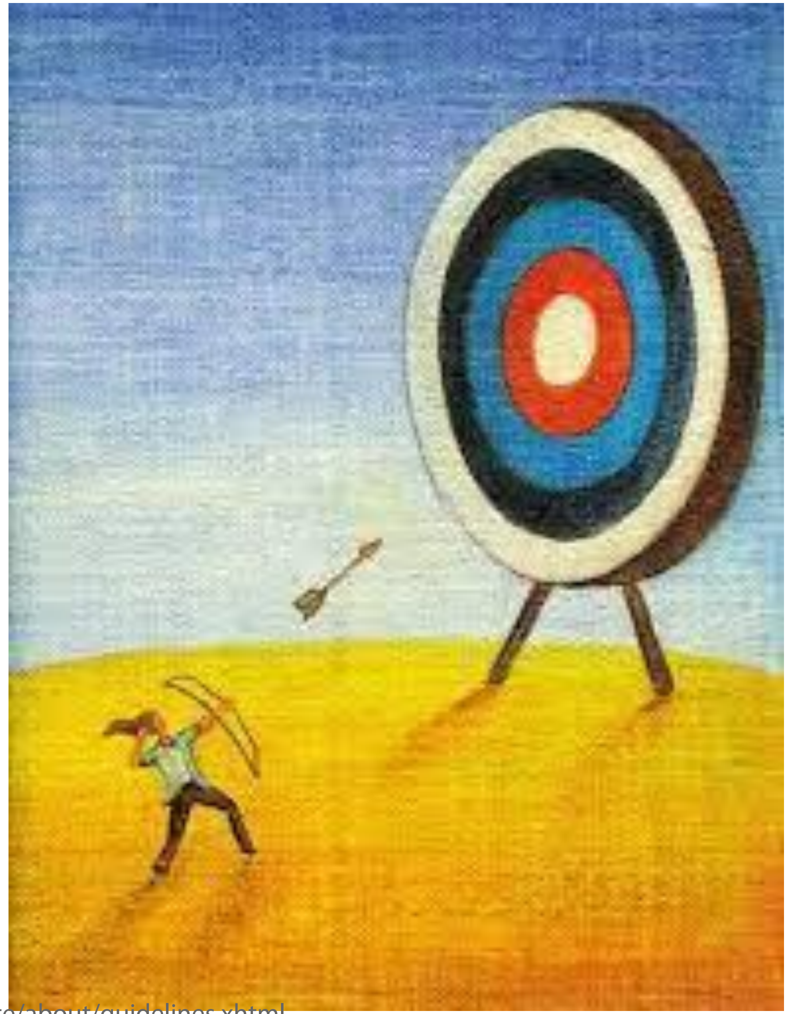

CHIEF SCIENTIST OFFICE

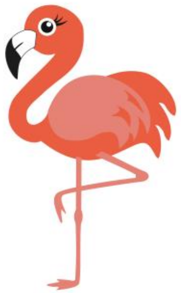

Introductions

- Who is here today?

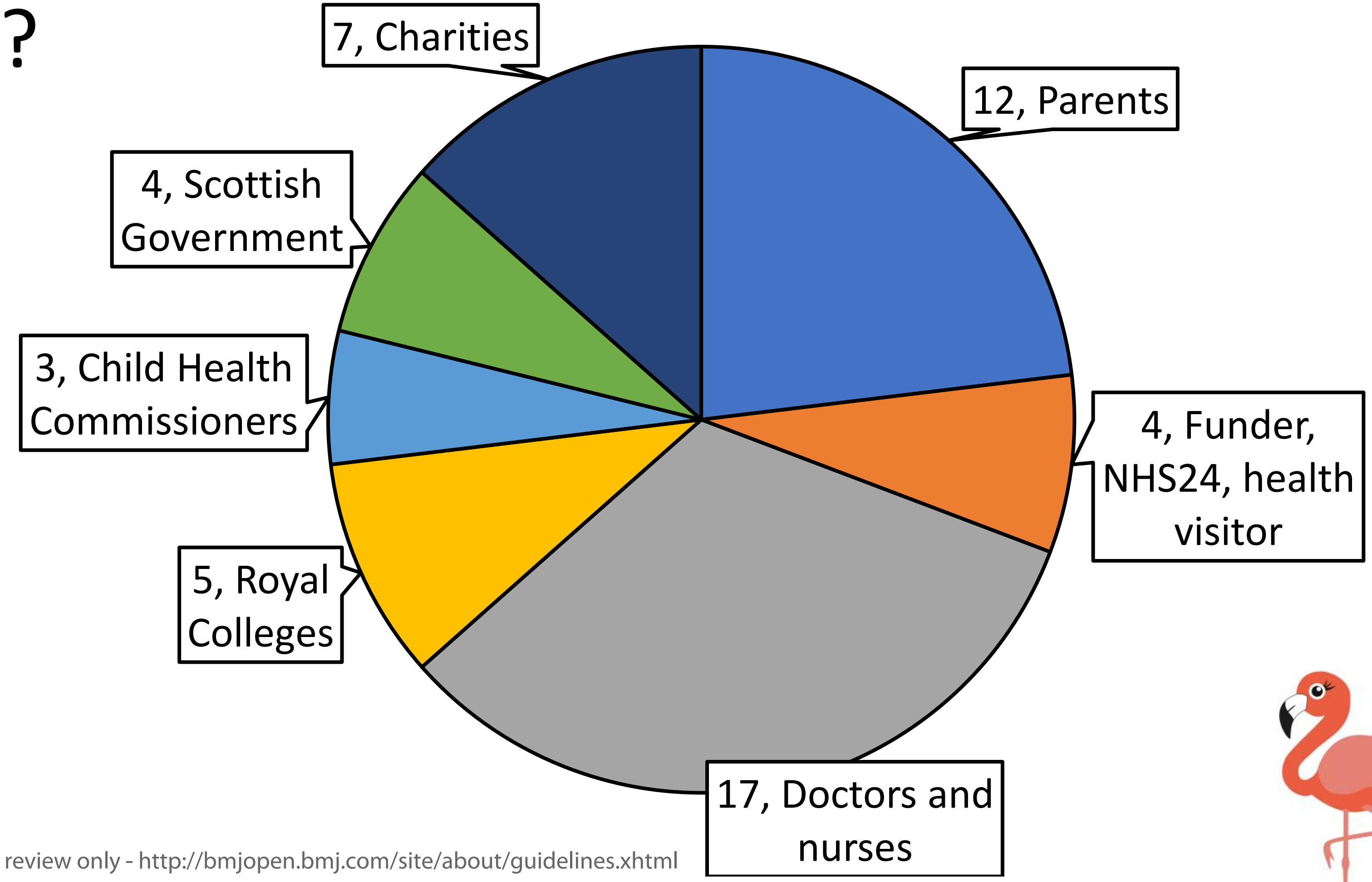

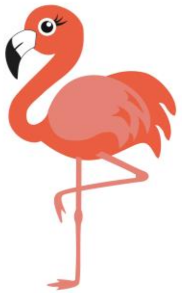

CHIEF SCIENTIST OFFICE

FLAMINGO

Flow of hospital admissions in children and young people

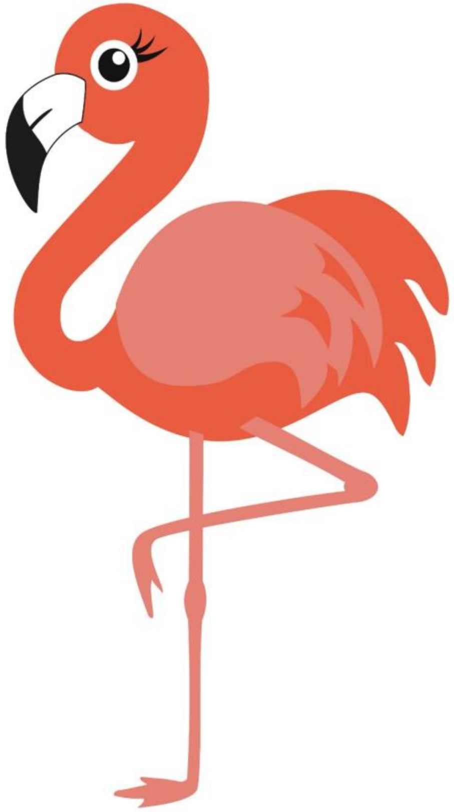

Potential for Innovation and Change

CHIEF SCIENTIST OFFICE

For peer review only - http://bmjopen.bmj.com/site/about/guidelines.xhtml

Practices & approaches tried & deemed successful by interviewees

- Access to specialist paediatric staff to inform decision-making, assessment & management of care for staff with less paediatric experience
- Change layout of Emergency Departments (EDs) to separate infectious & non-infectious patients
- Areas in ED where children can be observed for short periods
- 24-hour open access directly back to paediatric ward for patients following hospital discharge
- Dedicated specialist nursing teams for management of long-term conditions

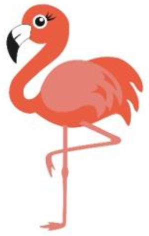

Two levels of change to consider

1. Quality Improvement initiatives within existing systems

- Quicker to implement
- Existing evidence for effectiveness
- 'Easy wins' around improving education & communication

2. System or Pathway changes

- Uncertain evidence for its effectiveness
- Required further research & development
- Take longer to implement
- Require significant resources

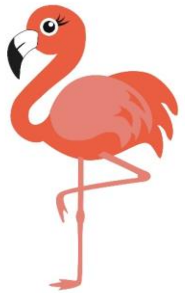

CHIEF SCIENTIST OFFICE

1. Suggestions for Quality Improvement

- More innovative educational resources – pictures, videos, practical education for common scenarios, websites/social media, including for non-English speakers
- Information sharing activities & social events alongside services where all attend e.g. immunisations
- Improve NHS secondary/primary care/education/social team communication to prevent children falling through the gap
- Access to clinical support for GPs from expert colleagues
- Rotation of GP and hospital trainees/junior staff
- Separate places for infectious/non-infectious admissions
- Fast-track appointment within one week as an alternative to admission
- Evaluate use of video conferencing for patient assessment and triage

CHIEF SCIENTIST OFFICE

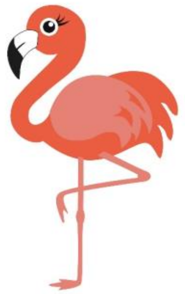

2. System or Pathway Changes

- Gap in acute paediatric nursing and medical skills in the community

- Assessment and observation in community settings
- Holistic children's hubs
- Hospital at Home model

- Care pathways for specific conditions

CHIEF SCIENTIST OFFICE

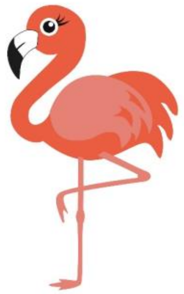

Gap in acute paediatric skills in the community

Hospital Nurse

It might be worth thinking about putting advanced paediatric nurse practitioners in GP surgeries. Again you've got well-experienced paediatric nurses that could go out into the community, see these patients, maybe be able to keep them at home by reassessing during the day, knowing what they're reassessing and also be able to do some teaching with the GPs. I think the way forward is maybe to try and put more paediatric experienced staff out in the community that can see acute unwell children.

What are the opportunities and challenges?

Would this be fair and sustainable for all patients?

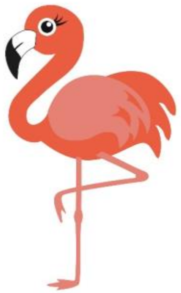

CHIEF SCIENTIST OFFICE

Assessment and observation in community settings

Hospital doctor

It'd be lovely to get more paediatric experience in the peripheries, in which case we might be able to reduce the number of patients that come down to us. And that’s for the parents' benefit as well and the family's benefit as well.

How do we get the right skills at the right time in the right place?

CHIEF SCIENTIST OFFICE

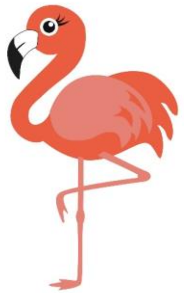

Holistic children's hubs linked to Children's Hospital

Hospital Doctor

What I find is that children are born and there's nothing else for children and families apart from a health visitor and, actually, it's almost like if we could have hubs and community hubs.

Hospital Doctor

Having a facility where you can sit and watch them, whether or not you actually do a specific intervention, even if you can just watch them and repeat their observations over an hour or two, we have found that that can help. We had a period of time where one of our consultants went out and spent time in the out-of-hours service and persuaded them to set aside an area in the out-of-hours building where children could wait and be observed. […]. You need space and you need the appropriate staff to look after them, you can't just sit them down and abandon them, so you need staff that are focused on acute assessment and acute treatment, you need a geographical location that allows you to do that.

Infection hubs? Assessment and observation hubs? Integrated holistic Physical, Mental and Social hubs? Range of staff and skills?

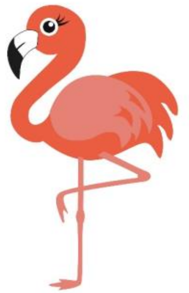

CHIEF SCIENTIST OFFICE

Hospital at Home model

Hospital doctor

So what we've found increasingly is that if there's no disease reason for the child being admitted it's actually do to with social support. And I think because we don’t have the community infrastructures in place to be able to say, ‘You were utterly distraught at two in the morning, we calmed you down, you went home and this person is going to come out to your house to see how you're doing in the day,' because we don’t have that level of sophistication, then often we use the hospital as a safety net.

Logistics of home visits - e.g. in rural areas / winter months / pandemics?

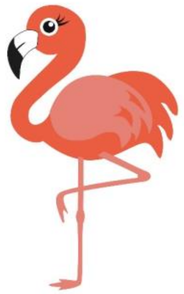

CHIEF SCIENTIST OFFICE

Specialist care pathways?

Parent

999 was the first call, just purely because it was a Sunday evening. Normally his diabetic nurse is absolutely amazing, so if that happened during the week we would phone her and get advice from her.

Parent

I really, really wasn't happy with the way that NHS111 call was handled. I immediately recognised she was having a seizure. The call handler basically made me feel like I was an overreacting mother. When the nurse phoned back was a bit more reassuring, but again, had gone with it being a viral response and I kept on saying 'I don't think it's a viral response.' I didn't even bother phoning 111 [the second time], I just took her straight up to A&E, but because everything was normal by the time we got there, again I was just sent home and kind of made to feel like I was a bit overreacting. So when she finally was seen and diagnosed by the clinician up at the hospital, who's a consultant, he was brilliant, he turned round and gave the diagnosis of it being epilepsy.

Suggested special pathways for admission
Early postnatal pathway for babies under 2 years? Wheezers? Bronchiolitis? Seizure?

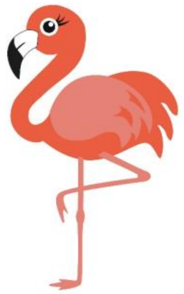

CHIEF SCIENTIST OFFICE

Is there an opportunity for a seizure pre-hospital care pathway?

A Case Study

CHIEF SCIENTIST OFFICE

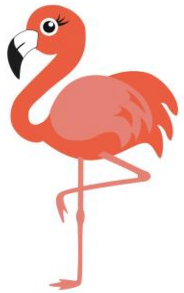

For peer review only - http://bmjopen.bmj.com/site/about/guidelines.xhtml

Characteristics of unscheduled paediatric hospital admissions for febrile and afebrile convulsions

Characteristic	Febrile Convulsion n (%)	Afebrile Convulsion n (%)
Unscheduled Admission	1916 (1.1)*	2661 (1.6)*
Zero Day Admission	976 (51.0)	1344 (51.0)
Sex:		
Female	424 (43.4)	633 (47.1)
Male	552 (56.6)	711 (52.9)
Age at Admission (years):		
< 1	96 (9.8)	131 (9.8)
1-4	818 (83.8)	499 (37.1)
5-9	54 (5.5)	387 (28.8)
10-15	5 (0.5)	323 (24.0)

* % of total emergency medical paediatric admissions to Scottish hospitals between 2015 and 2017 which was 171 039.

CHIEF SCIENTIST OFFICE

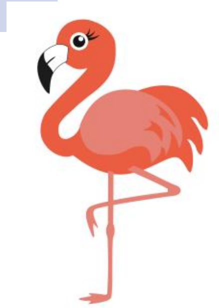

Interviews with health professionals and parents…

Health Care Professionals (HCPs)

- To explore their experiences of and decisions around pre-hospital referrals and subsequent ZDAs of children
- Range of HCPs including ED consultants, nurses, GPs (n=48)

Parents

- To explore their experiences of accessing hospital care resulting in a ZDA
- Range of experiences/conditions but only those focused on convulsions analysed for this presentation (n=4)

Epilepsy Specialist Nurses (ESNs)

- Specialist nursing service involved with and observers of a wide range of child and family experiences and scenarios – well placed to consider ZDAs for convulsions and suggest opportunities for change moving forward
- ESNs representing five Scottish HB areas (n=7)

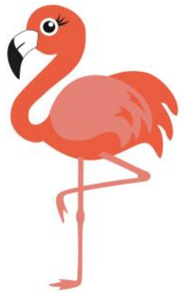

CHIEF SCIENTIST OFFICE

Findings from the interviews...

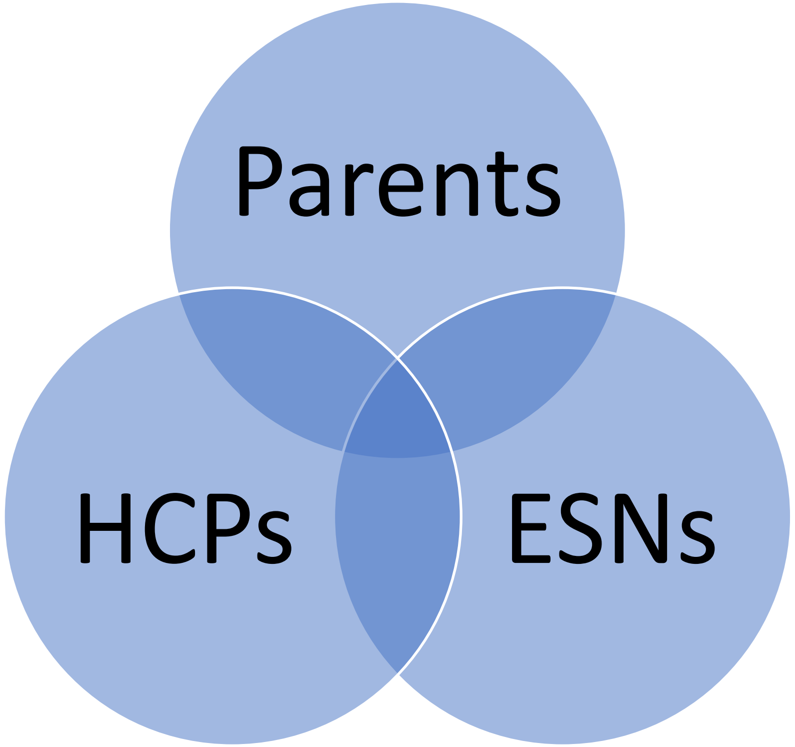

There was consistency and similarity in the themes emerging from interviews with these three different groups.

Analysis and synthesis of the interview data revealed three overarching themes related to experiences of unscheduled hospital attendance or admission of children with seizures

CHIEF SCIENTIST OFFICE

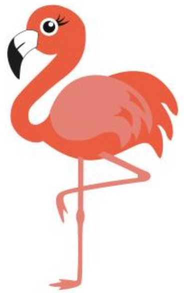

Overarching themes…

**Theme 1:** Anxiety and panic	• The overwhelming feelings of anxiety and panic experienced by parents when their child has a seizure and often contributes to hospital attendance or admission.
**Theme 2:** Reassurance, observation and forward planning	• Reassurance and ongoing support for parents through observation of their child following a seizure (by relevant HCP) and forward planning to diagnose new or manage existing seizures.
**Theme 3:** Care from the right person, in the right place, at the right time	• Care pathways for acute seizure care that ensures care is provided by a professional with knowledge and expertise of seizures in children, in the most appropriate setting and with timely access.

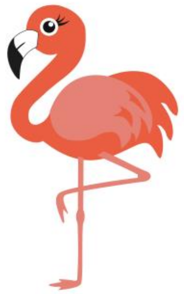

CHIEF SCIENTIST OFFICE

- Formally evaluate best practice around the use of video technology that was accelerated during the COVID-19 pandemic

- Explore the development of more child-oriented algorithms for NHS24 and ambulance service with additional education and training for these professionals.

- Raise awareness of febrile convulsions amongst parents, nurseries and schools, and the wider public.

- Expand the ESN service and nursing staff with expertise around acute seizure assessment and management.

CHIEF SCIENTIST OFFICE

For peer review only - http://bmjopen.bmj.com/site/about/guidelines.xhtml

Prioritising potential system innovations…

CHIEF SCIENTIST OFFICE

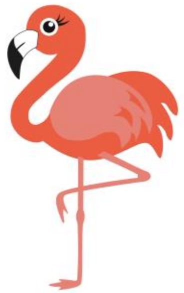

Prioritising potential system innovations…

- Address gap in acute paediatric skills of healthcare professionals in the community.
- Assessment and observation in community settings
- Holistic children’s hubs linked to Children’s Hospital
- Hospital at Home model
- Specific care pathways for under twos.
- Specific care pathways for wheezy children/bronchiolitis.
- Specific care pathway for seizure.

CHIEF SCIENTIST OFFICE

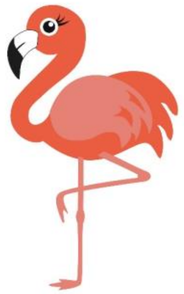

1495 UNIVERSITY OF ABERDEEN

nmahp-ru
Nursing, Midwifery and Allied Health Professions Research Unit
Improving health through research
CHIEF SCIENTIST OFFICE

NHS Grampian

UNIVERSITY of STIRLING

Edinburgh Napier UNIVERSITY

GIG CYMRU NHS WALES
Iechyd Cyhoeddus Cymru
Public Health Wales

Funded by: CHIEF SCIENTIST OFFICE

Questions to consider….

If you were in charge, what would you change first and why?

- Address gap in acute paediatric skills of healthcare professionals in the community
- Assessment and observation of patients in community settings
- Holistic children’s hubs linked to Children’s Hospital
- Hospital at Home model
- Specific Care Pathway for under 2 year olds
- Specific Care Pathway for wheezy children/bronchiolitis
- Specific Care Pathway for seizure
- Other ….. please state

Imagine you are in charge five year after your first choice has been in action. What problems would people come to you with about your “new” service?

See you back here at 2.45 ☺

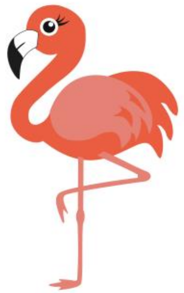

CHIEF SCIENTIST OFFICE

FLAMINGO
Flow of hospital admissions in children and young people – Wrap up

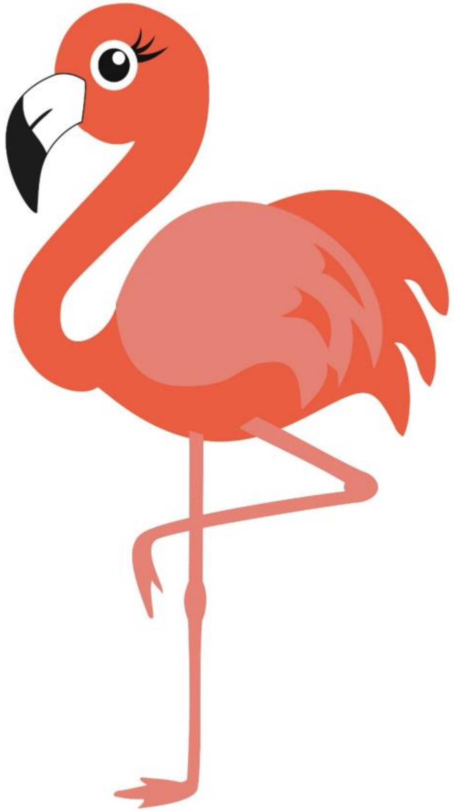

CHIEF SCIENTIST OFFICE

Why are we meeting?

This is stakeholder engagement

Two main aims

1. Share results with you
    - What surprises you
2. What are the priorities?
    - What would you do first?

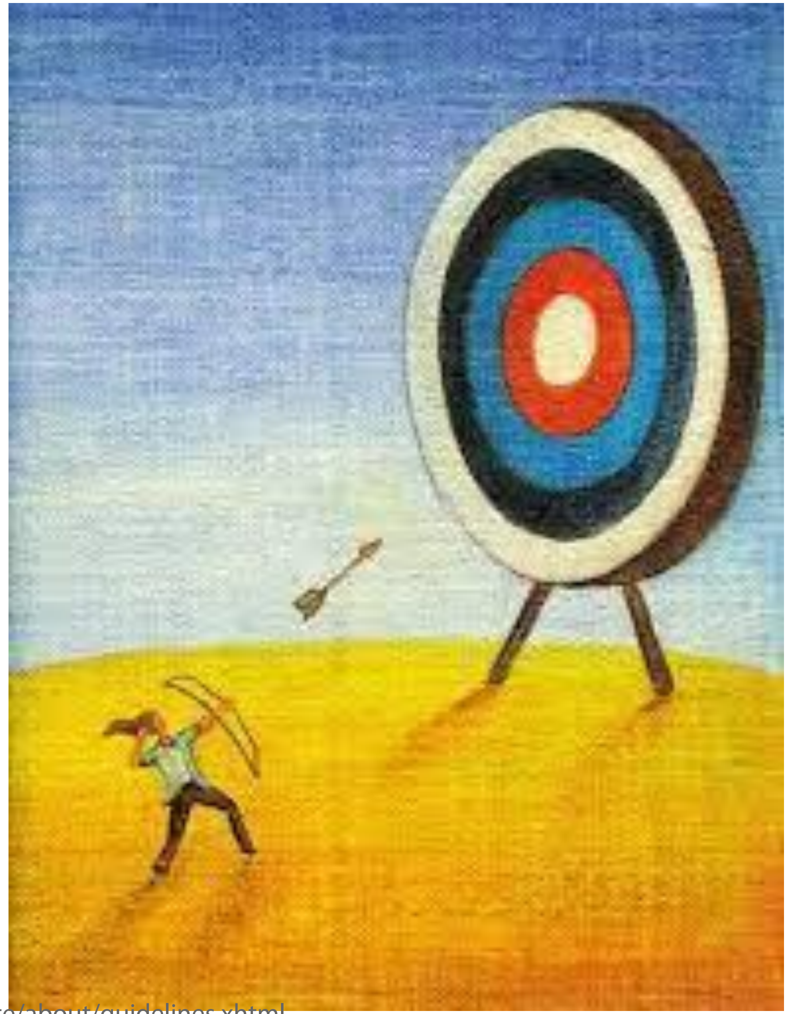

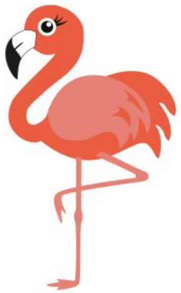

CHIEF SCIENTIST OFFICE

Why are we meeting?

Please can you find your piece of paper from this morning?

Did you meet that aim?

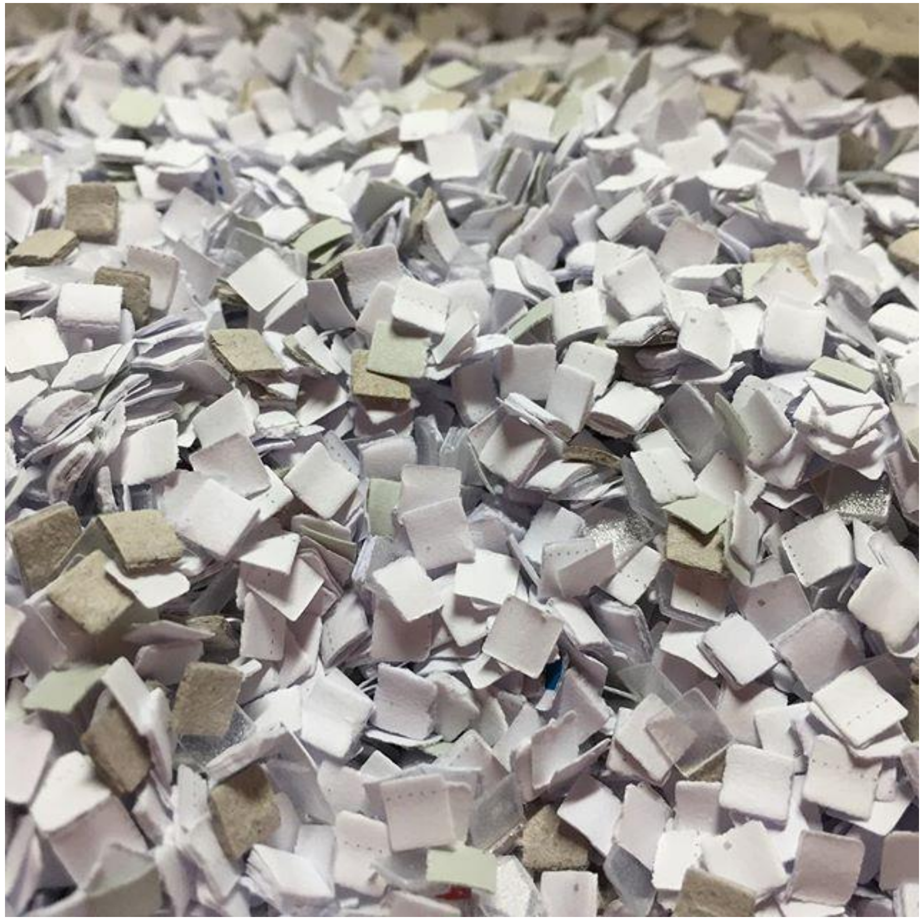

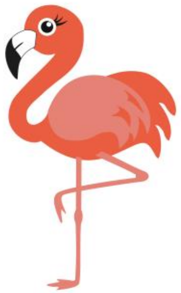

CHIEF SCIENTIST OFFICE

For peer review only - http://bmjopen.bmj.com/site/about/guidelines.xhtml

So where next?

- Finalise analysis
  - Quant
  - Qual
- Write papers
  - Overall findings and methodology
  - Regional variation
  - Seizures
  - Post discharge journey
  - Matched case-control

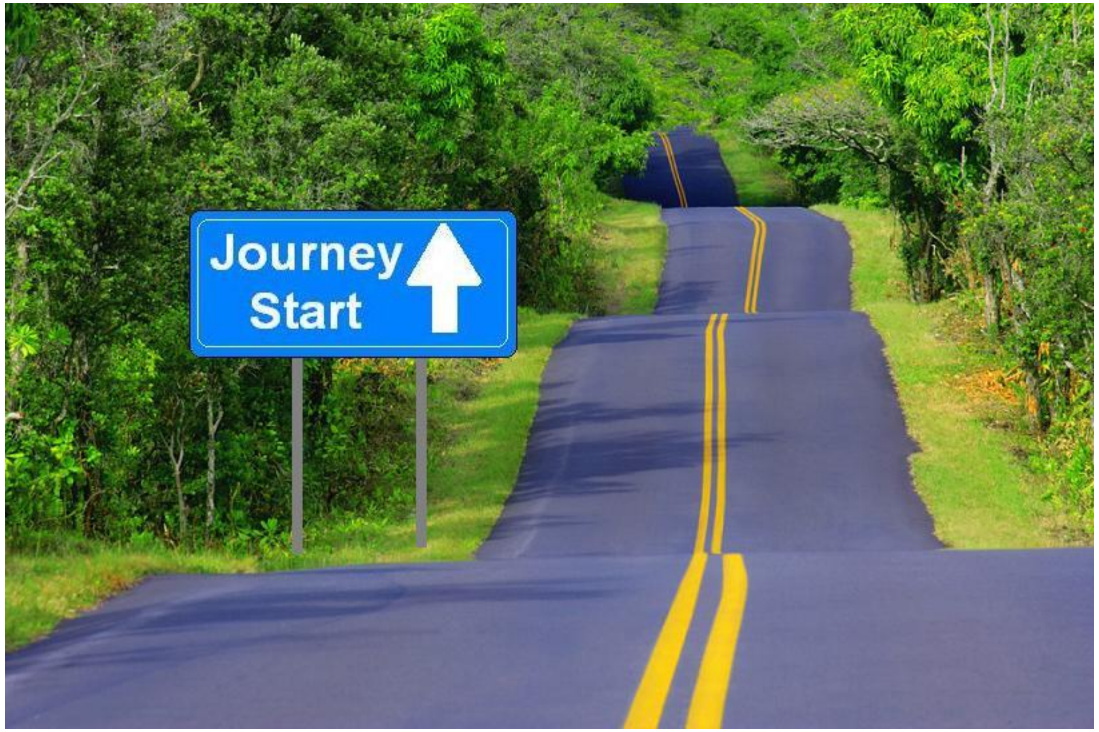

CHIEF SCIENTIST OFFICE

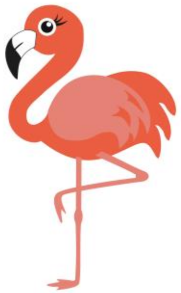

So where next?

Many potential ways to start

- Agreed referral pathways
- Rapid review clinics
- Communication
- More staff
- New buildings
- Etc

Original research

Interventions to reduce acute paediatric hospital admissions: a systematic review

Smita Dick,[1] Clare MacRae,[2] Claire McFaul,[1] Usman Rasul,[1] Philip Wilson,[3] Stephen W Turner[1]

What this study adds

- Since a review of this literature published in 2012, there has been a considerable increase in the number of publications describing interventions aimed at reducing unscheduled admissions.
- Despite these welcome additions to the literature, the evidence available lack robust studies (eg, randomised controlled trials) and is mostly based on single-centre experience.
- There is a need for multifaceted and multicentred interventions using resources from health (primary and secondary care) and social care to tackle the increasing admissions.

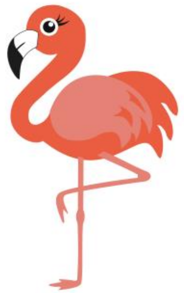

CHIEF SCIENTIST OFFICE

For peer review only - http://bmjopen.bmj.com/site/about/guidelines.xhtml

Thank you all very much

- Could not have done this without you
- Post meeting questionnaire on its way
- Have a fun weekend

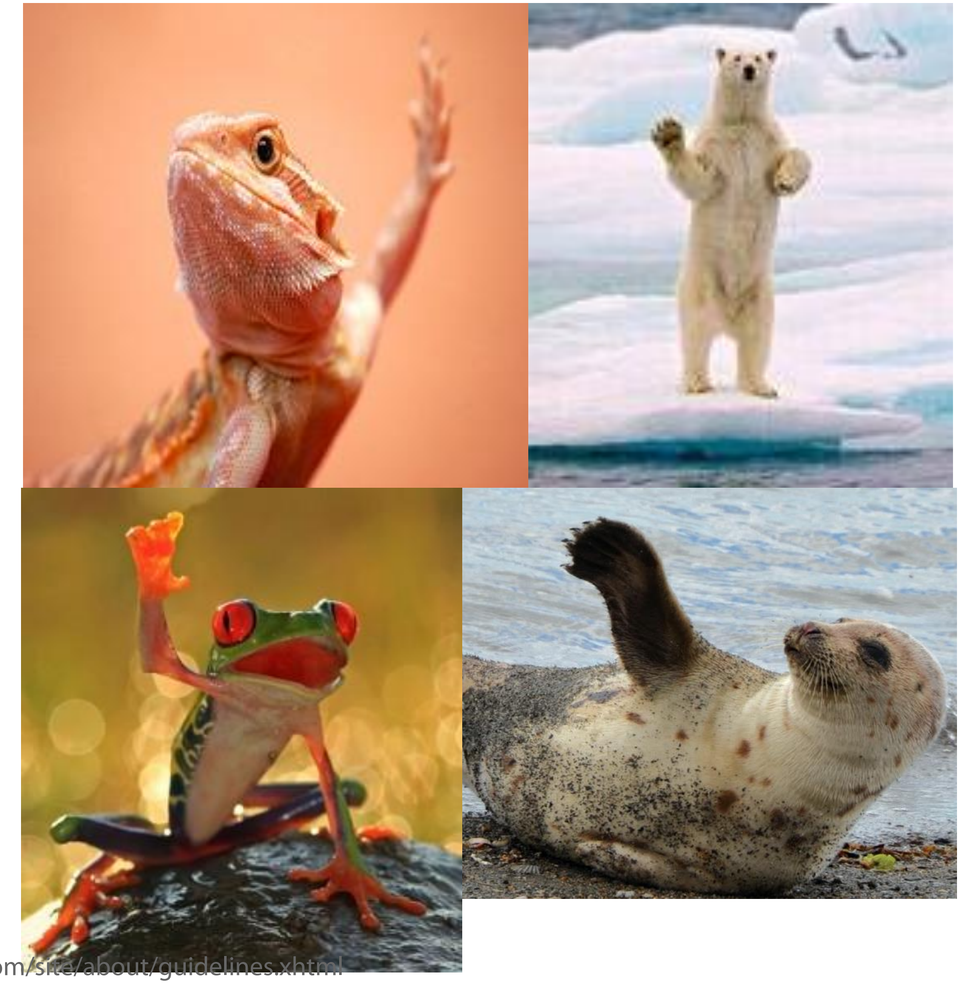

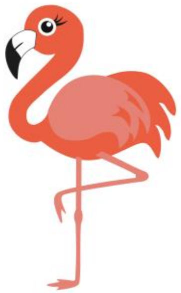

CHIEF SCIENTIST OFFICE

Supplementary file 7. Characteristics of qualitative interview participants

Full data available in: Malcolm C, King E, France E, et al. Short stay hospital admissions for an acutely unwell child: A qualitative study of outcomes that matter to parents and professionals. *PLoS One.* 2022 Dec 16;17(12):e0278777. doi: 10.1371/journal.pone.0278777. PMID: 36525432; PMCID: PMC9757586.

**Parents and children (n=21)**	
**Characteristic**	**Number (n)**
Parent's relationship to child:	
Mother	20
Father	1
Gender of child:	
Female	5
Male	16
Age of child at time of admission:	
0-5 years	16
6-10 years	4
11-16 years	1
Reason for admission (infection vs non-infection):	
Infection	15
Non-infection	6
Time elapsed since child's admission and the date of interview (months):	
0-24	17
>24	2
Missing data	2
Admission was pre-COVID (pre-March 2020):	
Yes	7
No	14
**Health Professionals (n=48)**	
**Role and Setting**	
**Primary Care/General Practice/OOH Service**	16
**Paediatric Hospital Emergency Department**	18
**District General Hospital Emergency Department**	14
Length of experience (years)	
<5	3
5-30	34
>30	4
Missing data	7

For peer review only

Full supporting interview quotations for each intervention

Addressing gaps in acute paediatric skills of health professionals working in community settings
**Increase specialist acute paediatric nursing roles**
It might be worth thinking about putting ANPPs in GP surgeries. Again, you've got well-experienced paediatric nurses that could go out into the community, see these patients, maybe be able to keep them at home by reassessing during the day, knowing what they're reassessing and also be able to do some teaching with the GPs. I think the way forward is maybe to try and put more paediatric experienced staff out in the community that can see acute unwell children. (C003_Nurse)
I think the emergency department was very busy, we were lucky to get some attention and we were moved fairly promptly down to the children's ward where we had…, it was clearly, [sic]. We were getting more specialist advice and you got much more attention down there, and they were able to fit the nebuliser properly onto my son's face which they hadn't really done in the ED. (Parent P002)
**Provide additional paediatric urgent care training for GPs**
Yeah, it's also unfortunate but true that paediatrics is not a requirement for training in general practice. So, you will unfortunately get, it's sad to say but the quality of referrals from primary care can be very poor. So, you have practitioners who are not prepared to take any chances themselves because of a lack of experience and this is a big problem for us in the winter months. (C014_Consultant)
I think training the GPs and the OOHs staff to have more experience and more confidence in paediatric problems should theoretically help but actually practically doing that is very, very difficult to get a significant amount of teaching and learning across to the body of individuals that work in primary care, so it's challenging, it's very difficult. (C005_Consultant)
I did quite a lot of paediatric training here, I didn't do any psychiatry […] but I managed to muddle through with psychiatry over the years but the paediatric training I felt was pretty essential […]. They [GP trainees] can breeze through without anything formally and paediatrics, you know, there's an enormous amount of pitfalls there with paediatrics. (C038_GP)

For peer review only

Then she [GP] went to do his SATS, so she had one of those little probes, sort of like a completely mobile standalone thing and he wriggled and fussed […] she did take it several times although my concern was it was an adult probe, so I was thinking it's probably not particularly accurate, but anyway she did try several times, she was only getting 86 and she did seem to have a good trace getting 86. […] So [GP] phoned [hospital] from the GP surgery and said 'I have this boy, he's known to you, I can only get SATS of 86, are you happy for him to come in?' So they said 'yes but he needs to come in an ambulance if his SATS are 86, he needs oxygen'. […] They [hospital] put a SATS monitor on him but for a small child and his SATS were like 99/100, so I think we were all like 'oh god, this seems such a waste of everyone's time and resources!' (Parent P010)
**Create rotational posts between primary and secondary care settings**
Part of my role when they took me on in post […] was going to be facilitation of learning and development of other people's skills. So, because COVID came in we like every other service had to set up a COVID assessment centre […]. So again, these were adult trained clinicians that came in and didn't have any paediatric experience, so they were heavily relying on GP support within the in-hours period if children were referred […]. So for a wee while as the schools and nurseries all started to go back we had a bit of a boom in paediatric presentations and I actually came out of my out of hours post for a period of four weeks to support in-hours so that there was somebody there that could see the children, so that there was somebody there that could if the nurse practitioners wanted to come in and shadow. I did a little bit of just ad hoc teaching there. […] So it is something that I have tried to look into and I am intermittently just trying to push on and do little bits of ad hoc education as and when I can but, like I say, unfortunately COVID impacted all of our plans for pushing forward with that. […]. I suppose I'm quite lucky that I have got established relationships with the paediatric ward because that's where I came from. So I do have an established support network if you like, and established relationships. (C027_Nurse)
Yeah I'm a GP […]I also am piloting at the moment a joint clinic with a couple of the consultant paediatricians […]. We have a community child health consultant once a month and a general medical consultant once a month that we co-consult with on some more kind of challenging cases to try and prevent access issues and things that can potentially be dealt with in primary care. […]. I also do out of hours, so occasionally work in Hospital6-1 as a GP, but seeing the stuff that gets triaged from Hospital6-1 as being more GP stuff, so you know, it's often viral things and things like that, so I do that as well. (C031_GP)
**Assessment and observation over time in community settings**
Sometimes very obviously the correct thing to do is not to discharge the child but to give a slightly longer period of observation. (C018_Consultant)

For peer review only

I think the main thing with a lot of these kids is actually just time and giving them a chance to let their anti-pyretic settle and giving them a bit of fluid and just a period of observation, which I think is the limiting factor in GP practices and in GP out of hours, you know, they just don't have the facilities to be able to watch these children for a period of time. (C015_Doctor)
They [children] can't tell you what's wrong, you know, so you might just have a baby that's a bit fractious or maybe not feeding as well, really soft signs, and I don't think we have the facilities even really to get all the accurate measurements that we would want, we don't have a paediatric SATS monitor, we can't do blood pressure in children either, so we're basing things more on their heart rate and their temperature and it's maybe kind of soft but just the general way they are. (C013_GP)
**Creation of holistic children's 'hubs'**
What I find is that then these children are born and there's nothing else for children and families apart from a health visitor and actually, you know, it's almost like if we could have hubs and community hubs where if you come in to get our health visiting weighed and things like that, you get taught about childhood diseases and when to worry, you know, and so almost like a mass public education programme that you get taught about when to worry, about when your child is sick. (C002_Consultant)
Interviewer: And how did you feel about taking him home [from hospital] at that point, did you feel fairly confident that you could look after, you know, if it was just bronchiolitis that you could look after that?<br>*Respondent: No, not at all [laugh] no I wasn't, no, I wasn't confident. I mean, he's a baby with the cold and I'll have to manage it but at that point he still wasn't feeding well, I probably wasn't very well at that point, yeah I was mentally not very well at that point and did not feel equipped to look after him at home, but then I guess I didn't know what else they could realistically do for him in hospital. So I was accepting of it.* [Parent P018]
Having a facility where you can sit and watch them, whether or not you actually do a specific intervention, even if you can just watch them and repeat their observations over an hour or two, we have found that that can help. We had a period of time where one of our consultants went out and spent time in the out-of-hours service and persuaded them to set aside an area in the out-of-hours building where children could wait and be observed. […]. You need space and

you need the appropriate staff to look after them, you can't just sit them down and abandon them, so you need staff that are focused on acute assessment and acute treatment, you need a geographical location that allows you to do that. (C005_Consultant)
**Hospital at Home model**
It's ebbs and flows and there are periods of times when you seem to get a number of referrals which you think, 'Surely that could be handled in the community, or can be managed in a different way rather than coming into hospital,' yeah. As to whether they could've handed in their urine sample of something, went away and then you can advise on what they're doing at home and representing, you know, safety-netting and so on. Or there are certain things where you think actually the best way to handle the particular scenario would've been to speak to someone who actually… rather than bring them in the night - and that happens fairly often, I would think, where a referral is received at five o'clock in the evening or something along those lines, and you know that they need some investigation or imaging that isn't going to happen that night or they're going to need to see, for instance, a child and adult mental health person, and that's not going to happen in the middle of the night - and you think, actually, that child could potentially be risk assessed and managed at home and then referred to the appropriate services the next day. (C020_Consultant)
The 111 nurse [NHS24]*, she was just [laugh], I think because I was obviously slightly panicked because he was bringing up foam and he was unable to breathe properly, so I was obviously like 'oh my gosh he's not breathing properly' and she was like 'calm down' and I was like 'aargh!' [laugh]. So I think, yeah, the 111 nurse [laugh] probably thought I was a completely over-anxious mum, but this was the worst that he's had the croup so I'd never seen him that poorly apart from when he was on neonatal, so I think it probably brought back some memories and I probably did panic. But the clinicians at the hospital were great, I mean, I explained to the doctor that I didn't want him catching anything and she was lovely and very understanding and said 'we'll try and send you home as soon as we can'.* (Parent P007)
**Extend specialised care pathways for sub-groups of children**
There are specialist nurses for most specialties, so there's specialist nurses for diabetes, specialist nurses for oncology, specialist nurse for cystic fibrosis, asthma, so […] different specialist nurses who are usually the first contact for such parents. […] So, yeah, these long-term conditions, we have changed completely the way we do it. (C010_Consultant)

For peer review only

Yeah, well again if they have a diagnosis of epilepsy and they've got a paediatric epilepsy nurse specialist in their area I think it's pretty straightforward for them, they have a clear plan of what to do and who to contact. As I say, my job is to keep them out of hospital so in between clinic appointments they would be phoning myself if they have a seizure, they're advised to phone myself, I'm a nurse prescriber so I change medications over the phone. A medication is never just prescribed without a clear pathway of how to increase it, when to increase it, what the maximum dose is […] so they [parents] always know that they can do something because it's mainly the helplessness that causes anxiety with epilepsy and the parents not being able to have control of the situation, whereas if you're giving them a wee bit of control of what to do and you give them permission to increase the medicine with a clear plan. I definitely find that cuts down the amount of phone calls you get, it cuts down the anxiety. (C041_Epilepsy Specialist Nurse)
Fevers in young children, vomiting in young children definitely are huge areas where we get lots and lots of referrals. Children under three months with a temperature, absolutely right to refer because it could be a septic baby but, like a two- or three-year-old who has had a temperature for 24 hours it's giving them the right advice by the GP that they can have Paracetamol and Ibuprofen […]. So I think fever in young children, vomiting in young children and respiratory illnesses. (C003_Nurse)

For peer review only

GUIDED – a guideline for reporting for intervention development studies.

Supplementary File 1: Blank Checklist

Item description	Explanation	Page in manuscript where item is located	Other*
1. Report the context for which the intervention was developed.	Understanding the context in which an intervention was developed informs readers about the suitability and transferability of the intervention to the context in which they are considering evaluating, adapting or using the intervention. Context here can include place, organisational and wider socio-political factors that may influence the development and/or delivery of the intervention (15).	4,5	
2. Report the purpose of the intervention development process.	Clearly describing the purpose of the intervention specifies what it sets out to achieve. The purpose may be informed by research priorities, for example those identified in systematic reviews, evidence gaps set out in practice guidance such as The National Institute for Health and Care Excellence or specific prioritisation exercises such as those undertaken with patients and practitioners through the James Lind Alliance.	4,5	
3. Report the target population for the intervention development process.	The target population is the population that will potentially benefit from the intervention – this may include patients, clinicians, and/or members of the public. If the target population is clearly described then readers will be able to understand the relevance of the intervention to their own research or practice. Health inequalities, gender and ethnicity are features of the target population that may be relevant to intervention development processes.	6-8	
4. Report how any published intervention development approach contributed to the development process	Many formal intervention development approaches exist and are used to guide the intervention development process (e.g. 6Squid (16) or The Person Based Approach to Intervention Development (17)). Where a formal intervention development approach is used, it is helpful to describe the process that was followed, including any deviations. More general approaches to intervention development also exist and have been categorised as follows (3):- Target Population-centred intervention development; evidence and theory-based intervention development; partnership intervention development; implementation-based intervention development; efficacy-based intervention development; step or phased-based intervention development; and intervention-specific intervention development (3). These approaches do not always have specific guidance that describe their use. Nevertheless, it is helpful to give a rich description of how any published approach was operationalised	6,7	
5. Report how evidence from different sources informed the intervention development process.	Intervention development is often based on published evidence and/or primary data that has been collected to inform the intervention development process. It is useful to describe and reference all forms of evidence and data that have informed the development of the intervention because evidence bases can change rapidly, and to explain the manner in which the evidence and/or data was used. Understanding what evidence was and was not available at the time of intervention development can help readers to assess transferability to their current situation.	8 +Table1	
6. Report how/if published theory informed the intervention development process.	Reporting whether and how theory informed the intervention development process aids the reader's understanding of the theoretical rationale that underpins the intervention. Though not mentioned in the e-Delphi or consensus meeting, it became increasingly apparent through the development of our guidance that this theory item could relate to either existing published theory or programme theory	6	
7. Report any use of components from an existing intervention in the current intervention development process.	Some interventions are developed with components that have been adopted from existing interventions. Clearly identifying components that have been adopted or adapted and acknowledging their original source helps the reader to understand and distinguish between the novel and adopted components of the new intervention.	7	
8. Report any guiding principles, people or factors that were prioritised when making decisions during the intervention development process.	Reporting any guiding principles that governed the development of the application helps the reader to understand the authors' reasoning behind the decisions that were made. These could include the examples of particular populations who views are being considered when designing the intervention, the modality that is viewed as being most appropriate, design features considered important for the target population, or the potential for the intervention to be scaled up.	8	

For peer review only - http://bmjopen.bmj.com/site/about/guidelines.xhtml

Duncan E, *et al. BMJ Open* 2020; 10:e033516. doi: 10.1136/bmjopen-2019-033516

Item description	Explanation	Page in manuscript where item is located	Other*
9. Report how stakeholders contributed to the intervention development process.	Potential stakeholders can include patient and community representatives, local and national policy makers, health care providers and those paying for or commissioning health care. Each of these groups may influence the intervention development process in different ways. Specifying how differing groups of stakeholders contributed to the intervention development process helps the reader to understand how stakeholders were involved and the degree of influence they had on the overall process. Further detail on how to integrate stakeholder contributions within intervention reporting are available (19).	5-6 +Table3	
10. Report how the intervention changed in content and format from the start of the intervention development process.	Intervention development is frequently an iterative process. The conclusion of the initial phase of intervention development does not necessarily mean that all uncertainties have been addressed. It is helpful to list remaining uncertainties such as the intervention intensity, mode of delivery, materials, procedures, or type of location that the intervention is most suitable for. This can guide other researchers to potential future areas of research and practitioners about uncertainties relevant to their healthcare context.	20	
11. Report any changes to interventions required or likely to be required for subgroups.	Specifying any changes that the intervention development team perceive are required for the intervention to be delivered or tailored to specific sub groups enables readers to understand the applicability of the intervention to their target population or context. These changes could include changes to personnel delivering the intervention, to the content of the intervention, or to the mode of delivery of the intervention.	21	
12. Report important uncertainties at the end of the intervention development process.	Intervention development is frequently an iterative process. The conclusion of the initial phase of intervention development does not necessarily mean that all uncertainties have been addressed. It is helpful to list remaining uncertainties such as the intervention intensity, mode of delivery, materials, procedures, or type of location that the intervention is most suitable for. This can guide other researchers to potential future areas of research and practitioners about uncertainties relevant to their healthcare context.	20	
13. Follow TIDieR guidance when describing the developed intervention.	Interventions have been poorly reported for a number of years. In response to this, internationally recognized guidance has been published to support the high quality reporting of health care? interventions[5]and public health interventions[14]. This guidance should therefore be followed when describing a developed intervention.	4 Followed INDEX	
14. Report the intervention development process in an open access format.	Unless reports of intervention development are available people considering using an intervention cannot understand the process that was undertaken and make a judgement about its appropriateness to their context. It also limits cumulative learning about intervention development methodology and observed consequences at later evaluation, translation and implementation stages. Reporting intervention development in an open access (Gold or Green) publishing format increases the accessibility and visibility of intervention development research and makes it more likely to be read and used. Potential platforms for open access publication of intervention development include open access journal publications, freely accessible funder reports or a study web-page that details the intervention development process.		

*e.g. if item is reported elsewhere, then the location of this information can be stated here.

For peer review only

For peer review only - http://bmjopen.bmj.com/site/about/guidelines.xhtml

Duncan E, et al. BMJ Open 2020; 10:e033516. doi: 10.1136/bmjopen-2019-033516

BMJ Open

Identifying and prioritising future interventions with stakeholders to improve paediatric urgent care pathways in Scotland, UK: a mixed-methods study

Journal:	*BMJ Open*
Manuscript ID	bmjopen-2023-074141.R1
Article Type:	Original research
Date Submitted by the Author:	18-Sep-2023
Complete List of Authors:	King, Emma; University of Stirling Department of Nursing and Midwifery,<br>France, Emma; University of Stirling, NMAHP Research Unit, School of Health Sciences<br>Malcolm, Cari; Edinburgh Napier University, School of Health and Social Care<br>Kumar, Simita; Public Health Scotland; Children's Health Scotland<br>Dick, Smita; University of Aberdeen, Child Health<br>Kyle, Richard G; University of Exeter<br>Wilson, Philip; University of Aberdeen, Institute of Health and Wellbeing<br>Aucott, Lorna; University of Aberdeen , Medical Statistics Team<br>Turner, Stephen; University of Aberdeen, Department of Child Health<br>Hoddinott, Pat; University of Stirling, Nursing, Midwifery and Allied Health Professional Research Unit
**Primary Subject Heading**:	Paediatrics
Secondary Subject Heading:	Emergency medicine, Paediatrics, Health services research
Keywords:	Patient Participation, Paediatric A&E and ambulatory care < PAEDIATRICS, QUALITATIVE RESEARCH, ACCIDENT & EMERGENCY MEDICINE

SCHOLARONE™
Manuscripts

I, the Submitting Author has the right to grant and does grant on behalf of all authors of the Work (as defined in the below author licence), an exclusive licence and/or a non-exclusive licence for contributions from authors who are: i) UK Crown employees; ii) where BMJ has agreed a CC-BY licence shall apply, and/or iii) in accordance with the terms applicable for US Federal Government officers or employees acting as part of their official duties; on a worldwide, perpetual, irrevocable, royalty-free basis to BMJ Publishing Group Ltd ("BMJ") its licensees and where the relevant Journal is co-owned by BMJ to the co-owners of the Journal, to publish the Work in this journal and any other BMJ products and to exploit all rights, as set out in our licence.

The Submitting Author accepts and understands that any supply made under these terms is made by BMJ to the Submitting Author unless you are acting as an employee on behalf of your employer or a postgraduate student of an affiliated institution which is paying any applicable article publishing charge ("APC") for Open Access articles. Where the Submitting Author wishes to make the Work available on an Open Access basis (and intends to pay the relevant APC), the terms of reuse of such Open Access shall be governed by a Creative Commons licence – details of these licences and which Creative Commons licence will apply to this Work are set out in our licence referred to above.

Other than as permitted in any relevant BMJ Author's Self Archiving Policies, I confirm this Work has not been accepted for publication elsewhere, is not being considered for publication elsewhere and does not duplicate material already published. I confirm all authors consent to publication of this Work and authorise the granting of this licence.

For peer review only

TITLE

IDENTIFYING AND PRIORITISING FUTURE INTERVENTIONS WITH STAKEHOLDERS TO IMPROVE PAEDIATRIC URGENT CARE PATHWAYS IN SCOTLAND, UK: A MIXED-METHODS STUDY

Authors:

E King[1](corresponding author), E France[1], C Malcolm[2], S Kumar[3], S Dick[4], R G Kyle[5], P Wilson[6], L Aucott[7] S Turner[4,8], P Hoddinott[1]

[1]Nursing, Midwifery and Allied Health Professions Research Unit, University of Stirling

[2] School of Health Sciences, University of Dundee

[3]Screening and Immunisation, Public Health Scotland

[4] Child Health, University of Aberdeen

[5] Academy of Nursing, Department of Health and Care Professions, Faculty of Health and Life Sciences, University of Exeter

[6] Centre for Rural Health, Institute of Applied Health Sciences, University of Aberdeen

[7] Centre for Randomised Healthcare Trials, University of Aberdeen

[8] Women and Children Division, NHS Grampian, Aberdeen

Corresponding author: emma.king@stir.ac.uk

KEY WORDS

Urgent care, paediatrics, Patient and Public Involvement, short stay admission, qualitative, mixed methods

For peer review only - http://bmjopen.bmj.com/site/about/guidelines.xhtml

ABSTRACT

Objectives

To identify, and prioritise interventions, from the perspectives of parents and health professionals, which may be alternatives to current unscheduled paediatric urgent care pathways.

Design

FLAMINGO (FLow of AdMissions in chIldren and youNG people) is a sequential mixed-methods study, with Public and Patient Involvement (PPI) throughout. Data linkage for urgent admissions and three referral sources: emergency department (ED), out of hours service (OOH), and general practice (GP), was followed by qualitative interviews with parents and professionals. Findings were presented and discussed at a stakeholder intervention prioritisation event.

Setting

National Health Service (NHS) in Scotland, UK.

Participants

Quantitative data: children with urgent medical admission to hospital from 2015-2017. Qualitative interviews**:** parents and health professionals with experiences of urgent short stay hospital admissions of children. PPI engagement was conducted with nine parent-toddler groups and a university-based PPI advisory group. Stakeholder event: parents, health professionals, and representatives from Scottish Government, academia, charities and PPI attended.

Results

Data for 171,039 admissions which included 92,229 short stay admissions (SSAs) were analysed and 48 health professionals and 21 parents were interviewed. The stakeholder event included 7 parents, 12 health professionals and 28 other stakeholders. Analysis and synthesis of all data identified seven interventions which were prioritised at the stakeholder event: 1) addressing gaps in acute paediatric skills of health professionals working in community settings; 2) assessment and observation of acutely unwell children in community settings; 3) creation of holistic children's 'hubs;' 4) adoption of 'hospital at home' models; and three specialised care pathways for sub-groups of children 5)convulsions, 6) being aged <2 years old, and 7) wheeze/bronchiolitis. 'Stakeholders prioritised interventions 1, 2 and 3; these could be

For peer review only - http://bmjopen.bmj.com/site/about/guidelines.xhtml

combined into a whole population intervention. Barriers to progressing these include resources, staffing and rurality.

Conclusions

Health professionals and families want future interventions that are patient-centred, community-based, and aligned to outcomes that matter to them.

STRENGTHS AND LIMITATIONS OF THIS STUDY

- Analysis of multiple sources of data from a robust mixed-methods study allowed us to identify interventions that stakeholders prioritised.
- Health professional contributions represented most professional groups providing care to acutely unwell children.
- Parents did not suggest specific interventions as solutions but shared their experiences and their values.
- Children's and paramedics' views are not represented.
- Solutions might differ for remote and rural communities and in a post-pandemic context.

For peer review only - http://bmjopen.bmj.com/site/about/guidelines.xhtml

INTRODUCTION

Unscheduled hospital admissions of children in the United Kingdom (UK) have increased steadily over past decades, largely due to a rise in urgent short stay admissions (SSAs), defined as a patient being admitted and discharged on the same calendar day [1, 2].. Since some parents cannot recall the precise time of admission and discharge, for qualitative sampling the definition of short stay admission used was parents recall of their child being admitted and discharged within 24hrs [3]. The rate of hospital medical admissions for children with acute illness in Scotland rose by 49% between 2000 and 2013, with SSAs rising by 186% from 8.6 to 24.6/1000 children per annum [2]. Children under two years of age account for the largest proportion of urgent SSAs [1], with upper and lower respiratory tract infections being a major reason for parents seeking an assessment [2]. Factors which influence decision making leading to an admission (other than the child's wellbeing) include staff shortages, workload pressures, bed shortages and for parents the distance to hospital, local pathways of care and the family's social circumstances [4], so changes to the present pathways of care are needed [5]. [6][7]

Two recent systematic reviews have found that there is a weak evidence base to inform interventions aimed at safely avoiding acute admissions; there was limited evidence supporting the use of telemedicine, reconfiguration of staff and short stay admission units[8, 9]. There is therefore a need for effective interventions to improve paediatric urgent care pathways to see if some admissions can be prevented[9] and improve family experiences [3, 4]. It is not known which parts of the pathway interventions should target, e.g. particular clinical settings or clinical presentations, or which interventions to develop [9, 10]. The identification and development of complex interventions needs to take account of, and build upon, existing evidence, and be conducted in collaboration with patient and public involvement (PPI) and other stakeholders [11, 12].

The FLAMINGO project (FLow of AdMissions in chIldren and youNG peOple) is a sequential three phase mixed-methods study to investigate the pathway leading to urgent SSAs in children incorporating PPI throughout[3]. Phase one, using linkage of national datasets, examined the pre-referral pathways for and characteristics of paediatric SSAs[13]. Phase two was informed by phase one findings and used qualitative interviews with parents and health professionals to explore contextual factors relating to SSAs, better understand referral pathways, and develop priorities for future interventions aimed at improving

For peer review only - http://bmjopen.bmj.com/site/about/guidelines.xhtml

unscheduled care pathways and the appropriateness of SSAs. In phase three, an engagement event attended by phase two participants and wider stakeholders was held to share project findings and debate and prioritise interventions identified during the interviews.

This paper focuses on identifying and prioritising, from the perspectives of parents and health professionals, interventions which may improve the efficacy of current unscheduled urgent care pathways for children for future research and development.

METHODS

Study design

FLAMINGO (FLow of AdMissioNs in children and younG peOple) is a three phase sequential mixed-methods study involving researchers from the NHS and various universities in the UK. The research team included a collaboration of experts in quantitative and qualitative methodology and clinical practice. This paper describes the identification of potential interventions, informed by the IdentifyiNg and assessing different approaches to Developing compleX interventions (INDEX) Approach (Co-author PH) [12] This systematic and consensus based intervention development guidance has not been previously applied to developing interventions aiming to improve paediatric care pathways for SSA.. It draws together findings from across the entire FLAMINGO study:

- PPI as a core component of the FLAMINGO project [14, 15].
- Phase 1: linked national datasets examining pre-referral pathways and characteristics of SSAs of children to Scottish hospitals. Methods are reported in supplementary file 1 and in Dick *et al.* [13]
- Phase 2: qualitative interviews with parents and health professionals providing insights into their values (including an important shared outcome of preserving the child's safety) [3] and experiences of unscheduled urgent care for children with SSAs and suggestions for change. Methods are reported in supplementary file 1 and in Malcolm *et al*. [3]
- In parallel a systematic review of hospital-based interventions to reduce acute paediatric admissions (co-authors SD, PW and ST) commenced at the start of the FLAMINGO study [9]. This

For peer review only - http://bmjopen.bmj.com/site/about/guidelines.xhtml

was used to help identify interventions, along with data from the quantitative and qualitative research and PPI input. A second systematic review of primary and community care interventions to reduce urgent paediatric admissions had commenced during Phase 2 but was not completed until after the intervention development work (co-authors SD, PW and ST) [8].

- Phase 3: a stakeholder event to discuss, debate and prioritise identified interventions for further research and development building on earlier PPI contributions.

FLAMINGO was undertaken in Scotland, where the NHS is organised into 14 geographically distinct Health Boards, each responsible for healthcare provision to their region's population. The project ran from January 2019 to December 2021 (Figure 1). The FLAMINGO team met monthly, the quantitative and qualitative sub-teams met separately in between.

Figure 1. Project timeline

Patient and Public Involvement

PPI was established at the outset (pre-COVID) to ensure the views of families were considered throughout all project stages. We involved nine parent-toddler groups and one university-based PPI advisory group attended by 112 adults and 107 children. Attendance at parent-toddler groups, including those accommodating people from lower socio-economic groups and minority ethnic backgrounds, and a university-based PPI advisory group, enabled parents to share their experiences of accessing healthcare and attending hospital for urgent healthcare. Their experiences informed the qualitative interview topic guide; ensured the materials used for recruitment were appropriate; and their experiences of urgent care supplemented the qualitative data to inform ideas for potential interventions. An independent PPI advisor critically reviewed and commented on manuscript drafts.

For peer review only - http://bmjopen.bmj.com/site/about/guidelines.xhtml

Intervention identification and development

Data Collection

The intervention development approach was target population-centred, incorporating front-line health professional and parent perspectives collected through PPI and qualitative interviews [12]. Potential future interventions were developed by the qualitative team [CM, EK, EF, PH] combining and interpreting FLAMINGO data, a systematic review [9], and the quantitative data showing the large contribution that wheeze/bronchitis and under 2's have to the number of paediatric SSAs.

Semi-structured interviews questions to health professionals asked about potential improvements to pathways for children between home and urgent SSAs; parents were asked about how their family's experiences could be improved in future. Interview topic guides are in Supplementary files 2 and 3 and Malcolm *et al.* [3]. Participant information sheets are included in Supplementary file 4. For identification of interventions to take forward for prioritisation, an intervention was defined as a change/innovation where there is equipoise, i.e. no evidence to support effectiveness, so it would need further research on acceptability, feasibility, effectiveness and cost-effectiveness before implementation.

A logic model was developed early in the study (Figure 2) informed by a Directed Acyclic Graph (DAG) model (a causal diagram) [16, 17]. The DAG was referred to iteratively throughout the FLAMINGO project and incorporated into a revised/refined logic model once the data analysis was complete (Supplementary file 5). The final logic model was informed by earlier FLAMINGO study qualitative analysis [3] about the shared outcomes of care that are important to both health professionals and parents that inform the design of a new care pathway: prioritising child safety; resolving uncertainty and anxiety about the illness trajectory; parents greatly valu timely access to care from experienced paediatric staff; and health professionals acknowledge the need to improve system pathways for pre-hospital care and support for families within the community.[3]

Figure 2: Original system/process logic model – the acutely sick child. May 2020

For peer review only - http://bmjopen.bmj.com/site/about/guidelines.xhtml

Data analysis

Interventions were identified from the qualitative interview data through the following steps, guided by framework analysis for applied policy research [18] applied in QSR International NVivo 12 software: familiarisation with transcripts; developing and agreeing a coding frame; indexing and further refinement of the coding framework; charting; and mapping and interpretation to search for patterns and explanations in the data. In-depth analysis was undertaken by EK and PH of potential solutions suggested by health professionals to improve urgent care pathways for children, any experiences of initiatives undertaken in their clinical settings, what had worked well and less well, and any consequences together with drawing on the collective experiences of their professions. Similarly, in-depth analysis was undertaken of parents' accounts through both PPI consultation and interviews about how their family's experiences could be improved in future. Families mainly voiced their problems and experiences, therefore possible solutions were implicit, whereas HCPs often suggested solutions explicitly. The team discussed the potential interventions generated through this process and decided whether each one met our intervention definition.

Reference was made to an underpinning systematic review of interventions to reduce acute paediatric hospital admissions, conducted by a FLAMINGO sub-team, to generate a final list of interventions for stakeholders to prioritise [9]. The review findings were shared with the FLAMINGO qualitative team (EK, CM, EF, PH) once qualitative data collection was complete (Table 1), although ST and PW contributed their knowledge gained from conducting the second systematic review in parallel with the qualitative data collection The review of mostly hospital-based/secondary care interventions in 28 studies [9] identified four groups of interventions: condition specific care pathways; staff reconfiguration; new building; and telemedicine. Telemedicine interventions were ruled out of our list of interventions to prioritise, based on the qualitative data from professionals and parents; there had been a huge shift to using video/telephone consultation during the COVID19 pandemic, therefore there was no longer equipoise. The review did not identify any additional interventions addressing the entire care pathway from home to hospital which showed sufficient promise to pursue in our prioritisation.

For peer review only - http://bmjopen.bmj.com/site/about/guidelines.xhtml

Intervention prioritisation

Data Collection

We held an interactive stakeholder event to discuss and prioritise potential interventions on 3 September 2021. Attendees could attend in person or by video conference and included parents, health professionals, and representatives from stakeholder organisations, e.g., charities and parent groups, government. Six discussion groups with attendees were facilitated and audio-recorded by a team member (SD, EF, EK, ST, CM, RGK, or PW) who also took written notes. Attendees individually ranked the priority of the interventions after the group discussions. Detailed methods are in Supplement 6.

Data analysis

Notes and transcripts from stakeholder group discussions were analysed in NVivo (by EF and EK) by coding data under 15 broad codes corresponding to confirming versus disconfirming perspectives for each of the interventions and a further code for other interventions suggested by attendees. Coded data were read repeatedly and themes identified. For the intervention priority ranking exercise, scores were summed and the average rank calculated for each of the identified interventions.

Ethics

The study was approved by the NHS North of Scotland Research Ethics Service (REC reference: 19/NS/0134). Participants in the qualitative interviews gave specific consent for their data to be used and anonymised quotes presented. Due to social distancing restrictions in place at the time written consent was replaced where possible by electronically signed consent forms and where families did not have the facilities to do this. All participants were provided with copies of the consent form by email in advance, with verbal recorded consent also given at the start of the interview. PPI and the stakeholder event did not require specific consent from individuals as they did not provide data. No voice recording was used for the PPI work. Those at the stakeholder event gave consent for voice recording for note taking purposes only, as such there are no quotes presented from the stakeholder event. Permissions for the routinely

For peer review only - http://bmjopen.bmj.com/site/about/guidelines.xhtml

collected data were given by the Public Benefit and Privacy Panel for Health and Social Care (reference 1718-0183). Data were analysed in the National Data Safe Haven of Scotland[13].

RESULTS

Intervention identification and development

The results report how different evidence sources informed a short-list of interventions for prioritisation at the stakeholder workshop. Data were available from 92,229 SSA admissions, which were linked to other databases [13], and interviews with 48 health professionals (including GPs, hospital doctors and consultants, community nurses, and hospital nurses) and 21 parents (including20 mothers and one father of a child who had had a SSA for acute illness). Characteristics of participants are provided in supplementary file 7 and elsewhere [3].

Identification of interventions

Analysis of all project data and a systematic review [9] identified seven potential interventions meeting our definition, four whole-system: 1) addressing gaps in acute paediatric skills of health professionals working in community settings, 2) assessment and observation of acutely unwell children in community settings, 3) creation of holistic children's 'hubs,' 4) adoption of 'hospital at home' models, and three sub-group interventions extending specialised care pathways for: 5)convulsions, 6) children age <2 years old, and 7) wheeze, cough, shortness of breath/bronchiolitis. Table 1 summarises FLAMINGO data linkage results and systematic review data that support each intervention.

For peer review only - http://bmjopen.bmj.com/site/about/guidelines.xhtml

Table 1. Summarised quantitative and review data that informed intervention selection, triangulated with the qualitative data, for the stakeholder event

Potential interventions identified from qualitative work	FLAMINGO Data linkage results [13]King et al. REGIONAL VARIATIONS IN SHORT STAY URGENT PAEDIATRIC HOSPITAL ADMISSIONS: A SEQUENTIAL MIXED METHODS APPROACH EXPLORING DIFFERENCES THROUGH DATA LINKAGE AND QUALITATIVE INTERVIEWS. In Press	Systematic review evidence [9] and other evidence sources.
Addressing gaps in acute paediatric skills of health professionals working inommunity settings	Admissions of children with respiratory infections dominated SSAs from all referral sources indicating the need to improve skills and confidence in management of respiratory infections. Asthma, bronchiolitis, croup, lower respiratory tract infection and cough/wheeze/shortness of breath accounted for a total of 17,764 SSA (19% all SSA)<br>Hypothesis: increasing paediatric skills of community staff in management of respiratory infections may benefit child outcomes and/or reduce SSA's	Not addressed by the review<br>Supported by other studies showing increasing proportion of SSA 2000-2013 [2]; a recognition that conditions may have been previously managed in the community [2, 5]; knowledge that ~70% of GPs have no post graduate paediatric training
Assessment and observation of acutely unwell children in community settings	N/A	The review focused on interventions in secondary care only
Creation of holistic children's 'hubs'	There were twice as many SSAs for children from the most deprived* compared to the least deprived communities (27% (n=25022) vs 13% (n=12032). Those referred by ED (n=29461) were over represented by children from white ethnic groups 72.5 % (n=21360) compared to 56% from GPs and 63% from out of hours .<br>Hypothesis: interventions in deprived communities may improve engagement with primary care; they may improve parent and child outcomes and/or reduce SSAs.	The review focused on interventions in secondary care only<br><br>Other evidence supports targeting more disadvantaged communities. [19, 20]

For peer review only - http://bmjopen.bmj.com/site/about/guidelines.xhtml

Adoption of 'hospital at home' models	Of all SSA's (n=92229) n=12378** readmissions within 30 days<br>Hypothesis: Hospital at home may improve parental and child outcomes; this may reduce the number of readmissions	The review focused on interventions delivered in secondary care only. There is some evidence to support this. [21]
Extending specialised care pathways for convulsions	Ten composite diagnoses accounted for 52% (n=47959) of SSA: asthma, bronchiolitis, convulsion (including febrile and afebrile convulsions), croup, gastroenteritis, upper respiratory tract infection (URTI), viral infection, tonsillitis, lower respiratory tract infections (LRTI) and admissions with a diagnosis of cough or wheeze or shortness of breath.<br>Convulsions are most commonly admitted directly from ED, rather than through OOH or GP referral.<br>Hypothesis: targeted interventions to the urgent care pathway that focus on specific diagnoses may improve parental and child outcomes and/or may reduce SSAs	Evidence from the review was that care pathways for specific conditions are instrumental in reducing admissions especially when they are standardised.<br>There was considerable heterogeneity between the studies and no randomised controlled trials were included. Publication bias was noted
Extending specialised care pathways for children age <2 years old	Most SSAs were for children under two years- of the 92,229 children with an SSA, 44,063 (48%) were <2 years, 28,306 (31%) were <1. The median age for SSA was 2.2 years (interquartile values 0.74-5.8).	Not included in the review<br>Supported by other evidence.[22]
Extending specialised care pathways for wheeze/bronchiolitis	Prevalence of respiratory symptoms (15.6% of SSA were due to asthma, bronchiolitis and cough, wheeze and shortness of breath)	As described above

* Using Scottish Index of Multiple Deprivation (SIMD) for postcode of reported https://www.gov.scot/collections/scottish-index-of-multiple-deprivation-2020/

** not previously reported

For peer review only - http://bmjopen.bmj.com/site/about/guidelines.xhtml

The first four suggested interventions align with reports published by Scottish Government since the early 1970s which advocate care in the community [23-25]. The perspectives of professionals, parents and PPI supporting the interventions are described with selected illustrative quotations in Table 2 and further quotations in Supplementary File 8.

Table 2 Selected supporting interview quotations for each intervention

**Addressing gaps in acute paediatric skills of health professionals working in community settings**
**Increase specialist acute paediatric nursing roles**
It might be worth thinking about putting ANPPs in GP surgeries. Again, you've got well-experienced paediatric nurses that could go out into the community, see these patients, maybe be able to keep them at home by reassessing during the day, knowing what they're reassessing and also be able to do some teaching with the GPs. I think the way forward is maybe to try and put more paediatric experienced staff out in the community that can see acute unwell children. (C003_Nurse)
**Provide additional paediatric urgent care training for GPs**
Yeah, it's also unfortunate but true that paediatrics is not a requirement for training in general practice. So, you will unfortunately get, it's sad to say but the quality of referrals from primary care can be very poor. So, you have practitioners who are not prepared to take any chances themselves because of a lack of experience and this is a big problem for us in the winter months. (C014_Consultant)
Then she [GP] went to do his SATS, so she had one of those little probes, sort of like a completely mobile standalone thing and he wriggled and fussed […] she did take it several times although my concern was it was an adult probe, so I was thinking it's probably not particularly accurate, but anyway she did try several times, she was only getting 86. They [hospital] put a SATS monitor on him but for a small child and his SATS were like 99/100, so I think we were all like 'oh god, this seems such a waste of everyone's time and resources!' (Parent P010)
**Create rotational posts between primary and secondary care settings**
Part of my role when they took me on in post […] was going to be facilitation of learning and development of other people's skills. So, because COVID came in we like every other service had to set up a COVID assessment centre […] So for a wee while as the schools and nurseries all started to go back we had a bit of a boom in paediatric presentations and I actually came out of my out of hours post for a period of four weeks to support in-hours so that there was somebody there that could see the children, so that there was somebody there that could if the nurse practitioners wanted to come in and shadow. [..] I suppose I'm quite lucky that I have got established relationships with the paediatric ward because that's where I came from. (C027_Nurse)

For peer review only - http://bmjopen.bmj.com/site/about/guidelines.xhtml

Yeah I'm a GP […]I also am piloting at the moment a joint clinic with a couple of the consultant paediatricians […]. We have a community child health consultant once a month and a general medical consultant once a month that we co-consult with on some more kind of challenging cases to try and prevent access issues and things that can potentially be dealt with in primary care. […]. (C031_GP)
**Assessment and observation over time in community settings**
I think the main thing with a lot of these kids is actually just time and giving them a chance to let their anti-pyretic settle and giving them a bit of fluid and just a period of observation, which I think is the limiting factor in GP practices and in GP out of hours, you know, they just don't have the facilities to be able to watch these children for a period of time. (C015_Doctor)
**Creation of holistic children's 'hubs'**
What I find is that then these children are born and there's nothing else for children and families apart from a health visitor and actually, you know, it's almost like if we could have hubs and community hubs where if you come in to get our health visiting weighed and things like that, you get taught about childhood diseases and when to worry, you know, and so almost like a mass public education programme that you get taught about when to worry, about when your child is sick. (C002_Consultant)
**Hospital at Home model**
It's ebbs and flows and there are periods of times when you seem to get a number of referrals which you think, 'Surely that could be handled in the community, or can be managed in a different way rather than coming into hospital,' yeah. As to whether they could've handed in their urine sample of something, went away and then you can advise on what they're doing at home and representing, you know, safety-netting and so on. Or there are certain things where you think actually the best way to handle the particular scenario would've been to speak to someone who actually..[…] where a referral is received at five o'clock in the evening or something along those lines, and you know that they need some investigation or imaging that isn't going to happen that night […], actually, that child could potentially be risk assessed and managed at home and then referred to the appropriate services the next day. (C020_Consultant)
**Extend specialised care pathways for sub-groups of children**
If they have a diagnosis of epilepsy and they've got a paediatric epilepsy nurse specialist in their area I think it's pretty straightforward for them, they have a clear plan of what to do and who to contact. As I say, my job is to keep them out of hospital so in between clinic appointments they would be phoning myself if they have a seizure, they're advised to phone myself […] (C041_Epilepsy Specialist Nurse)

For peer review only - http://bmjopen.bmj.com/site/about/guidelines.xhtml

Addressing gaps in acute paediatric skills of health professionals working in community settings

Health professional interviewees described how staff working in the community often lack the necessary training and experience to manage acute paediatric conditions which could contribute to the increasing referrals of children to hospital. Three potential changes to the provision of care were identified: i) increase specialist acute paediatric nursing roles, ii) provide additional paediatric training for GPs, and iii) create rotational posts between primary and secondary care settings.

Increase specialist acute paediatric nursing roles

Professionals and parents perceive an assessment by a health professional with acute paediatric skills, at an early stage in the acutely unwell child's pathway, as beneficial. Parents see specialist children's hospitals with a dedicated paediatric ED as a key advantage, with some criticising the lack of paediatric expertise in a general hospital ED. Health professionals recognise the potential benefits of increasing Advanced Nurse Practitioners with specialist paediatric clinical assessment skills (ANPPs), within community settings and EDs not located in dedicated children's hospitals. ANPPs require the autonomy to act as senior decision makers working with general practices may facilitate the management of acutely unwell children at home safely and with appropriate referral to other services.

Provide additional paediatric urgent care training for GPs

Paediatric training is not compulsory for GPs and expertise can vary across GPs and primary care staff A knock-on effect is that newly-qualified GPs may be trained by GPs who have limited or no formal paediatric qualifications. Health professionals based in hospitals perceived that some children were referred to ED because GPs lacked the experience or confidence to provide care in the community. GPs who had undertaken additional paediatric training acknowledged they had foregone training in other areas, such as care of the elderly or mental health.

Parental perspectives focused on comparing the reassurance and expertise they felt when encountering staff in dedicated paediatric settings and on the barriers that some encountered in gaining this in the community.

GPs with experience and skills in acute paediatrics may be able to improve access, triage and appropriate referral of acutely unwell children in the community. Additional training for GPs may be feasible within

For peer review only - http://bmjopen.bmj.com/site/about/guidelines.xhtml

the current UK three-year GP training prior to certification to practice. In the longer term this may improve urgent care pathways and reduce attendances at ED and/or SSAs.

Create rotational posts between primary and secondary care settings

Health professionals indicate that staff rotating between primary and secondary care can build capacity, capability, knowledge, experience of illness trajectories and relationships. Hospital staff gain greater awareness of issues such as the time pressures of 10-minute appointment schedules in general practice, and the concerns when a hospital paediatric service is hours away. Skills development is important, for example, ANPPs from hospital working in primary care can support development of acute paediatric assessment skills amongst primary care staff.

Some health professionals identified gaps in the primary care workforce as a key issue for timely access to appropriate care, for example where unfilled GP shifts in OOH has led to increased ED workload. Whilst rotating staff between primary and secondary care may improve urgent care pathway outcomes, in the current staff resource context the feasibility of this is uncertain.

Assessment and observation over time in community settings

A key outcome desired by parents and health professionals is to reduce uncertainty about the illness trajectory; this involves having time, space, and staff to effectively assess and observe an unwell child [3]. However, hospital health professionals noted the lack of appropriate spaces for observation and availability of appropriately skilled staff to perform it in the community.

Health professionals reported a large proportion of SSAs to hospital were the result of children requiring an extended period of assessment and observation to inform clinical decision making and safe discharge of the child home: e.g. responses to a fluid challenge in the vomiting child, or to antipyretics in the febrile child.

Parents prioritised timely assessment of their acutely unwell child by a health professional, therefore, few deemed hospital attendances to be inconvenient. PPI views were largely consistent; however, parents did consider general ED departments, where both children and adults are waiting to be seen, as an inappropriate setting for children. The challenges, as reiterated by health professionals for providing child-friendly community settings timely assessment and extended observation are: community paediatric skills gapsand the current lack of infrastructure, equipment, and suitable places, and the current NHS staff shortages.

For peer review only - http://bmjopen.bmj.com/site/about/guidelines.xhtml

The key overarching outcome of importance to both health professionals and families is safety of the child; parents value easy, direct access to urgent care for expert assessment and treatment [3]. Therefore, if community services were able to provide easy-access to staff with paediatric expertise and resources for timely assessment and observation, parents may be more likely to use community services than ED, with the potential to reduce SSAs to hospital.

Creation of holistic children's 'hubs'

PPI indicated that parents have difficulties taking their child to the GP due to busy services and parental work. Parents in low paid or unstable work lose money taking time off to attend appointments and instead attend pharmacies and ED.

Health professionals see holistic children's 'hubs' as an alternative to increasing paediatric services within existing primary care systems, although significantly more resources and personnel would be required. They described potential hubs as providing a 'one stop shop' for children's health and psychosocial needs, such as health education, routine appointments, community paediatric appointments, social work advice, and mental health services. Parents called for more tailored holistic care for both children and parents. Hubs could help by shifting more resources to community healthcare and integrating health visiting.

Some described hubs as combining the interventions discussed above: i.e. staffed by professionals skilled in paediatrics who could observe and assess children. Children's hubs could be an accessible location, target disadvantaged communities and may improve outcomes and/or impact on GP and hospital workload, although in remote and rural areas there may still be considerable travel distance.

Hospital at Home model

Parent and toddler group PPI strongly indicated that families face multiple financial and logistical barriers to attending healthcare services including: no car, the high cost of public transport and taxis; no 24-hour public transport; parking challenges; time availability; and lack of childcare for other children.

Health professionals suggested an intervention which would provide more support and medical care for families in their own home, through a 'hospital at home' model. They cited hospital admissions for non-medical reasons, for example parental concern, or lack of capacity in the current system to assess children or follow-up at home.

The experiences of some parents support the model, e.g. a child with croup and parents' desire for easy and/or direct access to urgent care from paediatric specialists (depending on the intervention design and

For peer review only - http://bmjopen.bmj.com/site/about/guidelines.xhtml

delivery). There is some complementarity and potential to combine interventions :i increasing primary-care staff paediatric skills, assessment and observation in the community, community hubs, and hospital at home to address the outcomes of importance to parents and professionals.[10}

Extend specialised care pathways for sub-groups of children

Some parents described specialist care pathways for their children with acute or chronic relapsing conditions. Consultants, nurses and GPs from spoke of the benefit of existing specialist care pathways for a minority of conditions, e.g. epilepsy and diabetes. A primary aim of these pathways is to equip and support parents to manage children at home, and thus avoid unnecessary trips to hospital. Interviewees proposed extending specialist care pathways to other acute conditions and/or age groups as a way of triaging to appropriate expertise.

Many health professionals viewed babies with fever, and young children with respiratory illnesses as primary targets for future interventions which may improve their outcomes and impact on attendances in primary and secondary care. Convulsions were frequently raised and are a common reason for SSA explored in depth elsewhere (Malcolm et al, 2023).

Results of Stakeholder Intervention Prioritisation

The stakeholder event had 47 attendees (22 attending online): 7 parents, 12 health professionals (primary and secondary care nurses, consultants, nurse consultants and GPs), and 28 representatives from stakeholder organisations (charities and parent groups' co-ordinators, academics, and government).

Increase community specialist acute paediatric nursing roles for care of the acutely unwell child

Stakeholders affirmed the importance of ANPPs with expertise in care of the acutely unwell child, especially in areas where patients live far from a hospital. A skilled ANPP in the GP practice could potentially send parents home with a care plan and prevent SSAs and/or reduce pressure on OOH services.

For peer review only - http://bmjopen.bmj.com/site/about/guidelines.xhtml

ANPPs are seen as appropriate professionals to undertake neonatal assessments and manage chronic paediatric conditions for which children would otherwise attend hospital out-patient clinics. A caveat expressed by stakeholders (but not interviewees), was the importance of ANPPs being rooted in secondary care, whether through rotation or regular shifts, to keep up to date and avoid losing their skills in dealing with acutely unwell children. More integrated and fluid professional roles across primary-secondary care services, rather than the current dichotomy of community versus hospital were envisioned.

Stakeholders identified several challenges for increasing community ANPP roles: to fully train an ANPP can take around five years, so increasing numbers needs advance planning; some hospital ANPPs do not see children under the ages of 5 or 12 years, and thus require more training to become confident in managing younger children; many community ANP roles are advertised for adult care only. Some hospital health professionals lamented the potential loss of their skilled ANPPs to primary care. Stakeholders with paediatric nursing experience suggested extending roles in primary care could de-skill other GP and nursing staff through reducing contact with unwell children.

Improve paediatric skills by creating rotational posts between primary and secondary care settings

Stakeholders recognised the value of observing acutely unwell children in community settings but acknowledged that it would require confidence-building in both staff and parents. Increasing paediatric skills in the community for GPs and nurses was acknowledged to be dependent on population geography, existing service structure and staff resource, with only one health professional specifically mentioning rotational posts for nurses. Others mentioned the benefits of less formal cross-overs between care settings, e.g. skills gained from previous roles, GPs training in secondary care, or hospital clinicians holding occasional clinics in primary care. A more integrated/fluid rotation of staff across primary and secondary care would provide a ‘safety net’ when creating a service that relies on a few highly specialised staff.

Creation of holistic children’s ‘hubs’

Similar to interviewees, stakeholders suggested potential hubs as providing holistic paediatric services include a focus on preventing and managing childhood illnesses; Rs and situated in the community with good transport links and parking. Hubs could provide care for extended hours, bridging the current gap

For peer review only - http://bmjopen.bmj.com/site/about/guidelines.xhtml

between general practice opening hours (8am-6pm) and the busiest time in urgent care (6pm -2am). A few health professionals commented that observation and assessment in hubs may particularly suit children where National Institute of Health and Care Excellence (NICE) pathways apply, e.g., bronchiolitis.

Unlike interviewees, stakeholders raised several concerns about potential hubs: they may become too busy; parents may become over-reliant rather than learning how to manage childhood illnesses at home; information and advice currently provided by the 24 hour NHS telephone advice and triage service (NHS24) could be replaced by the hubs, which may result in unforeseen issues such as a lack of access to the large bank of language interpreters. A substantial cultural shift in parental confidence with primary care and community services will be required, otherwise parents may simply bypass the hubs and go straight to ED.

Stakeholders perceived the massive investment and political change required for hubs as unrealistic, given previous promises of more investment in community health services have not translated into practice. Furthermore, they highlighted that interventions such as hubs would only be viable with adequate staffing which could be a barrier with the current workforce shortage.

Hospital at Home model

Stakeholders discussed the hospital at home model only briefly. Some stakeholders raised concerns about who would staff a hospital at home model because this might deplete staff in existing services, putting even more strain on them. Others felt that hospital at home might be a way of retaining staff, e.g., nurses, who no longer want to work on acute wards. In support of the model, some parents stated that, with hindsight, the help and reassurance they had received from SSAs could have been provided in the community and the model could potentially provide the continuity of care and familiar staff that parents want.

Extend specialised care pathways

Stakeholders spent less time discussing specialist care pathways for bronchiolitis, convulsions, and infants aged under 2 years. Some parents had experience of specialist diabetic and epilepsy nurses and praised the care their children had received. Health professionals preferred condition-based, rather than age-based, pathways. They felt that specialist nursing was appropriate for relatively uncommon conditions

For peer review only - http://bmjopen.bmj.com/site/about/guidelines.xhtml

but was not suitable for frequently occurring conditions and those with existing standardised NICE pathways, such as bronchiolitis. Even where NICE care pathways do exist, health professionals lamented that adherence is variable and education is needed. They recognised that if a child arrives with multiple issues, it often leads to health professionals' confusion over which pathway to follow.

Other specialised pathways were discussed, example e.g. a paediatric-specific NHS24 phoneline, but it was concluded this might be confusing for parents to have yet another telephone number, and that staffing it would be challenging. Stakeholders wanted greater recognition of direct referral to pre-hospital specialists, such as opticians (NHS24 already refer children straight to opticians when appropriate) and pharmacists, who could advise parents around common childhood illnesses.

Results of ranking the priority of interventions

Twenty attendees at the interactive stakeholder event (16 in person and four online) submitted their anonymised priority rankings for the interventions they considered most important with a score of 1 indicating the most popular option and 7 the least popular option. Table 3 shows a cluster of three more popular options (ranks 1-3) and options that were not so popular (ranks 4-7). Stakeholders considered creating specialist care pathways for bronchiolitis, convulsions, and infants aged under 2 years as lower priority interventions, with the lowest priority pathway being one for infants.

Potential intervention	Mean score	Rank
Addressing gaps in acute paediatric skills of health professionals working in community settings	2.53	1
Creation of holistic children's 'hubs'	2.73	2
Assessment and observation of acutely unwell children in community settings	3.00	3

For peer review only - http://bmjopen.bmj.com/site/about/guidelines.xhtml

Hospital at home model.	4.47	4
Specialised care pathways - for wheeze /bronchiolitis.	4.76	5
Specialised care pathways - for convulsions.	4.94	6
Specialised care pathways - for under twos.	5.33	7

Table 3. Results of stakeholder intervention prioritisation ranking

For peer review only

DISCUSSION

In a robust mixed-methods study combining analysis of quantitative and qualitative data, PPI engagement and systematic review evidence [9], we were able to identify and prioritise interventions with potential to improve urgent care pathways for children. Our results can inform more efficient care pathways to improve parent experiences when seeking care for acutely sick children and potentially reduce or prevent hospital admissions. Of seven potential interventions, stakeholders including parents and health professionals identified three higher priority and four lower priority future interventions. Higher priority interventions were to address gaps in the acute paediatric skills of health professionals working in community settings, to create holistic children's hubs, and to facilitate assessment and observation of acutely ill children in community settings. Lower priority interventions were "hospital at home" and specialised care pathways for convulsions, infants under two years old, and wheeze/bronchiolitis. The higher priority interventions could be combined into a whole population intervention which provides community-based assessment and observation by appropriately skilled health professionals in an accessible location/hub.

Strengths include using the INDEX guidance [12] for intervention development which has not been followed previously to address the gap in the systematic evidence [9] about how to improve pre-hospital acute paediatric care and combining qualitative and quantitative methods with stakeholder engagement and PPI. Different data collection, settings and framing of approach generate different perspectives and help in the search for disconfirming data. The health professional contributions are representative of

different professional groups (apart from paramedics who we tried unsuccessfully to recruit) and represented a range of urban and rural /remote areas.

Limitations include that parents talked about their care experiences and preferences, but did not offer specific solutions. Due to COVID19, we could not recruit parents and children face to face at hospitals as intended which limited sample diversity, although our spread of deprivation measured by SIMD was good. . Children's views are not represented, as many were infants and no-one took up the invitation to a video-conference or telephone interview with their child. Solutions for cities might look different to those for remote and rural communities which may have been under-represented at the stakeholder event. Rural and remote areas have distance, travel and different workload/ resource considerations. Generalisability of findings beyond Scotland and to the post-COVID years is uncertain, however many staff have cross-UK and international experience. The priority ranking exercise was exploratory and only 20 out of 47 attendees completed it.

Our study is unique in its inclusion of health professional perspectives and triangulation of different stakeholder perspectives to inform paediatric care intervention development. Previous published qualitative research has focused on parents' perspectives of admissions [26, 27]. Prioritising what matters to patients and health professional (the target population) is important when designing an intervention with a congruent theory of change and will inform future decisions about the focus for future evaluation design [28]. Our findings complement a recent rapid literature review of (not paediatric-specific) patient urgent and emergency care experiences [29]. Another recent review of the literature was published after our study was completed [8]. This second review found that telemedicine was useful in terms of preventing admissions from the community, whilst the initial systematic review had focussed on hospital-based interventions. Although this did not inform our findings it also calls for prompt introduction and evaluation of improved pre-hospital pathways for the care of acutely unwell children.

The importance of rotation of staff and the role of ANPPs in integrated services to facilitate early referral to the Community Children's Nursing Teams as an alternative to hospital care; strong personal relationships between consultants and community children's nurses are considered important [30]. In England, what reassures professionals, especially GPs, when referring to Community Children's Nurses are clear clinical governance protocols [31]. There is some evidence to indicate that an experienced GP with paediatric training may be the best person to decide whether a child is ill or not [32]. Research in Scotland revealed that the quality of relationships, communication, and expectations between GPs and hospital

For peer review only - http://bmjopen.bmj.com/site/about/guidelines.xhtml

specialists at the interface between primary and secondary care were important influences on patient care [33].

Evidence of the best short- and long-term outcomes for children and families is required before novel pathways are implemented into routine acute care pathways. Further intervention acceptability, feasibility/pilot testing research is required using randomised controlled trial methodology to establish effectiveness, cost-effectiveness and any unintended consequences over short to longer term horizons.

The key message for policy-makers is that to improve paediatric urgent care pathways stakeholders, including health professionals and families, want future interventions that are safe, patient-centred and community-based [3]. Close collaboration between academics and policy makers, senior decision makers and potential funders will be required to ensure that future interventions value contributions from parents and front-line health professionals and are rigorously evaluated to advance evidence based policy and care that improves child health outcomes.

FUNDING STATEMENT

This work was supported by the Chief Scientist Office of the Scottish Government, grant number HIPS/18/09.

COMPETING INTERESTS STATEMENT

No competing interests to declare.

DATA AVAILABILITY STATEMENT

The qualitative data collected and analysed during this study are not publicly available due to privacy issues. We are not able to share this data as they contain potentially identifying and sensitive participant information. Moreover, participants were not informed and did not give consent for their transcripts to be shared in a public repository. Queries regarding data access may be directed to the authors or the research governance team (researchgovernance@abdn.ac.uk).

ETHICS APPROVAL STATEMENT

The study was approved by the NHS North of Scotland Research Ethics Service (REC reference: 19/NS/0134). Local permissions were also obtained from individual Health Boards.

For peer review only - http://bmjopen.bmj.com/site/about/guidelines.xhtml

AUTHOR CONTRIBUTIONS

ST, RK, EF, CM, LA, PW and PH conceived the idea. SD, LA and ST undertook the quantitative research. CM, EK, EF and PH undertook the qualitative research. SK undertook patient and public involvement engagement and co-ordination. EK wrote the first draft of the manuscript. All authors made important contributions to revisions of the paper and have seen the final submitted version. ST is the guarantor of the work.

ACKNOWLEDGEMENTS

Amy Woodhouse of Children in Scotland registered charity critically reviewed and commented on drafts of this manuscript. We are grateful for Dave Kelly at Albasoft for providing primary care data. We are grateful to Rebecca Fairnie at Electronic Data Research and Innovation Service for managing our access to all data.

The authors would like to extend our thanks to the parents and health professionals who participated in this study, and to the stakeholders who participated in both PPI activities and the engagement and prioritisation event.

REFERENCES

1. Ruzangi J, Blair M, Cecil E, Greenfield G, Bottle A, Hargreaves DS, et al. Trends in healthcare use in children aged less than 15 years: a population-based cohort study in England from 2007 to 2017. BMJ Open. 2020;10(5):e033761.
2. Al-Mahtot M, Barwise-Munro R, Wilson P, Turner S. Changing characteristics of hospital admissions but not the children admitted-a whole population study between 2000 and 2013. European Journal Of Pediatrics. 2018;177(3):381-8.
3. Malcolm C, King E, France E, Kyle RG, Kumar S, Dick S, et al. Short stay hospital admissions for an acutely unwell child: A qualitative study of outcomes that matter to parents and professionals. PLoS One. 2022;17(12):e0278777.
4. King E, Dick S, Hoddinott P, Malcolm C, France E, Kyle RG, et al. Regional variations in short stay urgent paediatric hospital admissions - a sequential mixed methods approach exploring differences through data linkage and qualitative interviews. BMJ Open. Accepted.
5. Barwise-Munro R, Morgan H, Turner S. Physician and Parental Decision-Making Prior to Acute Medical Paediatric Admission. Healthcare (Basel, Switzerland). 2018;6(3).
6. Williams TC, MacRae C, Swann OV, Haseeb H, Cunningham S, Davies P, et al. Indirect effects of the COVID-19 pandemic on paediatric healthcare use and severe disease: a retrospective national cohort study. Archives of Disease in Childhood. 2021;106(9):911-7.
7. Morris J, Fisher E. Growing problems, in depth: The impact of Covid-19 on health care for children and young people in England. The Nuffield Trust, ; 2022 18/02/2022.

For peer review only - http://bmjopen.bmj.com/site/about/guidelines.xhtml

8. Dick S, MacRae C, McFaul C, Wilson P, Turner SW. Interventions in primary and community care to reduce urgent paediatric hospital admissions: systematic review. Archives of Disease in Childhood. 2023:archdischild-2022-324986.
9. Dick S, MacRae C, McFaul C, Rasul U, Wilson P, Turner SW. Interventions to reduce acute paediatric hospital admissions: a systematic review. Arch Dis Child. 2022;107(3):234-43.
10. Coon JT, Martin A, Abdul-Rahman AK, Boddy K, Whear R, Collinson A, et al. Interventions to reduce acute paediatric hospital admissions: a systematic review. Arch Dis Child. 2012;97(4):304-11.
11. Skivington K, Matthews L, Simpson SA, Craig P, Baird J, Blazeby JM, et al. Framework for the development and evaluation of complex interventions: gap analysis, workshop and consultation-informed update. 2021;25:57.
12. O'Cathain A, Croot L, Duncan E, Rousseau N, Sworn K, Turner KM, et al. Guidance on how to develop complex interventions to improve health and healthcare. BMJ Open. 2019;9(8):e029954.
13. Dick S, Kyle R, Wilson P, Aucott L, France E, King E, et al. Insights from and limitations of data linkage studies: analysis of short-stay urgent admission referral source from routinely collected Scottish data. Archives of Disease in Childhood. 2022:archdischild-2022-324171.
14. National Institute for Health Research. Briefing notes for researchers - public involvement in NHS, health and social care research 2021 [Available from: https://www.nihr.ac.uk/documents/briefing-notes-for-researchers-public-involvement-in-nhs-health-and-social-care-research/27371.
15. UK Standards for Public Involvement. UK Standards for Public Involvement 2016 [Available from: https://sites.google.com/nihr.ac.uk/pi-standards/home.
16. Williams TC, Bach CC, Matthiesen NB, Henriksen TB, Gagliardi L. Directed acyclic graphs: a tool for causal studies in paediatrics. Pediatr Res. 2018;84(4):487-93.
17. Textor J. DAGitty - draw and analyze causal diagrams [Available from: http://www.dagitty.net/.
18. Ritchie J, Spencer L. Qualitative data analysis for applied policy research. Analyzing qualitative data: Routledge; 2002. p. 187-208.
19. Coughlan CH, Ruzangi J, Neale FK, Nezafat Maldonado B, Blair M, Bottle A, et al. Social and ethnic group differences in healthcare use by children aged 0–14 years: a population-based cohort study in England from 2007 to 2017. Archives of Disease in Childhood. 2022;107(1):32-9.
20. Rose TC, Adams NL, Whitehead M, Wickham S, O'Brien SJ, Hawker J, et al. Neighbourhood unemployment and other socio-demographic predictors of emergency hospitalisation for infectious intestinal disease in England: A longitudinal ecological study. J Infect. 2020;81(5):736-42.
21. Sartain SA, Maxwell MJ, Todd PJ, Jones KH, Bagust A, Haycox A, et al. Randomised controlled trial comparing an acute paediatric hospital at home scheme with conventional hospital care. Arch Dis Child. 2002;87(5):371-5.
22. Jones E, Taylor B, Rudge G, MacArthur C, Jyothish D, Simkiss D, et al. Hospitalisation after birth of infants: cross sectional analysis of potentially avoidable admissions across England using hospital episode statistics. BMC Pediatrics. 2018;18(1):390.
23. Scottish Home and Health Department. Towards an integrated child health service: H.M. Stationery Office. Edinburgh.; 1973.
24. Dunhill Z. Developing a Community Child Health Service for the 21st Century. The Scottish Government, ; 2013.
25. Kerr A. Building a Health Service fit for the future. NHS Scotland; 2005.
26. Jones CH, Neill S, Lakhanpaul M, Roland D, Singlehurst-Mooney H, Thompson M. Information needs of parents for acute childhood illness: determining 'what, how, where and when'of safety netting using a qualitative exploration with parents and clinicians. BMJ open. 2014;4(1):e003874.
27. Neill SJ, Jones CHD, Lakhanpaul M, Roland DT, Thompson MJ. Parents' help-seeking behaviours during acute childhood illness at home: A contribution to explanatory theory. Journal Of Child Health Care: For Professionals Working With Children In The Hospital And Community. 2016;20(1):77-86.

For peer review only - http://bmjopen.bmj.com/site/about/guidelines.xhtml

28. Tovey D, Blaine C. How to clarify a clinical question 2022 [Available from: https://bestpractice.bmj.com/info/toolkit/learn-ebm/how-to-clarify-a-clinical-question.
29. The Patient Experience Library. Patient experience in urgent and emergency care. 2022.
30. Kyle RG, Banks M, Kirk S, Powell P, Callery P. Integrating community children's nursing in urgent and emergency care: a qualitative comparison of two teams in North West England. BMC pediatrics. 2012;12(1):101.
31. Kyle RG, Banks M, Kirk S, Powell P, Callery P. Avoiding inappropriate paediatric admission: facilitating General Practitioner referral to Community Children's Nursing Teams. BMC family practice. 2013;14(1):4.
32. Stephenson T. Paediatric primary care in Europe: variation between countries. Arch Dis Child. 2010;95(10):767-8.
33. Sampson R, Barbour R, Wilson P. The relationship between GPs and hospital consultants and the implications for patient care: a qualitative study. BMC Family Practice. 2016;17(1):45.

SUPPLEMENTARY FILES

Supplementary file 1. Detailed methods

Supplementary file 2. Semi-structured interview guide (health professionals)

Supplementary file 3. Semi-structured interview guide (parents)

Supplementary file 4. Participant information sheet- qualitative study

Supplementary file 5. Dag model

Supplementary file 5a. Revised logic model

Supplementary file 6. Materials & methods for stakeholder event

Supplementary file 6a. Stakeholder event morning slides

Supplementary file 6b. Stakeholder event afternoon slides

Supplementary file 7. Characteristics of qualitative interview participants

Supplementary file 8. Supporting interview quotations

Research Checklists:

GUIDED reporting checklist

For peer review only - http://bmjopen.bmj.com/site/about/guidelines.xhtml

For peer review only

For peer review only - http://bmjopen.bmj.com/site/about/guidelines.xhtml

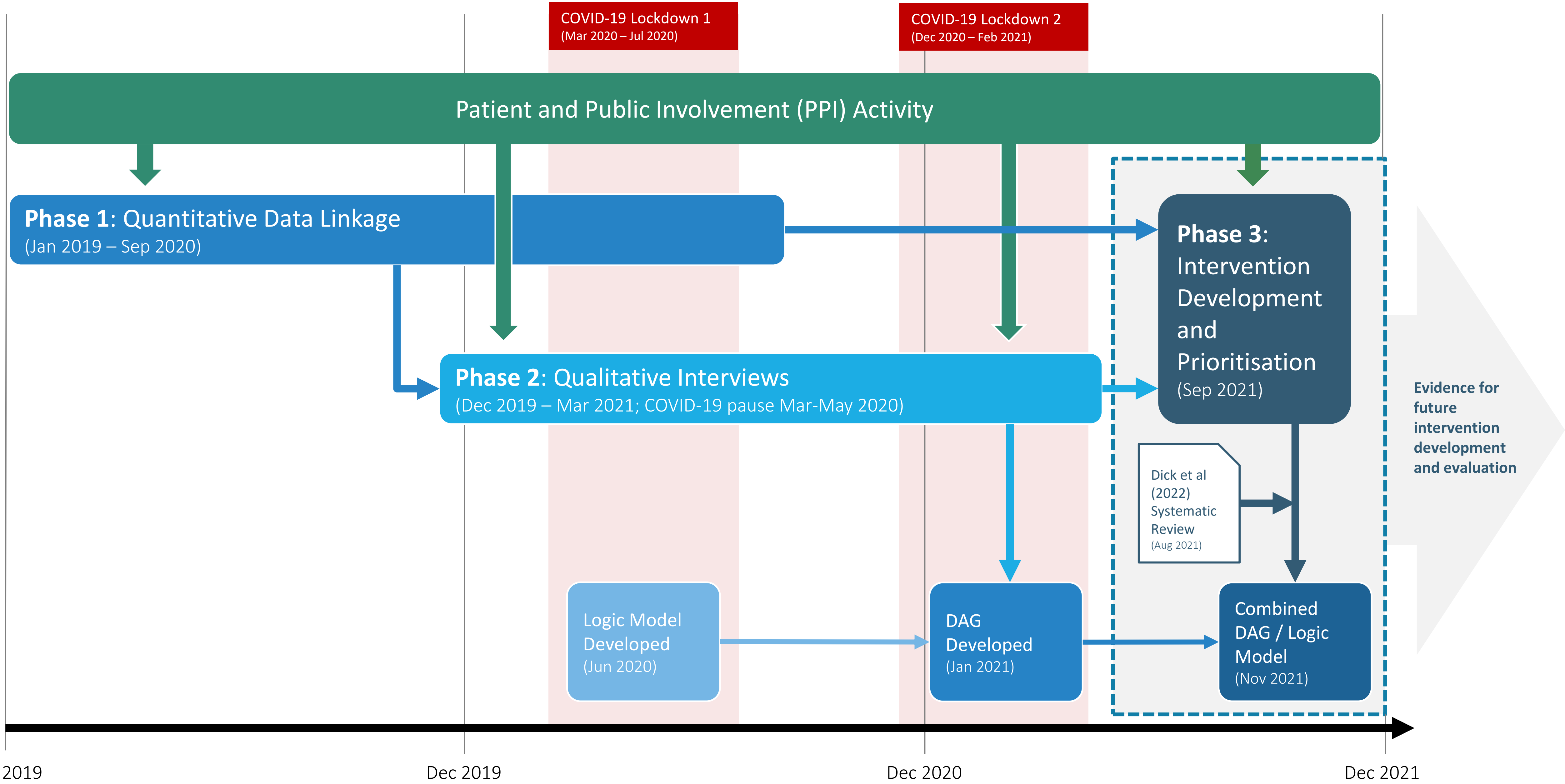
COVID-19 Lockdown 1
(Mar 2020 – Jul 2020)
COVID-19 Lockdown 2
(Dec 2020 – Feb 2021)
Patient and Public Involvement (PPI) Activity
Phase 1: Quantitative Data Linkage
(Jan 2019 – Sep 2020)
Phase 2: Qualitative Interviews
(Dec 2019 – Mar 2021; COVID-19 pause Mar-May 2020)
Phase 3: Intervention Development and Prioritisation
(Sep 2021)
Dick et al (2022) Systematic Review
(Aug 2021)
Evidence for future intervention development and evaluation
Logic Model Developed
(Jun 2020)
DAG Developed
(Jan 2021)
Combined DAG / Logic Model
(Nov 2021)
Jan 2019
Dec 2019
Dec 2020
Dec 2021

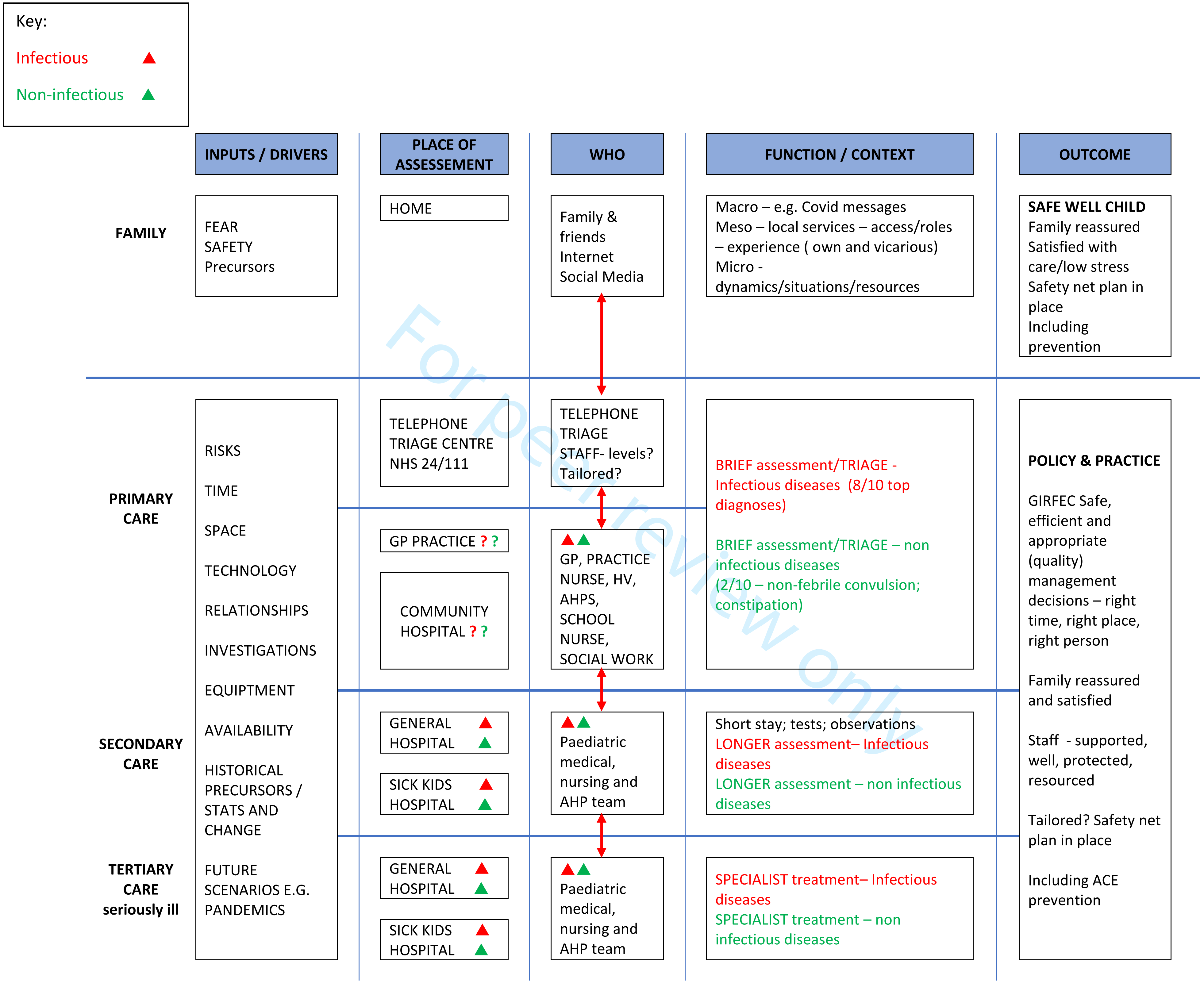

For peer review only

Supplementary file 1. FLAMINGO study detailed methods for data linkage and qualitative interviews

Study design and setting

The mixed-methods FLAMINGO study was undertaken in Scotland, where the NHS is organised into 14 geographically distinct terrestrial Health Boards, each responsible for healthcare provision to their region's population. The intervention development used a mixed methods phased approach and was target population centred, incorporating front-line health professional and patient perspectives collected through Public and Patient Involvement (PPI), stakeholders, and qualitative interviews. Patient and public involvement occurred throughout the project from inception to prioritisation of findings. Prioritising outcomes that matter to patient and staff participants (the target population) is important when designing an intervention with a congruent theory of change and will inform future decisions about the Population Intervention Comparator Outcome (PICO) for future evaluation design.[1] A programme theory of change was developed early in Phase 1 informed by the DAGIT process/model. [2] [Supplement 5] The DAGIT was referred to iteratively throughout the FLAMINGO study and incorporated into a final diagrammatic theory of change once the data analysis was complete [Supplement 5a]. The whole Flamingo team met monthly, the quantitative and qualitative sub teams met separately in between. An underpinning Systematic Review of interventions to reduce acute paediatric hospital admissions was undertaken by a FLAMINGO sub-team.[3] It was unclear at the start whether the systematic review would identify an intervention with sufficient promise to be adapted or whether developing a new intervention would be indicated. The findings were shared with the FLAMINGO team once qualitative data collection was complete and were incorporated into the intervention prioritisation process.

The study was approved by the NHS North of Scotland Research Ethics Service (REC reference: 19/NS/0134).

Data linkage methods

Study design

The data linkage study is reported in detail elsewhere.[4] Data from the following five clinical services were linked: Hospital admission details (Scottish Morbidity Record 01, SMR01); Out-of-Hours, Emergency Department and NHS 24 (all within the Unscheduled Care Datamart, UCD); general

For peer review only

practice data (provided by Albasoft, an NHS trusted third party). The period of was 2015-2017 and was determined by the availability of data from UCD (beginning April 2014) and the onset of the study (2019). A referral source was defined as the location (ED, GP, OOH or combinations thereof) where the child had a clinical record for the day of admission. When there was contact with more than one referral source on the day of admission, sources were placed in chronological order where possible (time of GP contact was not provided in the majority of records). When no referral source was identified we took two additional approaches (i) contact with a referral source the day before admission was sought (ii) we determined whether the child had been discharged from hospital on the same day as admission or the day before (“open access admissions”).

Setting

Two NHS Scotland terrestrial health boards (Lothian, and Greater Glasgow and Clyde) have ED facilities staffed by paediatricians but in all other health boards, staff trained in emergency medicine see children in an ED. Three health boards have very limited in-patient paediatric facilities (Orkney, Shetland, and Western Isles) and no data from these boards were included in this analysis. Four health boards have children’s hospitals (Greater Glasgow and Clyde, Grampian, Lothian, and Tayside).

Participants

Individuals <16 years with ≥ one urgent admission to hospital under the care of a medical paediatric team in Scotland in the calendar years 2015-2017. Children seen in and discharged from the ED or admitted under paediatric surgical, orthopaedic or dental teams were ineligible.

Ethical approval

The study was approved by the Public Benefit and Privacy Panel for Health and Social Care (reference 1718-0183). Data were analysed in the National Data Safe Haven of Scotland,[5] a secure repository accessed through a virtual private network by trained researchers.

Data sources

Scottish Morbidity Record 01, SMR01.[6] The SMR01 data are assessed for quality and completeness and are estimated to be 99% complete as of March 2018.[7] Data provided were: sex; ethnic group; decimal age; date of admission; date of discharge; admission type (emergency or elective); Scottish Index of Multiple Deprivations (SIMD) quintile; up to six ICD-10 diagnoses; Health Board of admission; specialty (paediatric medicine, paediatric surgery or paediatric dentistry). Time of admission was not available.

For peer review only

Unscheduled Care Datamart (ED, OOH and NHS24 data). This resource provides data from ED, OOH and NHS 24 (the latter including the Scottish Ambulance Service). All records have a valid CHI number.[8]

GP Data (Albasoft). GP data were made available through NHS Trusted Third Party (Albasoft) using a previously described method.[9] Briefly all Scottish general practices have software potentially accessible by Albasoft and practices can opt in and consent to share data with researchers via Albasoft. Typically, 15-20% of practices agree to provide data.[9] Where records were available, Read codes were used to distinguish clinical contact leading to a referral from administrative entries arising from clinical contact (e.g. a letter from ED after presenting there).

Bias

Completeness of data for SMR01 and the UCD ensures no bias. GP data were only available after practices opted in and this may introduce bias.

Study size

The study size was all individuals aged <16 years with an urgent admission to hospitals in Scotland in the calendar years 2015-2017.

Statistics

Descriptive statistics are provided for comparisons between groups. Multivariate logistic models were used to compare odds ratio (OR) for the ten most common composite diagnoses stratified by referral source. Composite diagnosis was defined as a group of very similar conditions, e.g. “asthma” included International Classification of Disease (ICD)-10 code J45.0 (predominantly allergic asthma), J45.9 (other and unspecified asthma) and J46X (Status Asthmaticus).[10] The benefit of using composite diagnoses is that they minimise the potential for variations in coding between units.[11] The following accounted for 46.4% of all urgent admissions in Scotland 2000-2013: asthma, bronchiolitis, convulsion (including febrile and afebrile convulsions), croup, gastroenteritis, upper respiratory tract infection (URTI), viral infection, tonsillitis, lower respiratory tract infections (LRTI) and admissions with a diagnosis of cough or wheeze or shortness of breath.[10] A stringent p value of <0.001 was used to indicate a meaningful association since the sample size was so large. Standard statistical software was used for the analysis (IBM® SPSS® version 24).

For peer review only

FLAMINGO qualitative interview methods

A qualitative exploratory approach was used to gain a better understanding of the experiences of parents and health professionals with regards to the circumstances around unscheduled SSAs of children (up to the age of 16) to hospital.

Qualitative setting

The FLAMINGO project team purposively identified five Health Boards to act as cases that would allow for maximal variation in characteristics such as deprivation, urban-rural, and hospital structure (with or without a dedicated children's hospital or short stay facility). Case selection was also informed by the quantitative data emerging from the data linkage exercise to allow for variation in numbers of unscheduled hospital admissions.

Participants and sampling

Health professionals were recruited by the team from a paediatric medical conference in Edinburgh in 2019, using their professional networks, and snowball sampling. Purposive sampling was used to invite health professionals working in primary care, GP out of hours services (OOH), and hospital emergency departments (ED)/ paediatric ED (PEDs) workplaces, NHS24, and the Scottish Ambulance service in the five case Health Boards, with responsibility for referring or receiving children for acute paediatric medical admission. Recruitment was unsuccessful from NHS24 and the Scottish Ambulance service.

Initial Patient and Public Involvement discussions revealed that many parents were unsure at what point in a hospital visit their child was officially categorised being admitted. The quantitative analysis of the data linkage analysed SSA where admission and discharge occur on the same calendar date.[4] To improve clarity when recruiting parents for interview, the definition of a SSA was therefore adapted to include stays in hospital lasting less than 24 hours. Parents were eligible to participate if they had experience of a SSA for their child with an acute medical illness and taken place within the past five years. SSAs for surgical or dental conditions were excluded.

The COVID-19 pandemic coincided with our qualitative recruitment phase and associated restrictions such as 'lockdowns' meant that previously planned in person recruitment methods at hospitals and places frequented by parents could not be undertaken. Alternative convenience sampling methods were adopted including online platforms and social media, press releases and advertisements in local newspapers. Parents responding to the invitation were sent a participant information sheet, study details and guidance on how to contact the main researcher (EK) to arrange a telephone interview.

For peer review only

Due to the challenges of recruiting to health research during the pandemic,[12] and the project's objective of ensuring maximal variation of participants, the recruitment press release in January 2021 invited eligible participants from outside the five Health Board case areas.

In addition, Phase 1 quantitative analysis and early interviews with families raised seizures as a particularly interesting reason for a SSA. Further purposive sampling and recruitment of health professionals and families with experience of seizure and findings will be reported separately.

Data collection

Interviews were conducted by two experienced qualitative researchers (EK, CM) with different backgrounds (health services research and paediatric nursing) and reflective field notes were kept. Semi-structured interviews were chosen for consistency between the two interviewers (EK, CM) whilst allowing interviewees to spontaneously raise issues. Separate semi-structured interview topic guides were developed for parents and health professional interviews by our multidisciplinary research team, with patient and public involvement (PPI) and drawing on existing literature on urgent short stay hospital admissions [the interview topic guides are in Supplementary files 2 and 3]. The topic guides included a specific question about recommendations for change regarding urgent SSAs. Health professionals were asked to propose potential solutions to improve urgent care pathways for children between home and hospital admission. Parents were asked questions relating to how their family's experiences could be improved in future.

The interviews took place between December 2019 and March 2021, pausing due to the COVID-19 lockdown between March and May 2020. Telephone interviews replaced face-to-face interviews. The COVID-19 pandemic was a context disruptor and data collected during this period might not be fully representative of typical SSA practices; therefore, interviewees were asked to speak about experiences pre-COVID. Interviewees were then asked specifically if and how they had observed differences during, or as a consequence of, the pandemic.

The duration of the interviews ranged from 18 to 62 minutes (median 27) and each interview was audio recorded, transcribed verbatim and anonymised.

For peer review only

Data analysis

Anonymised transcripts were uploaded to QSR NVivo 12 data management software (QSR International Pty Ltd., Version 12, 2019, Victoria, Australia) and were analysed using the Framework Method, used widely across health services research, which offers a systematic and flexible model for supporting qualitative thematic analysis.[13] It is particularly useful in situations where there is a desire to identify themes through making comparisons both within and between cases.[13] This was the aim of the current research which set out to explore the experiences of SSAs from the perspective of both parents and health professionals. The framework approach involves a five-stage iterative process of 1) data familiarisation; 2) identification of a thematic coding framework; 3) indexing and further refinement of the coding framework; 4) charting; and 5) mapping and interpretation to search for patterns and explanations in the data. During the initial data familiarisation stage, members of the FLAMINGO qualitative team (EK, CM, EF, PH) independently read a sample of four transcripts and field notes, two from each participant group (parents and health professionals), in depth and a high-level coding framework was devised by each member. The group met on several occasions to discuss overlaps and differences between the four coding indexes which were merged to form the first version of the coding framework. This framework, and the main thematic areas to focus on in the analysis, was then shared with the wider FLAMINGO team for additional comments and input. A subset of six transcripts were independently coded by EK and CM and the results compared to reveal considerable reliability in coding. Subsequently, minor changes to the codes were made, in order to further improve consistency.

The third indexing stage involved systematically applying the analytical coding framework to the remaining transcripts in NVivo. Line by line coding was carried out by three researchers (EK, CM, EF), with ongoing discussion and debate at weekly meetings. A framework matrix was developed to allow for organisation of the data samples (parents and health professionals) by themes to facilitate cross-case and within-sample comparisons. The FLAMINGO qualitative team reviewed the final themes to reach consensus in the mapping and interpretation of the data, thus enhancing rigour and trustworthiness.

Analysis of interview data relating to the outcomes and values that matter to parents and professionals when designing an intervention to improve care for children with SSAs is reported in Malcolm et al.[14]

For peer review only

References

1. Tovey D, Blaine C. How to clarify a clinical question 2022 [Available from: https://bestpractice.bmj.com/info/toolkit/learn-ebm/how-to-clarify-a-clinical-question.
2. Williams TC, Bach CC, Matthiesen NB, Henriksen TB, Gagliardi L. Directed acyclic graphs: a tool for causal studies in paediatrics. Pediatr Res. 2018;84(4):487-93.
3. Dick S, MacRae C, McFaul C, Rasul U, Wilson P, Turner SW. Interventions to reduce acute paediatric hospital admissions: a systematic review. Arch Dis Child. 2022;107(3):234-43.
4. Dick S, Kyle R, Wilson P, Aucott L, France E, King E, et al. Insights from and limitations of data linkage studies: analysis of short-stay urgent admission referral source from routinely collected Scottish data. Archives of Disease in Childhood. 2022:archdischild-2022-324171.
5. ISD Scotland. National Safe Haven [Available from: https://www.isdscotland.org/Products-and-Services/eDRIS/Use-of-the-National-Safe-Haven/.
6. ISD Scotland. General Acute Inpatient and Day Case - Scottish Morbidity Record (SMR01) [Available from: https://www.ndc.scot.nhs.uk/National-Datasets/data.asp?SubID=5.
7. ISD SG. Data Quality and Completeness for the Annual Acute Hospital Activity and NHS Beds Activity Publication 2018 [Available from: https://www.isdscotland.org/Health-Topics/Hospital-Care/Publications/2018-09-25/Data-Issues-and-Completeness-Sep18.pdf.
8. ISD Scotland. Unscheduled Care Datamart (UCD) [Available from: https://www.ndc.scot.nhs.uk/National-Datasets/data.asp?SubID=111.
9. Ellis DA, McQueenie R, McConnachie A, Wilson P, Williamson AE. Demographic and practice factors predicting repeated non-attendance in primary care: a national retrospective cohort analysis. Lancet Public Health. 2017;2(12):e551-e9.
10. Al-Mahtot M, Barwise-Munro R, Wilson P, Turner S. Changing characteristics of hospital admissions but not the children admitted-a whole population study between 2000 and 2013. European Journal Of Pediatrics. 2018;177(3):381-8.
11. Burns EM, Rigby E, Mamidanna R, Bottle A, Aylin P, Ziprin P, et al. Systematic review of discharge coding accuracy. J Public Health (Oxf). 2012;34(1):138-48.
12. Singh JA, Bandewar SV, Bukusi EA. The impact of the COVID-19 pandemic response on other health research. Bull World Health Organ. 2020;98(9):625-31.
13. Gale NK, Heath G, Cameron E, Rashid S, Redwood S. Using the framework method for the analysis of qualitative data in multi-disciplinary health research. BMC Medical Research Methodology. 2013;13(1):117.
14. Malcolm C, King E, France E, Kyle RG, Kumar S, Dick S, et al. Short stay hospital admissions for an acutely unwell child: A qualitative study of outcomes that matter to parents and professionals. PLoS One. 2022;17(12):e0278777.

For peer review only

The FLAMINGO project (**FL**ow of **A**d**M**issions in ch**I**ldren and you**NG** pe**O**ple)

Health Professional Interview Topic Guide

Can you start by confirming what your role is and your employer?
Probes – confirm role, employer, how long they have been in this role.

Experience of short stay admissions (SSAs)

We are interested in unplanned or emergency hospital admissions in children, particularly short stay admissions (SSA) where a child is admitted and then discharged from hospital on the same day (less than 24 hours).

Can you tell me about your experiences of SSAs of children?
Probes: frequency; typical reasons for them; referral sources – self, GP, NHS 111 etc; conditions, different decision makers (e.g. junior or senior paediatrician, A+E doctor, nurse practitioner) any other care pathways that we haven't talked about.

HOSPITAL STAFF ONLY. Please tell me about your experiences of receiving referrals for/admitting a child where you suspected they did not need to be admitted to hospital. (Probe – can you give me a specific example…)

COMMUNITY STAFF ONLY Can you think of any examples of where a child was admitted to hospital where you thought it was inappropriate/unnecessary?

Do you think that any hospital admissions of less than 24 hours could be safely avoided? (Probe – how?)

What involvement does the child and family have in the referral and admission decision-making process?

What role do the child and family's circumstances have in the referral and admission decision-making process, if any?

What role do other staff in the hospital (probe - nurses, managers) or in the community (e.g. information from the GP about the family) play. Can you think of any examples?

Some people we have talked to refer to appropriate and inappropriate admissions. What are your thoughts about these terms….? What do you consider to be an appropriate referral of an unwell child to a hospital for a short stay? Tell me about some of your experiences.

Readmissions

Can you think of occasions where a child was admitted to hospital for less than 24 hours and then required re-admission within a week or so for the same episode?

For peer review only

Tell me about your experiences of readmission (Probes: something that happens frequently/infrequently; when these types of readmissions do occur, what do you think are the reasons; factors which contribute to this; typical presentation – child/family)

Communication processes between the professional referring and the professional admitting child to hospital

Can you think of an example where communication, between the professional referring and the professional admitting child to hospital, went really well?

- What helped?

Can you think of an example where communication, between the professional referring and the professional admitting child to hospital, did not run so smoothly?

- What were the issues, what were the consequences, how could this be improved?

Question for Referrers only

Please tell me about your experiences of referring a child where you were uncertain if they needed to be admitted, to hospital.

- How do families that you know feel about referral to hospital and short admissions of < 24 hours
- What factors trigger a decision for you to refer a child [when you suspect a SSA might be the outcome]? Probe: for example, the family/parental circumstances

Thinking about the range of different children and families who access our emergency care service:

- Do family social issues have any impact on decisions (give examples)
- Do language issues (such as when English is not the first language) have any impact on decisions to admit children?
- Does the distance to hospital/transport issues/rurality have any impact on decisions to admit children?
- What are your reflections on health inequalities or deprivation and the potential impact on children's admissions, particularly SSAs?

Current situation – COVID-19 pandemic

- With the current situation with COVID-19 are you seeing a change in children attending hospital for unplanned visits? [Probe – which conditions etc]
- How do you think care of sick children at home is changing as a result of Covid-19? (prompts… are there any particular examples you have come across? Tell me more…. Any different examples…?)
- Are there any changes that you think will or should remain after the pandemic?

Final questions- looking forward

Thinking about moving forward and SSAs in children….

- What would be your recommendations for change regarding admissions of less than 24 hours?

For peer review only

- Are there any particular issues or health conditions you would highlight for the next stages of our research?
- What do you think will be happening with children's SSAs in five years' time?
- Is there someone you work with/a clinician you know of who has views very different to yours with respect to admitting children to hospital. Who do suggest we should be speaking to gain different perspectives on SSAs?

For peer review only

The FLAMINGO project (**FL**ow of **A**d**M**issions in ch**I**ldren and you**NG** pe**O**ple)

Family Interview Topic Guide

We are particularly interested in children who are unwell who spend a short time, less than 24 hours, in hospital. We are interested in infections and children who are unwell, so this research is not about accidents and injuries or operations.

Past medical history

- Brief history of child's health and past hospital admissions (purpose to determine the frequency of short stay admissions experienced and identify the admission that the family choose to talk about)

Explore the events and experiences leading up to the child's most recent or most memorable short stay hospital admission, asking families to tell their story from the start…

- What was worrying you about your child (ie. same as symptoms experienced in past? different? worrying?)
- What reasons do you recall for seeking advice?
- Who did you seek advice from? (ie. services and professionals such GP, pharmacist, NHS 24 111, internet, minor injuries unit, A&E; family; friends; other)
- Can you tell me what you understand by the service provided by: Emergency Departments [probe A&E vs minor injuries], NHS 111 / NHS 24?
- What happened next, what were the outcome/s of these initial consultations?
- What other challenges do you face as a family when your child is sick? [Probe: No money in phone, no access to technology, transport, other childcare.]

Explore the family's experience of a zero-day hospital admission,

- Can you tell me what happened from when you walked through the door of the hospital?
- Who did you see first, what happened next, can you recall any feelings experienced?
- Who made the decision that your child would be admitted to hospital? What involvement did you and your family have in any decision making related to the admission?
- What thoughts and feelings did the family have about the decision to admit your child to hospital?
- How often did you move to a different area in the hospital?
- How confident were you in the care team who looked after your child? How consistent was the information that you received from different people?
- Would anything have made a difference to your experience?

Explore the family's experiences of going home after the admission…

- Can you tell me about how the decision was made to discharge your child from hospital and send them home? What was your involvement in that decision making?

For peer review only

- What happened once you were back at home? Did any questions or concerns arise the first few days after discharge from hospital? Was there anything you felt unsure about or wanted further advice on?
- Was your child readmitted for the same illness within two weeks of being sent home? If so how did you feel about this?
- How did your experience change what you would do if a similar situation occurred again?

Explore how the family's experiences could be improved in future…

- How satisfied were you with the process of accessing services for your child?
- Tell me about communication with professionals involved in your child's care?
- What (if anything) would they have liked to be different about your story?
- What other stories have you heard from family and friends about short-stay admissions?
- Some people we have spoken to think some short-stay admissions are unnecessary… what are your thoughts about this?
- What are your views about telephone and face to face consultations for sick children?
- Could technology make a difference – e.g. video call to a children's specialist doctor?

Reflect on current situation – COVID-19 pandemic

- How has the current situation with COVID-19 changed how you would access healthcare for your child?
- How do you feel about monitoring and caring for your child at home?
- How do you think this will impact on how you access healthcare in the future?

Closing the interview…

Thank the parents for sharing their experiences.

Explain how they can get in touch with the research team if they have any thoughts or concerns after the interview.

Explain how they will find out about the results of the research.

For peer review only

FLAMINGO

FLow of AdMissions to hospital in chIldren and younG peOple

Experience of unwell children needing a short stay in hospital (less than 24hrs)? We would like to talk with you.

We would like to invite you to take part in a research project. The project is led by Professor Steve Turner of the University of Aberdeen in partnership with the University of Stirling and Edinburgh Napier University. This part of the research is being carried out by the Nursing Midwifery and Allied Health professions (NMAHP) Research Unit at the University of Stirling.

Before you decide, it is important for you to understand why the research is being done and what it will involve. Please take time to read the following information carefully and to discuss it with others if you wish. Ask us if there is anything that is not clear or if you would like more information. Take time to decide whether you wish to take part. Thank you for reading this.

What is the purpose of the research?

The aim of this project is to understand why children are admitted to hospital for stays of less than 24 hours. To answer this question we are collecting lots of data about hospital admissions and speaking to doctors and nurses. We are also talking to parents about their experience of having a child being admitted to hospital.

Who can take part?

Parents or carers whose child has ever been admitted to hospital for a stay of less than 24 hours for an illness such as a chest infection or fever and not for a broken bone.

Do I have to take part?

No. It is up to you to decide whether you want to take part or not. If you do decide to take part, you will be asked to sign a consent form. You will still be free to stop taking part at any time and without giving a reason. If you stop taking part, or decide not to take part, this will not affect the standard of health care you and your child receive.

What happens if I take part?

You will take part in a short (about 20-45 minutes) interview where a researcher will ask questions about your experiences of your child being admitted to hospital. We can do the interview in a place of your choice, for example, in your home or in a meeting room at the

For peer review only - http://bmjopen.bmj.com/site/about/guidelines.xhtml

university. If you wish, your partner or someone close to you to be present. If your child is 6 years of age or older and they want to take part, then they can take part in the interview with you at the same time. We will give your child age appropriate information on the study and ask them to complete their own consent form.

We might ask the person who referred your child to hospital to also take part in this study. We will audio-record the interview so that we can transcribe what you said and then analyse it. The person who is transcribing (i.e. putting into written text what you have said) will be a third party (i.e. not one of the research team) who will not know who you are. You will have up to 7 days after your interview to let us know if you no longer want us to use your comments. Some parents may find the interview upsetting. If you get upset our experienced interviewers will encourage you to take breaks, you can stop at any time and not take part any more.

What are the possible benefits of taking part?
There will be no direct personal benefit in taking part in this study. However, we hope that your views will be useful in improving the care of children in the future.

What are the possible risks of taking part?
There is a possibility that some parents may become upset. Our staff are trained and very experienced in providing support during interviews

Will my participation in the study be kept confidential?
Yes. We will keep your personal information, such as your name, address and telephone number, confidential in records held in Stirling University. We will record the interview so that your comments can be transcribed and analysed afterwards. We might quote something you said in project publications but we will make sure you cannot be identified. Your comments will not be linked to your name or other personal information and will be stored securely. All data will be managed in line with current data protection guidelines.

If you tell us something during the study which gives us cause for concern about your safety or that of your child, then we have a duty of care to take appropriate action. This could mean that we cannot keep what you told us confidential.

How will we use information about you?
We will need to use information from you for this research project.
This information will include your name and contact details. People will use this information to do the research or to check your records to make sure that the research is being done properly.

People who do not need to know who you are will not be able to see your name or contact details. Your data will have a code number instead. We will keep all information about you safe and secure.

For peer review only - http://bmjopen.bmj.com/site/about/guidelines.xhtml

Once we have finished the study, we will keep some of the data so we can check the results. We will write our reports in a way that no-one can work out that you took part in the study.

What are your choices about how your information is used?
You can stop being part of the study at any time, without giving a reason, but we will keep information about you that we already have.
We need to manage your records in specific ways for the research to be reliable. This means that we won't be able to let you see or change the data we hold about you.

Where can you find out more about how your information is used?
You can find out more about how we use your information

- at www.hra.nhs.uk/information-about-patients/
- our leaflet available from the research team
- by sending an email to dpo@abdn.ac.uk, or by ringing us on 01224-272596.

Who has reviewed this project?
The North of Scotland Research Ethics Committee and specialists in research and health care have reviewed this project.

Who is funding this study?
The Chief Scientist's Office of the Scottish Government.

What if I want to complain?
If you have a concern about any aspect of this project, you should ask to speak to the researchers first 01786-466277. If you are still unhappy and wish to complain formally, you can do this by contacting the University of Aberdeen Research Governance Team via researgovernance@abdn.ac.uk or by phoning 01224-554362.

What do I do if I want to take part?
If you are interested in taking part please call the researcher Emma King at the University of Stirling on 01786-466277, or you can email us on flamingo@stir.ac.uk

Contact details for queries: Dr Emma France. Telephone 01786-466421. Email: emma.france@stir.ac.uk

For peer review only - http://bmjopen.bmj.com/site/about/guidelines.xhtml

FLAMINGO

FLow of AdMissions to hospital in chIldren and younG peOple

Experience of unwell children needing a short stay in hospital (less than 24hrs)? We would like to talk with you.

We would like to invite you to participate in a research project. Before you decide, it is important for you to understand why the research is being done and what it will involve. Please take time to read this following information carefully and to discuss it with others if you wish. Ask us if there is anything that is not clear or if you would like more information. Take time to decide whether you wish to take part. Thank you for reading this.

What is the purpose of the research?

The aim of this study is to understand why children are admitted to hospital. To achieve this aim we are using both quantitative and qualitative methods. We are keen to talk to clinicians or practitioners who refer and receive acute paediatric medical cases, so that we can gain a clinician's perspective on why children are admitted to hospital.

Why have I been chosen?

We have approached you about this study because you are a clinician or practitioner who is involved in acute paediatric admissions to hospital.

Do I have to take part?

No, it is up to you to decide whether or not to take part.

What happens if I take part?

You will take part in a short (about 10 minute) telephone interview where a researcher will ask questions about the decision making which leads to a child being admitted to hospital or discharged home.

What are the possible benefits of taking part?

There will be no direct personal benefit in taking part in this study. However, we hope that your views will be useful in improving the care of children in future.

What are the possible risks of taking part?

There are no risks.

Will participation in the study be kept confidential?

Yes. We will keep your personal information, such as your name and telephone number, confidential. We will record the interview so that we can then transcribe and analyse it. We might quote something you said in project publications but we will make sure you cannot be identified. Your comments will not be linked to your name, workplace name or other personal information and will be stored securely. All data will be managed in line with current data protection guidelines.

For peer review only - http://bmjopen.bmj.com/site/about/guidelines.xhtml

The University of Aberdeen is the sponsor for this study based in the United Kingdom. We will be using information from you in order to undertake this study and will act as the data controller for this study. This means that we are responsible for looking after your information and using it properly. The University of Aberdeen will keep identifiable information about you for 10 years after the study has finished.

Your rights to access, change or move your information are limited, as we need to manage your information in specific ways in order for the research to be reliable and accurate. If you withdraw from the study, we will keep the information about you that we have already obtained. To safeguard your rights, we will use the minimum personally-identifiable information possible.

Your local NHS board will keep your name, and contact details confidential and will not pass this information to University for Aberdeen. Your NHS board will use this information as needed, to contact you about the research study, and make sure that relevant information about the study is recorded for your care, and to oversee the quality of the study. Certain individuals from University of Aberdeen and regulatory organisations may look at your research records to check the accuracy of the research study. The University of Aberdeen will only receive information without any identifying information. The people who analyse the information will not be able to identify you and will not be able to find out your name, or contact details. Your local NHS board will not keep identifiable information about you from this after the study has finished.

You can find out more about how we use your information at http://www.abdn.ac.uk/privacy

Who has reviewed this study?
The study has been reviewed by the North of Scotland Research Ethics Committee and also specialists in the field.

What if I want to complain?
If you have a concern about any aspect of this project, you should ask to speak to the researchers first on 01786-466277. If you are still unhappy and wish to complain formally, you can do this by contacting Professor Jayne Donaldson, the Dean of the Faculty of Health Sciences and Sport, University of Stirling on 01786-466340 or jayne.donaldson@stir.ac.uk.

You have the right to lodge a complaint against the University of Stirling regarding data protection issues with the Information Commissioner's Office (https://ico.org.uk/concerns/).
The University's Data Protection Officer is Joanna Morrow, Deputy Secretary. If you have any questions relating to data protection these can be sent to data.protection@stir.ac.uk.

What do I do now?
If you are interested in taking part, please contact the researcher Dr Emma King at the University of Stirling on 01786-466277. Email flamingo@stir.ac.uk

If you require any further information, Dr Emma France. Telephone 01786-466421. Email: emma.france@stir.ac.uk

For peer review only - http://bmjopen.bmj.com/site/about/guidelines.xhtml

DAG model Nov 2021

Purpose and population

Intervention development in the pathway for unscheduled acute illness in children, access to care and admissions to hospital

Whole Systems and Multi-Stakeholder Approach to Intervention Development

Systematic Review Evidence

Programme Theory

Context- macro

t Rising admissions

- Covid pandemic
- NHS staff shortages and workload
- Health Inequalities

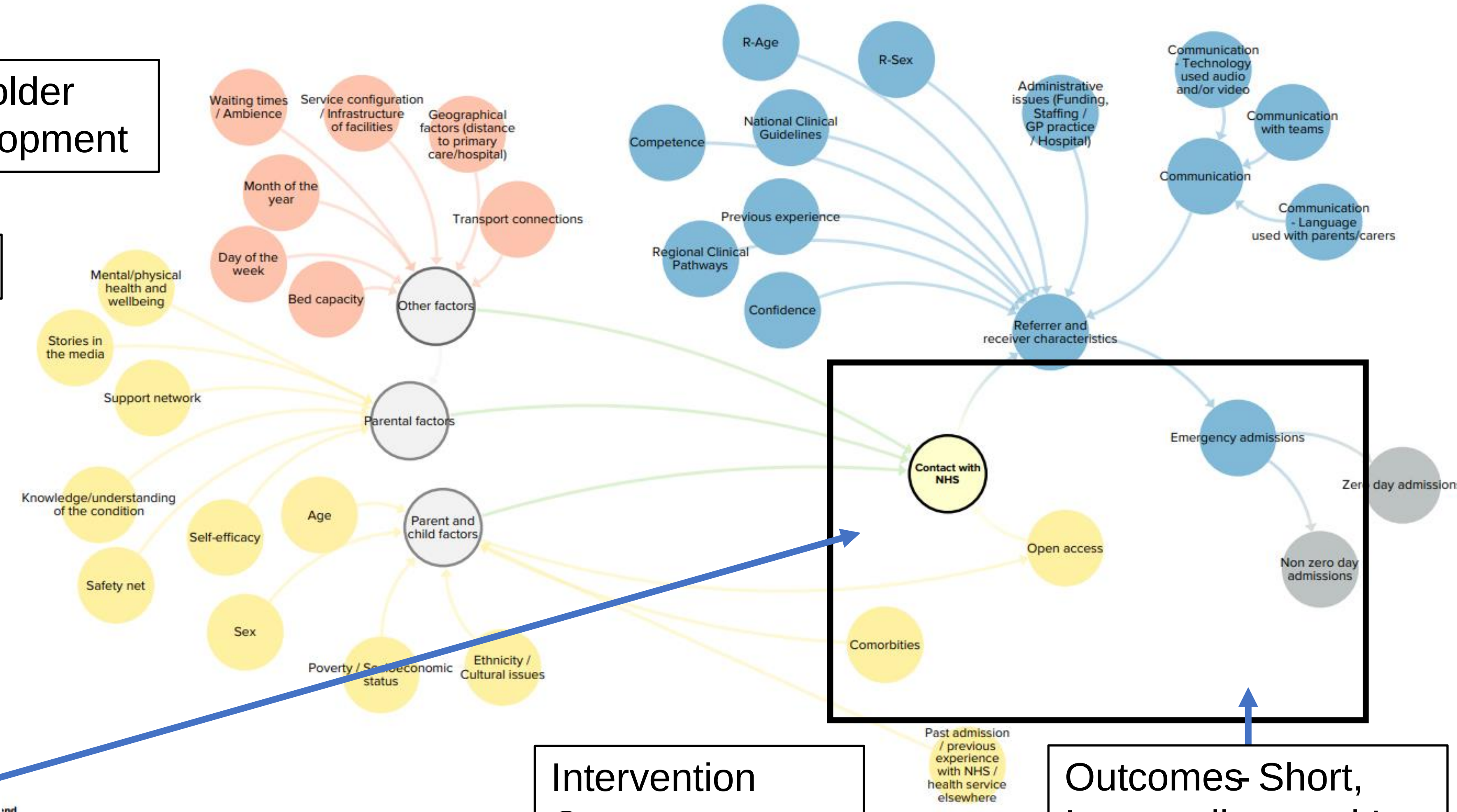

Right method, time, place, person, process,

Family, Staff and Society Values

Intervention Components, Processes and Mechanisms: TIDieR

Outcomes - Short, Intermediate and Long Term that matter to children, families, staff, society

FLAMINGO Revised Logic Model post data analysis

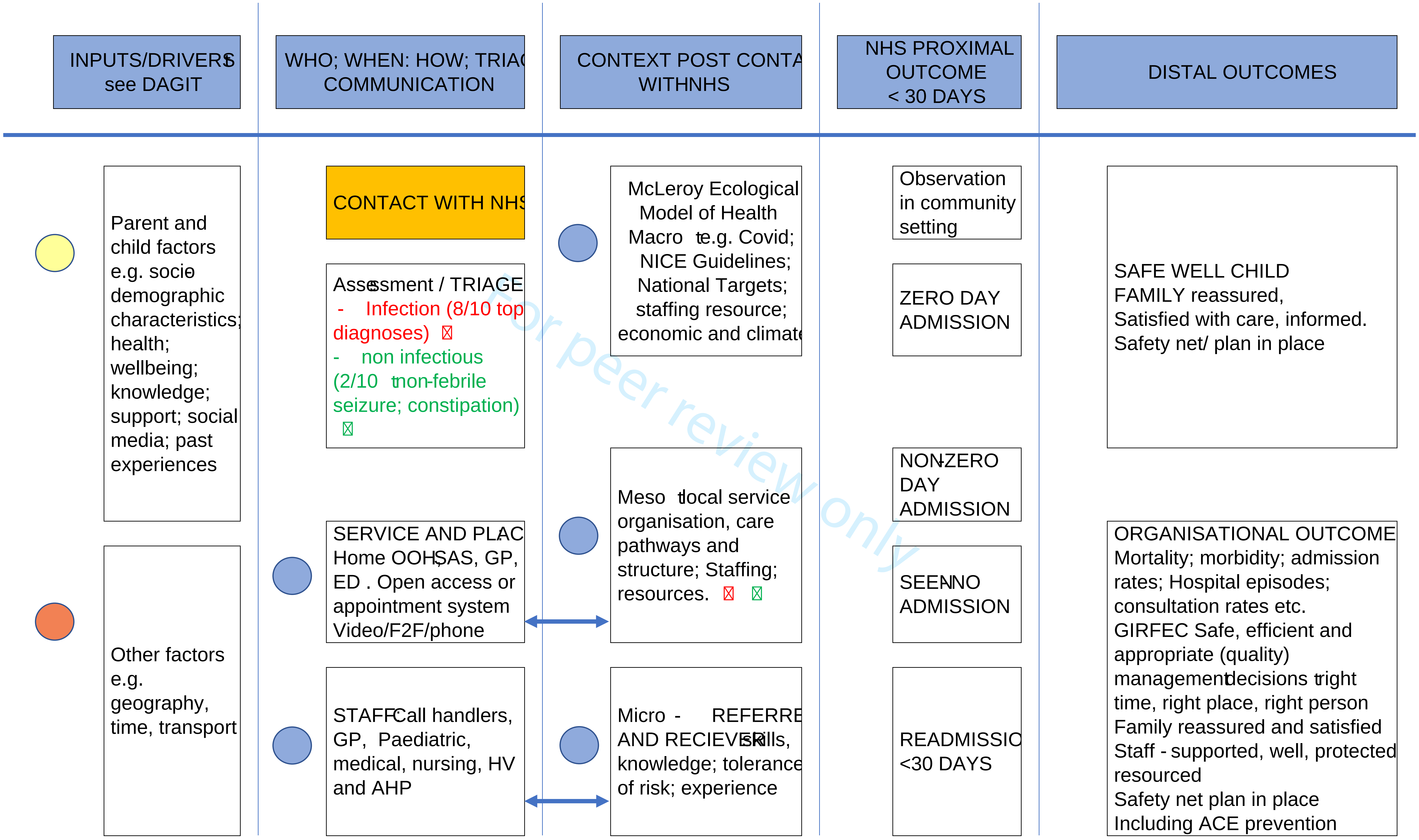

For peer review only

Supplementary file 6. Flamingo stakeholder event methods and materials

Purpose/objective

To present project findings and to discuss prioritisation of potential interventions.

Setting

The five-hour event was held on 3 September 2021 at Edinburgh Zoo, a location accessible for most stakeholders. Travel expenses were offered to families and health professionals who had taken part in interviews.

Participants

The face-to-face and online attendees included parent and health professional interviewees, health professionals, Principle Investigators for the case study sites and a purposive sample of professional contacts of the team with relevant experience, a representative from the study funder the Chief Scientist Office, and representatives from stakeholder organisations e.g. charities and parent groups.

Agenda

10-10.15 Introduction

10.15-11.00* What we found

11-12ish Group discussions followed by time to explore the zoo

12.30-1.15 Lunch

1.15-2.15* Opportunities for change

2.15-2.45 Group discussions

2.45-3.00 Wrap up

*tea and coffee available

Procedures & data collection

The Stakeholder Event discussed FLAMINGO project findings in the morning and prioritisation of potential new interventions in the afternoon. Attendees were split into six groups, five in-person and one online. In-person attendees were seated at pre-allocated tables of five attendees with a mix of families, health professionals and other stakeholders (e.g. third sector or government representatives). One member from the FLAMINGO team (SD, EF, EK, ST, or PW) facilitated each

For peer review only

[illegible] facilitated by two remote [illegible] members of the FLAMINGO team (CM, RK). Facilitators took written notes during or after the discussion and audio-recorded all discussions to assist accurate note-taking. To ensure all contributions were captured, attendees were also asked to write any ideas they had on post-it notes which were collated and recorded. After the morning presentations, facilitators asked each group three questions to stimulate discussion about the study findings:

1. What is the most important thing you have heard this morning?
2. What surprised you the most?
3. What did not surprise you?

In the afternoon the project team presented the seven potential future health systems interventions Subsequently, the groups were asked the following two questions to spark discussion about prioritisation of interventions:

1. If you were in charge, what would you change first and why?
2. Imagine you are in charge five years after your first choice has been in action. What problems [illegible]

The second question drew on Klein and Kahneman & Klein [illegible][1, 2] [illegible] method [illegible] to improve decisions, which asks groups to prospectively imagine the future and predict what has gone wrong; the pre-mortem can overcome the suppression of dissenting views and increases identification of risks [illegible]

Finally, attendees were asked to individually rank the priority of seven interventions during the stakeholder event on a pro-forma, where 1 was the highest priority and 7 was the lowest. Online participants accessed an online pro-forma.

Analysis

Notes taken during discussions were checked for accuracy and completeness against the audio-recordings where possible, however, due to high background noise in two recordings facilitators produced more detailed notes of the discussions from memory and post-it notes were checked and incorporated into the discussion notes. Notes were uploaded into NVivo and were coded (by EF and EK) under broad codes for each of the seven interventions with separate codes for confirming or disconfirming perspectives, plus a further code for other interventions attendees suggested. Codes

For peer review only

were read repeatedly and themes identified. For the intervention priority ranking exercise, scores were summed and the average rank calculated for each of the seven interventions.

1. Klein G. Performing a Project PreMortem. Harvard Business Review. 2007.
2. Kahneman D, Klein G. Conditions for intuitive expertise: A failure to disagree. American Psychologist. 2009;64(4):515-26.

For peer review only

FLAMINGO
Flow of hospital admissions in children and young people - Introduction

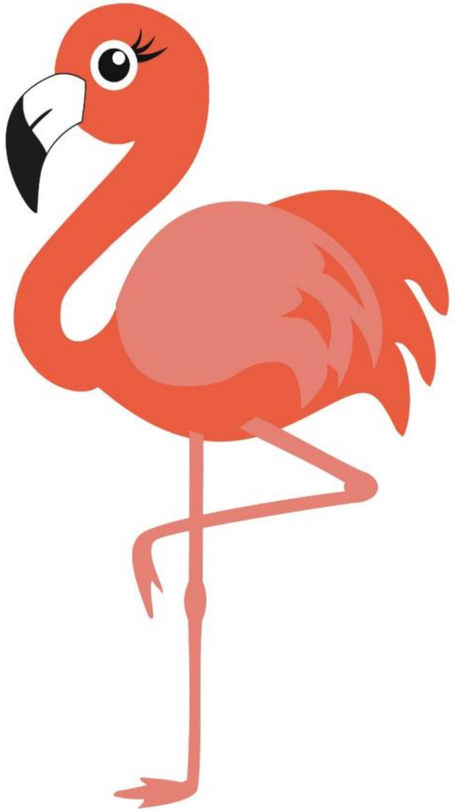

CHIEF SCIENTIST OFFICE

For peer review only - http://bmjopen.bmj.com/site/about/guidelines.xhtml

Good morning. Welcome

- Housekeeping
- Thanks
- Why are we meeting?
- Plan for the day
- Introductions

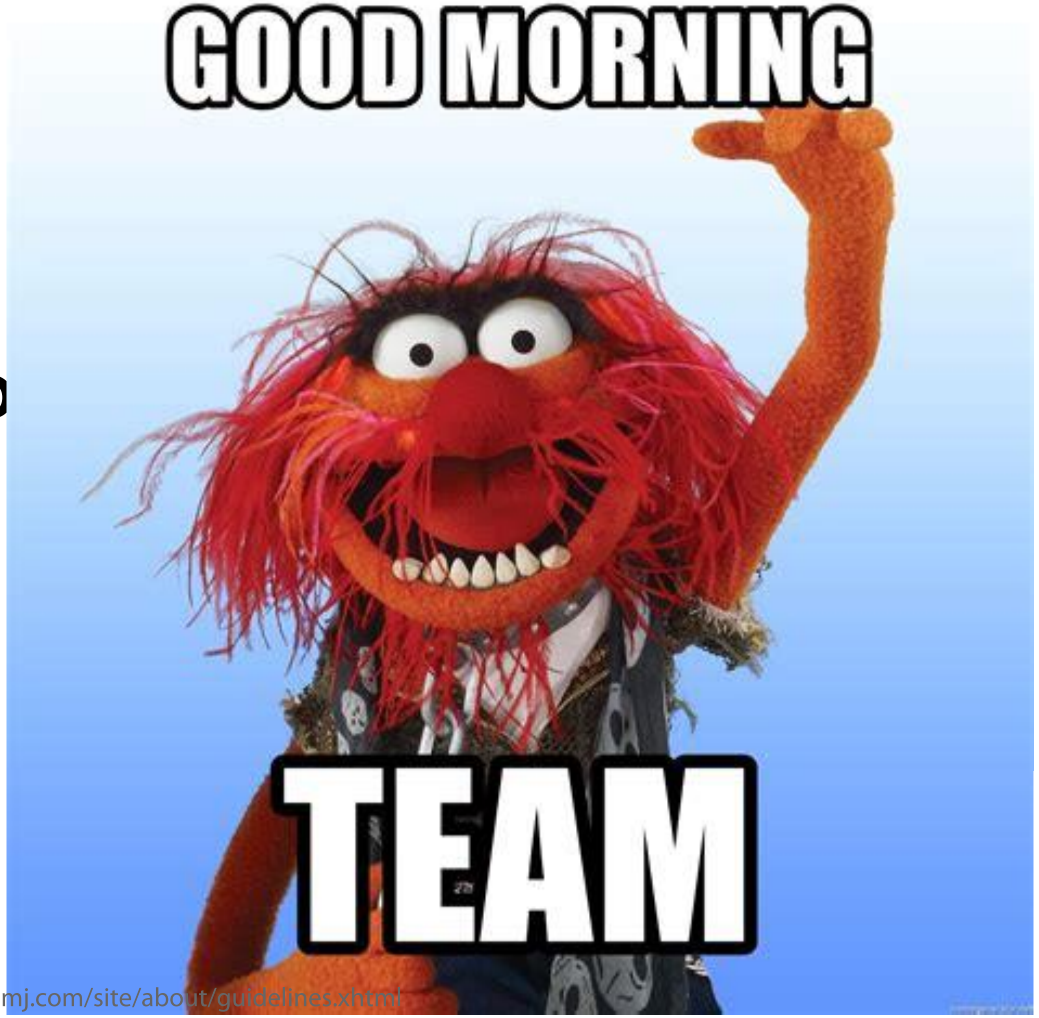

CHIEF SCIENTIST OFFICE

Housekeeping

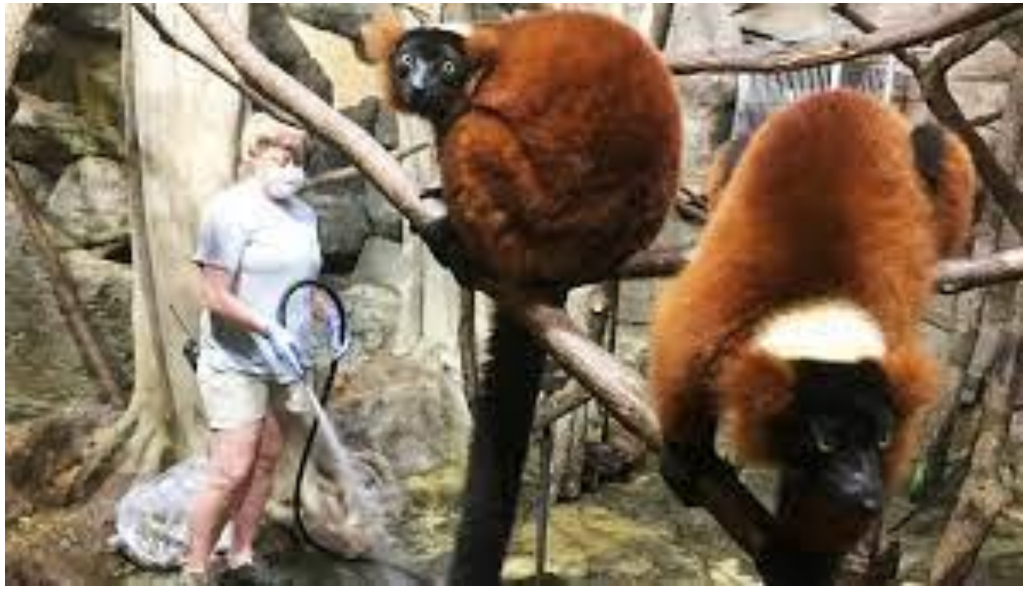

- Fire exits, toilets, mobile phones
- Please don’t share/tweet
- Permission to record (note taking)
- COVID considerations
- Please speak into microphone
- No jargon. Please don’t be afraid to ask for clarification
- Teams. Camera on, microphone on mute please. Raise hand.

CHIEF SCIENTIST OFFICE

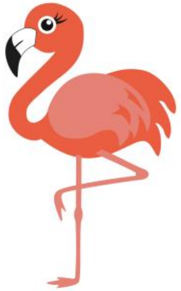

Why are we meeting?

- Zero day admissions rising
- Is there a........
- Win (children)
- Win (parents/carers)
- Win (primary care)
- Win (secondary care)
- Win (NHS Scotland/Government)

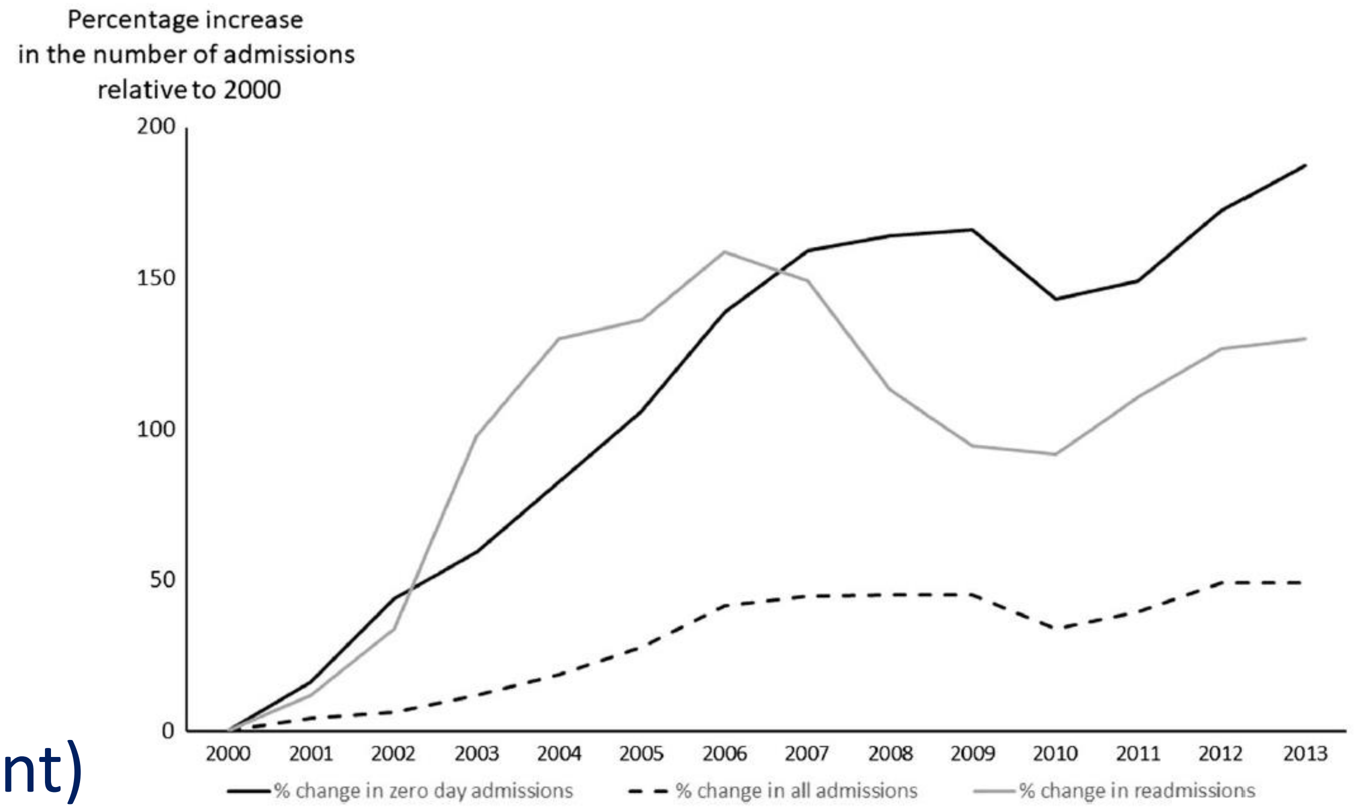

Eur J Pediatr 2018 ;177:381-388

CHIEF SCIENTIST OFFICE

Why are we meeting?

This is stakeholder engagement

Two main aims

1. Share results with you
   - What surprises you
2. What are the priorities?
   - What would you do first?

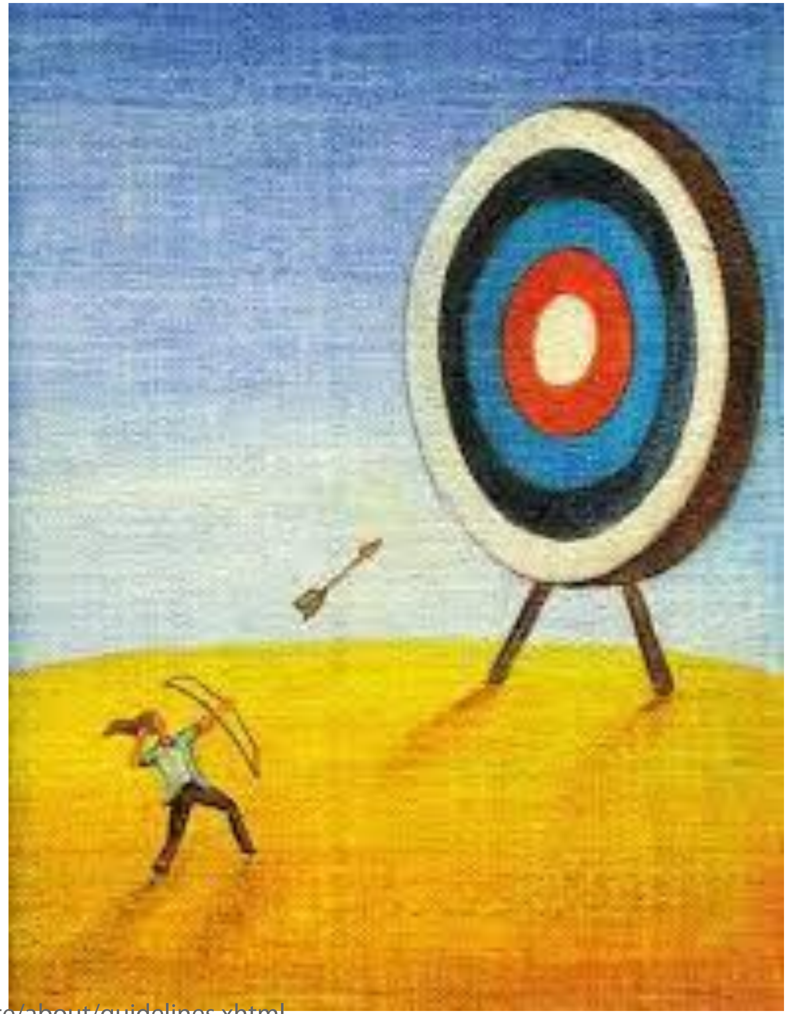

CHIEF SCIENTIST OFFICE

What is the plan for today?

10-10.15 introduction

10.15-11.00* What we found

11-12ish Group discussions, zoo

12.30-1.15 Lunch

1.15-2.15* Opportunities for change

2.15-2.45 Group discussions

2.45-3.00 Wrap up

*

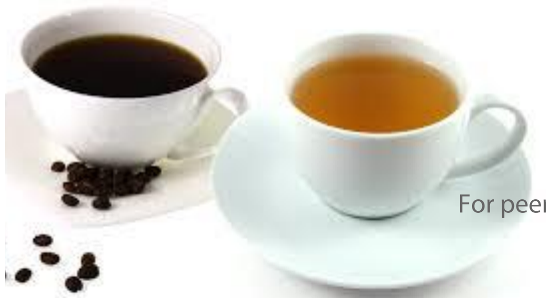

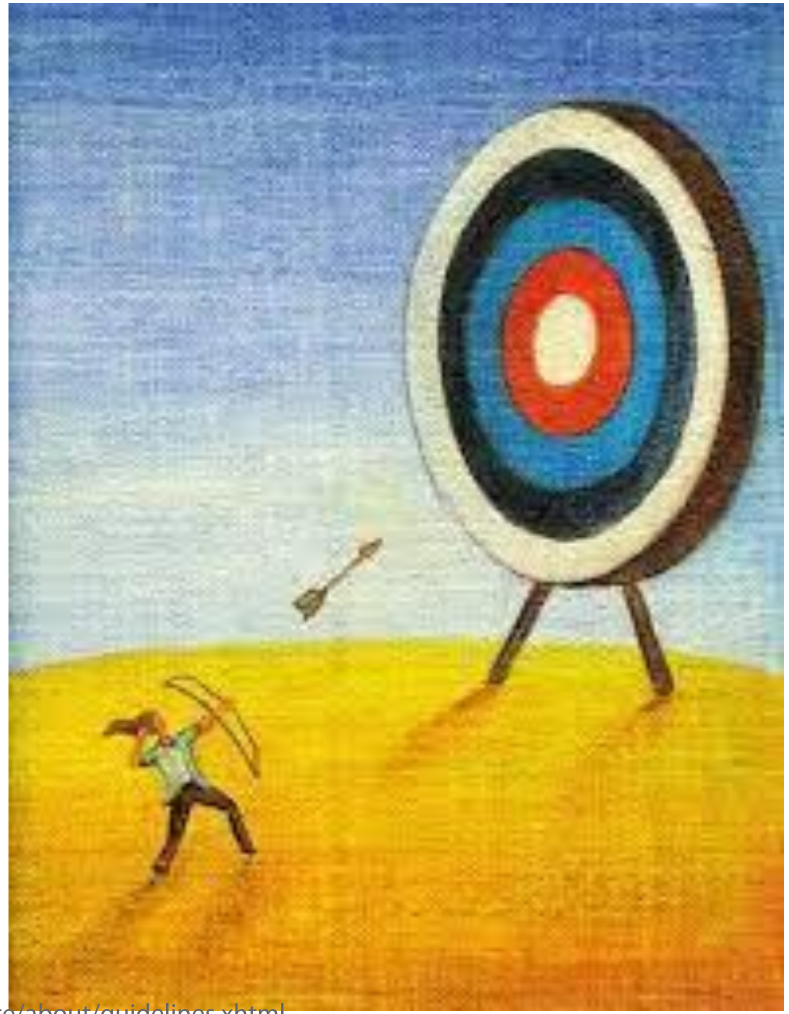

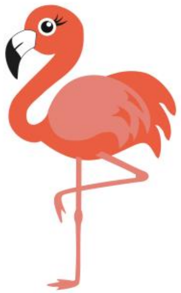

CHIEF SCIENTIST OFFICE

Introductions

- Who is here today?

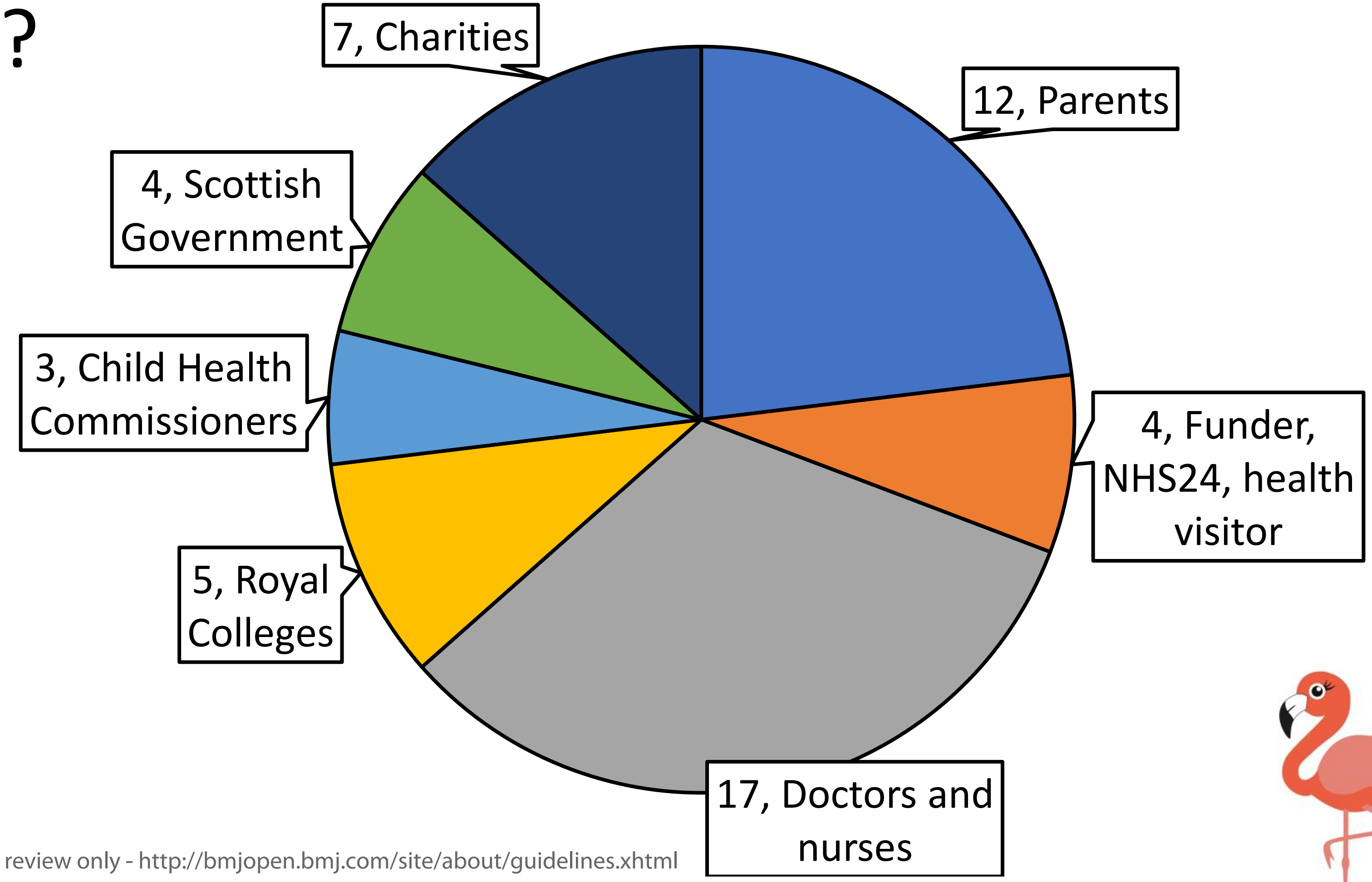

CHIEF SCIENTIST OFFICE

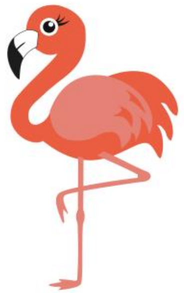

Introductions

- ~35+5 in person
- ~19+2 on Teams
- Some just for am/pm
- ?no reps from
  - Orkney
  - Western Isles
  - Tayside
  - D&G
  - Forth Valley
  - Lanarkshire

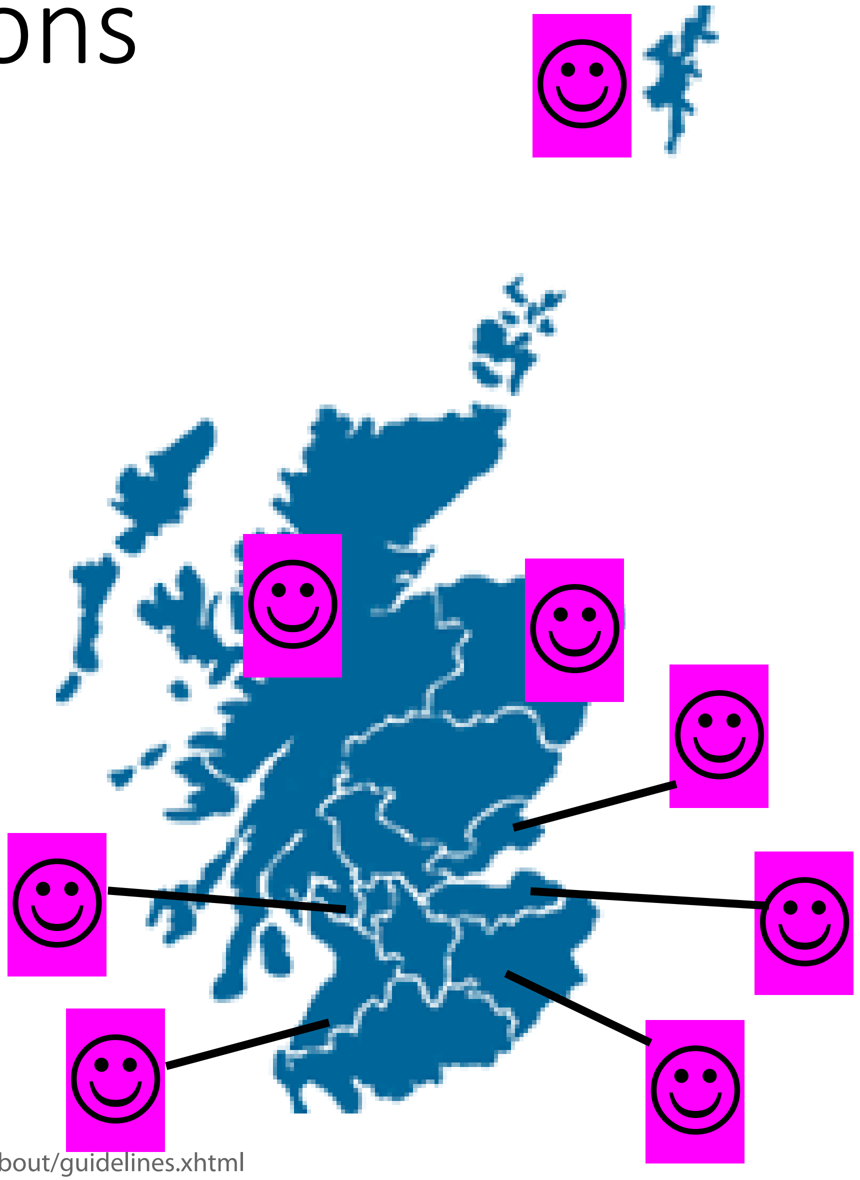

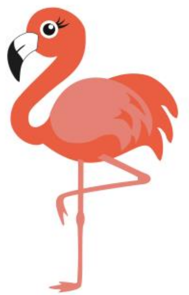

CHIEF SCIENTIST OFFICE

Introductions

Please spend a few minutes

At the Zoo:

- Name
- Which group you are representing
- Why are you here?

Teams:

Please put this in the chat

Note to self:

What do you want to achieve by 3pm?

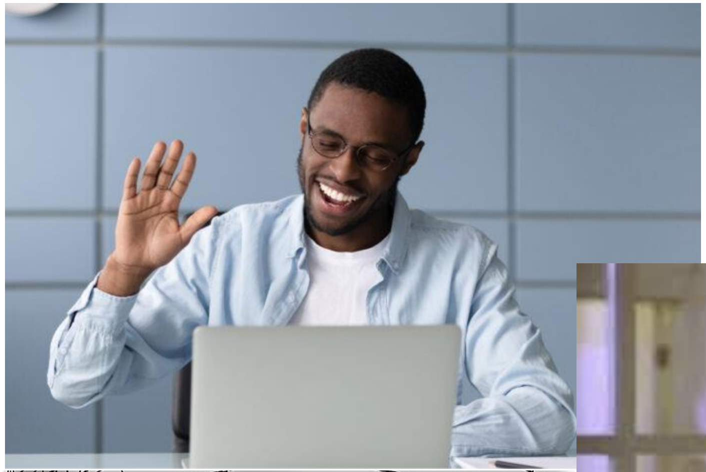

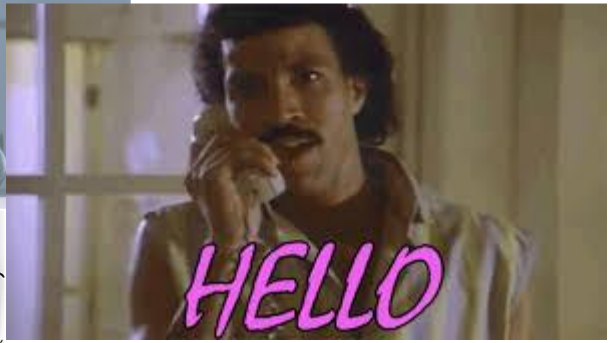

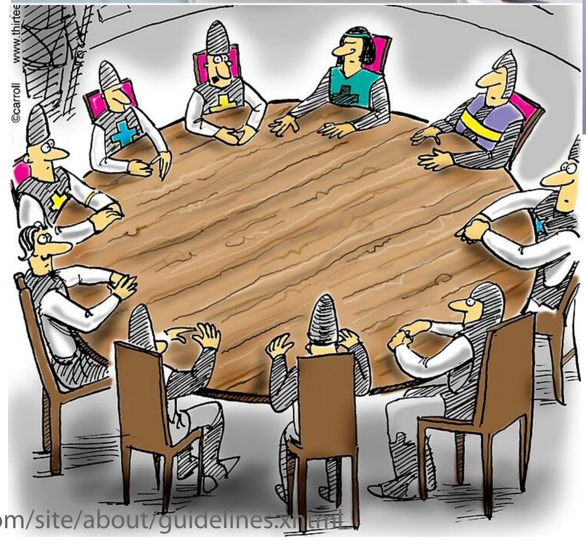

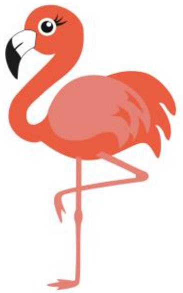

CHIEF SCIENTIST OFFICE

FLAMINGO

Flow of hospital admissions in children and young people – Methods and Results

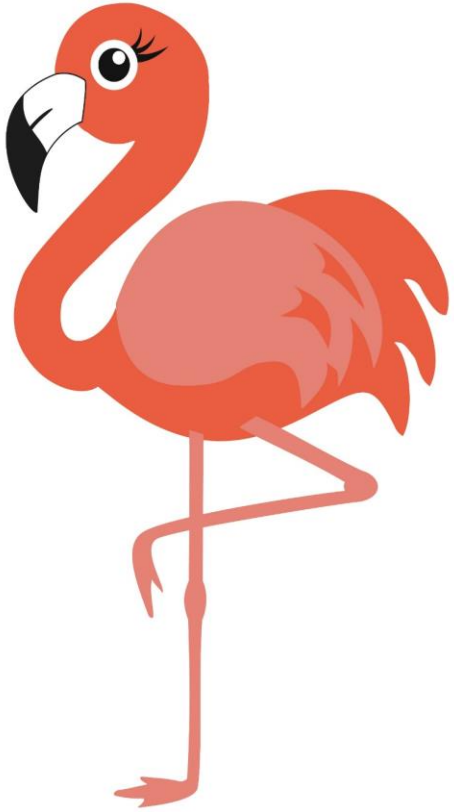

CHIEF SCIENTIST OFFICE

Overview

- Definitions
- Why we did this
- How we did this
- What we found

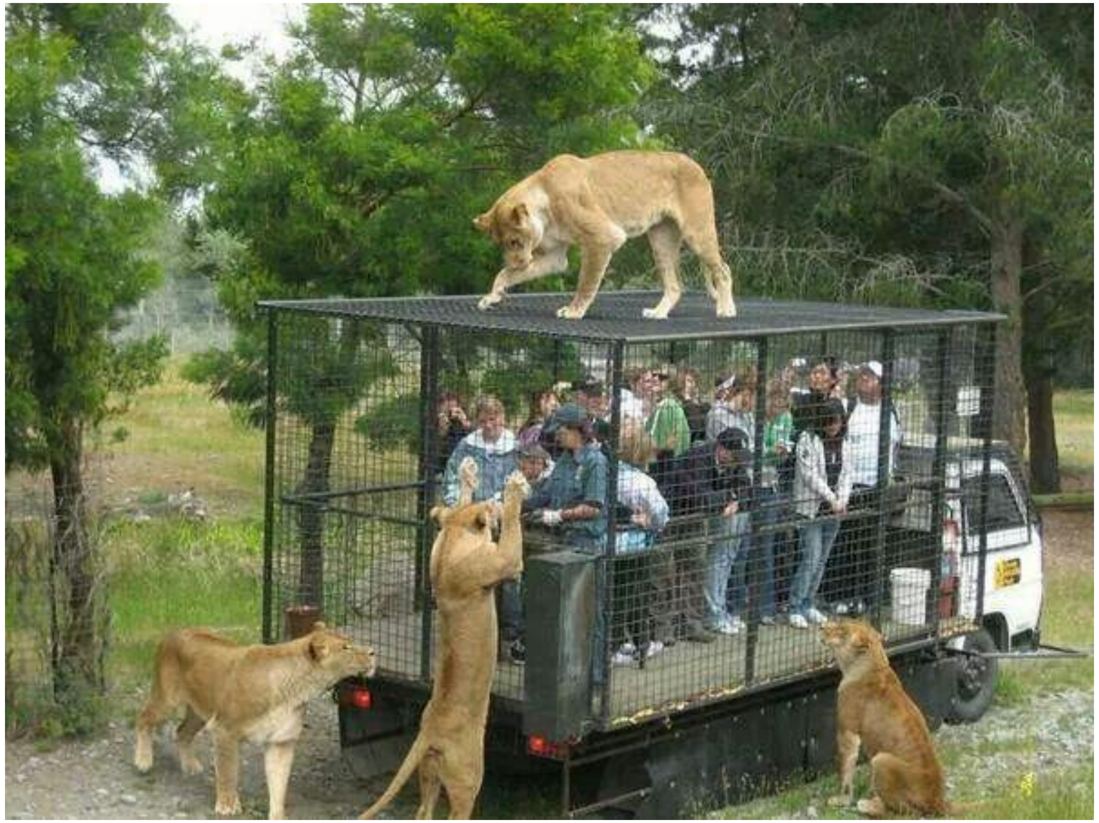

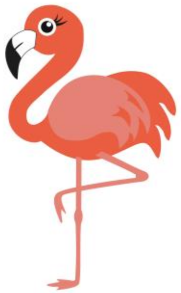

CHIEF SCIENTIST OFFICE

Definitions

- Emergency department
- General practice
- NHS 24
- Out of Hours
- Health board
- Admission
- Zero day admission

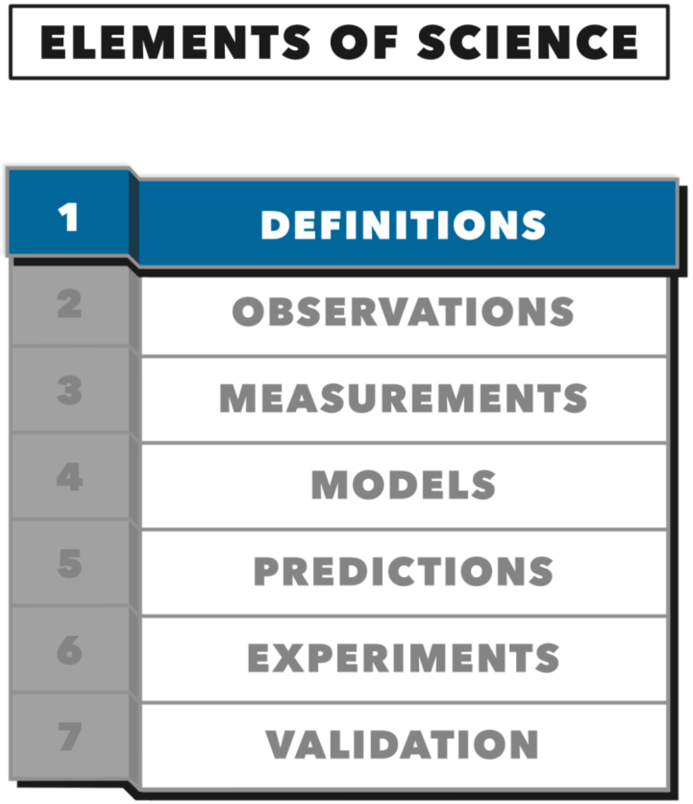

CHIEF SCIENTIST OFFICE

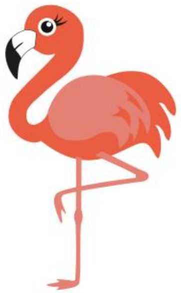

Why

Risers and fallers?

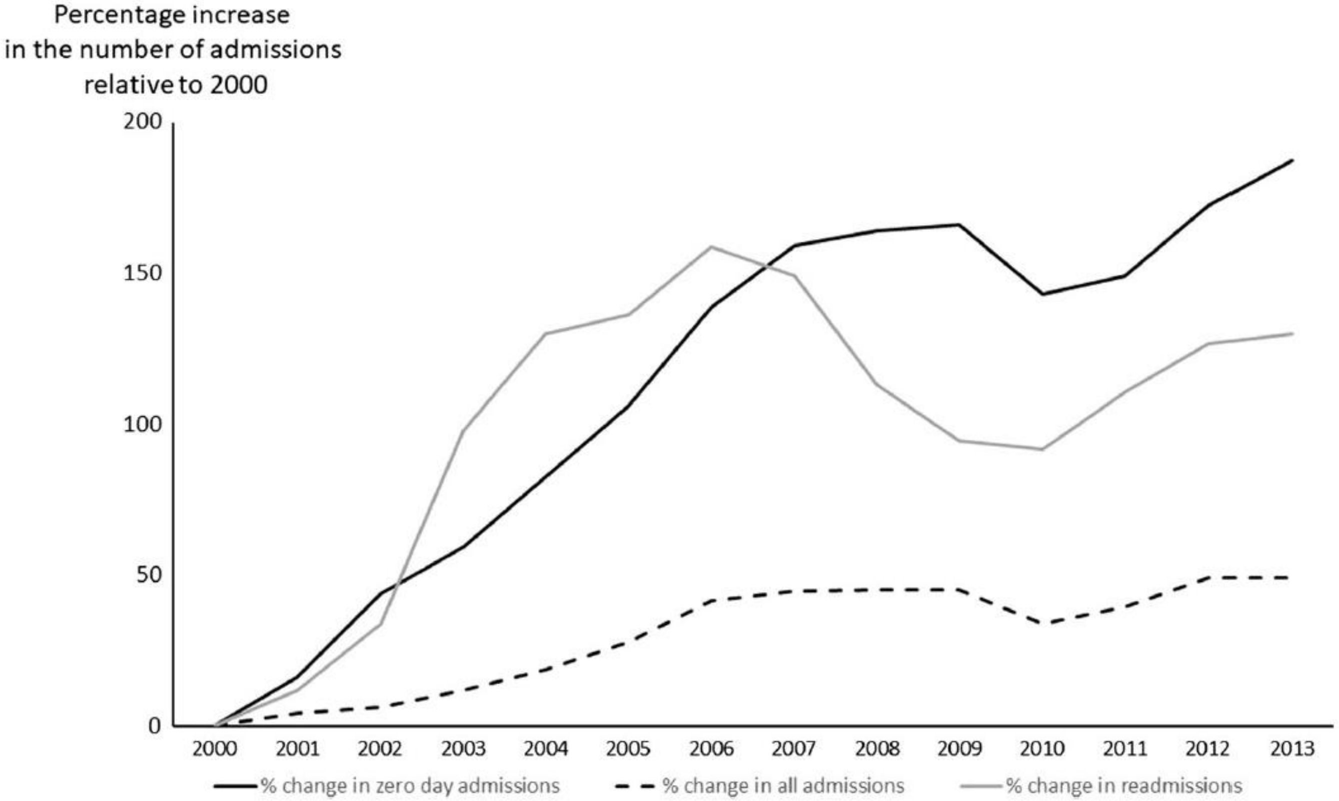

Eur J Pediatr 2018 ;177:381-388

CHIEF SCIENTIST OFFICE

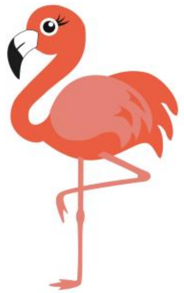

Why

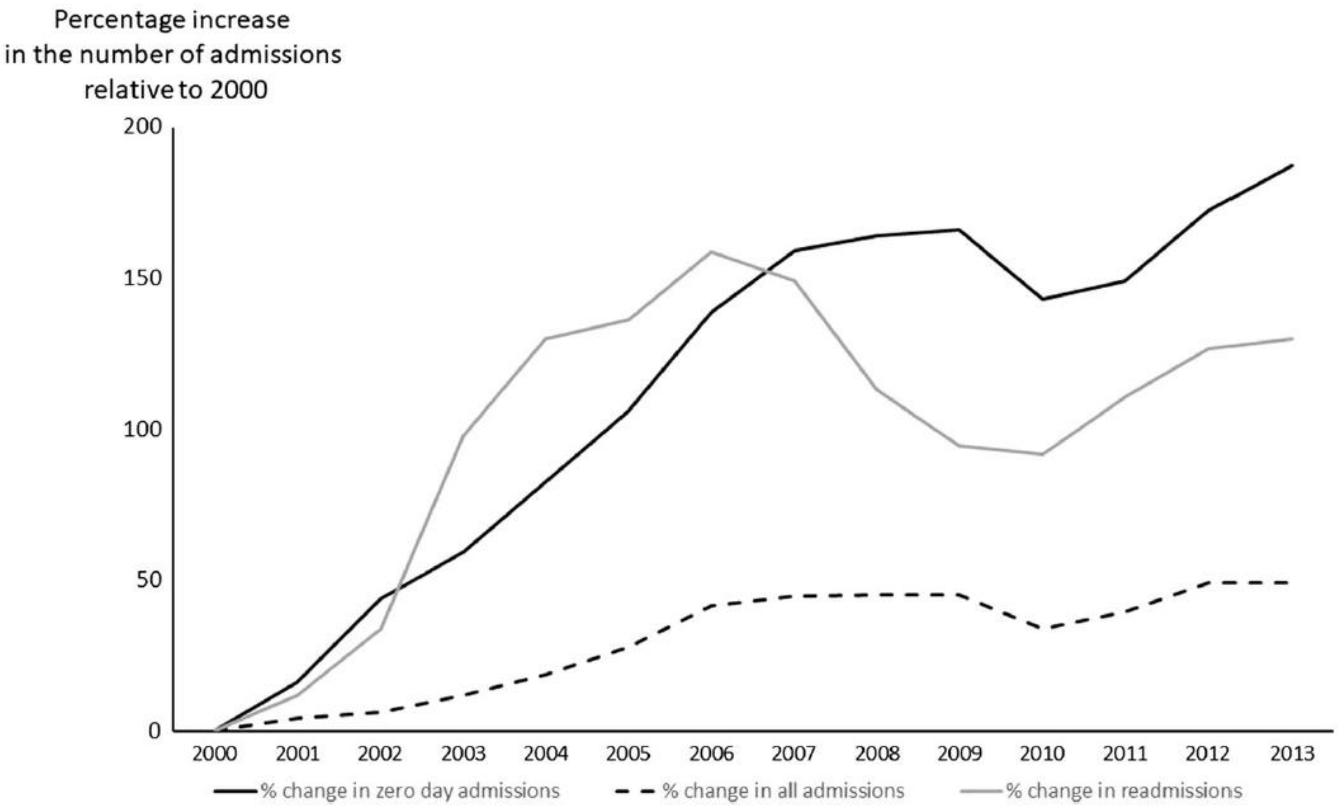

Eur J Pediatr 2018 ;177:381-388

Risers and fallers

↑ 100% "viral infection"

↑45% tonsillitis

↑25% bronchiolitis

↑10% chest infection

↓15% croup

↓15% gastroenteritis

↓25% convulsions

↓25% asthma

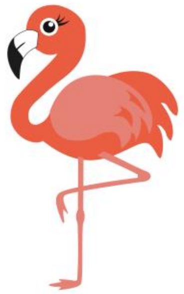

CHIEF SCIENTIST OFFICE

Why

- True change
  - Asthma, convulsions
- Change in parent help-seeking
  - Poverty
  - Expectations
- Change in primary care
- Change in hospital
- All of the above!

Risers and fallers:

↑ 100% “viral infection”

↑45% tonsillitis

↑25% bronchiolitis

↑10% chest infection

↓15% croup

↓15% gastroenteritis

↓25% convulsions

↓25% asthma

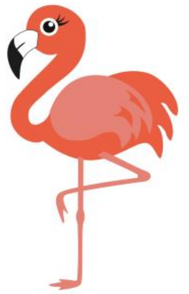

CHIEF SCIENTIST OFFICE

Why

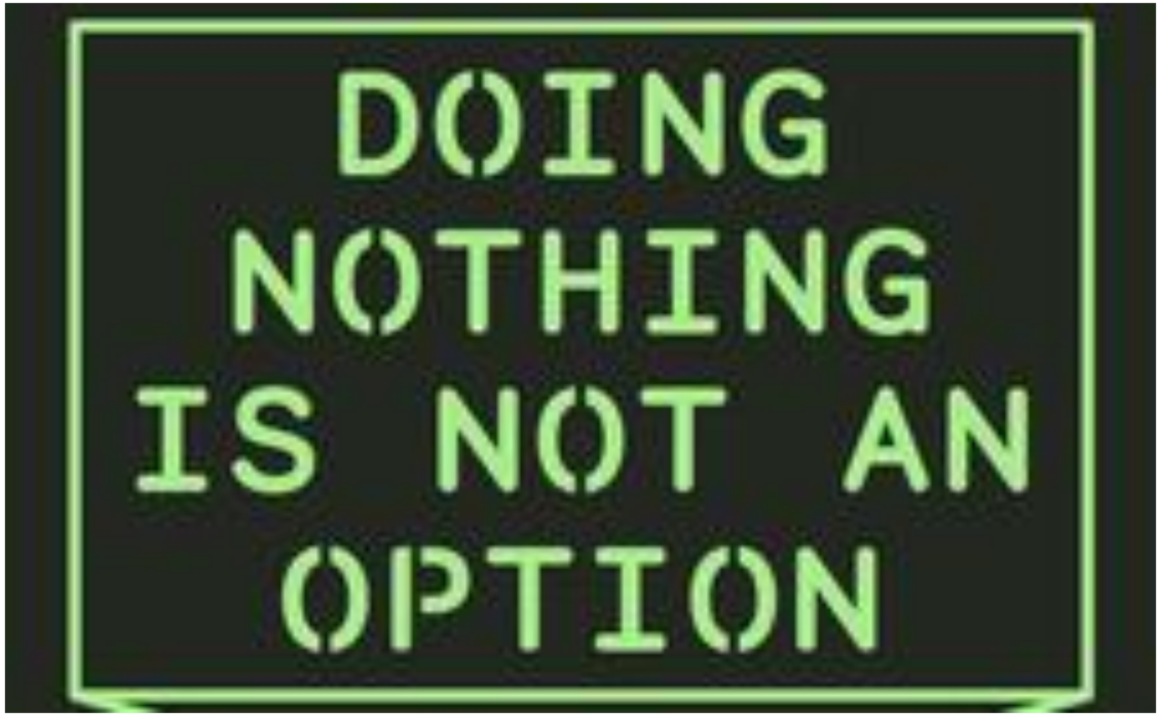

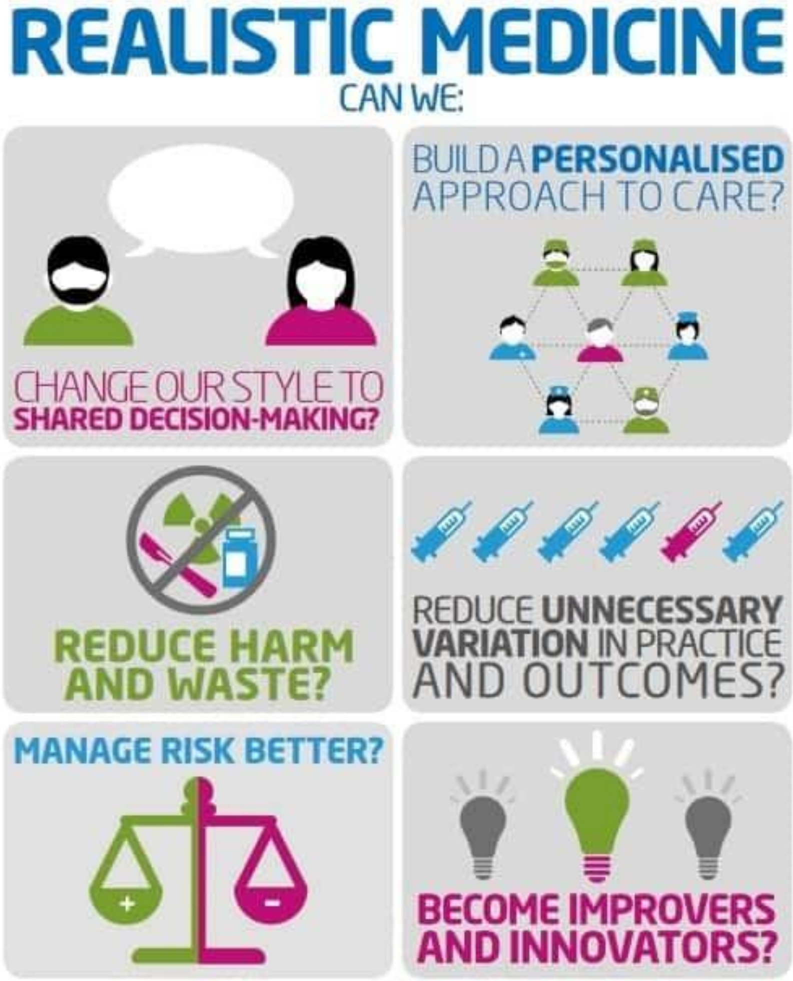

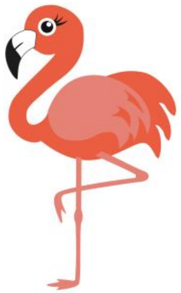

CHIEF
SCIENTIST
OFFICE

Question

1. How can we best involve parents/carers and children in research to improve emergency paediatric admission pathways?

CHIEF SCIENTIST OFFICE

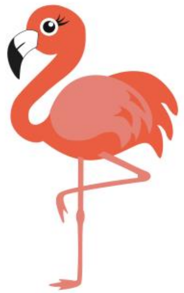

[illegible]

1. How can we best involve parents/carers and children in research to improve emergency paediatric admission pathways?

1. Work with parents (mother-baby clubs, hospitals). Pre-COVID.

CHIEF SCIENTIST OFFICE

For peer review only - http://bmjopen.bmj.com/site/about/guidelines.xhtml

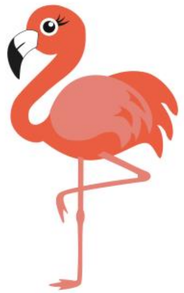

[illegible]

1. How can we best involve parents/carers and children in research to improve emergency paediatric admission pathways?
2. Within the 'flow' of zero day admissions, are there identifiable 'streams' of zero day admissions which could be slowed?

1. Work with parents (mother-baby clubs, hospitals). Pre-COVID.

CHIEF SCIENTIST OFFICE

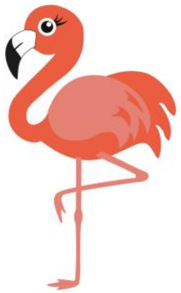

[illegible]

1. How can we best involve parents/carers and children in research to improve emergency paediatric admission pathways?
2. Within the ‘flow’ of zero day admissions, are there identifiable ‘streams’ of zero day admissions which could be slowed?

1. Work with parents (mother-baby clubs, hospitals). Pre-COVID.
2. Collect data (numbers and words).

CHIEF SCIENTIST OFFICE

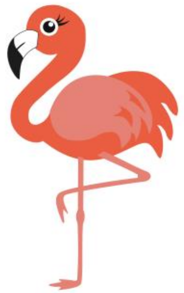

For peer review only - http://bmjopen.bmj.com/site/about/guidelines.xhtml

[illegible]

1. How can we best involve parents/carers and children in research to improve emergency paediatric admission pathways?
2. Within the ‘flow’ of zero day admissions, are there identifiable ‘streams’ of zero day admissions which could be slowed?
3. Which of these ‘streams’ is the most important to slow down first?

1. Work with parents (mother-baby clubs, hospitals). Pre-COVID.
2. Collect data (numbers and words).

CHIEF SCIENTIST OFFICE

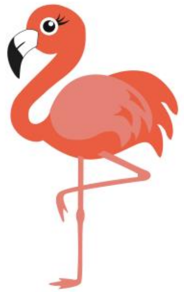

For peer review only - http://bmjopen.bmj.com/site/about/guidelines.xhtml

[illegible]

1. How can we best involve parents/carers and children in research to improve emergency paediatric admission pathways?
2. Within the ‘flow’ of zero day admissions, are there identifiable ‘streams’ of zero day admissions which could be slowed?
3. Which of these ‘streams’ is the most important to slow down first?

1. Work with parents (mother-baby clubs, hospitals). Pre-COVID.
2. Collect data (numbers and words).
3. Meeting at the zoo.

CHIEF SCIENTIST OFFICE

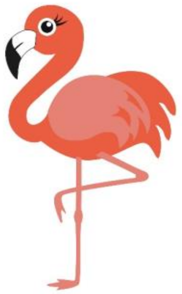

Numbers and words

Numbers = what is happening (quant)

Words = why it is happening (qual)

Mixed methods, sum>parts

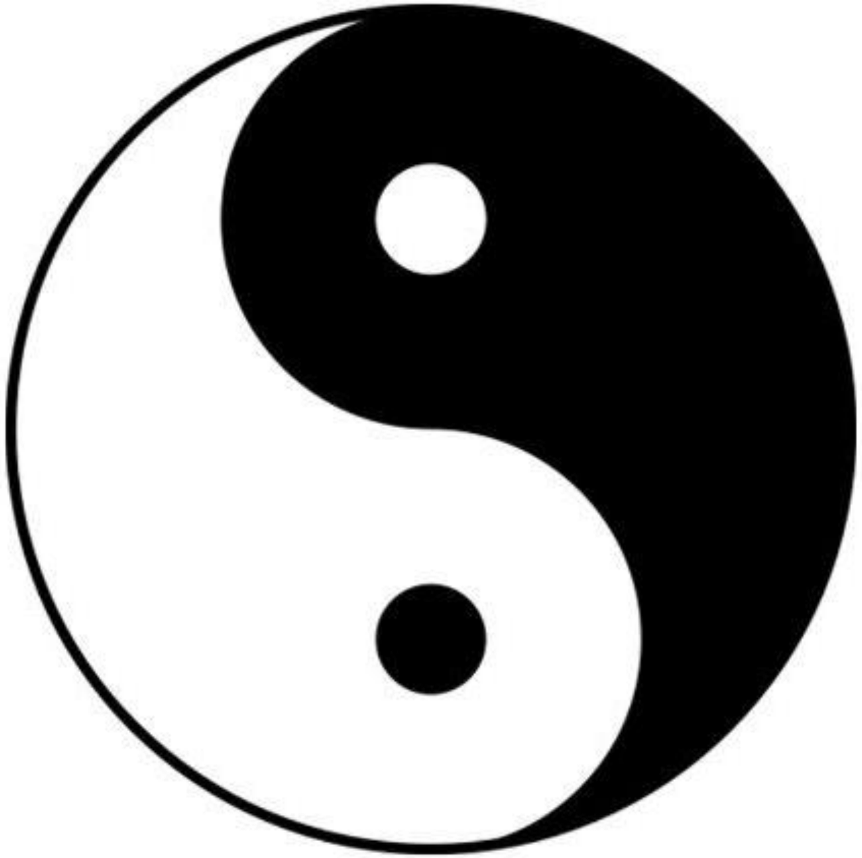

CHIEF SCIENTIST OFFICE

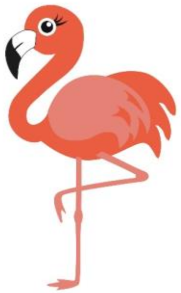

Qualitative Methods

Development of research questions

Visiting a number of parent and toddler groups

Health boards case studies

Five health boards chosen for diversity of geography and population size

Recruitment

Social media, posters, sharing by stakeholder groups, emails to contacts

Telephone interviews

Coding and analysis

Transcription. Coded and analysed by the qualitative team

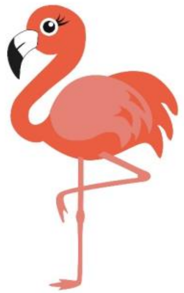

CHIEF SCIENTIST OFFICE

Quantitative Methods

- NHS Scotland collects data
- Collect data from 3 routes into hospital
- 2015-2017
- Link them to map the journey
- Identify “streams” within the “flow”
- Permissions

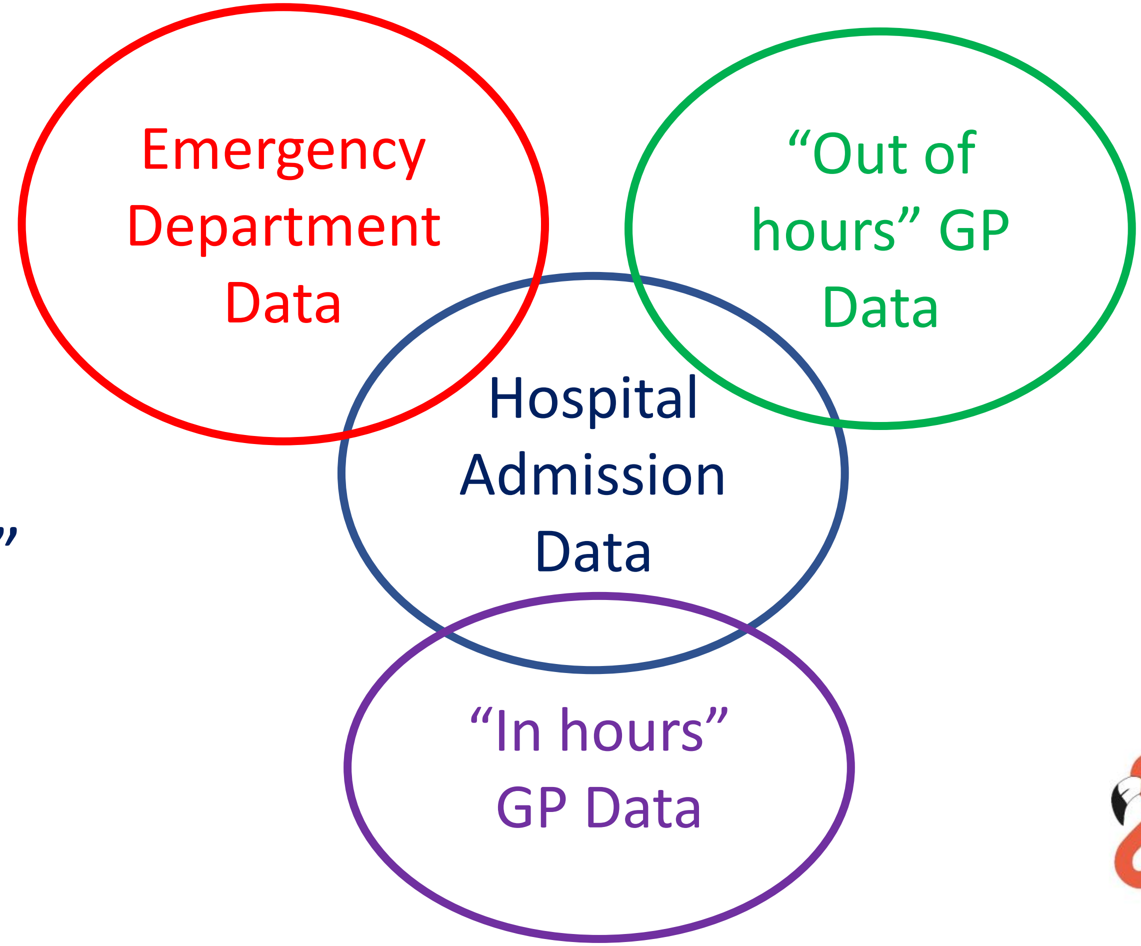

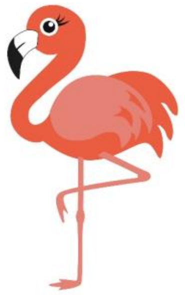

CHIEF SCIENTIST OFFICE

Quantitative Results

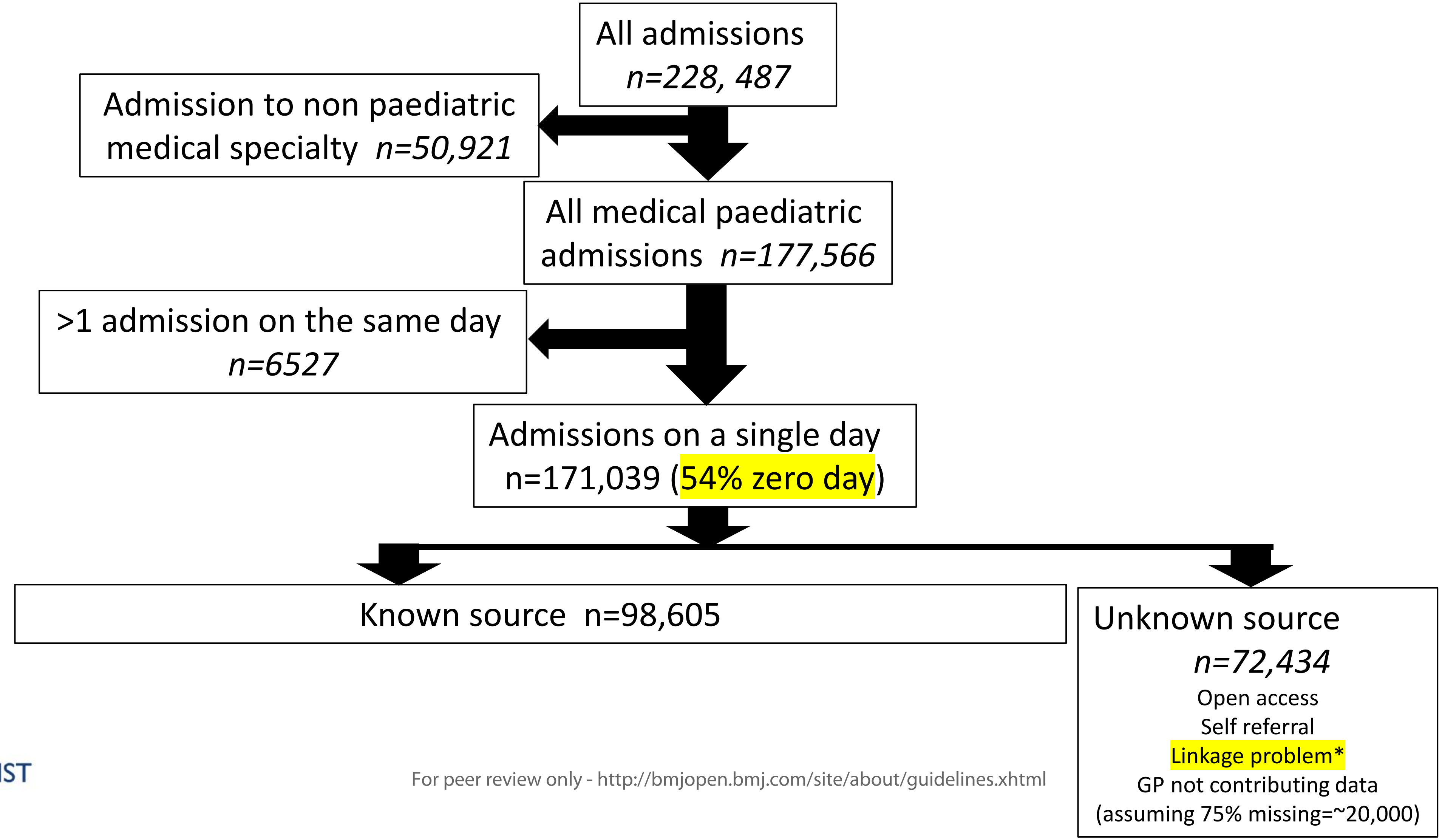

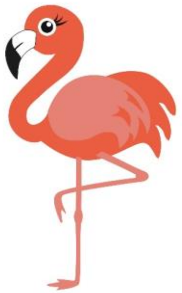

CHIEF SCIENTIST OFFICE

Quantitative Results

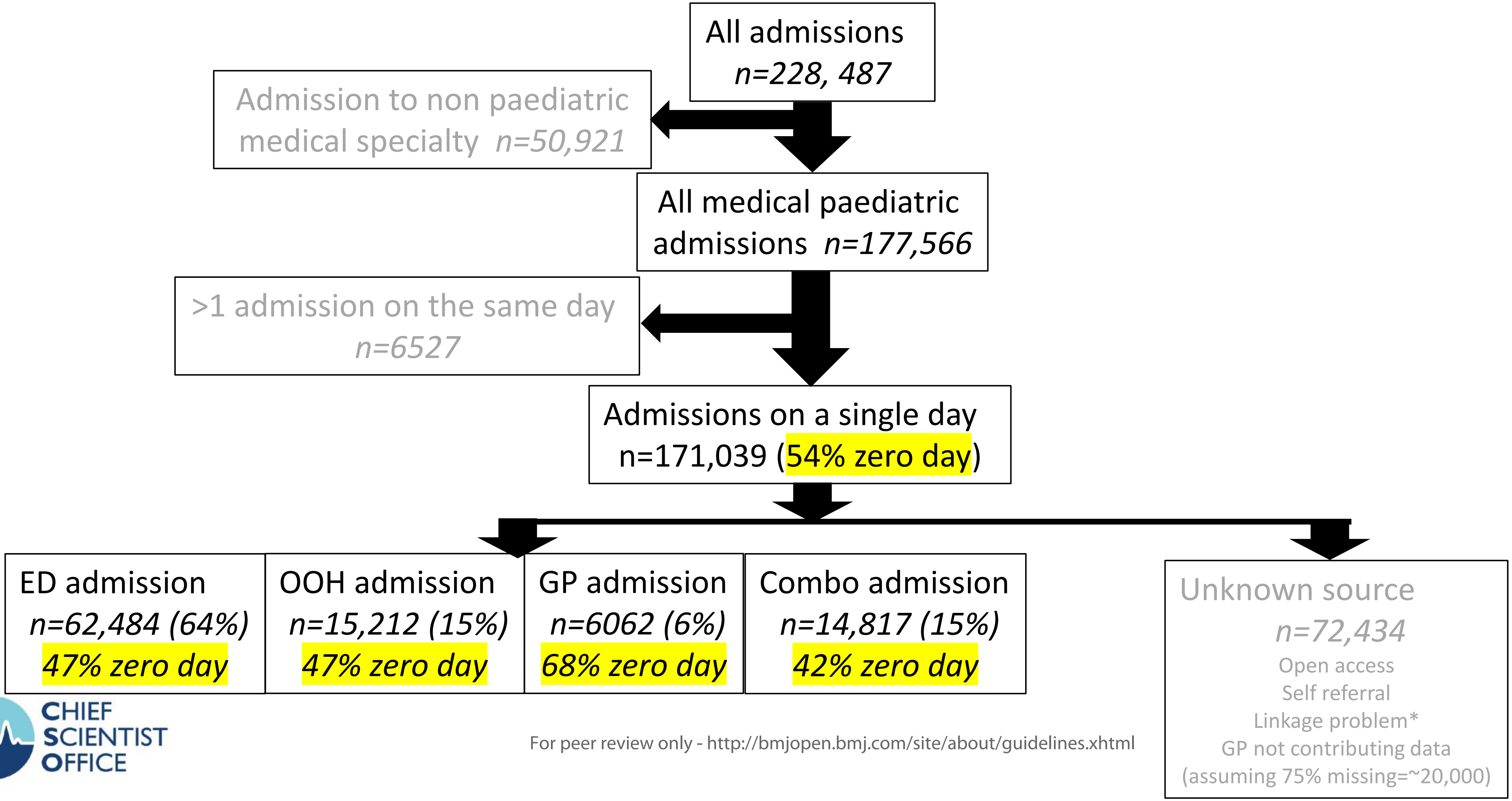

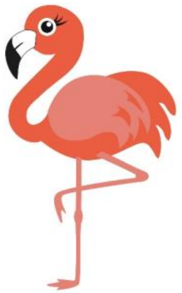

Quantitative Results

- What about regional variation?
- Conditions don't recognise boundaries
- Can this reflect differences in journeys through NHS?

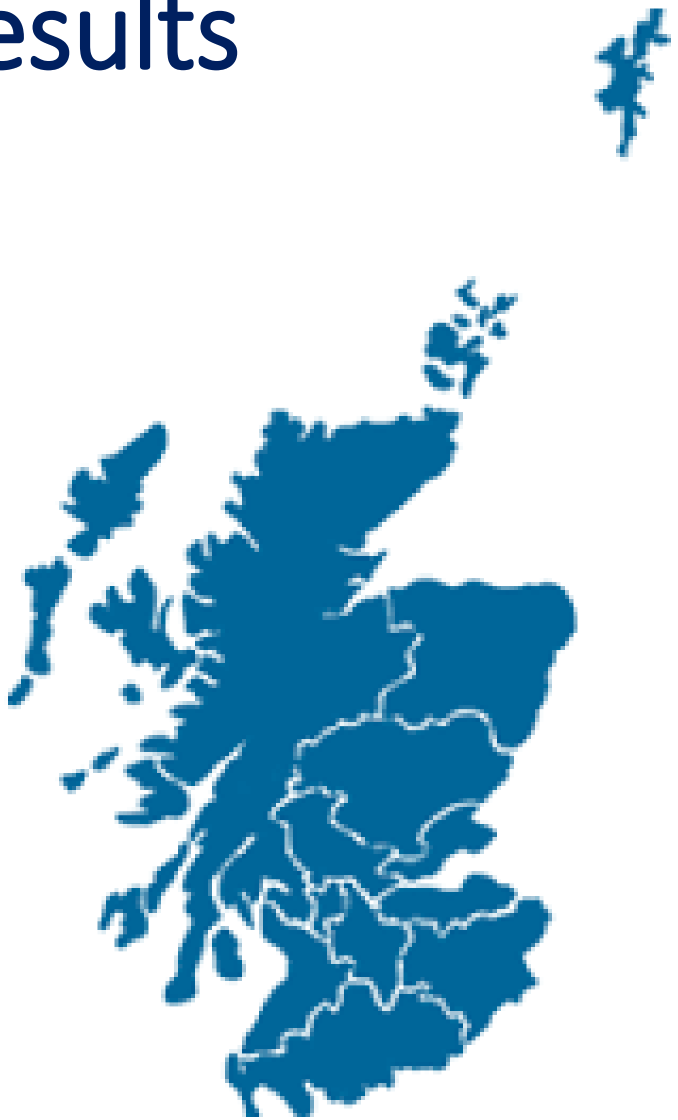

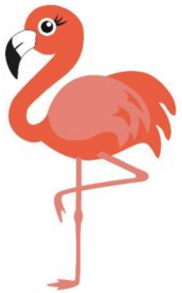

CHIEF SCIENTIST OFFICE

Quantitative Results

- What about regional variation?
- Conditions don't recognise boundaries
- Can this reflect differences in journeys through NHS?

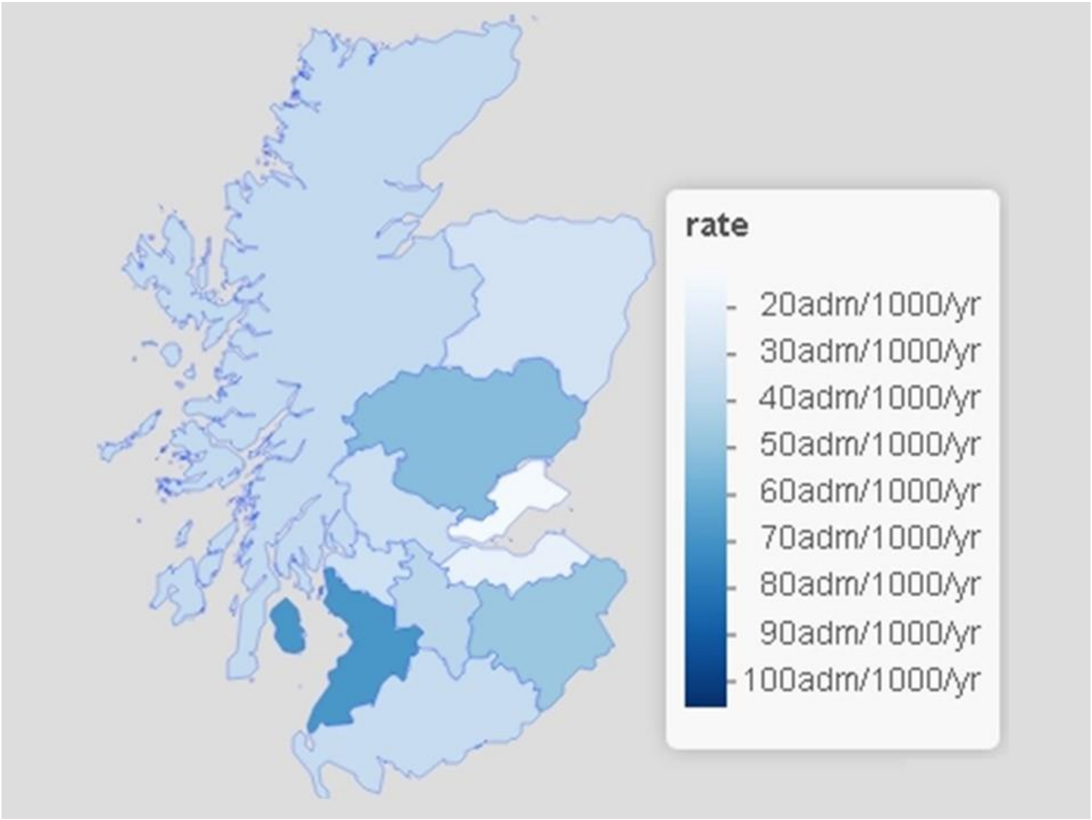

CHIEF SCIENTIST OFFICE

Quantitative Results

Lower than average form this source. Higher than average from this source

Zero day admissions	GP 8.9%	OOH 15.2%	ED 62.5%	Combo 13.4%
Asthma (n=2148)	5.7%	14.3%	68.7%	11.3%
Bronchiolitis (n=6295)	10.1%	18.1%	56.3%	15.4%
Croup (n=2799)	5.2%	14.1%	64.3%	16.4%
Afebrile convulsion (n=1344)	2.3%	2.1%	87.3%	8.3%
Febrile convulsion (n=976)	1.6%	2.2%	84.3%	11.9%
Chest infection (n=2552)	10.4%	20.3%	56.5%	12.8%
Tonsillitis (n=3706)	9.2%	21.0%	54.5%	15.4%
URTI (n=8165)	7.2%	18.5%	58.3%	16.0%
Viral infection (n=10,236)	7.8%	18.1%	58.7%	15.4%

CHIEF SCIENTIST OFFICE

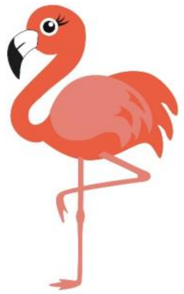

Quantitative Results

Lower than average form this source. Higher than average from this source

Zero day admissions	GP [illegible]	[illegible]	ED [illegible]	Combo 13.4%
Asthma (n=2148)	[illegible]	[illegible]	[illegible]	11.3%
Bronchiolitis (n=6295)	[illegible]	[illegible]	[illegible]	15.4%
Croup (n=2799)	[illegible]	[illegible]	[illegible]	16.4%
Afebrile convulsion (n=1344)	2.3%	[illegible]	[illegible]	8.3%
Febrile convulsion (n=976)	1.6%	2.2%	[illegible]	11.9%
Chest infection (n=2552)	10.4%	20.3%	56.5%	[illegible]
Tonsillitis (n=3706)	9.2%	21.0%	54.5%	15.4%
URTI (n=8165)	7.2%	18.5%	58.3%	16.0%
Viral infection (n=10,236)	7.8%	18.1%	58.7%	15.4%

All very interesting but what is the story behind the numbers?

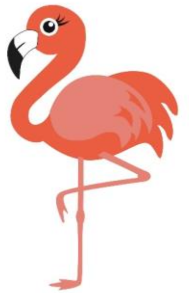

CHIEF SCIENTIST OFFICE

Quantitative Results

- Match cases and controls
- What “discretionary factors” differ
- Time of presentation (0-6am highest)
- Causation not proven!

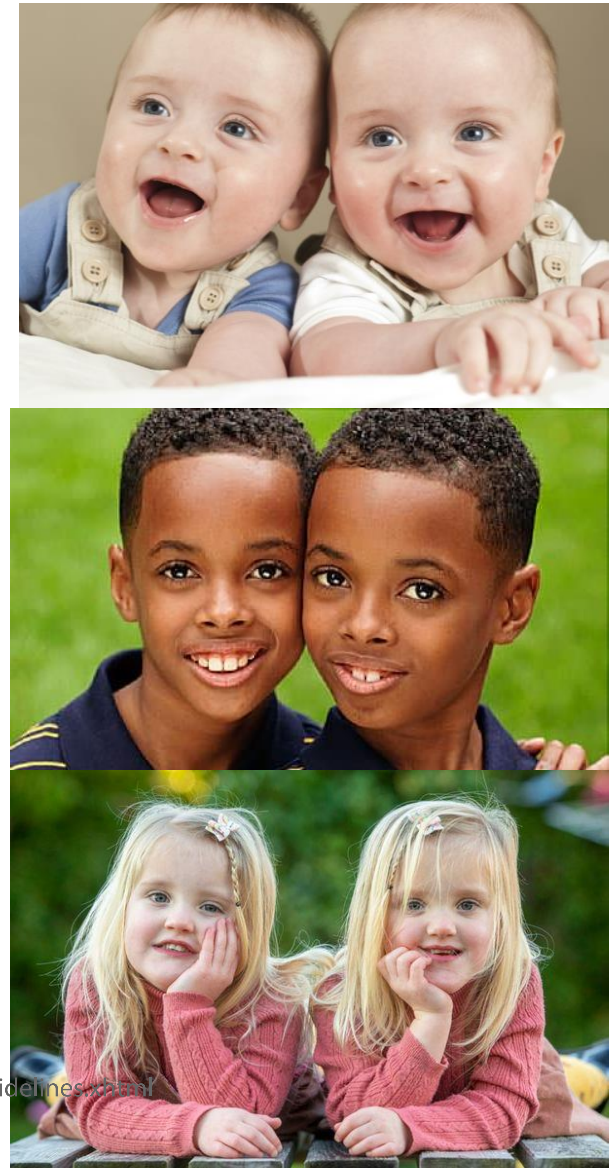

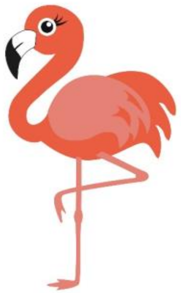

CHIEF SCIENTIST OFFICE

For peer review only - http://bmjopen.bmj.com/site/about/guidelines.xhtml

Quantitative Results

Summary

- There are streams within the flow
- Zero day admissions vary by
  - Referral source
  - Geography
  - Time of presentation
- Deprivation gradient as for all admissions
  - 30% vs 13%
- Time for some interviews!

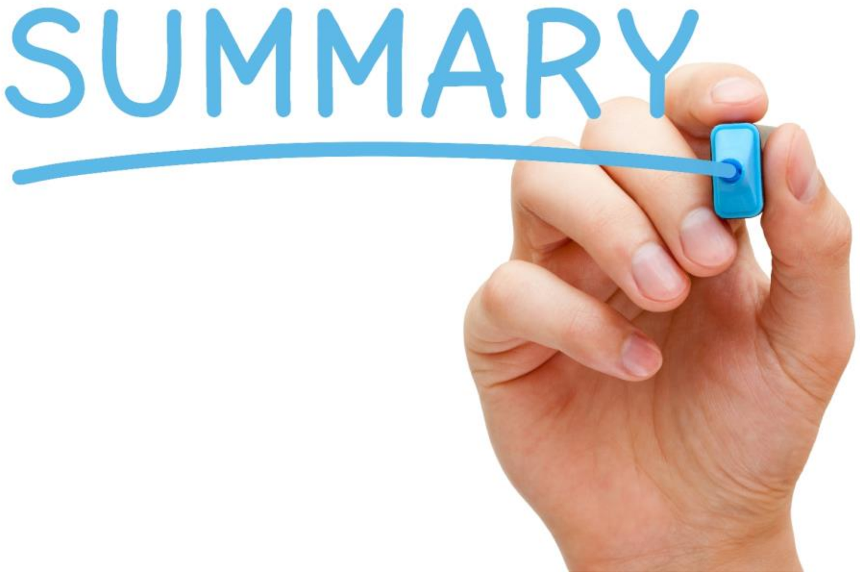

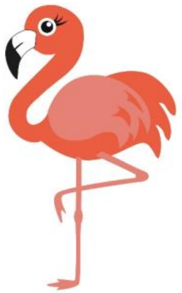

CHIEF SCIENTIST OFFICE

Research interview participants

Type of participant	GP	Nurse (community)	Doctor (hospital)	Consultant (hospital)	Nurse (hospital)	Families	Total
**No.**	10	5	7	10	16	21	**69**

CHIEF SCIENTIST OFFICE

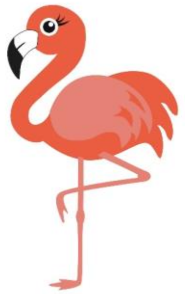

Recording admissions

Consultant

It gets terribly confusing - how do you actually count referrals, admissions, turnarounds whatever? The way our unit is set up, the assessment unit has got X beds in it and it is physically, geographically next door to the ward. And I think if, for whatever reason, the child is transferred through to the ward, then that becomes an admission. Otherwise it is not counted as an admission. The child will be sent through to the ward because there's no space in the assessment unit to let them sit for three or four hours but then they're sent home from the ward after another couple of hours. That still falls into your criteria of a one day admission or less than one day admission, but it wouldn't be counted as an admission on our numbers.

How are admissions recorded in different hospitals and how does this then impact on the quantitative data for zero day admissions?

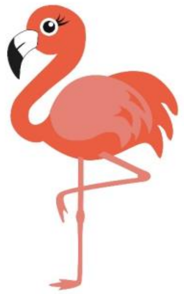

CHIEF SCIENTIST OFFICE

Children going to different areas/ health boards

Doctor

So even the ones that present at hospitals A and B - which is about a two, two-and-a-half hour drive away, ‘cause they're an adult hospital and got no paediatric services, so they see and treat children under the advice from us - … we can VC into them and help them, but if that child needs to be admitted then there's no services elsewhere for them, they have to come down here.

Parent

The medical team in hospital C debated sending Ella (fictitious name) to hospital D and I was surprised that the decision to send her to hospital D wasn’t made sooner. When the Senior Nurse came on shift, things started to move quicker and thankfully the decision was made to send us to hospital D. My husband and daughter left the hospital at 6pm to be taken to the air ambulance to fly to hospital D.

Potential impact on zero day numbers from children having to travel to specialist hospitals outside their own health board?

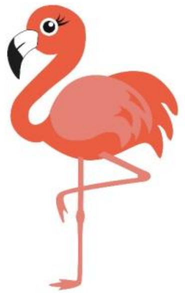

CHIEF SCIENTIST OFFICE

For peer review only - http://bmjopen.bmj.com/site/about/guidelines.xhtml

Resources: people and physical

Doctor

Overnight when a child is admitted we don’t have nursing staff for our observations unit so therefore the child goes straight to inpatients and is admitted as an inpatient overnight, ‘cause generally the majority of them that come in overnight stay for the morning anyway.

Doctor

If I wasn’t busy on an evening, a nightshift, I would wander down to the out-of-hours because it was on the same site and just said, ‘Well look, I'll just come down and see the child there.' And all they wanted was a bit of reassurance that what they were doing was correct and the child was okay.

How do resources impact on whether a child can be managed in that area, or on where a child is sent and therefore how an admission is recorded?

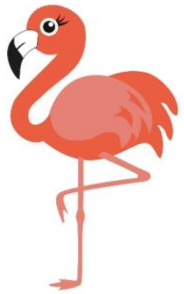

CHIEF SCIENTIST OFFICE

Policies and targets

Consultant

So all of the patients that come through our department are subject to the Scottish Government's unscheduled care four-hour target. So obviously that target does drive our practice and behaviour to a certain extent. We had set up an adjacent area and badged it as a clinical decision unit to essentially allow our department to continue to function with the volume of patients that we expected to allow patients to flow in and flow out of the back, while still obviously being able to meet that target.

GP

They have a rule in the ward in NHS X that they won't keep children longer than 24 hours, that’s their rule. If the child needs to stay in hospital beyond 24 hours the child needs to go to Hospital E.

How do NHS, health board, or hospital targets impact on decisions made about a child’s care?

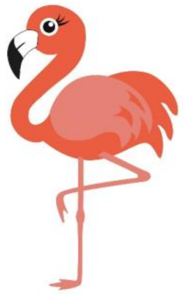

CHIEF SCIENTIST OFFICE

Physical access – primary care

Parent

Where we stay we're kind of out the way a wee bit, and they were saying it's taking ages to get an ambulance … I would've probably taken him in the car and just drove … but yeah public transport would be a no go.

GP

We are quite rural but I think it has an effect of families in two ways; one way the patient is reluctant to do that travel, they'd prefer to stay at home but also it actually means that sometimes we're taking on more risk because if things do go badly it takes longer to get someone into hospital as well.

How does physical access impact on decisions in primary care about whether to refer a child to hospital or treat them in the community?

CHIEF SCIENTIST OFFICE

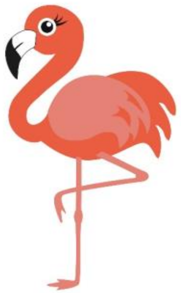

Physical access – secondary care

Consultant

I guess one thing with our geography, so the juniors, whenever they come and ask me advice about kids I'm always, like, 'where do they stay?' and they're, like, 'why does that matter?' but once you get to know our geography - so, for example, it can be over an hour to get back to the hospital, so I would be more likely to bring those kids in, particularly out-of-hours, if they've got croup or bronchiolitis or something, sometimes they'd get watched overnight, whereas if they were very close to the hospital you would send them home.

How does physical access impact on decisions made by hospital staff about whether a child is kept in or discharged home?

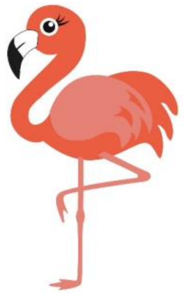

CHIEF SCIENTIST OFFICE

Social issues, including deprivation, and language issues

Consultant

Actually I will definitely be influenced by if they are going home to a dirty home. If they don't have a car then I'm not going to expect them to somehow come in on three buses to come and get an IV antibiotic 'cause it saves me a bed. It does influence your decision making.

Consultant

So there is a lot of deprivation but then you have pockets of really well off communities. So on the one hand you'd have the stereotype of the more deprived family maybe presenting more often, but then you've also got the more entitled wealthier people maybe feeling more entitled and therefore would they present more often.

How do social issues and deprivation/language issues impact on whether or not a child is admitted or discharged home?

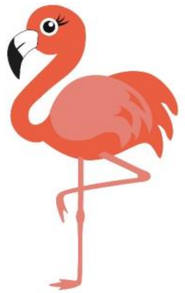

CHIEF SCIENTIST OFFICE

Expectations and experiences of services

Nurse

So I personally believe we're the victim of our own success and with what's happening out in the GP community and things like that, I think we can't turn back time. People do not feel safe and secure being managed out in the community and I think they feel it's safer that they're being assessed in the hospital and that's a feeling they get from the press and from GPs and things like that. I think we need to accept that this is our workload and we should get better at managing it.

Parent

If he was to get that breathless again I would probably phone an ambulance 'cause I probably didn't realise at the time how serious it was…. But we are only along the road so it wasn't as if we're miles away but I probably wouldn't phone 111 the next time, I would just take him in or phone an ambulance.

How and where do families seek healthcare advice and what do they expect from that?

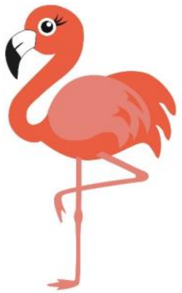

1495 UNIVERSITY OF ABERDEEN

nmahp-ru
Nursing, Midwifery and Allied Health Professions Research Unit
Improving health through research CHIEF SCIENTIST OFFICE

NHS Grampian

UNIVERSITY of STIRLING

Edinburgh Napier UNIVERSITY

GIG CYMRU NHS WALES | Iechyd Cyhoeddus Cymru Public Health Wales

Funded by: CHIEF SCIENTIST OFFICE

[illegible]

1. **What is the most important thing you have heard this morning?**

2. **What surprised you most?**

3. **What did not surprise you?**

See you back here at 1.15 ☺

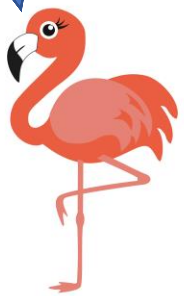

CHIEF SCIENTIST OFFICE

Welcome back

Time for a recap and to welcome new colleagues

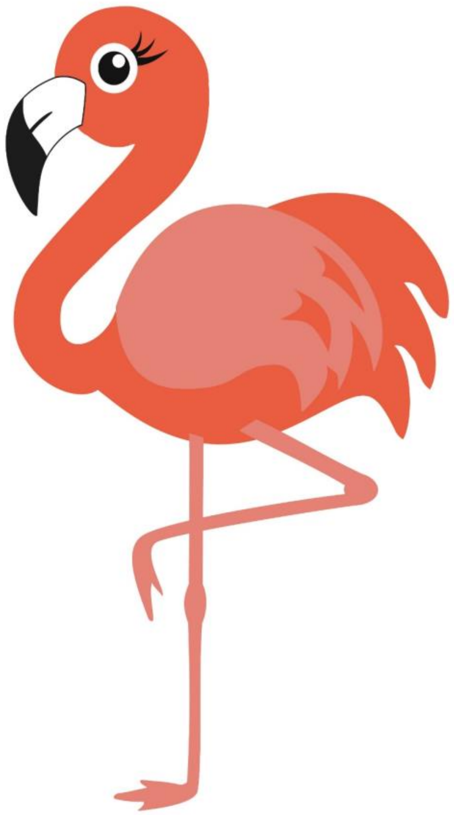

CHIEF SCIENTIST OFFICE

Why are we meeting?

This is stakeholder engagement

Two main aims

1. Share results with you
   - What surprises you
2. What are the priorities?
   - What would you do first?

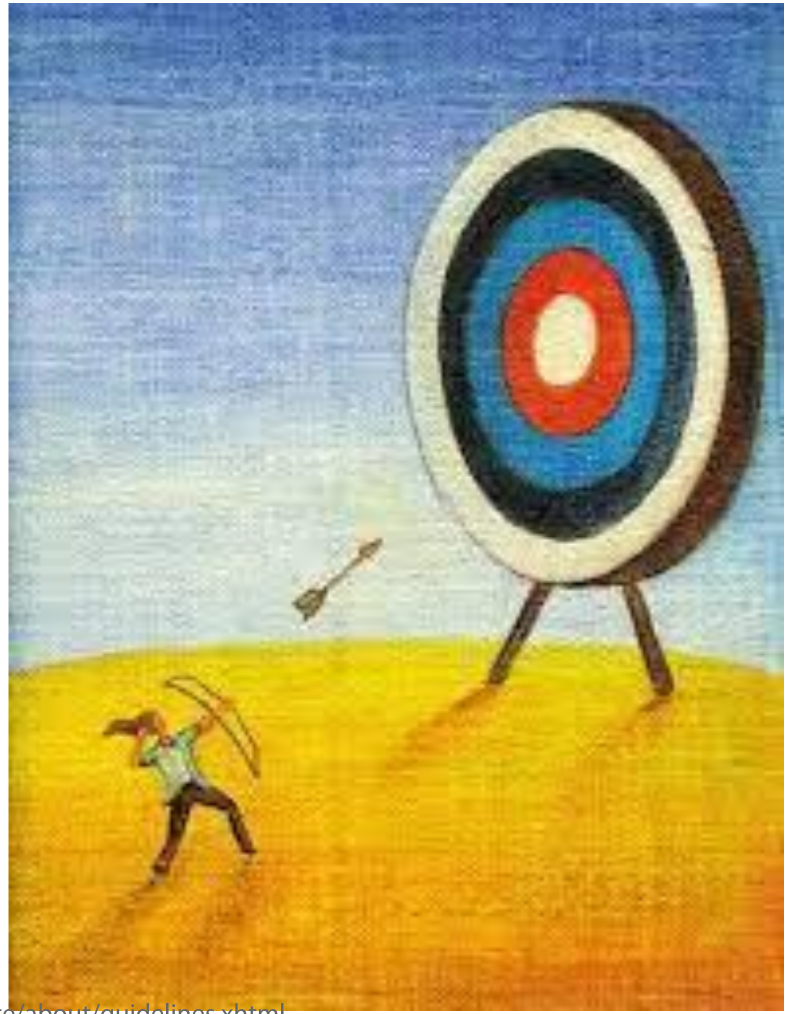

CHIEF SCIENTIST OFFICE

For peer review only - http://bmjopen.bmj.com/site/about/guidelines.xhtml

Introductions

- Who is here today?

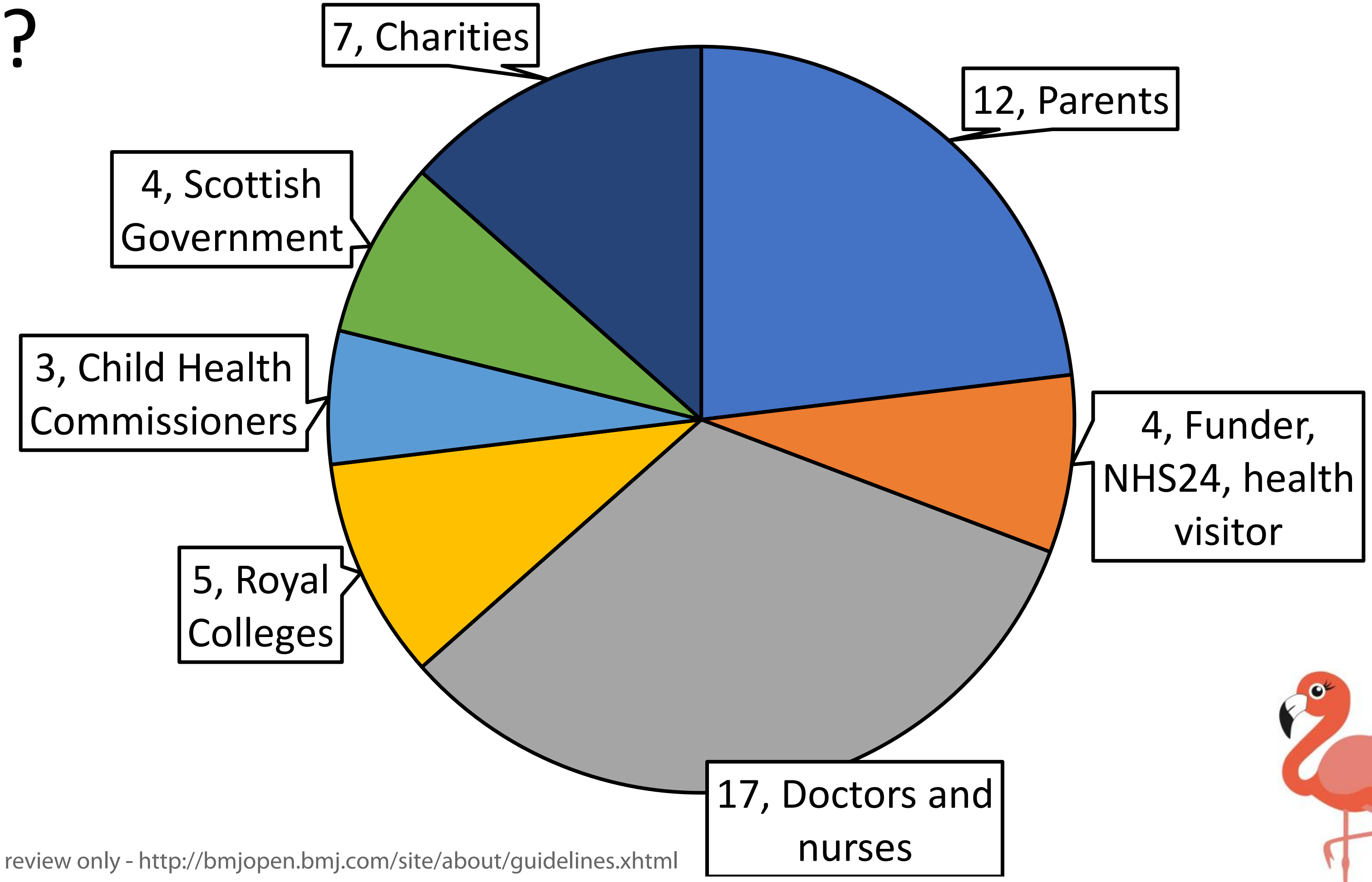

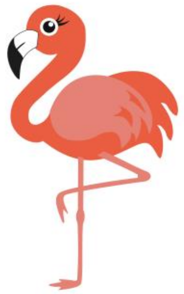

CHIEF SCIENTIST OFFICE

For peer review only - http://bmjopen.bmj.com/site/about/guidelines.xhtml

FLAMINGO

Flow of hospital admissions in children and young people

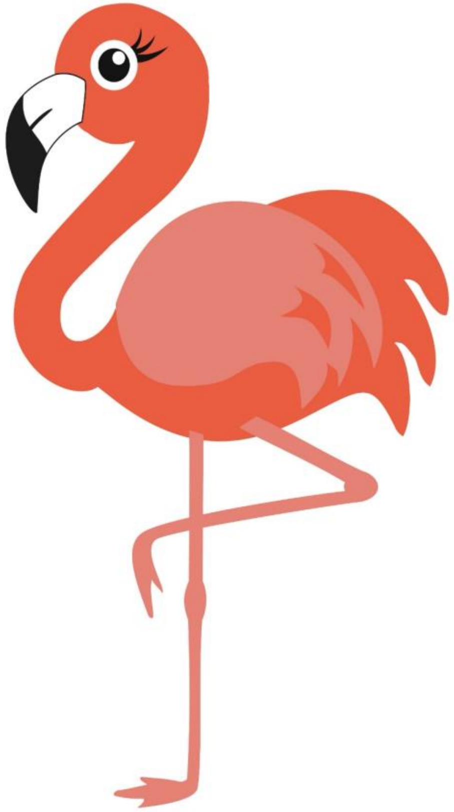

Potential for Innovation and Change

CHIEF SCIENTIST OFFICE

For peer review only - http://bmjopen.bmj.com/site/about/guidelines.xhtml

Practices & approaches tried & deemed successful by interviewees

- Access to specialist paediatric staff to inform decision-making, assessment & management of care for staff with less paediatric experience
- Change layout of Emergency Departments (EDs) to separate infectious & non-infectious patients
- Areas in ED where children can be observed for short periods
- 24-hour open access directly back to paediatric ward for patients following hospital discharge
- Dedicated specialist nursing teams for management of long-term conditions

CHIEF SCIENTIST OFFICE

For peer review only - http://bmjopen.bmj.com/site/about/guidelines.xhtml

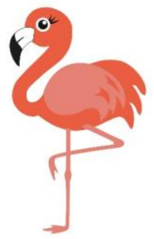

Two levels of change to consider

1. Quality Improvement initiatives within existing systems

- Quicker to implement
- Existing evidence for effectiveness
- ‘Easy wins’ around improving education & communication

2. System or Pathway changes

- Uncertain evidence for its effectiveness
- Required further research & development
- Take longer to implement
- Require significant resources

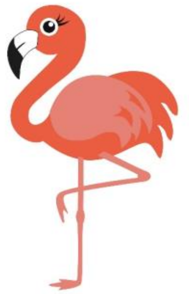

CHIEF SCIENTIST OFFICE

For peer review only - http://bmjopen.bmj.com/site/about/guidelines.xhtml

1. Suggestions for Quality Improvement

- More innovative educational resources – pictures, videos, practical education for common scenarios, websites/social media, including for non-English speakers
- Information sharing activities & social events alongside services where all attend e.g. immunisations
- Improve NHS secondary/primary care/education/social team communication to prevent children falling through the gap
- Access to clinical support for GPs from expert colleagues
- Rotation of GP and hospital trainees/junior staff
- Separate places for infectious/non-infectious admissions
- Fast-track appointment within one week as an alternative to admission
- Evaluate use of video conferencing for patient assessment and triage

CHIEF SCIENTIST OFFICE

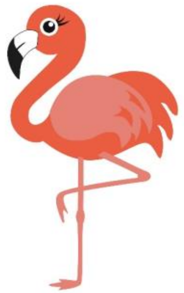

2. System or Pathway Changes

- Gap in acute paediatric nursing and medical skills in the community

- Assessment and observation in community settings
- Holistic children’s hubs
- Hospital at Home model

- Care pathways for specific conditions

CHIEF SCIENTIST OFFICE

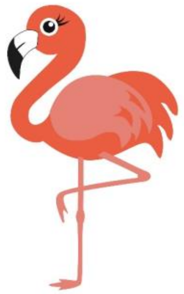

Gap in acute paediatric skills in the community

Hospital Nurse

It might be worth thinking about putting advanced paediatric nurse practitioners in GP surgeries. Again you've got well-experienced paediatric nurses that could go out into the community, see these patients, maybe be able to keep them at home by reassessing during the day, knowing what they're reassessing and also be able to do some teaching with the GPs. I think the way forward is maybe to try and put more paediatric experienced staff out in the community that can see acute unwell children.

What are the opportunities and challenges?

Would this be fair and sustainable for all patients?

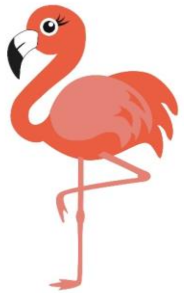

CHIEF SCIENTIST OFFICE

Assessment and observation in community settings

Hospital doctor

It'd be lovely to get more paediatric experience in the peripheries, in which case we might be able to reduce the number of patients that come down to us. And that’s for the parents' benefit as well and the family's benefit as well.

How do we get the right skills at the right time in the right place?

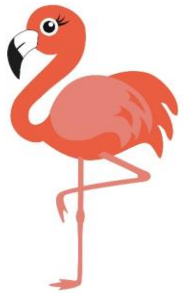

CHIEF SCIENTIST OFFICE

Holistic children’s hubs linked to Children’s Hospital

Hospital Doctor

What I find is that children are born and there's nothing else for children and families apart from a health visitor and, actually, it's almost like if we could have hubs and community hubs.

Hospital Doctor

Having a facility where you can sit and watch them, whether or not you actually do a specific intervention, even if you can just watch them and repeat their observations over an hour or two, we have found that that can help. We had a period of time where one of our consultants went out and spent time in the out-of-hours service and persuaded them to set aside an area in the out-of-hours building where children could wait and be observed. […]. You need space and you need the appropriate staff to look after them, you can't just sit them down and abandon them, so you need staff that are focused on acute assessment and acute treatment, you need a geographical location that allows you to do that.

Infection hubs? Assessment and observation hubs? Integrated holistic Physical, Mental and Social hubs? Range of staff and skills?

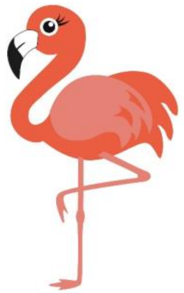

CHIEF SCIENTIST OFFICE

Hospital at Home model

Hospital doctor

So what we've found increasingly is that if there's no disease reason for the child being admitted it's actually do to with social support. And I think because we don’t have the community infrastructures in place to be able to say, ‘You were utterly distraught at two in the morning, we calmed you down, you went home and this person is going to come out to your house to see how you're doing in the day,' because we don’t have that level of sophistication, then often we use the hospital as a safety net.

Logistics of home visits - e.g. in rural areas / winter months / pandemics?

CHIEF SCIENTIST OFFICE

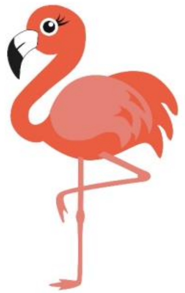

Specialist care pathways?

Parent

999 was the first call, just purely because it was a Sunday evening. Normally his diabetic nurse is absolutely amazing, so if that happened during the week we would phone her and get advice from her.

Parent

I really, really wasn't happy with the way that NHS111 call was handled. I immediately recognised she was having a seizure. The call handler basically made me feel like I was an overreacting mother. When the nurse phoned back was a bit more reassuring, but again, had gone with it being a viral response and I kept on saying 'I don't think it's a viral response.' I didn't even bother phoning 111 [the second time], I just took her straight up to A&E, but because everything was normal by the time we got there, again I was just sent home and kind of made to feel like I was a bit overreacting. So when she finally was seen and diagnosed by the clinician up at the hospital, who's a consultant, he was brilliant, he turned round and gave the diagnosis of it being epilepsy.

Suggested special pathways for admission
Early postnatal pathway for babies under 2 years? Wheezers? Bronchiolitis? Seizure?

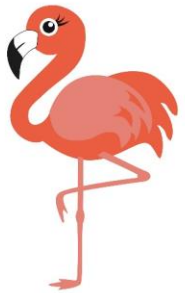

CHIEF SCIENTIST OFFICE

Is there an opportunity for a seizure pre-hospital care pathway?

A Case Study

CHIEF SCIENTIST OFFICE

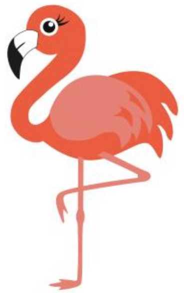

For peer review only - http://bmjopen.bmj.com/site/about/guidelines.xhtml

Characteristics of unscheduled paediatric hospital admissions for febrile and afebrile convulsions

Characteristic	Febrile Convulsion n (%)	Afebrile Convulsion n (%)
Unscheduled Admission	1916 (1.1)*	2661 (1.6)*
Zero Day Admission	976 (51.0)	1344 (51.0)
Sex:		
Female	424 (43.4)	633 (47.1)
Male	552 (56.6)	711 (52.9)
Age at Admission (years):		
< 1	96 (9.8)	131 (9.8)
1-4	818 (83.8)	499 (37.1)
5-9	54 (5.5)	387 (28.8)
10-15	5 (0.5)	323 (24.0)

* % of total emergency medical paediatric admissions to Scottish hospitals between 2015 and 2017 which was 171 039.

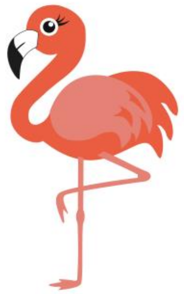

CHIEF SCIENTIST OFFICE

Interviews with health professionals and parents…

Health Care Professionals (HCPs)

- To explore their experiences of and decisions around pre-hospital referrals and subsequent ZDAs of children
- Range of HCPs including ED consultants, nurses, GPs (n=48)

Parents

- To explore their experiences of accessing hospital care resulting in a ZDA
- Range of experiences/conditions but only those focused on convulsions analysed for this presentation (n=4)

Epilepsy Specialist Nurses (ESNs)

- Specialist nursing service involved with and observers of a wide range of child and family experiences and scenarios – well placed to consider ZDAs for convulsions and suggest opportunities for change moving forward
- ESNs representing five Scottish HB areas (n=7)

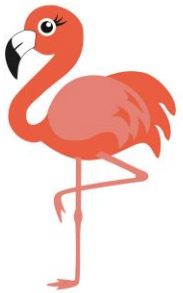

CHIEF SCIENTIST OFFICE

Findings from the interviews...

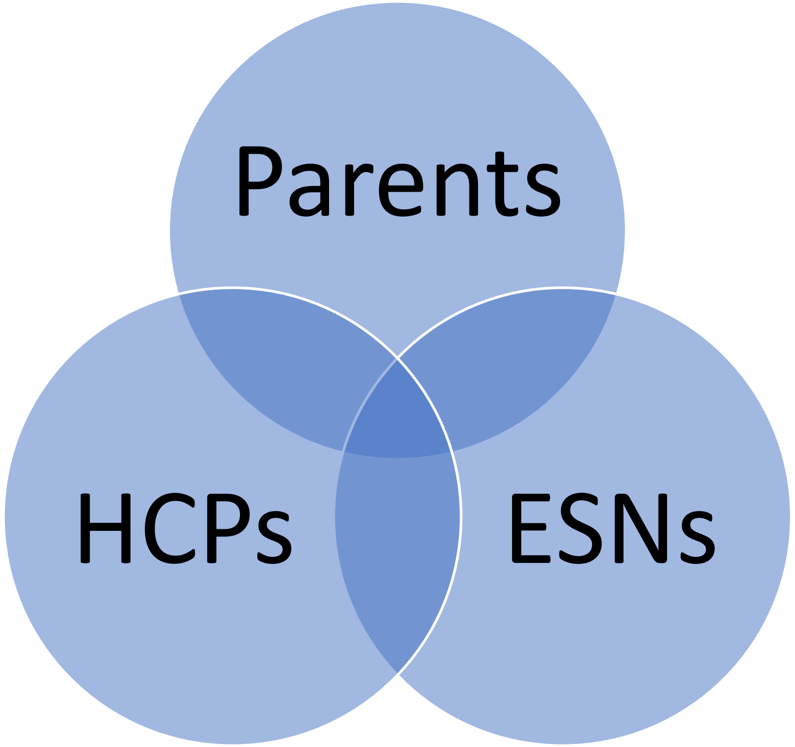

There was consistency and similarity in the themes emerging from interviews with these three different groups.

Analysis and synthesis of the interview data revealed three overarching themes related to experiences of unscheduled hospital attendance or admission of children with seizures

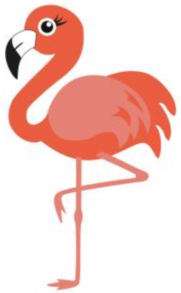

CHIEF SCIENTIST OFFICE

Overarching themes…

**Theme 1:** Anxiety and panic	• The overwhelming feelings of anxiety and panic experienced by parents when their child has a seizure and often contributes to hospital attendance or admission.
**Theme 2:** Reassurance, observation and forward planning	• Reassurance and ongoing support for parents through observation of their child following a seizure (by relevant HCP) and forward planning to diagnose new or manage existing seizures.
**Theme 3:** Care from the right person, in the right place, at the right time	• Care pathways for acute seizure care that ensures care is provided by a professional with knowledge and expertise of seizures in children, in the most appropriate setting and with timely access.

CHIEF SCIENTIST OFFICE

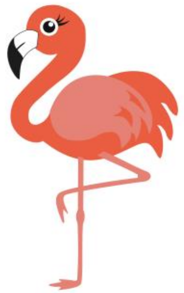

- Formally evaluate best practice around the use of video technology that was accelerated during the COVID-19 pandemic

- Explore the development of more child-oriented algorithms for NHS24 and ambulance service with additional education and training for these professionals.

- Raise awareness of febrile convulsions amongst parents, nurseries and schools, and the wider public.

- Expand the ESN service and nursing staff with expertise around acute seizure assessment and management.

CHIEF SCIENTIST OFFICE

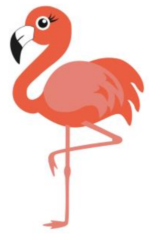

Prioritising potential system innovations…

CHIEF SCIENTIST OFFICE

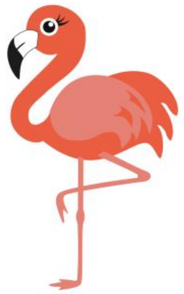

For peer review only - http://bmjopen.bmj.com/site/about/guidelines.xhtml

Prioritising potential system innovations…

- Address gap in acute paediatric skills of healthcare professionals in the community.
- Assessment and observation in community settings
- Holistic children's hubs linked to Children's Hospital
- Hospital at Home model
- Specific care pathways for under twos.
- Specific care pathways for wheezy children/bronchiolitis.
- Specific care pathway for seizure.

CHIEF SCIENTIST OFFICE

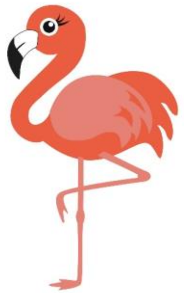

1495 UNIVERSITY OF ABERDEEN

nmahp-ru
Nursing, Midwifery and Allied Health Professions Research Unit
Improving health through research

NHS Grampian

UNIVERSITY of STIRLING

Edinburgh Napier UNIVERSITY

GIG CYMRU NHS WALES
Iechyd Cyhoeddus Cymru
Public Health Wales

Funded by: CHIEF SCIENTIST OFFICE

Questions to consider....

If you were in charge, what would you change first and why?

- Address gap in acute paediatric skills of healthcare professionals in the community
- Assessment and observation of patients in community settings
- Holistic children's hubs linked to Children's Hospital
- Hospital at Home model
- Specific Care Pathway for under 2 year olds
- Specific Care Pathway for wheezy children/bronchiolitis
- Specific Care Pathway for seizure
- Other ..... please state

Imagine you are in charge five year after your first choice has been in action. What problems would people come to you with about your "new" service?

See you back here at 2.45 ☺

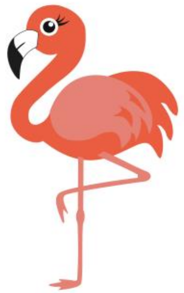

CHIEF SCIENTIST OFFICE

For peer review only - http://bmjopen.bmj.com/site/about/guidelines.xhtml

FLAMINGO
Flow of hospital admissions in children and young people – Wrap up

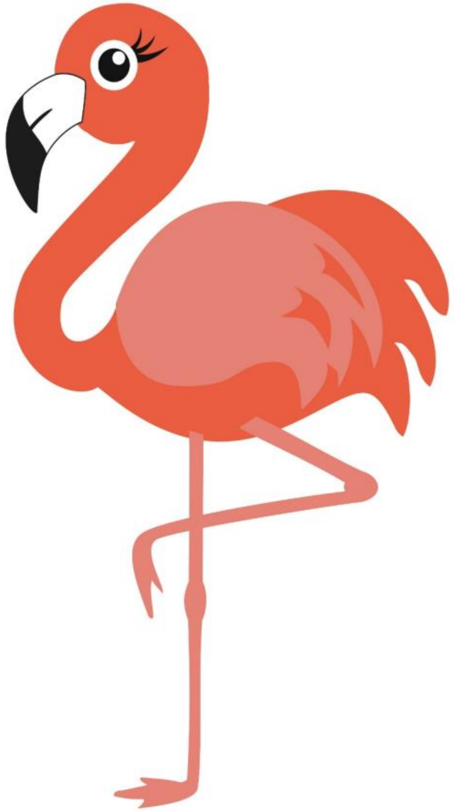

CHIEF SCIENTIST OFFICE

For peer review only - http://bmjopen.bmj.com/site/about/guidelines.xhtml

Why are we meeting?

This is stakeholder engagement

Two main aims

1. Share results with you
   - What surprises you
2. What are the priorities?
   - What would you do first?

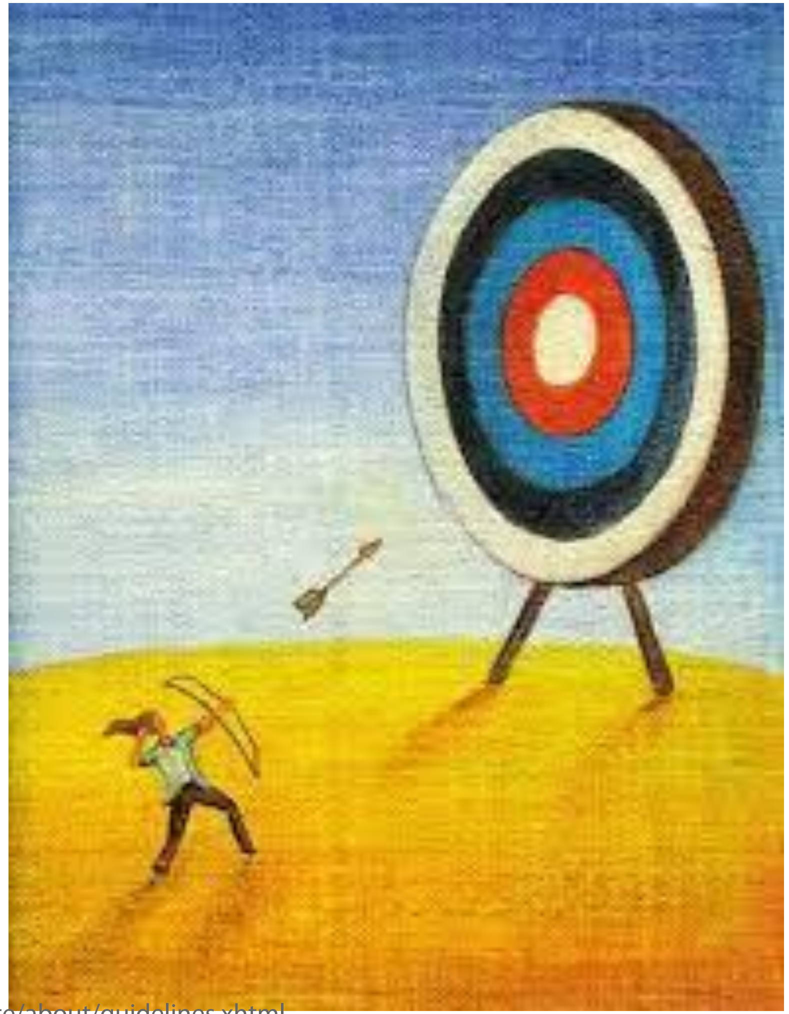

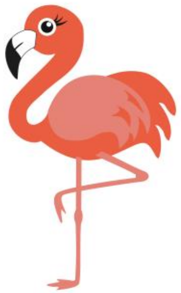

CHIEF SCIENTIST OFFICE

For peer review only - http://bmjopen.bmj.com/site/about/guidelines.xhtml

Why are we meeting?

Please can you find your piece of paper from this morning?

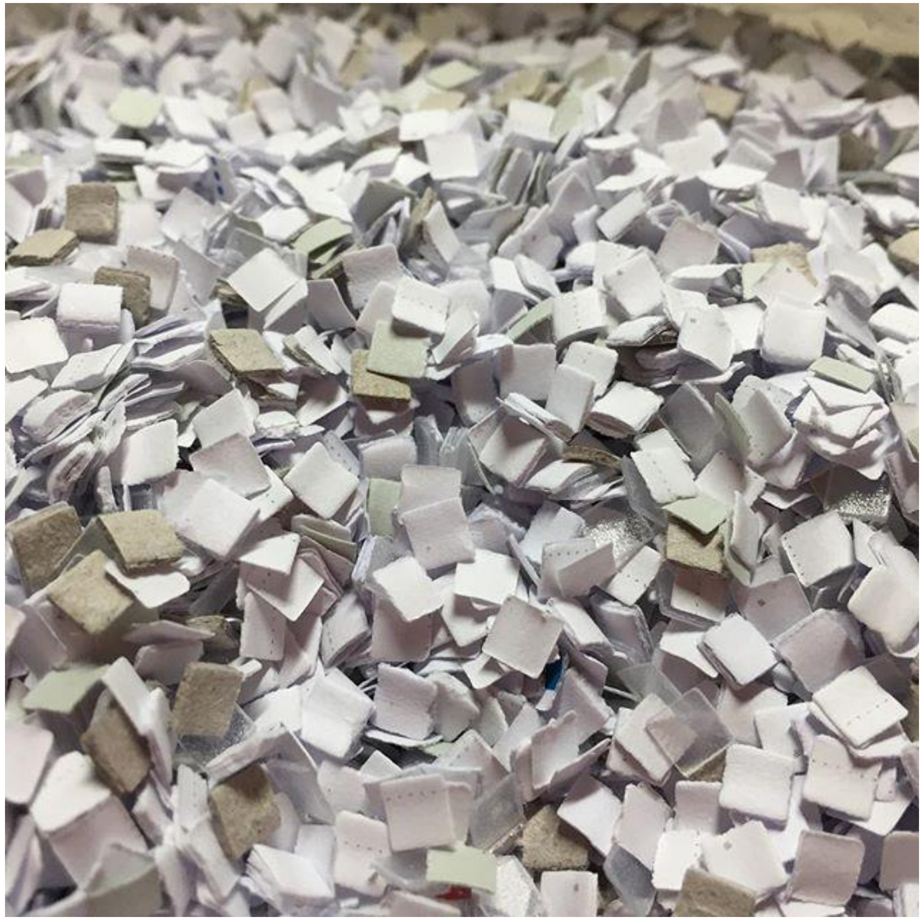

Did you meet that aim?

CHIEF SCIENTIST OFFICE

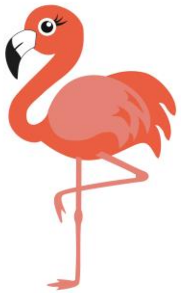

So where next?

- Finalise analysis
  - Quant
  - Qual
- Write papers
  - Overall findings and methodology
  - Regional variation
  - Seizures
  - Post discharge journey
  - Matched case-control

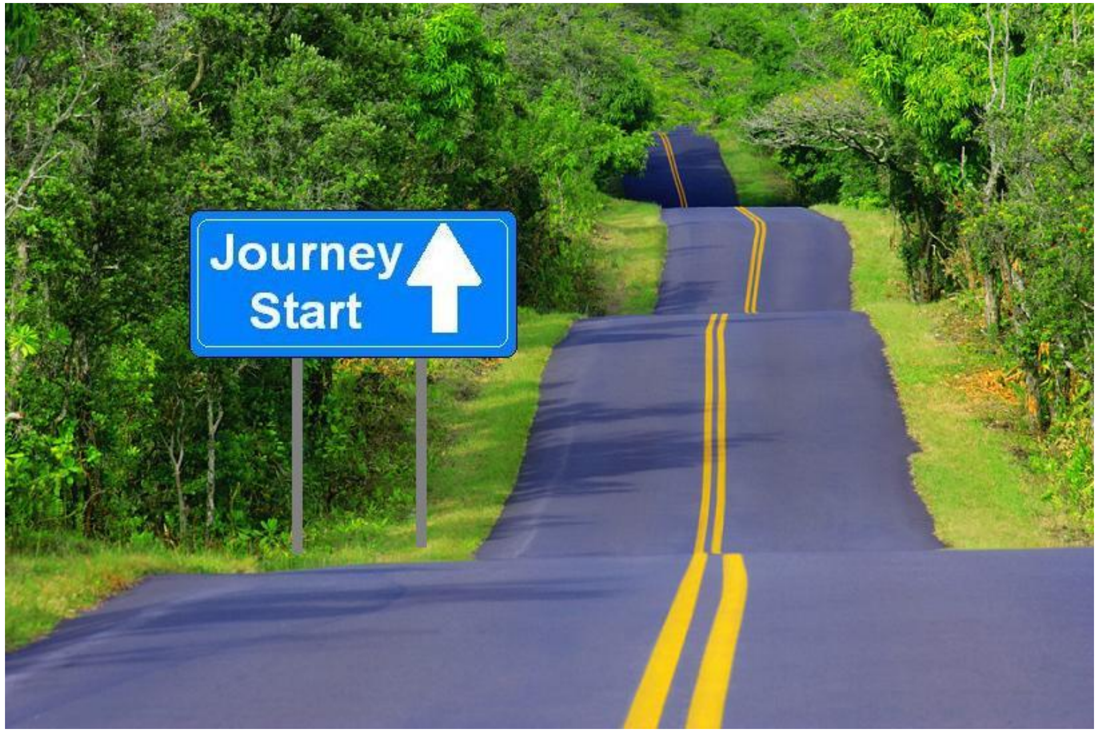

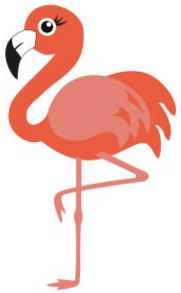

CHIEF SCIENTIST OFFICE

So where next?

Many potential ways to start

- Agreed referral pathways
- Rapid review clinics
- Communication
- More staff
- New buildings
- Etc

Original research

Interventions to reduce acute paediatric hospital admissions: a systematic review

Smita Dick ,[1] Clare MacRae,[2] Claire McFaul,[1] Usman Rasul,[1] Philip Wilson ,[3] Stephen W Turner [1]

What this study adds

- Since a review of this literature published in 2012, there has been a considerable increase in the number of publications describing interventions aimed at reducing unscheduled admissions.
- Despite these welcome additions to the literature, the evidence available lack robust studies (eg, randomised controlled trials) and is mostly based on single-centre experience.
- There is a need for multifaceted and multicentred interventions using resources from health (primary and secondary care) and social care to tackle the increasing admissions.

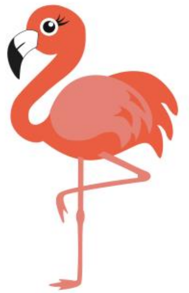

CHIEF SCIENTIST OFFICE

For peer review only - http://bmjopen.bmj.com/site/about/guidelines.xhtml

Thank you all very much

- Could not have done this without you
- Post meeting questionnaire on its way
- Have a fun weekend

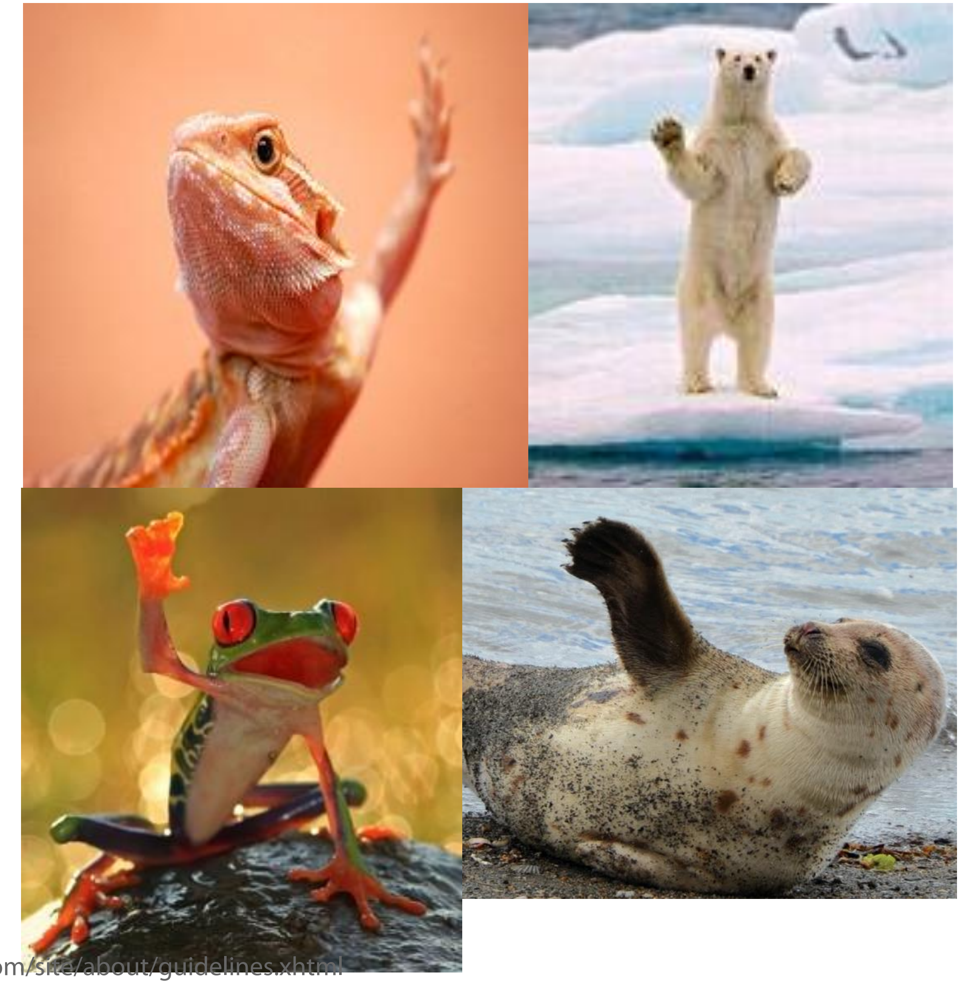

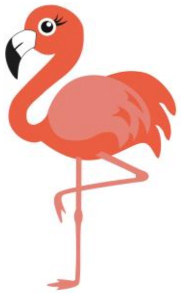

CHIEF SCIENTIST OFFICE

For peer review only - http://bmjopen.bmj.com/site/about/guidelines.xhtml

Supplementary file 7. Characteristics of qualitative interview participants

Full data available in: Malcolm C, King E, France E, et al. Short stay hospital admissions for an acutely unwell child: A qualitative study of outcomes that matter to parents and professionals. *PLoS One.* 2022 Dec 16;17(12):e0278777. doi: 10.1371/journal.pone.0278777. PMID: 36525432; PMCID: PMC9757586.

**Parents and children (n=21)**	
**Characteristic**	**Number (n)**
Parent's relationship to child:	
Mother	20
Father	1
Gender of child:	
Female	5
Male	16
Age of child at time of admission:	
0-5 years	16
6-10 years	4
11-16 years	1
Average SIMD 2020 Decile (range)	5.1 (1-10)
Reason for admission (infection vs non-infection):	
Infection	15
Non-infection	6
Time elapsed since child's admission and the date of interview (months):	
0-24	17
>24	2
Missing data	2
Admission was pre-COVID (pre-March 2020):	
Yes	7
No	14
**Health Professionals (n=48)**	
**Role and Setting**	
**Primary Care/General Practice/OOH Service**	16
**Paediatric Hospital Emergency Department**	18
**District General Hospital Emergency Department**	14
Length of experience (years)	
<5	3
5-30	34
>30	4
Missing data	7

For peer review only

Full supporting interview quotations for each intervention

Addressing gaps in acute paediatric skills of health professionals working in community settings
**Increase specialist acute paediatric nursing roles**
It might be worth thinking about putting ANPPs in GP surgeries. Again, you've got well-experienced paediatric nurses that could go out into the community, see these patients, maybe be able to keep them at home by reassessing during the day, knowing what they're reassessing and also be able to do some teaching with the GPs. I think the way forward is maybe to try and put more paediatric experienced staff out in the community that can see acute unwell children. (C003_Nurse)
I think the emergency department was very busy, we were lucky to get some attention and we were moved fairly promptly down to the children's ward where we had…, it was clearly, [sic]. We were getting more specialist advice and you got much more attention down there, and they were able to fit the nebuliser properly onto my son's face which they hadn't really done in the ED. (Parent P002)
**Provide additional paediatric urgent care training for GPs**
Yeah, it's also unfortunate but true that paediatrics is not a requirement for training in general practice. So, you will unfortunately get, it's sad to say but the quality of referrals from primary care can be very poor. So, you have practitioners who are not prepared to take any chances themselves because of a lack of experience and this is a big problem for us in the winter months. (C014_Consultant)
I think training the GPs and the OOHs staff to have more experience and more confidence in paediatric problems should theoretically help but actually practically doing that is very, very difficult to get a significant amount of teaching and learning across to the body of individuals that work in primary care, so it's challenging, it's very difficult. (C005_Consultant)
I did quite a lot of paediatric training here, I didn't do any psychiatry […] but I managed to muddle through with psychiatry over the years but the paediatric training I felt was pretty essential […]. They [GP trainees] can breeze through without anything formally and paediatrics, you know, there's an enormous amount of pitfalls there with paediatrics. (C038_GP)

Then she [GP] went to do his SATS, so she had one of those little probes, sort of like a completely mobile standalone thing and he wriggled and fussed […] she did take it several times although my concern was it was an adult probe, so I was thinking it's probably not particularly accurate, but anyway she did try several times, she was only getting 86 and she did seem to have a good trace getting 86. […] So [GP] phoned [hospital] from the GP surgery and said 'I have this boy, he's known to you, I can only get SATS of 86, are you happy for him to come in?' So they said 'yes but he needs to come in an ambulance if his SATS are 86, he needs oxygen'. […] They [hospital] put a SATS monitor on him but for a small child and his SATS were like 99/100, so I think we were all like 'oh god, this seems such a waste of everyone's time and resources!' (Parent P010)
**Create rotational posts between primary and secondary care settings**
Part of my role when they took me on in post […] was going to be facilitation of learning and development of other people's skills. So, because COVID came in we like every other service had to set up a COVID assessment centre […]. So again, these were adult trained clinicians that came in and didn't have any paediatric experience, so they were heavily relying on GP support within the in-hours period if children were referred […]. So for a wee while as the schools and nurseries all started to go back we had a bit of a boom in paediatric presentations and I actually came out of my out of hours post for a period of four weeks to support in-hours so that there was somebody there that could see the children, so that there was somebody there that could if the nurse practitioners wanted to come in and shadow. I did a little bit of just ad hoc teaching there. […] So it is something that I have tried to look into and I am intermittently just trying to push on and do little bits of ad hoc education as and when I can but, like I say, unfortunately COVID impacted all of our plans for pushing forward with that. […]. I suppose I'm quite lucky that I have got established relationships with the paediatric ward because that's where I came from. So I do have an established support network if you like, and established relationships. (C027_Nurse)
Yeah I'm a GP […]I also am piloting at the moment a joint clinic with a couple of the consultant paediatricians […]. We have a community child health consultant once a month and a general medical consultant once a month that we co-consult with on some more kind of challenging cases to try and prevent access issues and things that can potentially be dealt with in primary care. […]. I also do out of hours, so occasionally work in Hospital6-1 as a GP, but seeing the stuff that gets triaged from Hospital6-1 as being more GP stuff, so you know, it's often viral things and things like that, so I do that as well. (C031_GP)
**Assessment and observation over time in community settings**
Sometimes very obviously the correct thing to do is not to discharge the child but to give a slightly longer period of observation. (C018_Consultant)

I think the main thing with a lot of these kids is actually just time and giving them a chance to let their anti-pyretic settle and giving them a bit of fluid and just a period of observation, which I think is the limiting factor in GP practices and in GP out of hours, you know, they just don't have the facilities to be able to watch these children for a period of time. (C015_Doctor)
They [children] can't tell you what's wrong, you know, so you might just have a baby that's a bit fractious or maybe not feeding as well, really soft signs, and I don't think we have the facilities even really to get all the accurate measurements that we would want, we don't have a paediatric SATS monitor, we can't do blood pressure in children either, so we're basing things more on their heart rate and their temperature and it's maybe kind of soft but just the general way they are. (C013_GP)
**Creation of holistic children's 'hubs'**
What I find is that then these children are born and there's nothing else for children and families apart from a health visitor and actually, you know, it's almost like if we could have hubs and community hubs where if you come in to get our health visiting weighed and things like that, you get taught about childhood diseases and when to worry, you know, and so almost like a mass public education programme that you get taught about when to worry, about when your child is sick. (C002_Consultant)
Interviewer: And how did you feel about taking him home [from hospital] at that point, did you feel fairly confident that you could look after, you know, if it was just bronchiolitis that you could look after that?<br>*Respondent: No, not at all [laugh] no I wasn't, no, I wasn't confident. I mean, he's a baby with the cold and I'll have to manage it but at that point he still wasn't feeding well, I probably wasn't very well at that point, yeah I was mentally not very well at that point and did not feel equipped to look after him at home, but then I guess I didn't know what else they could realistically do for him in hospital. So I was accepting of it.* [Parent P018]
Having a facility where you can sit and watch them, whether or not you actually do a specific intervention, even if you can just watch them and repeat their observations over an hour or two, we have found that that can help. We had a period of time where one of our consultants went out and spent time in the out-of-hours service and persuaded them to set aside an area in the out-of-hours building where children could wait and be observed. […]. You need space and

For peer review only

you need the appropriate staff to look after them, you can't just sit them down and abandon them, so you need staff that are focused on acute assessment and acute treatment, you need a geographical location that allows you to do that. (C005_Consultant)
**Hospital at Home model**
It's ebbs and flows and there are periods of times when you seem to get a number of referrals which you think, 'Surely that could be handled in the community, or can be managed in a different way rather than coming into hospital,' yeah. As to whether they could've handed in their urine sample of something, went away and then you can advise on what they're doing at home and representing, you know, safety-netting and so on. Or there are certain things where you think actually the best way to handle the particular scenario would've been to speak to someone who actually… rather than bring them in the night - and that happens fairly often, I would think, where a referral is received at five o'clock in the evening or something along those lines, and you know that they need some investigation or imaging that isn't going to happen that night or they're going to need to see, for instance, a child and adult mental health person, and that's not going to happen in the middle of the night - and you think, actually, that child could potentially be risk assessed and managed at home and then referred to the appropriate services the next day. (C020_Consultant)
The 111 nurse [NHS24]*, she was just [laugh], I think because I was obviously slightly panicked because he was bringing up foam and he was unable to breathe properly, so I was obviously like 'oh my gosh he's not breathing properly' and she was like 'calm down' and I was like 'aargh!' [laugh]. So I think, yeah, the 111 nurse [laugh] probably thought I was a completely over-anxious mum, but this was the worst that he's had the croup so I'd never seen him that poorly apart from when he was on neonatal, so I think it probably brought back some memories and I probably did panic. But the clinicians at the hospital were great, I mean, I explained to the doctor that I didn't want him catching anything and she was lovely and very understanding and said 'we'll try and send you home as soon as we can'.* (Parent P007)
**Extend specialised care pathways for sub-groups of children**
There are specialist nurses for most specialties, so there's specialist nurses for diabetes, specialist nurses for oncology, specialist nurse for cystic fibrosis, asthma, so […] different specialist nurses who are usually the first contact for such parents. […] So, yeah, these long-term conditions, we have changed completely the way we do it. (C010_Consultant)

For peer review only

Yeah, well again if they have a diagnosis of epilepsy and they've got a paediatric epilepsy nurse specialist in their area I think it's pretty straightforward for them, they have a clear plan of what to do and who to contact. As I say, my job is to keep them out of hospital so in between clinic appointments they would be phoning myself if they have a seizure, they're advised to phone myself, I'm a nurse prescriber so I change medications over the phone. A medication is never just prescribed without a clear pathway of how to increase it, when to increase it, what the maximum dose is […] so they [parents] always know that they can do something because it's mainly the helplessness that causes anxiety with epilepsy and the parents not being able to have control of the situation, whereas if you're giving them a wee bit of control of what to do and you give them permission to increase the medicine with a clear plan. I definitely find that cuts down the amount of phone calls you get, it cuts down the anxiety. (C041_Epilepsy Specialist Nurse)
Fevers in young children, vomiting in young children definitely are huge areas where we get lots and lots of referrals. Children under three months with a temperature, absolutely right to refer because it could be a septic baby but, like a two- or three-year-old who has had a temperature for 24 hours it's giving them the right advice by the GP that they can have Paracetamol and Ibuprofen […]. So I think fever in young children, vomiting in young children and respiratory illnesses. (C003_Nurse)

For peer review only

GUIDED – a guideline for reporting for intervention development studies.

Supplementary File 1: Blank Checklist

Item description	Explanation	Page in manuscript where item is located	Other*
1. Report the context for which the intervention was developed.	Understanding the context in which an intervention was developed informs readers about the suitability and transferability of the intervention to the context in which they are considering evaluating, adapting or using the intervention. Context here can include place, organisational and wider socio-political factors that may influence the development and/or delivery of the intervention (15).	4,5	
2. Report the purpose of the intervention development process.	Clearly describing the purpose of the intervention specifies what it sets out to achieve. The purpose may be informed by research priorities, for example those identified in systematic reviews, evidence gaps set out in practice guidance such as The National Institute for Health and Care Excellence or specific prioritisation exercises such as those undertaken with patients and practitioners through the James Lind Alliance.	4,5	
3. Report the target population for the intervention development process.	The target population is the population that will potentially benefit from the intervention – this may include patients, clinicians, and/or members of the public. If the target population is clearly described then readers will be able to understand the relevance of the intervention to their own research or practice. Health inequalities, gender and ethnicity are features of the target population that may be relevant to intervention development processes.	6-8	
4. Report how any published intervention development approach contributed to the development process	Many formal intervention development approaches exist and are used to guide the intervention development process (e.g. 6Squid (16) or The Person Based Approach to Intervention Development (17)). Where a formal intervention development approach is used, it is helpful to describe the process that was followed, including any deviations. More general approaches to intervention development also exist and have been categorised as follows (3):- Target Population-centred intervention development; evidence and theory-based intervention development; partnership intervention development; implementation-based intervention development; efficacy-based intervention development; step or phased-based intervention development; and intervention-specific intervention development (3). These approaches do not always have specific guidance that describe their use. Nevertheless, it is helpful to give a rich description of how any published approach was operationalised	6,7	
5. Report how evidence from different sources informed the intervention development process.	Intervention development is often based on published evidence and/or primary data that has been collected to inform the intervention development process. It is useful to describe and reference all forms of evidence and data that have informed the development of the intervention because evidence bases can change rapidly, and to explain the manner in which the evidence and/or data was used. Understanding what evidence was and was not available at the time of intervention development can help readers to assess transferability to their current situation.	8 +Table1	
6. Report how/if published theory informed the intervention development process.	Reporting whether and how theory informed the intervention development process aids the reader's understanding of the theoretical rationale that underpins the intervention. Though not mentioned in the e-Delphi or consensus meeting, it became increasingly apparent through the development of our guidance that this theory item could relate to either existing published theory or programme theory	6	
7. Report any use of components from an existing intervention in the current intervention development process.	Some interventions are developed with components that have been adopted from existing interventions. Clearly identifying components that have been adopted or adapted and acknowledging their original source helps the reader to understand and distinguish between the novel and adopted components of the new intervention.	7	
8. Report any guiding principles, people or factors that were prioritised when making decisions during the intervention development process.	Reporting any guiding principles that governed the development of the application helps the reader to understand the authors' reasoning behind the decisions that were made. These could include the examples of particular populations who views are being considered when designing the intervention, the modality that is viewed as being most appropriate, design features considered important for the target population, or the potential for the intervention to be scaled up.	8	

For peer review only - http://bmjopen.bmj.com/site/about/guidelines.xhtml

Duncan E, *et al. BMJ Open* 2020; 10:e033516. doi: 10.1136/bmjopen-2019-033516

Item description	Explanation	Page in manuscript where item is located	Other*
9. Report how stakeholders contributed to the intervention development process.	Potential stakeholders can include patient and community representatives, local and national policy makers, health care providers and those paying for or commissioning health care. Each of these groups may influence the intervention development process in different ways. Specifying how differing groups of stakeholders contributed to the intervention development process helps the reader to understand how stakeholders were involved and the degree of influence they had on the overall process. Further detail on how to integrate stakeholder contributions within intervention reporting are available (19).	5-6 +Table3	
10. Report how the intervention changed in content and format from the start of the intervention development process.	Intervention development is frequently an iterative process. The conclusion of the initial phase of intervention development does not necessarily mean that all uncertainties have been addressed. It is helpful to list remaining uncertainties such as the intervention intensity, mode of delivery, materials, procedures, or type of location that the intervention is most suitable for. This can guide other researchers to potential future areas of research and practitioners about uncertainties relevant to their healthcare context.	20	
11. Report any changes to interventions required or likely to be required for subgroups.	Specifying any changes that the intervention development team perceive are required for the intervention to be delivered or tailored to specific sub groups enables readers to understand the applicability of the intervention to their target population or context. These changes could include changes to personnel delivering the intervention, to the content of the intervention, or to the mode of delivery of the intervention.	21	
12. Report important uncertainties at the end of the intervention development process.	Intervention development is frequently an iterative process. The conclusion of the initial phase of intervention development does not necessarily mean that all uncertainties have been addressed. It is helpful to list remaining uncertainties such as the intervention intensity, mode of delivery, materials, procedures, or type of location that the intervention is most suitable for. This can guide other researchers to potential future areas of research and practitioners about uncertainties relevant to their healthcare context.	20	
13. Follow TIDieR guidance when describing the developed intervention.	Interventions have been poorly reported for a number of years. In response to this, internationally recognized guidance has been published to support the high quality reporting of health care? interventions[5]and public health interventions[14]. This guidance should therefore be followed when describing a developed intervention.	4 Followed INDEX	
14. Report the intervention development process in an open access format.	Unless reports of intervention development are available people considering using an intervention cannot understand the process that was undertaken and make a judgement about its appropriateness to their context. It also limits cumulative learning about intervention development methodology and observed consequences at later evaluation, translation and implementation stages. Reporting intervention development in an open access (Gold or Green) publishing format increases the accessibility and visibility of intervention development research and makes it more likely to be read and used. Potential platforms for open access publication of intervention development include open access journal publications, freely accessible funder reports or a study web-page that details the intervention development process.		

*e.g. if item is reported elsewhere, then the location of this information can be stated here.

For peer review only

Duncan E, et al. BMJ Open 2020; 10:e033516. doi: 10.1136/bmjopen-2019-033516
